# A Bright Ratiometric Dipyrene Probe for Functional Imaging of Condensate Microenvironments in Living Cells

**DOI:** 10.1002/advs.76419

**Published:** 2026-07-16

**Authors:** Koki Matsumoto, Sora Kitai, Yoshio Nishiyama, Shogo Amemori, Kei Makiyama, Dini Kurnia Ikliptikawati, Kentaro Ohira, Yuta Kozuka, Maho Tobita, Shih‐Cheng Chen, Chien‐Hung Yu, Wei‐Min Liu, De‐Chen Lin, Motohiro Mizuno, Kazuma Ogawa, Koichi Ogami, Kenji Takahashi, Hiroshi I. Suzuki, Richard W. Wong, Takahiro Soeta, Masaharu Hazawa

**Affiliations:** ^1^ Division of Transdisciplinary Sciences Graduate School of Frontier Science Initiative Kanazawa University Kanazawa Ishikawa Japan; ^2^ Division of Material Sciences Graduate School of Natural Science and Technology Kanazawa University Kanazawa Japan; ^3^ Institute for Frontier Science Initiative Kanazawa University Kanazawa Ishikawa Japan; ^4^ NanoMaterials Research Institute Kanazawa University Kanazawa Japan; ^5^ School of Chemistry College of Science and Engineering Kanazawa University Kanazawa Japan; ^6^ Division of Biological Science and Technology Graduate School of Natural Science and Technology Kanazawa Ishikawa Japan; ^7^ National Institute of Cancer Research National Health Research Institutes Tainan City Taiwan; ^8^ Department of Biochemistry and Molecular Biology College of Medicine National Cheng Kung University Tainan City Taiwan; ^9^ Institute of Basic Medical Sciences, College of Medicine National Cheng Kung University Tainan City Taiwan; ^10^ Department of Chemistry Fu Jen Catholic University New Taipei City Taiwan; ^11^ Center for Craniofacial Molecular Biology Herman Ostrow School of Dentistry University of Southern California Los Angeles California USA; ^12^ Norris Comprehensive Cancer Center University of Southern California Los Angeles California USA; ^13^ Graduate School of Medical Sciences Kanazawa University Kanazawa Ishikawa Japan; ^14^ Division of Molecular Oncology Center for Neurological Diseases and Cancer Nagoya University Graduate School of Medicine Nagoya Aichi Japan; ^15^ Institute for Glyco‐Core Research (iGCORE) Nagoya University Nagoya Aichi Japan; ^16^ Center for One Medicine Innovative Translational Research (COMIT) Nagoya University Nagoya Aichi Japan; ^17^ Inamori Research Institute for Science (InaRIS) Kyoto Japan; ^18^ WPI Nano Life Science Institute Kanazawa University Kanazawa Ishikawa Japan

**Keywords:** biomolecular condensates, dipyrene probe, live‐cell imaging, physicochemical fingerprints, ratiometric imaging

## Abstract

Biomolecular condensates exhibit spatially heterogeneous microenvironments shaped by intertwined physicochemical factors, yet practical small‐molecule tools for mapping these states in living cells remain limited. Here, we report PyLUMI, a bright dipyrene ratiometric probe, optimized by linker engineering to improve fluorescence output under aqueous conditions while preserving excimer‐to‐monomer (E/M) readout capability. Solution‐phase characterization identifies a scaffold with enhanced fluorescence efficiency and an E/M response to coupled polarity–viscosity microenvironmental changes. In recombinant protein condensates, PyLUMI provides substantially increased signal and enables pixel‐resolved visualization of intra‐condensate heterogeneity. In living cells, the probe enables low‐exposure ratiometric imaging of centrosome‐associated condensate compartments and reveals microenvironmental signatures linked to cell‐cycle progression and pathology‐associated centrosome amplification beyond morphological size differences. These results establish PyLUMI as a functional chemical reporter for spatially resolved profiling of integrated physicochemical fingerprints in condensate‐associated compartments.

## Graphical abstract

Ratiometric dipyrene imaging resolves monomer (M) and excimer (E) channels and computes pixel‐wise E/M maps within biomolecular condensates, yielding physicochemical fingerprints shaped by coupled polarity‐related solvation and microviscosity. These spatial fingerprints enable quantitative phenotyping of cellular states, exemplified by separation of interphase and mitotic centrosomes in UMAP, and provide a general workflow for imaging‐based classification.

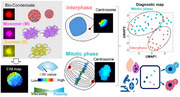

## 1 Introduction

Biomolecular condensates (membrane‐less organelles) are dynamic assemblies that can arise through liquid–liquid phase separation (LLPS) of proteins and nucleic acids [1, 2]. By enriching specific biomolecules in space and time, condensates organize diverse cellular processes, including gene regulation, signaling, and stress responses [1, 2]. Prominent examples include nucleoli, nuclear speckles, stress granules, and centrosomes, and transcriptional coactivators such as BRD4/Mediator can form condensate‐like assemblies linked to super‐enhancer function [3]. Aberrant condensation has been implicated in aging and disease contexts, motivating quantitative approaches that can interrogate condensate states in living cells [4].

Beyond composition and assembly, condensate “state” is often discussed in terms of material behavior and local physicochemical microenvironments that shape molecular mobility, partitioning, and reactivity [2]. However, robust tools to quantify such local states in situ remain limited. FRAP and related analyses are widely used to extract recovery timescales, but these kinetics typically report an effective mobility that conflates multiple processes (diffusion, binding/reaction, and exchange with surrounding pools) rather than a single, transferable material parameter [5, 6]. In phase‐separated assemblies, recovery is further influenced by droplet size and geometry, the bleached fraction, and boundary conditions, making quantitative comparisons across condensates and experimental configurations strongly model‐ and protocol‐dependent [5]. Consequently, FRAP can serve as a useful proxy for dynamics but may be insufficient to resolve spatially heterogeneous microenvironments within individual condensates.

**FIGURE 1:**
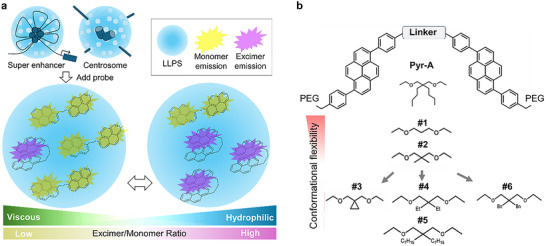
Conceptual basis and linker‐engineering strategy for dipyrene ratiometric physicochemical fingerprinting. (a) Schematic illustrating how a dipyrene reporter yields distinct monomer (M) and excimer (E) emissions whose intensity ratio (E/M) reflects local physicochemical microenvironments. Changes in local microviscosity and polarity‐related solvation modulate the intramolecular encounter probability of the pyrene pair, resulting in characteristic E/M fingerprints within condensed versus dilute phases. (b) Linker‐engineering strategy used to tune excimer–monomer balance without changing the pyrene chromophore. A series of Pyr‐A–derived analogues with varied linker flexibility and steric/hydrophobic constraints was synthesized to systematically modulate intramolecular geometry and optimize aqueous brightness and microenvironmental responsiveness.

Small‐molecule fluorescent probes provide an orthogonal strategy that is readily deployable and chemically tunable, enabling minimally perturbative readouts of local physicochemical microenvironments [7]. Ratiometric probes are particularly advantageous because they provide internal self‐calibration, as previously reported (Figure 1a); pyrene excimer/monomer dual emission offers a compact chemical basis for ratiometric normalization [8]. Importantly, in aqueous and cellular environments, polarity‐related solutions and microviscosity typically change together rather than independently, so isolating a single target property in situ is inherently challenging. As a result, many environment‐sensitive fluorophores should be interpreted as reporting integrated physicochemical fingerprints shaped by viscosity, polarity (often proxied by dielectric constant), and hydration/solvation, rather than a uniquely defined material parameter [9, 10]. This challenge becomes acute in condensates, where partitioning‐driven enrichment and optical heterogeneity can confound intensity‐based readouts. Thus, the goal is not to measure viscosity or polarity in isolation but to obtain an internally normalized ratiometric fingerprint of the local microenvironment. In this framework, the excimer‐to‐monomer (E/M) ratio helps reduce the influence of probe concentration and acquisition‐related intensity variations, while reporting an integrated physicochemical fingerprint shaped by coupled viscosity, polarity, and solvation. Ratiometric strategies therefore offer a useful self‐normalizing approach for condensate imaging [11].

Here we optimize a dipyrene ratiometric reporter and implement a two‐tier in vitro benchmarking workflow prior to cellular application. The resulting analogue enables ratiometric mapping and microenvironment‐informed phenotyping of centrosome‐associated condensates across the cell cycle, including a pathology‐associated model of centrosome amplification.

## 2 Results

### 2.1 Rational Linker Engineering Targets Excimer–Monomer Balance and Aqueous Fluorescence Efficiency

The distance and relative orientation between the two pyrene moieties—whether they can approach to form an excimer or remain sufficiently separated to emit as monomers—are governed by linker flexibility and geometry. We therefore hypothesized that systematic linker engineering would tune intramolecular encounter probability and thereby modulate the E/M readout of condensate microenvironments. In addition to tuning E/M behavior, our first‐generation dipyrene probe Pyr‐A is limited by relatively low fluorescence efficiency in aqueous environments [8], motivating analogue designs that improve photon output in water while preserving the ratiometric excimer/monomer mechanism. We reasoned that linker topology and local steric/hydrophobic environments around the pyrene pair could influence non‐radiative relaxation pathways and, consequently, fluorescence efficiency, providing a second design axis alongside excimer–monomer equilibrium.

To test these ideas, we synthesized a six‐member analogue series spanning distinct linker topologies and steric/hydrophobic constraints (Figure 1b, Figures S1 and S2). Specifically, #1 removes bulky dibutyl substituents to increase conformational freedom, #2 introduces a dimethyl‐substituted carbon center to impose moderate restrictions, and #3 uses a cyclopropyl motif to maximize rigidity and enforce separation. In parallel, #4–#6 progressively increase hydrophobic bulk and shape (diethyl, diheptyl, and dibenzyl), with #6 additionally introducing aromatic substituents with potential *π*–*π* interactions. Collectively, these analogues establish a rational gradient of linker‐imposed spatial constraint and show minimal cytotoxicity (Figure S3). We therefore proceeded to evaluate the analogue series by solution‐phase photophysical measurements.

### 2.2 Solution‐Phase Screening Selects Probe #2 for Improved Aqueous Fluorescence Efficiency and Ratiometric Polarity–Viscosity Responsiveness

**FIGURE 2:**
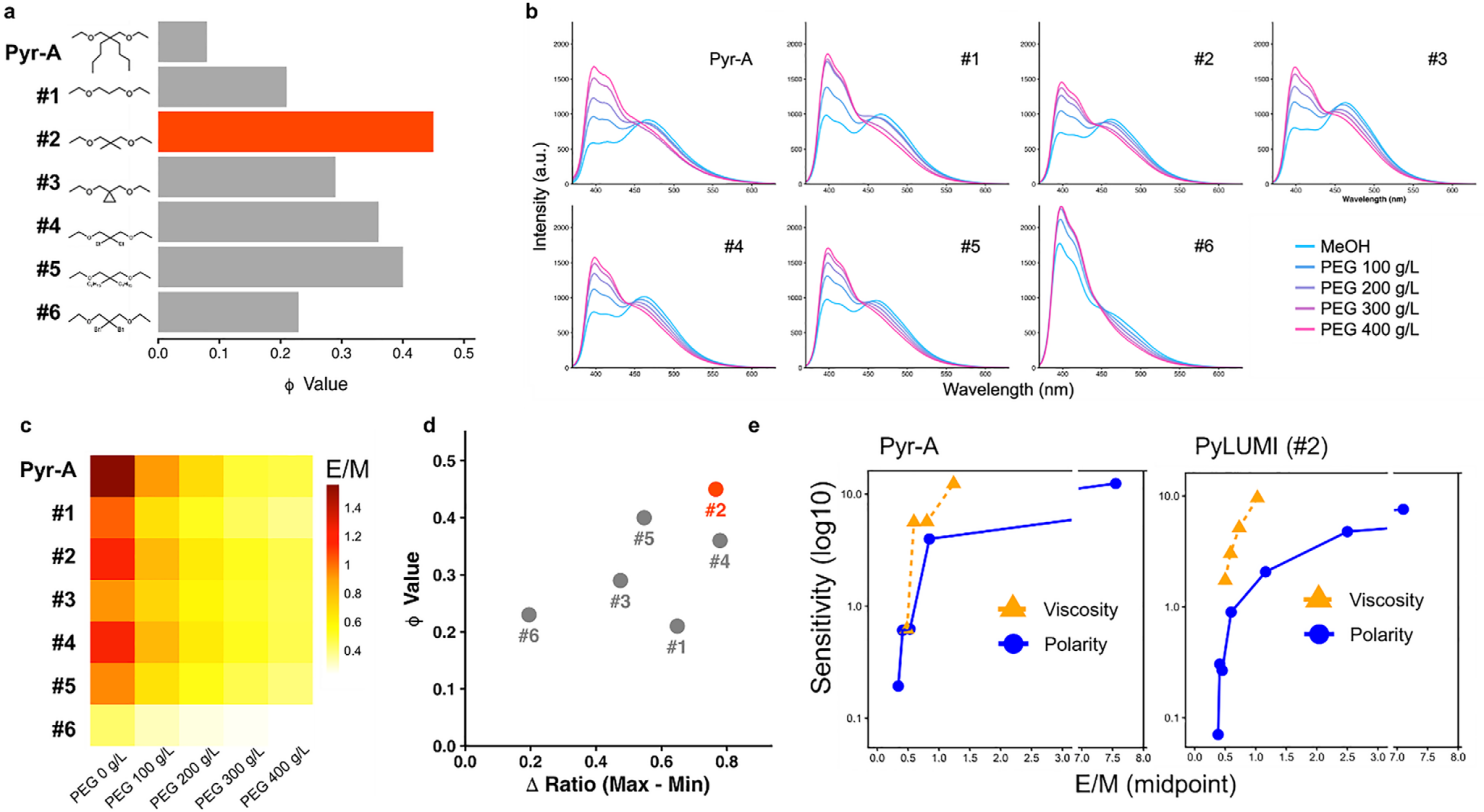
Solution‐phase screening selects probe #2 (PyLUMI) with improved aqueous fluorescence efficiency and ratiometric polarity–viscosity responsiveness. (a) Fluorescence quantum yield (ΦF) of Pyr‐A and analogues in aqueous conditions. (b) Fluorescence emission spectra of Pyr‐A and probe #2 acquired across increasing PEG 600 concentrations (MeOH/PEG 600 mixtures). (c) Heatmap visualization of E/M ratios quantified from (B) across PEG 600 concentrations. (d) Integrated selection plot comparing fluorescence quantum yield (ΦF) and E/M dynamic span (ΔE/M; max–min) for the analogue series, highlighting probe #2. (e) Dimensionless sensitivity analysis comparing polarity‐ and viscosity‐weighted responsiveness of Pyr‐A and probe #2 (PyLUMI). Polarity sensitivity was estimated using dielectric‐constant–tuned solvent mixtures, and viscosity sensitivity was derived from PEG 600–dependent viscosity values based on prior reports (see Methods and Figure S5). This analysis identifies a stronger viscosity‐weighted component for PyLUMI under the tested calibration conditions, while supporting interpretation of E/M as an integrated polarity–viscosity readout. Probe concentration was ∼1.0 µm unless otherwise indicated; excitation wavelength, 358 nm.

To prioritize an optimized reporter from the analogue series, we benchmarked solution‐phase photophysical performance under aqueous conditions. Fluorescence quantum yield (ΦF) measurements showed that probe #2 exhibited the highest fluorescence efficiency in water among the six analogues and exceeded that of the first‐generation probe Pyr‐A (Figure 2a), indicating that linker engineering can improve photon output in the aqueous regime where Pyr‐A is intrinsically limited [8].

We next assessed E/M responsiveness to viscosity modulation using PEG‐based solutions. Fluorescence spectra acquired across increasing PEG concentrations showed systematic changes in excimer and monomer emission for both Pyr‐A and #2 (Figure 2b). Quantification of these spectra as E/M ratios demonstrated that #2 retained a robust ratiometric response while providing higher signal, enabling clearer resolution of E/M shifts across viscosity conditions (Figure 2c). To integrate brightness and ratiometric resolving power into a single selection criterion, we compared (i) aqueous fluorescence efficiency and (ii) the E/M dynamic span across the PEG series. Based on this integrated performance (Figure 2d), #2 was prioritized for further physicochemical analysis.

Finally, because E/M should be interpreted as an integrated physicochemical fingerprint shaped by coupled polarity‐related solvation and microviscosity [9, 10], we compared the relative contributions of polarity versus viscosity using a dimensionless sensitivity metric. Polarity sensitivity was estimated from dielectric‐constant–tuned solvent mixtures (Figure S4), whereas viscosity sensitivity was derived from PEG‐dependent viscosity values from prior literature and/or empirical calibration (see Methods and Figure S5). In the E/M regime relevant to our intracellular measurements, Pyr‐A can display comparable polarity and viscosity sensitivities, whereas #2 showed a stronger viscosity‐weighted component under the tested calibration conditions (Figure 2e). Within this dimensionless comparison, #2 can therefore be described as showing a viscosity‐biased response; however, the E/M ratio remains an integrated polarity–viscosity fingerprint rather than a viscosity‐only readout. Together with its improved photon output and ratiometric normalization [11], these properties support #2 as a bright ratiometric reporter for condensate‐associated microenvironmental profiling. Based on this integrated performance, we designated probe #2 as PyLUMI (**Py** rene‐lineage **L** inker‐modulated **U** ltra‐bright **M** icroenvironment **I** mager) and refer to it hereafter as PyLUMI.

### 2.3 Whole‐Droplet Analysis Decouples E/M Heterogeneity from BRD4‐IDR Enrichment in Recombinant Condensates

**FIGURE 3:**
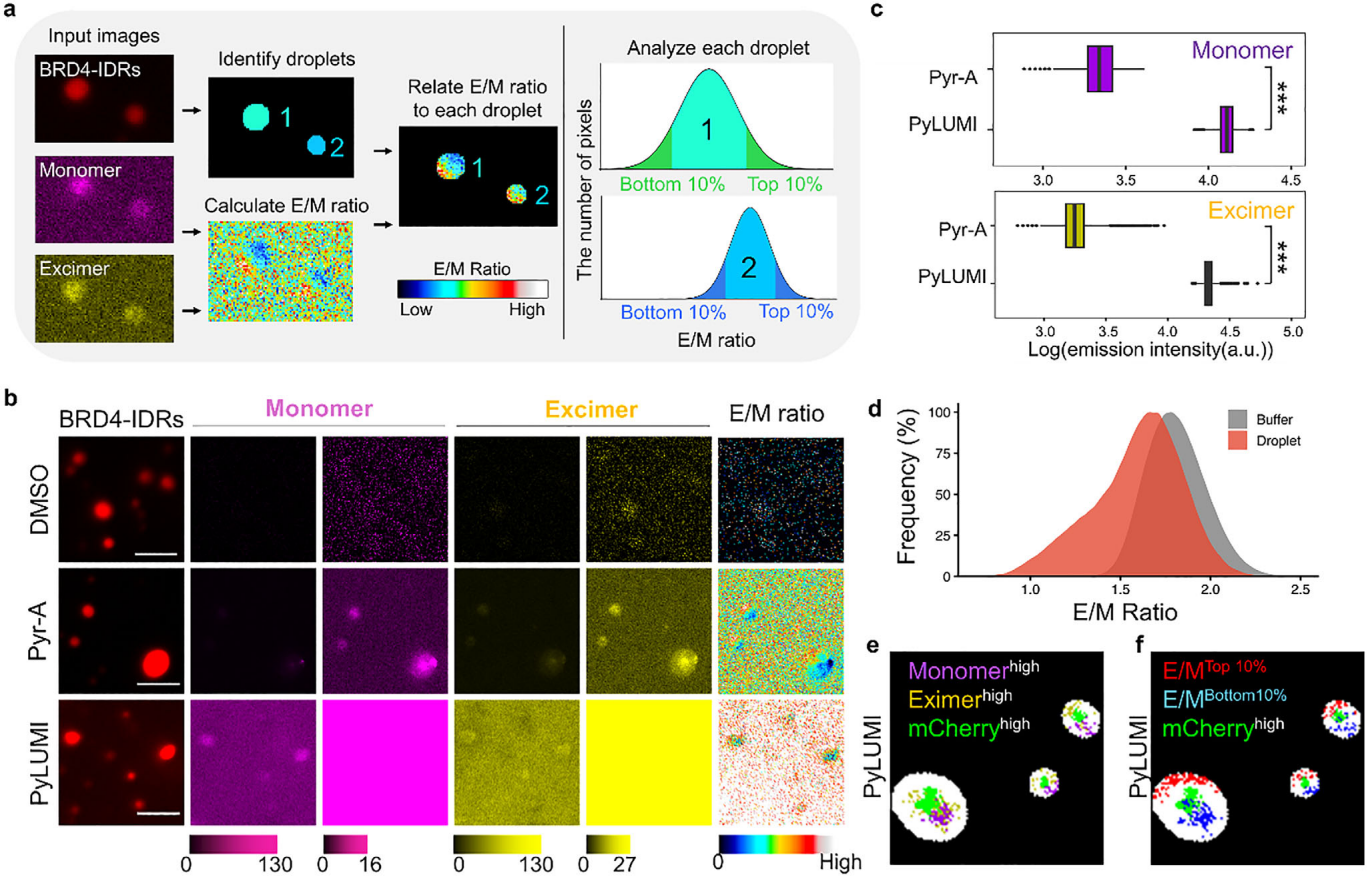
Whole‐droplet ratiometric profiling reveals intra‐condensate E/M heterogeneity that is decoupled from BRD4‐IDR enrichment. (a) Schematic of the whole‐droplet analysis workflow. BRD4‐IDR (mCherry) images were used to identify droplets, monomer and excimer channels were extracted, and pixel‐wise E/M ratio maps were computed and assigned to individual droplets. Per‐droplet E/M distributions were further summarized using decile‐based metrics (Top 10% and Bottom 10%). (b) Representative fluorescence images of recombinant BRD4‐IDR droplets labeled with Pyr‐A or PyLUMI showing monomer and excimer channels and the corresponding pixel‐wise E/M map. (c) Quantification of monomer and excimer emission intensities for Pyr‐A and PyLUMI across droplets (log scale), demonstrating increased photon availability with PyLUMI. (d) Smoothed histograms of E/M ratios comparing pixels inside droplets versus the surrounding dilute medium, highlighting a broadened E/M range within condensates. (e) Representative classification overlays showing that monomer‐high and excimer‐high pixels occupy distinct sub‐droplet territories and do not simply coincide with BRD4‐IDR–enriched regions. (f) Representative classification overlays based on E/M extremes, illustrating the spatial distribution of E/M^TOP10%^ and E/M^Bottom10%^ pixels relative to BRD4‐IDR intensity.

To validate the selected probe under phase‐separated conditions, we compared Pyr‐A and PyLUMI in recombinant BRD4‐IDR condensates and quantified droplet‐scale and intra‐droplet readouts using a whole‐droplet analysis workflow (Figure 3a). Briefly, droplets were identified from the BRD4‐IDR signal, monomer and excimer channels were extracted, and pixel‐wise E/M maps were computed and assigned to each segmented droplet. For each droplet, we further quantified the distribution of E/M values, including summary statistics and the top and bottom deciles to capture intra‐droplet heterogeneity.

Before applying PyLUMI to condensate imaging, we examined whether intermolecular pyrene encounters could contribute detectably to the excimer‐like emission. A mono‐pyrene control probe, Pyr‐M, showed only monomer emission without a detectable broad excimer‐like band at 1 µmm, whereas PyLUMI showed characteristic excimer emission (Figure S6a,b). In addition, PyLUMI fluorescence intensity increased linearly with probe concentration over 0.5–5 µm in aqueous solution (Figure S6c,d). Because intermolecular excimer formation would be expected to introduce nonlinear concentration dependence due to concentration‐dependent intermolecular encounter probability, these results argue against a substantial contribution from intermolecular excimer formation under the tested conditions.

Across condensates, PyLUMI produced higher emission intensities than Pyr‐A in both monomer and excimer channels, improving photon availability for quantitative ratiometric imaging (Figure 3b,c). To test whether the increased signal could reflect probe aggregation in the droplet phase, we compared monomer and excimer intensities inside droplets with those in the surrounding dilute medium. Although both signals tend to increase inside droplets, the shifts were of similar order and not disproportionate enhancement, arguing against strong probe aggregation in the dense phase (Figure S7). We further visualized E/M distributions inside versus outside droplets as histograms to highlight the broadened E/M range within condensates (Figure 3d). Importantly, the spatial distribution of monomer‐ and excimer‐high pixels did not simply coincide with BRD4‐IDR–enriched regions, arguing against dominant artifacts from protein‐driven probe compaction or enrichment bias. Instead, pixel‐wise E/M maps remained well resolved and revealed pronounced intra‐droplet heterogeneity. Visual inspection showed that monomer‐ or excimer‐dominant regions occupy distinct sub‐droplet territories, and decile‐based metrics (Top 10% and Bottom 10%) captured these patterns quantitatively at the droplet level (Figure 3e,f). To further quantify heterogeneity with respect to BRD4‐IDR enrichment, we computed, for each droplet ROI, the Pearson correlation coefficient between pixel‐wise E/M values and mCherry intensity. Across 249 droplets, the sign of the correlation was nearly evenly split, with 51.0% (127 droplets) showing positive correlations (r > 0) and 49.0% (122 droplets) showing negative correlations (r < 0), consistent with bidirectional intra‐droplet heterogeneity rather than a uniform enrichment trend (Figure S8). Moreover, within size‐matched droplet populations, correlations between mCherry intensity and E/M, monomer intensity, or excimer intensity were not consistently positive and instead included both positive and negative relationships, further arguing against dominant protein‐driven probe compaction or concentration bias (Figure S9).

We further examined whether pixel‐wise E/M values were simply determined by total PyLUMI fluorescence intensity. Total intensity was defined as the sum of monomer and excimer intensities within each droplet ROI. Representative pixel‐wise scatter plots and droplet‐wise correlation analysis showed no uniform relationship between total fluorescence intensity and E/M ratio (Figure S10a,b). In addition, intensity‐binned analysis showed broad overlap of E/M distributions across five total‐intensity bins, indicating that pixel‐wise E/M values were not merely brightness‐dependent readouts (Figure S10c).

Together, these results establish a whole‐droplet analysis framework for spatially resolved ratiometric profiling. The mono‐pyrene control, concentration‐dependence analysis, droplet enrichment analysis, BRD4‐IDR correlation analysis, and total‐intensity analysis collectively argue against dominant artifacts from intermolecular excimer formation, probe aggregation, protein‐driven enrichment, or total‐intensity dependence. These findings support the interpretation of E/M patterns as reflecting local microenvironmental heterogeneity within condensates.

### 2.4 PyLUMI Captures Cell‐Cycle–Dependent Centrosome Signatures Beyond Area Changes

**FIGURE 4:**
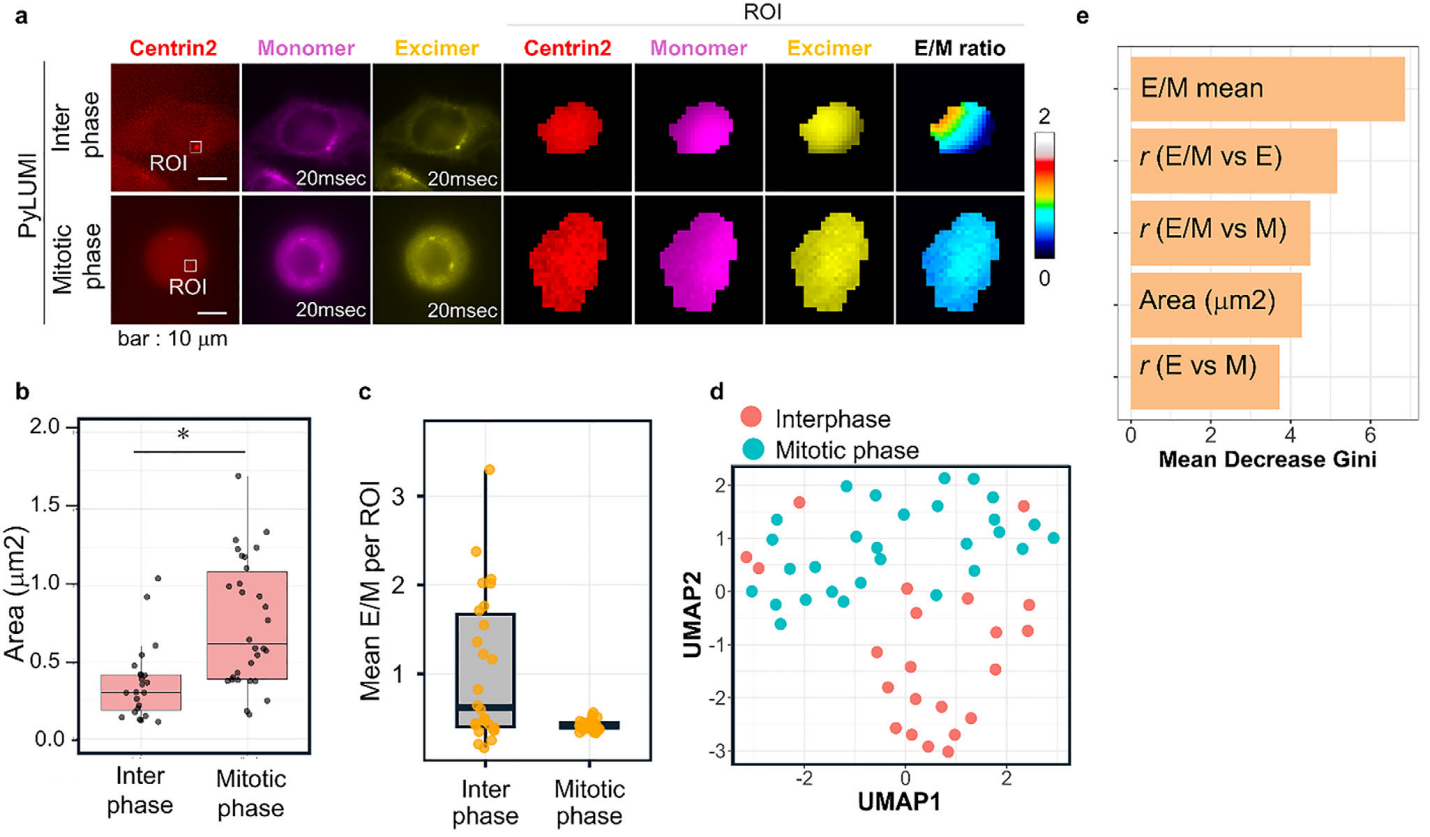
PyLUMI maps centrosome E/M fingerprints and enables cell‐cycle phenotyping beyond size differences. (a) Representative images of Centrin2‐labeled centrosomes and PyLUMI monomer and excimer channels acquired at 20 ms exposure per channel. Pixel‐wise E/M maps were computed within Centrin2‐defined ROIs. (b) ROI area comparison showing that centrosomes are significantly larger in mitosis than interphase. (c) Comparison of mean E/M within centrosome ROIs between interphase and mitotic cells. (d) UMAP embedding of five ROI‐level features (area, mean E/M, r(E/M, M), r(E/M, E), and r(M, E)) separates interphase and mitotic centrosomes in feature space. (e) Feature‐importance analysis (mean decrease Gini) identifies E/M‐derived parameters, particularly mean E/M, as the dominant contributors to classification relative to area and intensity‐correlation features.

We next applied PyLUMI to centrosomes to test whether ratiometric microenvironmental fingerprints can report cell‐cycle–dependent states in cells. Using Centrin2 to define centrosome ROIs (see Figure S11), we acquired monomer and excimer channels (20 ms each) and computed pixel‐wise E/M maps within each ROI (Figure 4a). Consistent with centrosome maturation during mitosis, centrosome size increased during mitosis: ROI area was significantly larger in mitotic than interphase centrosomes (Figure 4b). In parallel, PyLUMI reported a systematic shift in mean E/M between interphase and mitotic centrosomes (Figure 4c), indicating that centrosome‐associated microenvironments are remodeled across the cell cycle.

To assess whether these differences support phenotyping beyond a single metric, we extracted ROI‐level features including area, E/M mean, and three correlation coefficients, r(E/M vs M), r(E/M vs E), and r(M vs E). Unsupervised embedding of these multivariate features (UMAP) separated interphase and mitotic centrosomes (Figure 4d), demonstrating that the combined feature set captures cell‐cycle–dependent signatures. Importantly, feature‐importance analysis showed that E/M‐derived parameters, particularly E/M mean, contributed more strongly to classification than area (Figure 4e). Thus, while centrosome enlargement is a prominent mitotic hallmark, PyLUMI reveals an additional, dominant axis of phenotyping encoded in ratiometric microenvironmental features rather than size alone.

### 2.5 Pathology‐Associated Centrosome Amplification Exhibits a Persistent Elevation of E/M Fingerprints Without Size Changes

**FIGURE 5:**
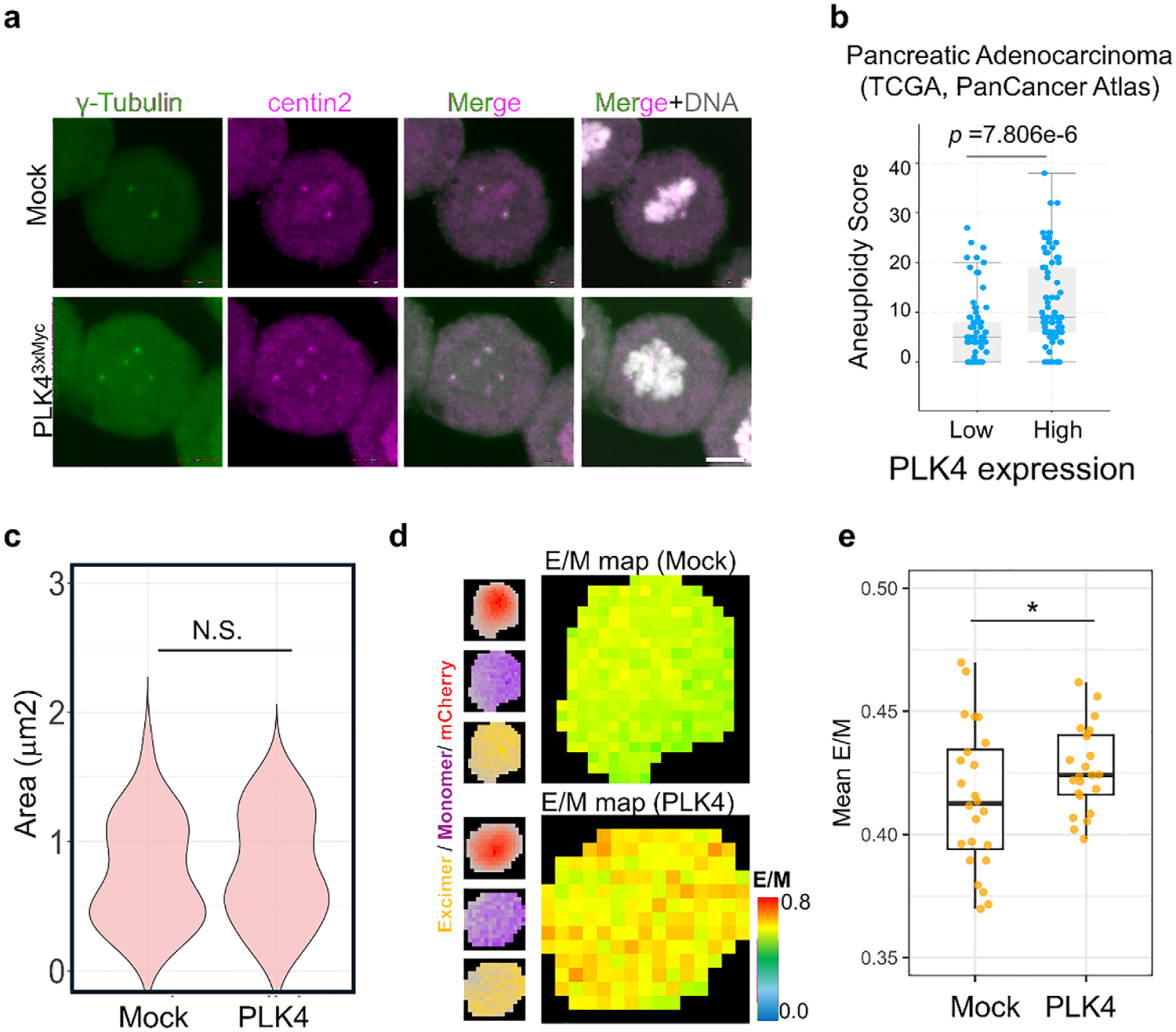
PyLUMI detects pathology‐associated centrosome amplification via persistent elevation of E/M fingerprints without size changes. (a) Representative images of centrosomes in control and PLK4‐overexpression conditions. Centrin2‐defined ROIs were used for subsequent quantification. (b) TCGA‐based analysis showing association between centrosome/aneuploidy‐related signatures and tumor contexts. (c) Centrosome ROI area comparison showing no significant size difference between normal and oncogenic mitotic centrosomes. (d) Representative pixel‐wise E/M maps within Centrin2‐defined centrosome ROIs for normal and oncogenic mitotic centrosomes. (e) Quantification of mean E/M within centrosome ROIs showing a significant elevation in oncogenic centrosomes relative to normal mitotic centrosomes and incomplete convergence to the normal mitotic distribution.

To test whether PyLUMI can detect pathology‐associated centrosome states beyond cell‐cycle maturation, we next examined a centrosome amplification model linked to chromosomal instability. Representative images illustrate centrosomes in control and PLK4‐overexpression conditions and highlight the morphological context used for downstream quantification (Figure 5a and Figure S11). Consistent with clinical relevance, TCGA‐based analyses support an association between centrosome/aneuploidy‐related signatures and tumor contexts (Figure 5b).

We first asked whether amplified centrosomes in mitosis could be distinguished from normal mitotic centrosomes by size. ROI‐based quantification showed no significant difference in centrosome area between normal and aneuploidy‐associated (oncogenic) mitotic centrosomes (Figure 5c), indicating that gross morphology alone is insufficient to capture the altered state. We then performed ratiometric mapping of centrosomes using PyLUMI and computed pixel‐wise E/M landscapes within Centrin2‐defined ROIs (Figure 5d). Unlike size, the PyLUMI fingerprint revealed a clear shift in the microenvironmental readout: mean E/M remained significantly elevated in oncogenic centrosomes compared with normal mitotic centrosomes (Figure 5e). Notably, the distribution did not return to the normal mitotic range, indicating a persistent microenvironmental alteration associated with centrosome amplification. Together, these results show that PyLUMI uncovers pathology‐associated centrosome states that are not explained by size differences.

## 3 Discussion

In this study, we established PyLUMI, an optimized dipyrene‐based ratiometric reporter for spatially resolved profiling of polarity–viscosity fingerprints in phase‐separated condensates. Rational linker engineering improved aqueous fluorescence efficiency while preserving excimer–monomer ratiometry, enabling robust E/M mapping in solution, recombinant protein droplets, and centrosome‐associated compartments in cells. Combined pixel‐level quantification with whole‐droplet and ROI‐based feature extraction further supports ratiometric microenvironmental fingerprints as informative axes for phenotyping centrosome states across the cell cycle and in a pathology‐associated centrosome amplification model.

A key practical advance of PyLUMI is improved photon efficiency relative to the first‐generation probe Pyr‐A, achieved through linker engineering without changing the pyrene chromophore. We speculate that the #2 linker architecture reduces non‐radiative relaxation by constraining intramolecular motions and local solvation around the pyrene pair, consistent with the general principle that environment‐sensitive fluorophores are strongly governed by coupled microviscosity/polarity and conformational dynamics [7, 9, 10]. This improvement lowered the imaging burden in live cells. PyLUMI enabled centrosome E/M mapping at 1 µm with 20 ms exposure per channel, whereas Pyr‐A required 10 µm and 200 ms exposure, and these Pyr‐A conditions were accompanied by a significant reduction (less than half) of interphase mCherry signal (Figure S12). Because photodamage scales with excitation dose and exposure, reducing fluorophore dose and acquisition time is essential for preserving physiology and quantitative accuracy [12, 13, 14, 15].

Our centrosome analyses decouple morphology from microenvironment. Although the centrosome area increased during mitosis, E/M‐derived features outperformed area in classification, indicating information beyond gross size. This is consistent with centrosomes/PCM behaving as selective condensate‐like assemblies that concentrate factors for microtubule organization [16] and with pericentrin condensation contributing to centrosome assembly in human cells [17]. Moreover, recent work demonstrates that phosphorylation‐dependent regulation can tune the dynamics and material properties of the PCM scaffold under load, supporting a framework in which centrosome “state” includes regulated material behavior rather than structure alone [18]. Centrosome integrity is also actively maintained by PCM‐stabilizing mechanisms that preserve spindle pole robustness during disassembly, emphasizing that centrosomes are mechanically constrained organelles whose stability is regulated during division [19]. Using optically induced intracellular flows, Mittasch et al. showed that PCM strength and ductility are actively tuned across mitosis and drop sharply at anaphase onset [20], and Wong et al. proposed that centrioles assemble two scaffolds with distinct biophysical properties, supporting a modular view of centrosome maturation rather than a simple uniform increase in stiffness [21]. Within this framework, the centrosomal E/M shifts detected by PyLUMI should be interpreted as relative, integrated polarity–viscosity fingerprints of centrosome‐associated microenvironments. This concept is important because centrosome state is not fully captured by morphology or size alone; rather, ratiometric physicochemical fingerprints provide an additional readout of regulated material‐state remodeling during centrosome maturation and amplification.

A major motivation for condensate biophysics is disease relevance, as altered phase behavior and condensate material states are implicated across neurodegeneration, cancer, and infection [22]. In our pathology‐associated centrosome amplification model, amplified centrosomes did not differ in area from normal mitotic centrosomes, yet PyLUMI revealed elevated mean E/M that did not fully converge to the normal mitotic distribution. PLK4 overexpression is a well‐established route to centrosome amplification and can promote tissue pathology in vivo [23], and centrosome amplification correlates with adverse clinical features and worse outcomes in breast cancer [24]. Moreover, supernumerary centrosomes can drive aneuploidy and spontaneous tumorigenesis in mammals [25]. These observations support the idea that ratiometric polarity–viscosity fingerprinting can reveal pathology‐associated physicochemical remodeling that is not apparent from morphology alone. At the same time, extension of this framework to additional condensate classes will require condensate‐specific validation and, in some cases, further optimization of probe targeting or retention.

Overall, these findings highlight the value of integrated polarity–viscosity fingerprints as functional readouts of condensate‐associated microenvironments beyond morphology alone. Further optimization of targeting and multiplexing strategies may extend this approach to broader condensate classes and pathology‐relevant microenvironmental mapping.

## 4 Conclusions

PyLUMI is a bright dipyrene ratiometric probe enabled by linker engineering to improve fluorescence output under aqueous conditions while preserving excimer‐to‐monomer readout. The probe supports pixel‐resolved imaging of integrated physicochemical fingerprints in condensate‐associated compartments and reveals biologically relevant state differences in living cells beyond morphology alone. These results establish PyLUMI as a functional platform for spatially resolved profiling of condensate microenvironments.

## 5 Experimental Section

### 5.1 Synthesis of Pyr‐A Analogs

Pyr‐A derivatives were synthesized by reaction of dipyrene derivative 5 with a terminal hydroxy group and polyethylene glycol methyl ether tosylate (average Mn = 900 g mol^−1^) (see Supporting Information) according to a method known in the literature [8]. The chemical structures were determined by 1H‐NMR, 13C‐NMR, IR, and HRMS (See Figures S1 and S2).

### 5.2 Spectrophotometric and Spectrofluorometric Measurements

UV–vis absorption spectra were recorded on a spectrophotometer (JASCO V‐750), and fluorescence emission spectra were acquired on a spectrofluorometer (JASCO FP‐8300). Measurements were performed at 25 ± 1°C. Unless otherwise noted, probe concentrations were ∼1.0 µm. Fluorescence quantum yield (ΦF) was determined using quinine sulfate as a reference standard (Φstd = 0.55). Solvent polarity of mixtures was expressed using the ET(30) parameter, which was determined from the absorption maximum of Betaine 30.

### 5.3 Dimensionless Sensitivity Evaluation

To compare viscosity‐ and polarity‐weighted responsiveness of the E/M readout, we quantified a dimensionless sensitivity metric from calibration series using adjacent measurement points. For each probe, E/M ratios were measured across (i) a viscosity series (methanol/PEG 600 mixtures) and (ii) a polarity series (dielectric‐constant–tuned solvent mixtures). Data points were ordered by the environmental parameter (viscosity η or dielectric constant DC), and sensitivities were computed between each pair of neighboring points.

For viscosity sensitivity, we defined the midpoint‐normalized fractional changes as

$$\Delta_{rel}\left(E/M\right)=\frac{{\left(E/M\right)}_{i+1}-{\left(E/M\right)}_{i}}{{\left(E/M\right)}_{mid}},\;\;{\left(E/M\right)}_{mid}=\frac{{\left(E/M\right)}_{i}+{\left(E/M\right)}_{i+1}}{2}$$

$$\Delta_{rel}\left(\eta \right)=\frac{\eta_{i+1}-\eta_{i}}{\eta_{mid}},\;\;\eta_{mid}=\frac{\eta_{i}+\eta_{i+1}}{2}$$

The dimensionless viscosity sensitivity was then calculated as the magnitude of the ratio of these fractional changes:

$$S_{\eta }=\left|\frac{\Delta_{rel}\left(E/M\right)}{\Delta_{rel}\left(\eta \right)}\right|$$

Polarity sensitivity was computed analogously by replacing η with the dielectric constant DC:

$$S_{DC}=\left|\frac{\Delta_{rel}\left(E/M\right)}{\Delta_{rel}\left(DC\right)}\right|$$

with *DC_mid_* = (*DC_i_* + *DC*_i+1_)/2.

For each probe, we report *Sη* and *S_DC_* as functions of the midpoint value *(E/M)_mid_* and *η_mid_* or *DC_mid_*, respectively. Outlier values from polarity calibration were excluded prior to calculation (E/M > 15). Viscosities of the solvent mixtures were calculated from the molar fraction of PEG 600, based on the relationship reported previously [26].

### 5.4 Cell Culture and Cell Viability Assay

HeLa cells were obtained from ATCC (ATCC Cat# CCL‐2, RRID:CVCL_0030) and cultured in Dulbecco's Modified Eagle Medium (Invitrogen) with 10% fetal bovine serum and penicillin/streptomycin in a humidified atmosphere with 5% CO_2_ at 37°C. We did not authenticate this cell line in our laboratory. The effects of the probes on cell viability were evaluated using an MTT assay following previously described protocols [27].

### 5.5 Transfections, Viral Particle Production, and Infection

DNA transfection was performed using Lipofectamine 2000. Lentiviral particles were produced with the MISSION Lentiviral Packaging System (Sigma–Aldrich). HeLa cells were transduced with the lentiviral particles in the presence of 8 µg/mL Polybrene (Sigma–Aldrich) for 48 h following previously described [28].

### 5.6 Molecular Cloning

Recombinant BRD4 IDR‐mCherry was expressed using the pET28a‐BRD4IDR‐mCherry vector, as described in our previous work [30], and purified using Ni‐NTA affinity chromatography. The pET28a‐BRD4IDR‐mCherry was constructed as recently reported (25). In brief, IDRs of BRD4 (674‐1351aa) was picked out from the pcDNA5‐Flag‐BRD4‐WT vector (addgene # 90331, RRID:Addgene_90331) and ligated into the pmCherry‐N1 vector using BamHI (TOYOBO, BAH‐111) and XhoI (New England BioLabs, R0146) with following primers: F1: 5’‐ CTCGAGGCCACCATGTGTTTGCGGAAGAAAAGG ‐3’; R1: 5’‐GGATCCCGACTCTGGAAATTCATG‐3’. Further, BRD4‐IDRs‐mCherry was amplified with F2: 5’‐GAATTCATGTGTTTGCGGAAGAAAAGGAAACC‐3’; R2: 5’‐ GCGGCCGCCTACTTGTACAGCTCGTC ‐3’, and ligated into the pET28a vector (Novagen, Cat# 69864‐3) using EcoRI (New England BioLabs, R0101) and NotI (Nippon gene 316–01451). For the construction of overexpressing Centrin2‐mCherry vector, the pmCherry‐C1 vector was amplified using the primer set (Forward: CTCTACTAGAGGATCCCACCATGGTGAGCAAGGGC, Reverse: GCCCTCTAGACTCGAGCTTGTACAGCTCGTCC), and fused into the pLEX vector digested with BamHI and XhoI using the In‐fusion method. The backbone was subsequently obtained from the pLEX vector using XhoI and AgeI (Nippon gene, 313–02561). The Centrin2 region was amplified from a custom‐synthesized Centrin2 plasmid (GENEWIZ) using the primer set (F3: GCTGTACAAGCTCGAGATGGCCTCCAACTTTAAG, R3: GAGAGGGGCGACCGGTTTAATAGAGGCTGGTC), and inserted into the backbone using the In‐fusion method.

### 5.7 Protein Purification

Recombinant BRD4 IDR‐mCherry was expressed using the pET28a‐BRD4IDR‐mCherry vector, as described in our previous work [29, 30], and purified using Ni‐NTA affinity chromatography.

### 5.8 In Vitro Droplet Assay

Recombinant BRD4‐IDR‐mCherry protein was diluted into a final concentration of 10 µm in droplet buffer (50 mm Tris–HCl pH 7.5, 10% glycerol, 1 mm DTT, 10% PEG), with or without 10 µm of probe, then immediately loaded on a glass‐bottom dish (MATSUNAMI) and covered with a coverslip.

Samples were allowed to incubate for 30 min at Room temperature and subsequently imaged using a Zeiss LSM5 EXCITER microscope with a mercury lamp and a Plan‐Apochromat 100×/1.4 oil objective. Monomer emission was examined using a customized fluorescent cube I (Ex365/DM390/Em425, Zeiss), while excimer emission was monitored using fluorescent cube II (Ex365/DM400/Em490, Zeiss). Axio Vision software (version 4.8, Zeiss: AxioVision Imaging System (RRID:SCR_002677)) was used for image acquisition.

### 5.9 Immunocytochemistry and Confocal Microscopy

Anti‐mCherry (ab167453, RRID:AB_2571870) Rabbit polyclonal antibody and anti‐γ‐Tubulin mouse monoclonal antibody (ab11316, RRID:AB_297920) were acquired from Abcam. Secondary antibodies were acquired from Cell Signaling Technology (Mouse: Cat# 7076, RRID:AB_330924; Rabbit: Cat# 7074, RRID:AB_2099233). For immunofluorescence, synchronized HeLa cells expressing centrin2‐mCherry were washed in phosphate‐buffered saline (PBS, used at a 1× dilution) and fixed for 10 min in cold Methanol at room temperature. Cells were then permeabilized with 0.3% Triton X‐100 in PBS (1×) for 10 min at room temperature. Coverslips were blocked with 4% bovine serum albumin in PBS (1×) for 1 h and incubated with primary antibodies overnight at 4°C and then secondary antibodies for 1 h at room temperature. Coverslips were mounted onto glass slides using Pro‐Long Gold Antifade reagent with DAPI (Life Technologies) and examined using a confocal microscope (FluoView FV10i, Olympus, objective ×60/1.2). All confocal images were analyzed using FV10i software (version 4.1).

### 5.10 Live‐Cell Fluorescence Microscopic Imaging

Cells were seeded onto a glass‐bottom dish (MATSUNAMI) and incubated for 24 h to allow attachment. Then, cells were synchronized using thymidine and nocodazole block to enrich a mitotic population [31] and incubated in medium containing Pyr‐A (10 µm) or PyLUMI (1 µm). After 4 h treatment, cells were rinsed twice with PBS (1×), and fresh culture medium was added. Cells were then examined using a Zeiss LSM5 EXCITER microscope with a mercury lamp and a Plan‐Apochromat 100×/1.4 oil objective. Axio Vision software (version 4.8) was used for acquisition.

### 5.11 Image‐Based Feature Extraction and Aggregation

To quantify centrosomes at image‐level properties relevant to fluorescent probe performance, pixel‐level data were aggregated for each image based on predefined regions of centrosomes marked by centrin2‐mCherry (ROIs). The following features were computed: average E/M ratio, ROI area (size), and Pearson correlation coefficient between “Excimer and Monomer signals (pearson r)”, “E/M and Monomer signals (pearson r)”, and “E/M and Excimer signals (pearson r)”. All features were calculated using dplyr (RRID:SCR_016708) in R. These features were used for UMAP input.

### 5.12 Dimensionality Reduction with UMAP

To visualize sample distribution in a low‐dimensional space, we applied Uniform Manifold Approximation and Projection (UMAP) on scaled image (ROI) ‐level features using the UMAP package [32]. UMAP results were visualized using ggplot2 (RRID:SCR_014601), and plotted points were colored by cell cycle status by PyLUMI.

### 5.13 Random Forest‐Based Feature Importance Analysis

Random forest classifiers were trained separately for PyLUMI to evaluate the relative importance of image‐level features in classifying cell cycle phase. Classification was performed using the randomForest package with 500 trees per model [33]. Feature importance was assessed based on the Mean Decrease in Gini index, and visualizations were generated using ggplot2.

### 5.14 Statistical Analysis

Statistical analyses were performed using R software (R Project for Statistical Computing (RRID:SCR_001905)). Statistically significant differences in mean values between respective groups were tested by Student's t‐test or Mann–Whitney test. *p* values < 0.05 were considered to indicate a statistically significant difference.

## Author Contributions

**Shogo Amemori**: investigation, methodology, validation, writing – review and editing. **Koki Matsumoto**: conceptualization, methodology, investigation, validation, formal analysis, visualization, writing – review and editing, writing – original draft. **Kei Makiyama**: investigation, validation. **Kazuma Ogawa**: investigation, methodology. **Kentaro Ohira**: investigation, validation. **Shih‐Cheng Chen**: investigation, methodology. **Sora Kitai**: conceptualization, methodology, investigation, visualization, writing – original draft, writing – review and editing, resources. **Dini Kurnia Ikliptikawati**: investigation, validation. **Yoshio Nishiyama**: methodology, investigation, validation, formal analysis, writing – review and editing. **Wei‐Min Liu**: investigation, methodology. **Yuta Kozuka**: investigation. **Chien‐Hung Yu**: investigation, methodology. **Richard W. Wong**: funding acquisition, supervision, visualization, project administration, writing – review and editing. **Koichi Ogami**: methodology, investigation. **De‐Chen Lin**: visualization, writing – review and editing. **Takahiro Soeta**: conceptualization, supervision, funding acquisition, visualization, project administration, writing – original draft, writing – review and editing. **Maho Tobita**: investigation. **Kenji Takahashi**: methodology, investigation, project administration. **Masaharu Hazawa**: conceptualization, supervision, funding acquisition, visualization, project administration, writing – original draft, writing – review and editing. **Motohiro Mizuno**: methodology, investigation. **Hiroshi I Suzuki**: funding acquisition, resources, writing – review & editing.

## Conflicts of Interest

The author declares no conflict of interest.

## Supporting information




**Supporting File 1**: advs76419‐sup‐0001‐SuppMat.docx.

**Supporting File 2**: advs76419‐sup‐0002‐FigureS2_NMR.pdf.

## Data Availability

The data that support the findings of this study are available from the corresponding author upon reasonable request.
