# Supplementary material for: A Bright Ratiometric Dipyrene Probe for Functional Imaging of Condensate Microenvironments in Living Cells
Source: Adv Sci (Weinh). 2026 Jul 16;13(54):e76419. doi: 10.1002/advs.76419 (PMC13374536; doi:10.1002/advs.76419)
Supplement: Supplementary file 1 [file ADVS-13-e76419-s002.pdf]

KS-4-45-fr
1H

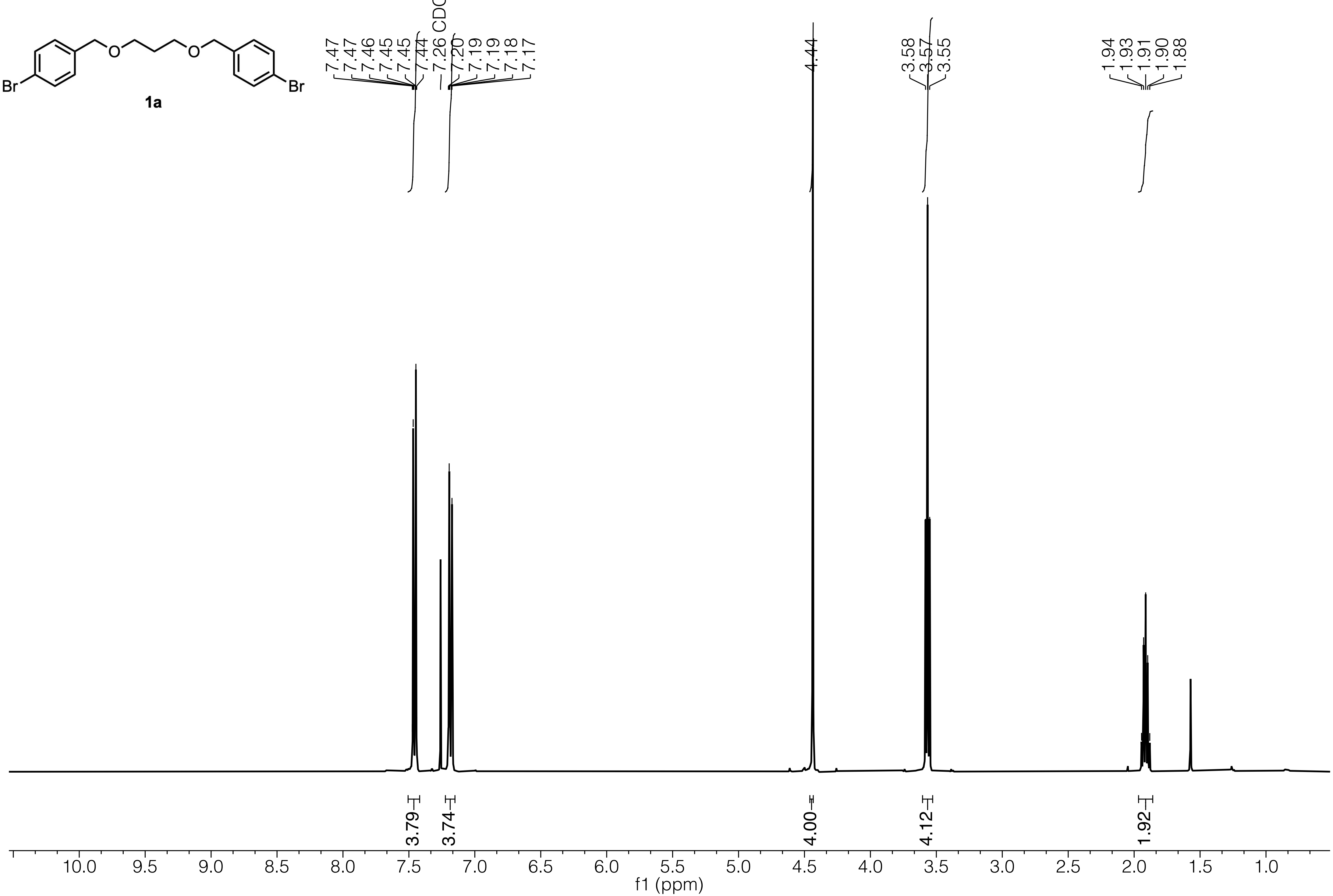

Br
O
O
Br
1a
7.47
7.47
7.46
7.45
7.45
7.44
7.26 CDCl3
7.20
7.19
7.19
7.18
7.17
4.44
3.58
3.57
3.55
1.94
1.93
1.91
1.90
1.88
3.79
3.74
4.00
4.12
1.92
10.0
9.5
9.0
8.5
8.0
7.5
7.0
6.5
6.0
5.5
5.0
4.5
4.0
3.5
3.0
2.5
2.0
1.5
1.0
f1 (ppm)

KS-4-45-fr
13C

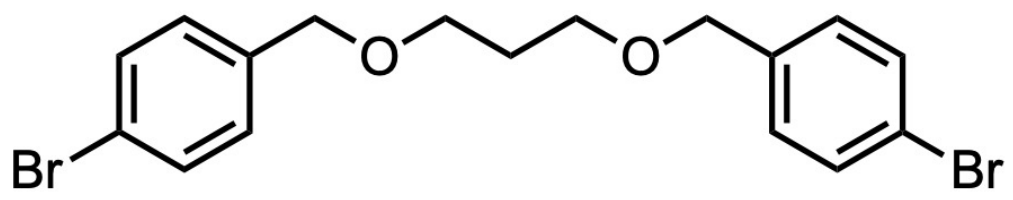

1a

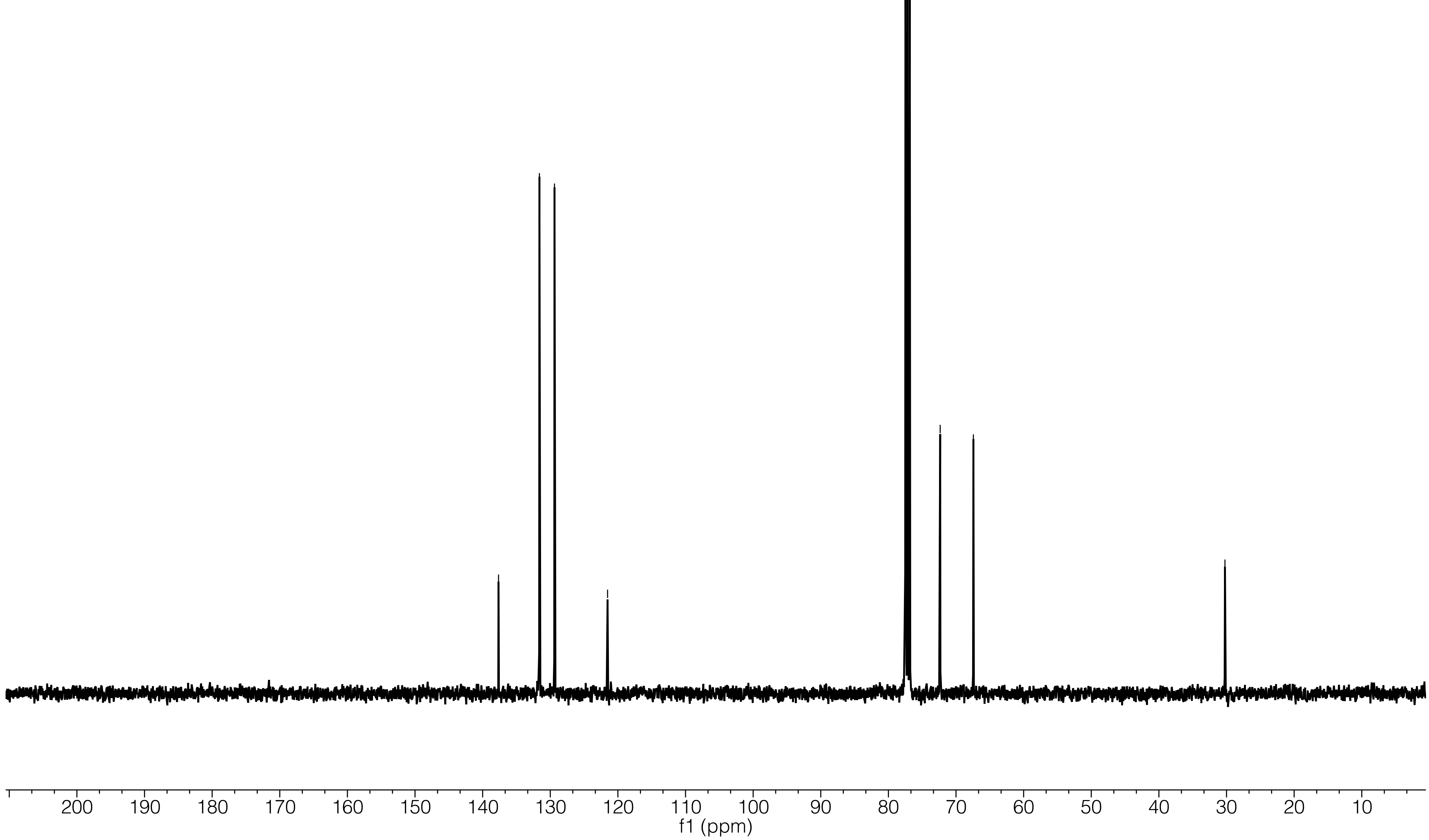

KS-4-44-fr
1H

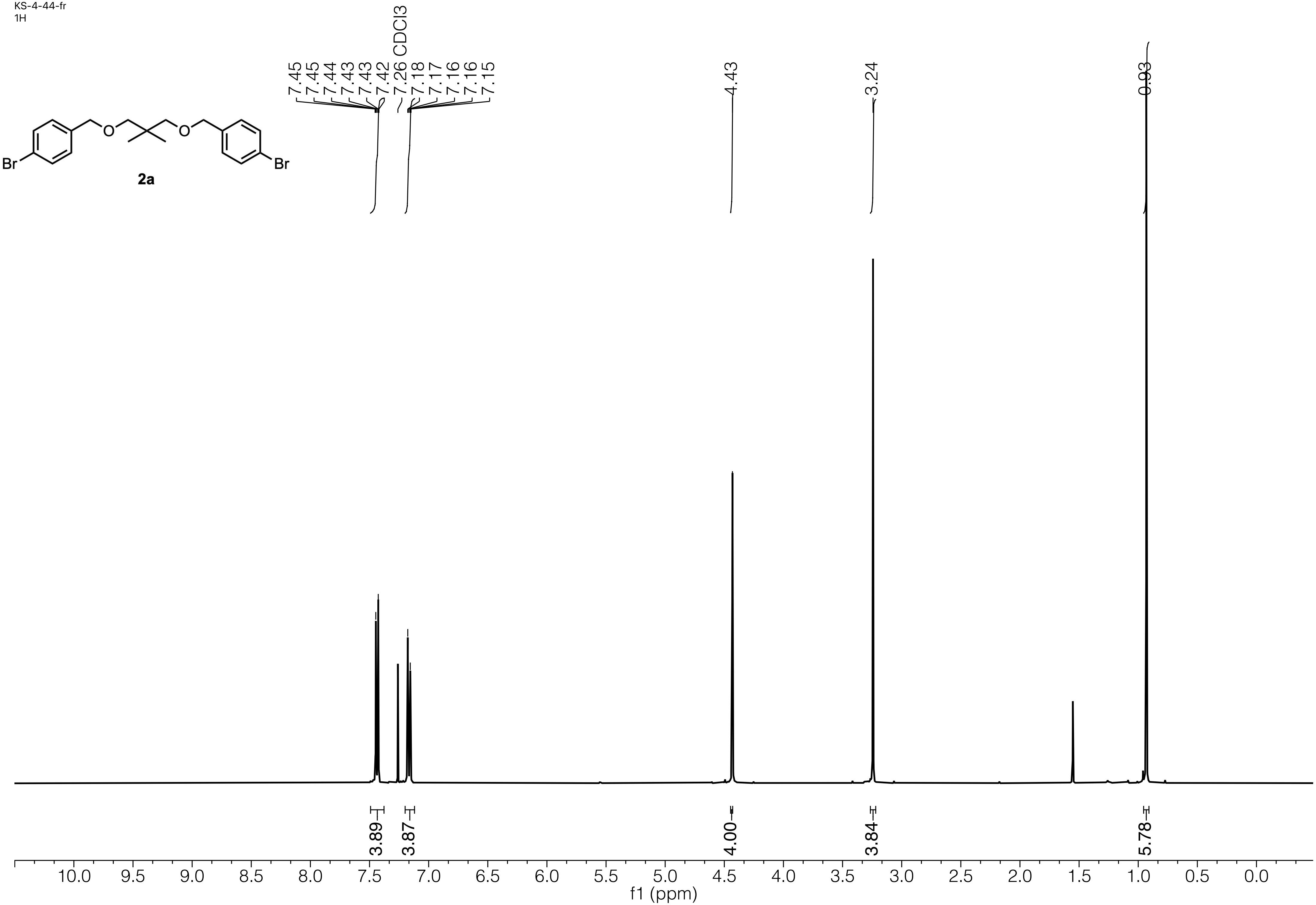
7.45
7.45
7.44
7.43
7.43
7.42
7.26 CDCl3
7.18
7.17
7.16
7.16
7.15
4.43
3.24
0.93
Br
O
O
Br
2a
3.89
3.87
4.00
3.84
5.78
10.0
9.5
9.0
8.5
8.0
7.5
7.0
6.5
6.0
5.5
5.0
4.5
4.0
3.5
3.0
2.5
2.0
1.5
1.0
0.5
0.0
f1 (ppm)

KS-4-44-fr
13C

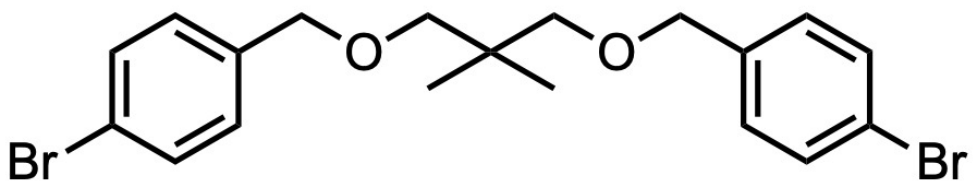

2a

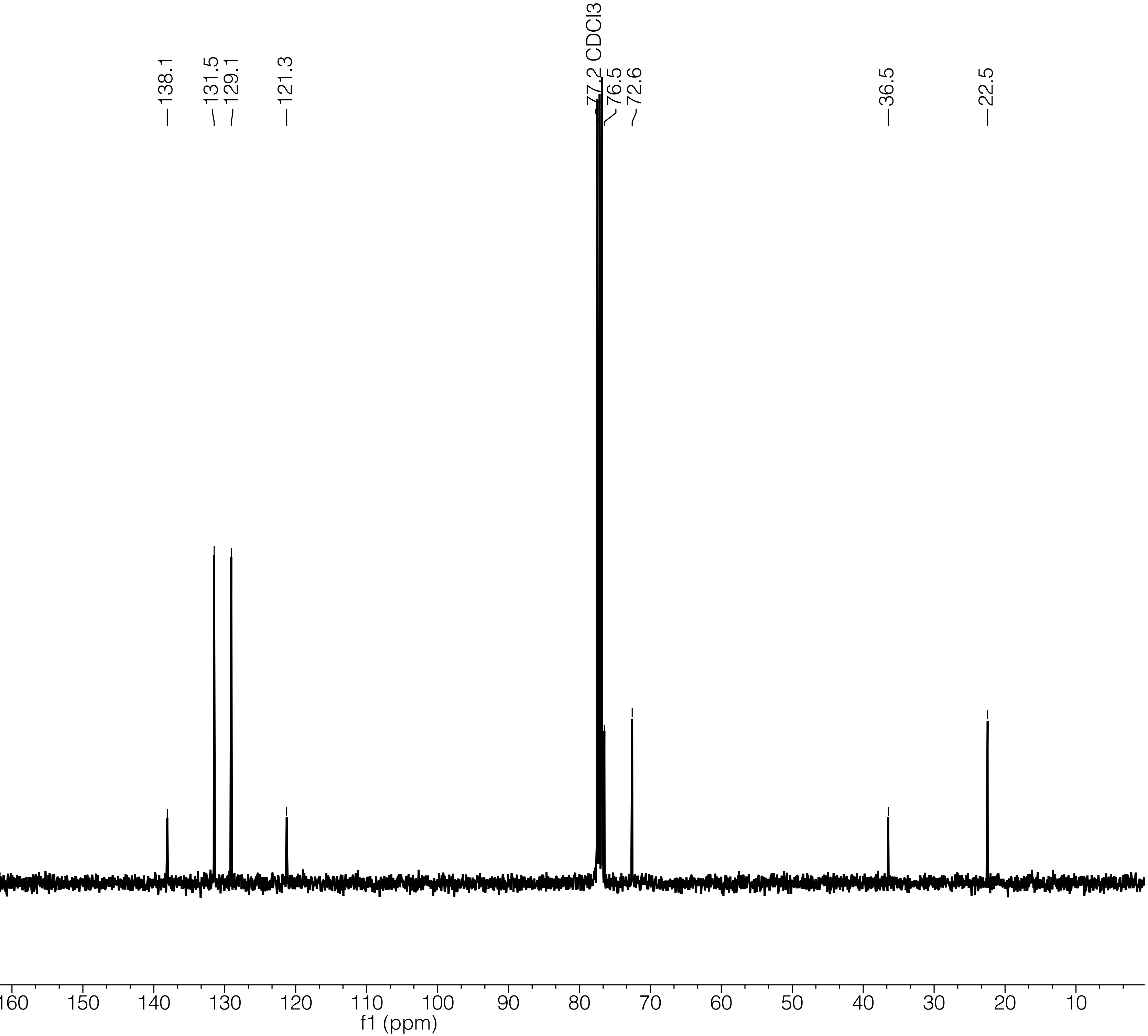

KS-4-42-fr
1H

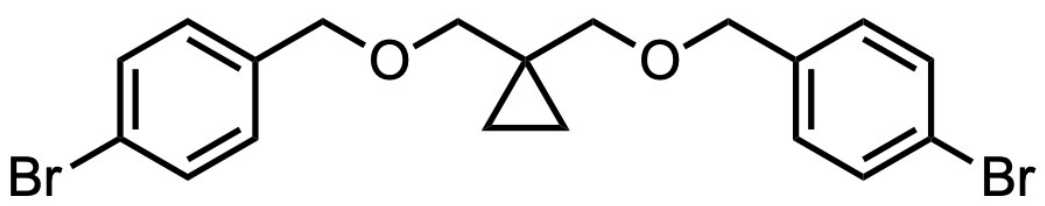

3a

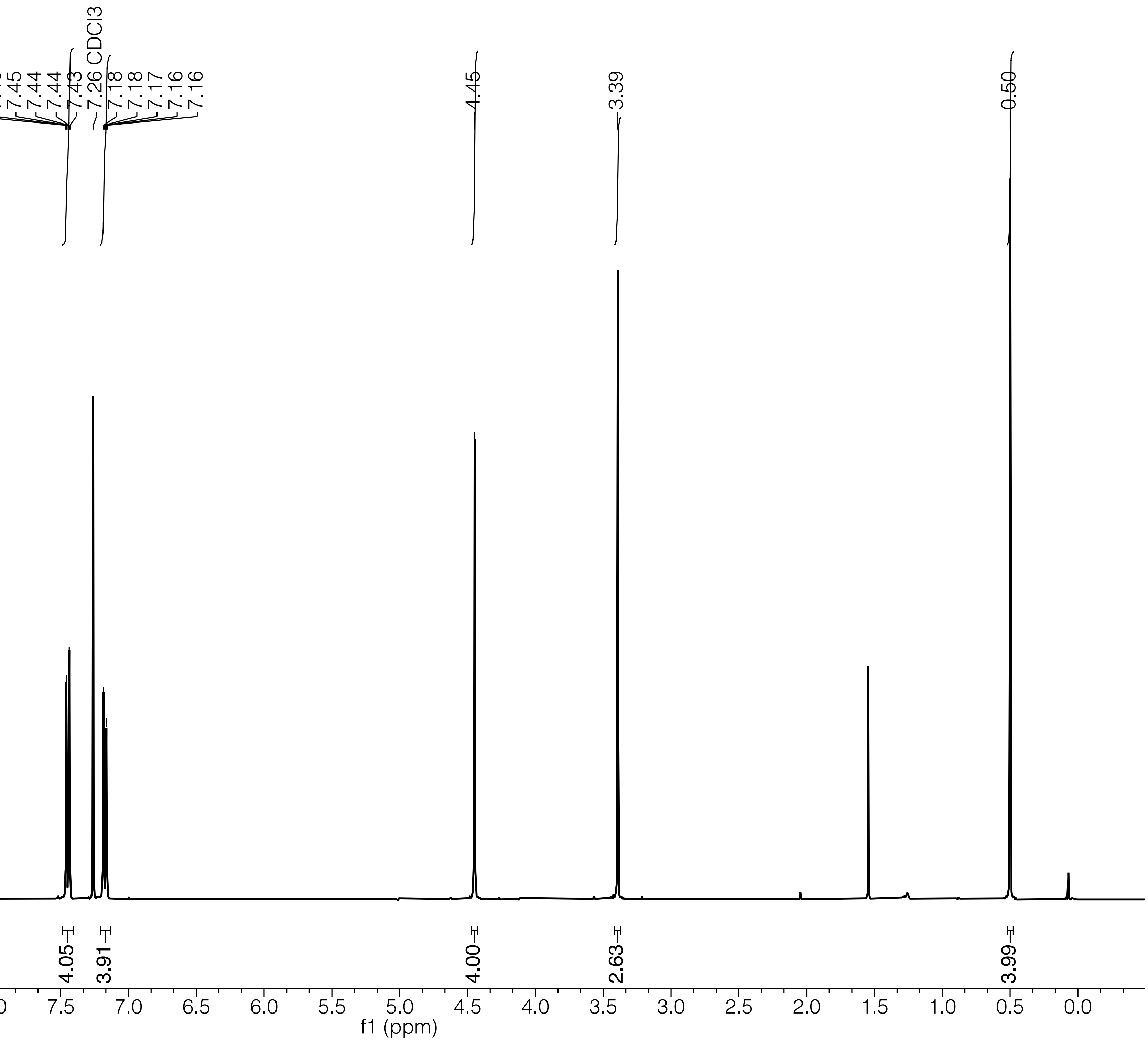

KS-4-42-fr
13C

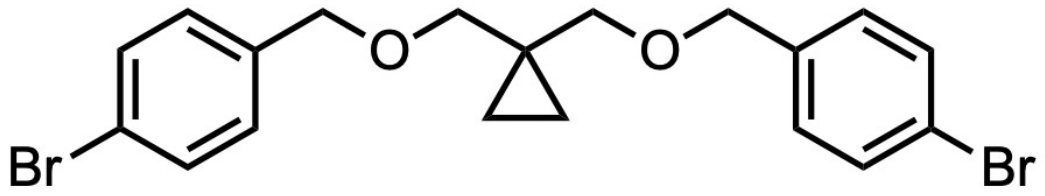

3a

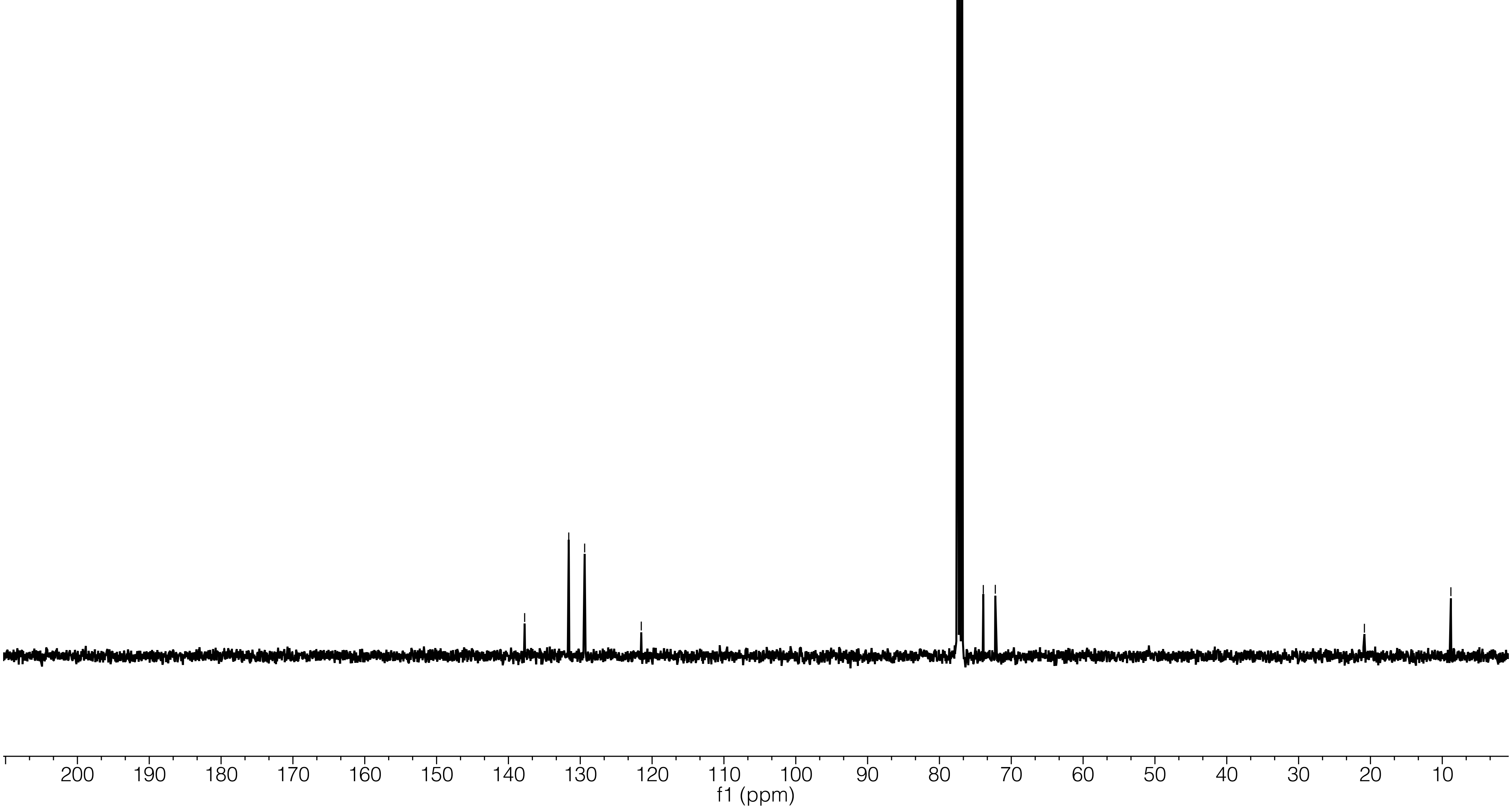

KS-4-96-fr1
1H

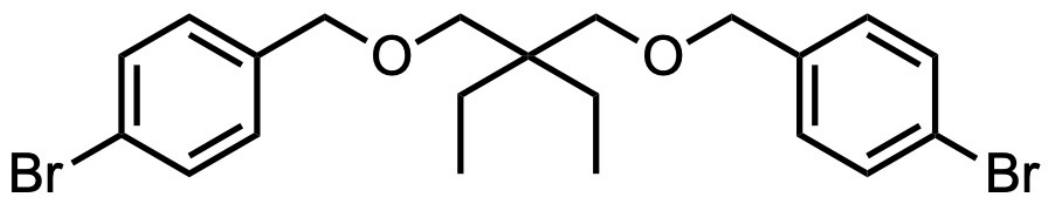

4a

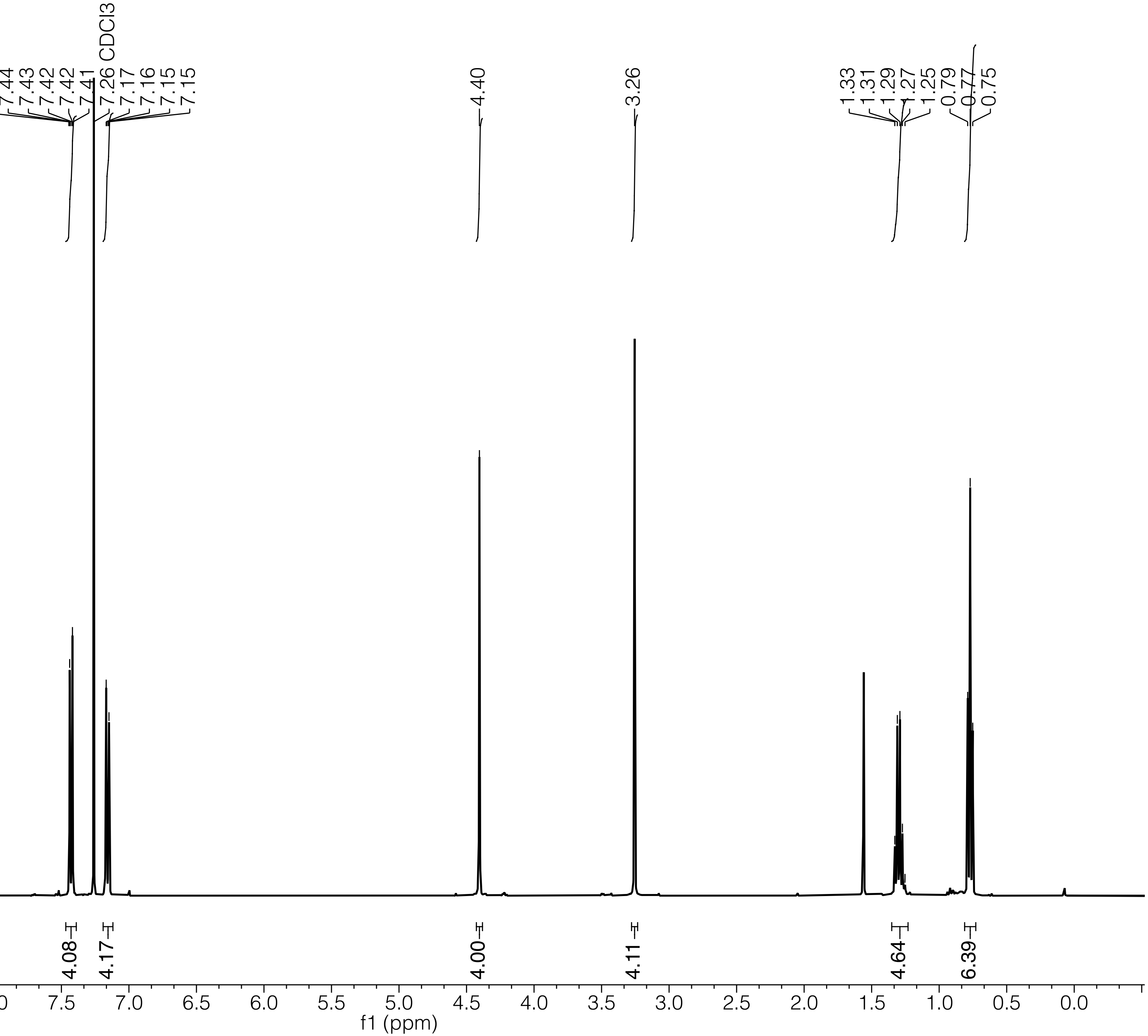

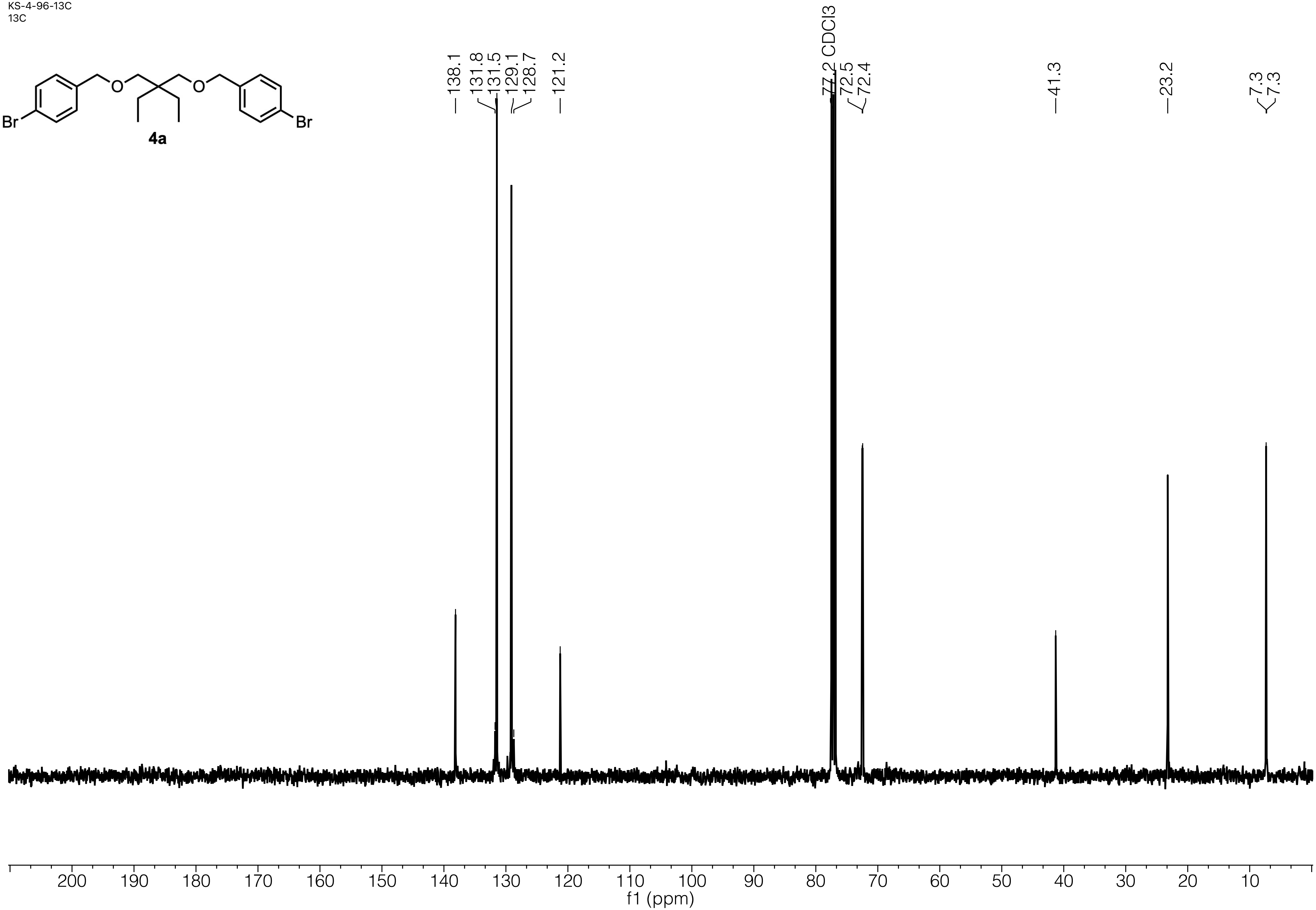
KS-4-96-13C
13C
Br
Br
O
O
4a
138.1
131.8
131.5
129.1
128.7
121.2
77.2 CDCl3
72.5
72.4
41.3
23.2
7.3
7.3
200
190
180
170
160
150
140
130
120
110
100
90
80
70
60
50
40
30
20
10
f1 (ppm)

KS-5-7-fr4
1H

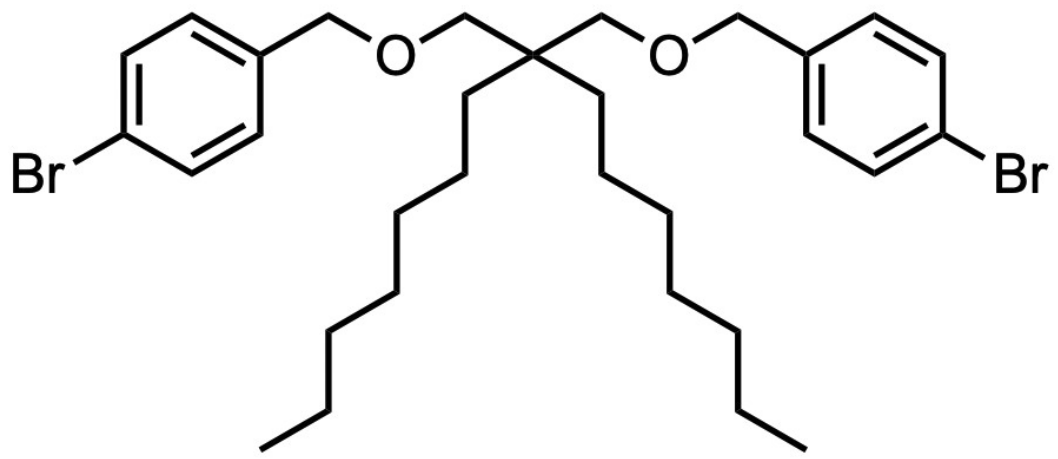

5a

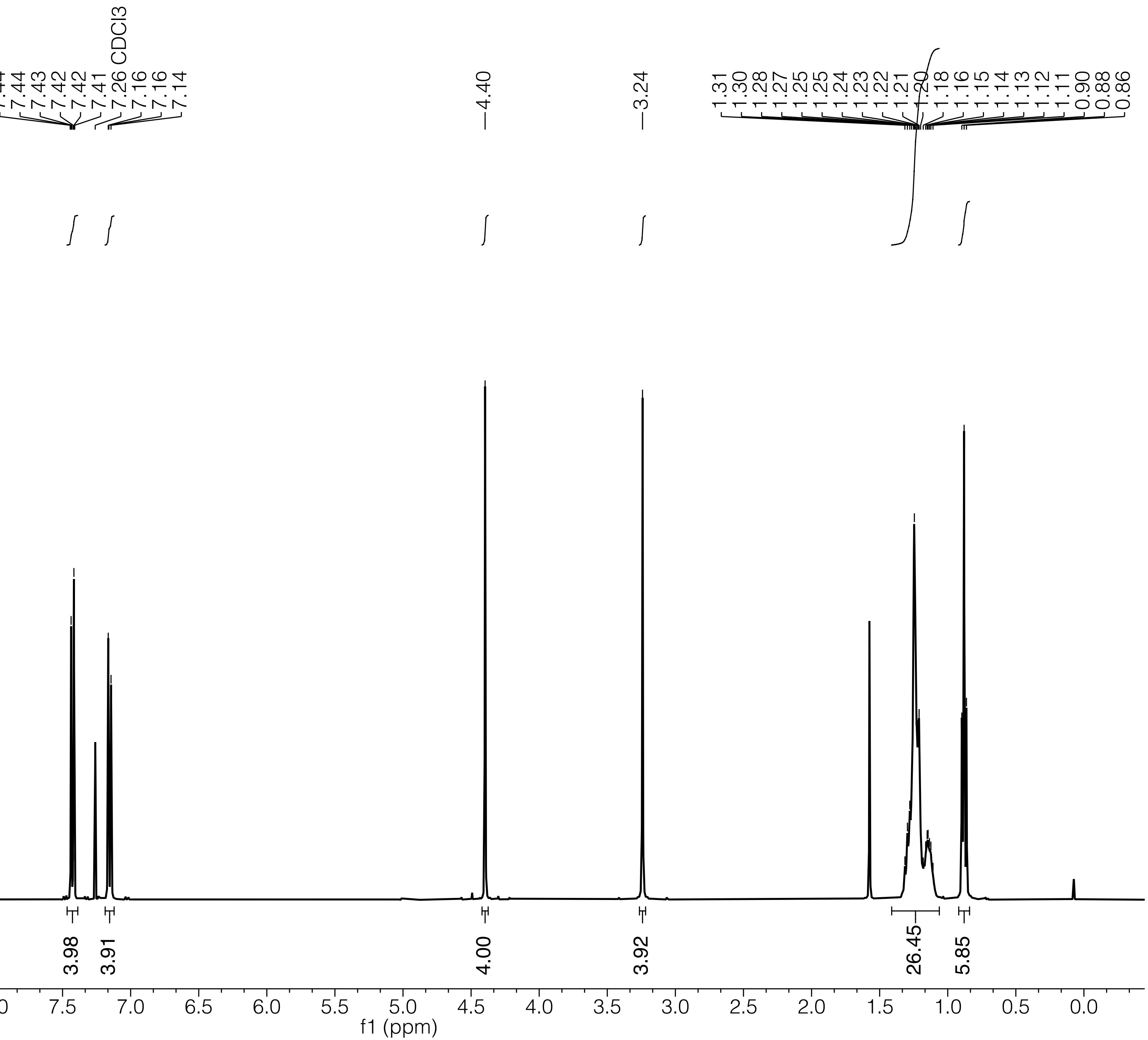

KS-5-7-fr4
13C

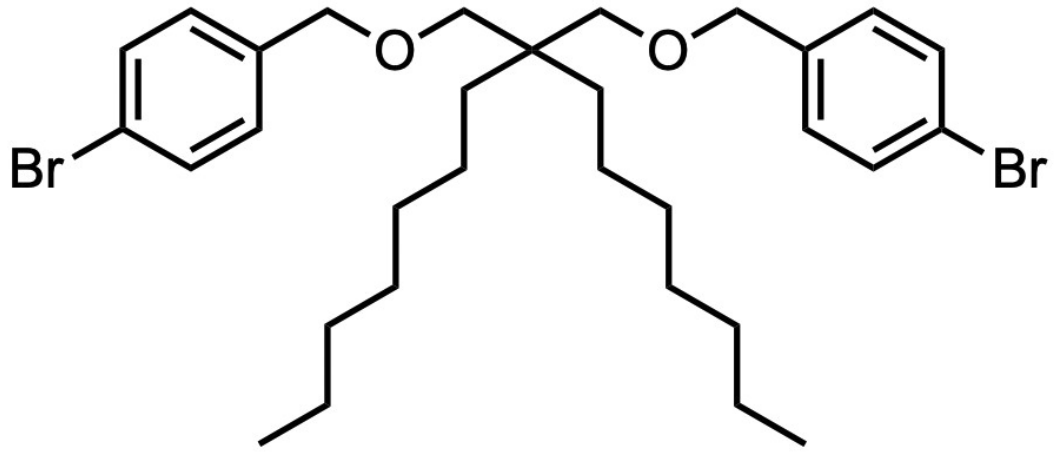

5a

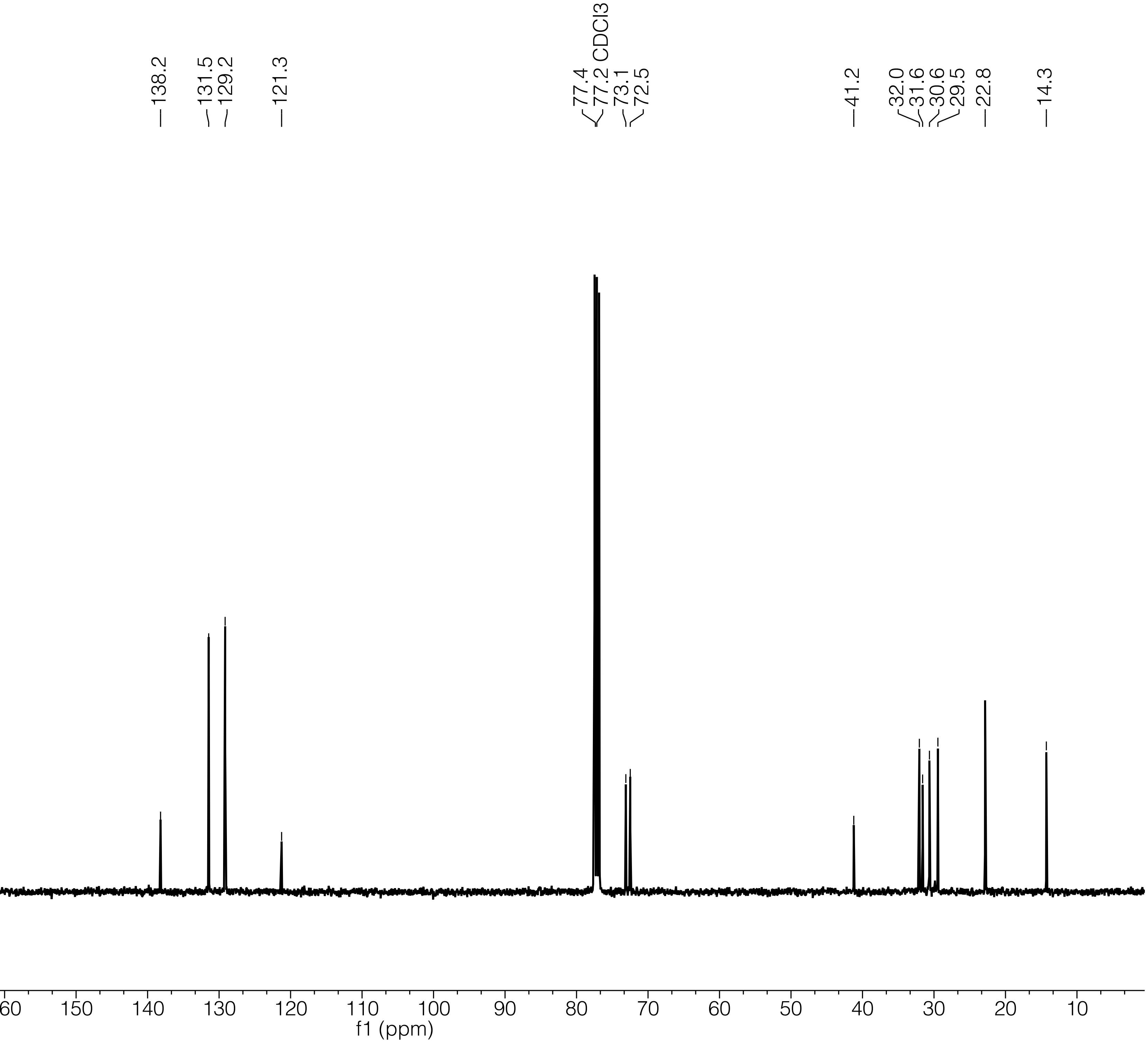

KS-5-8-Acetone-d6
1H

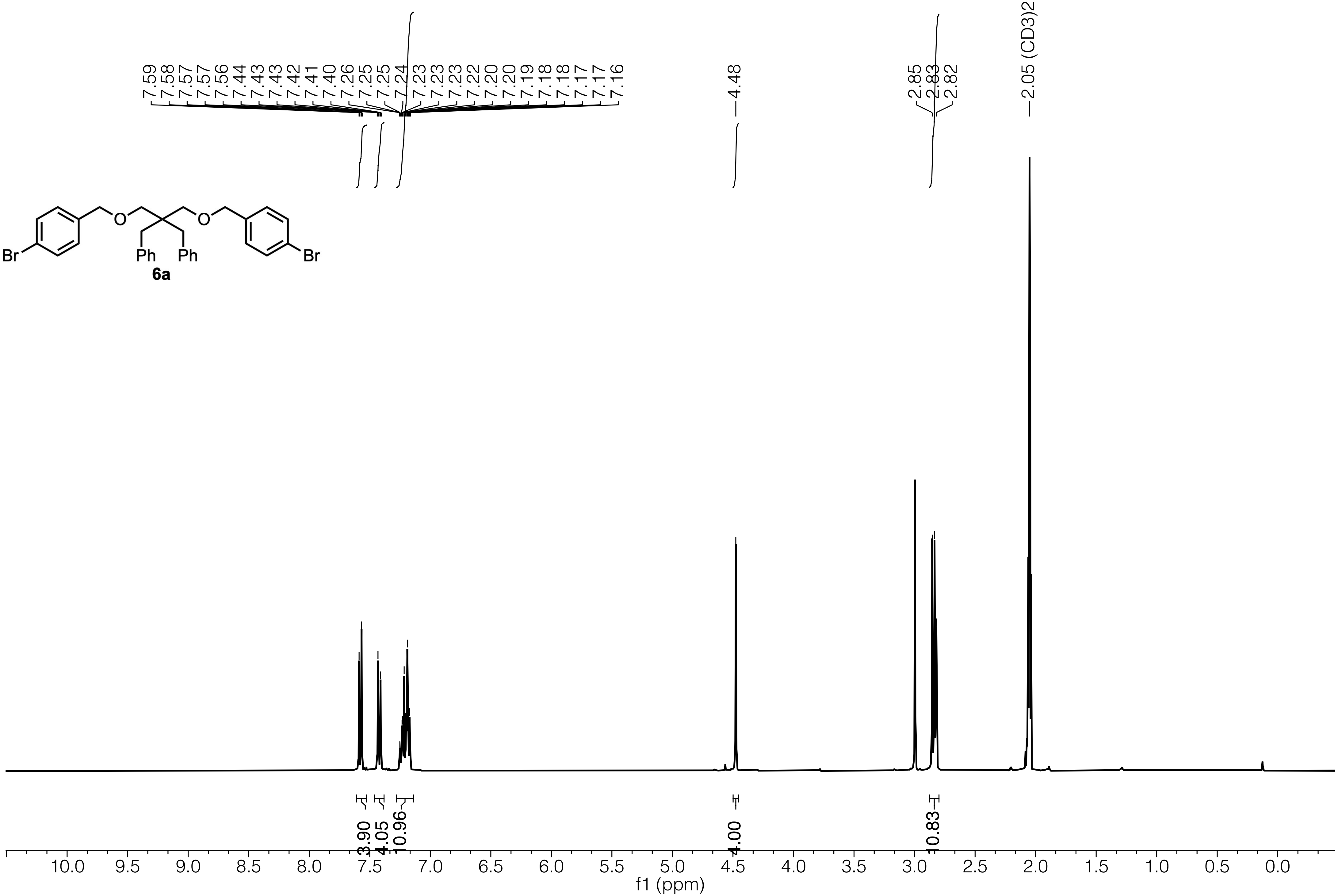

6a

KS-5-8-fr1
13C

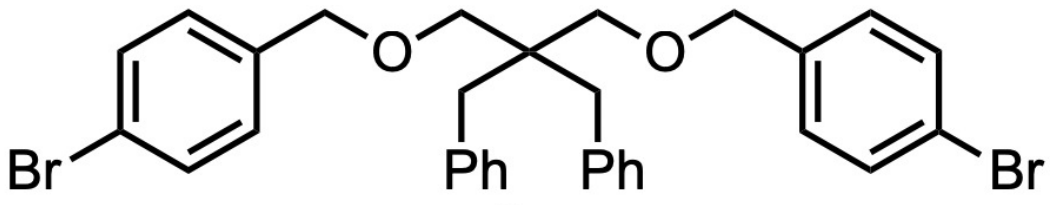

6a

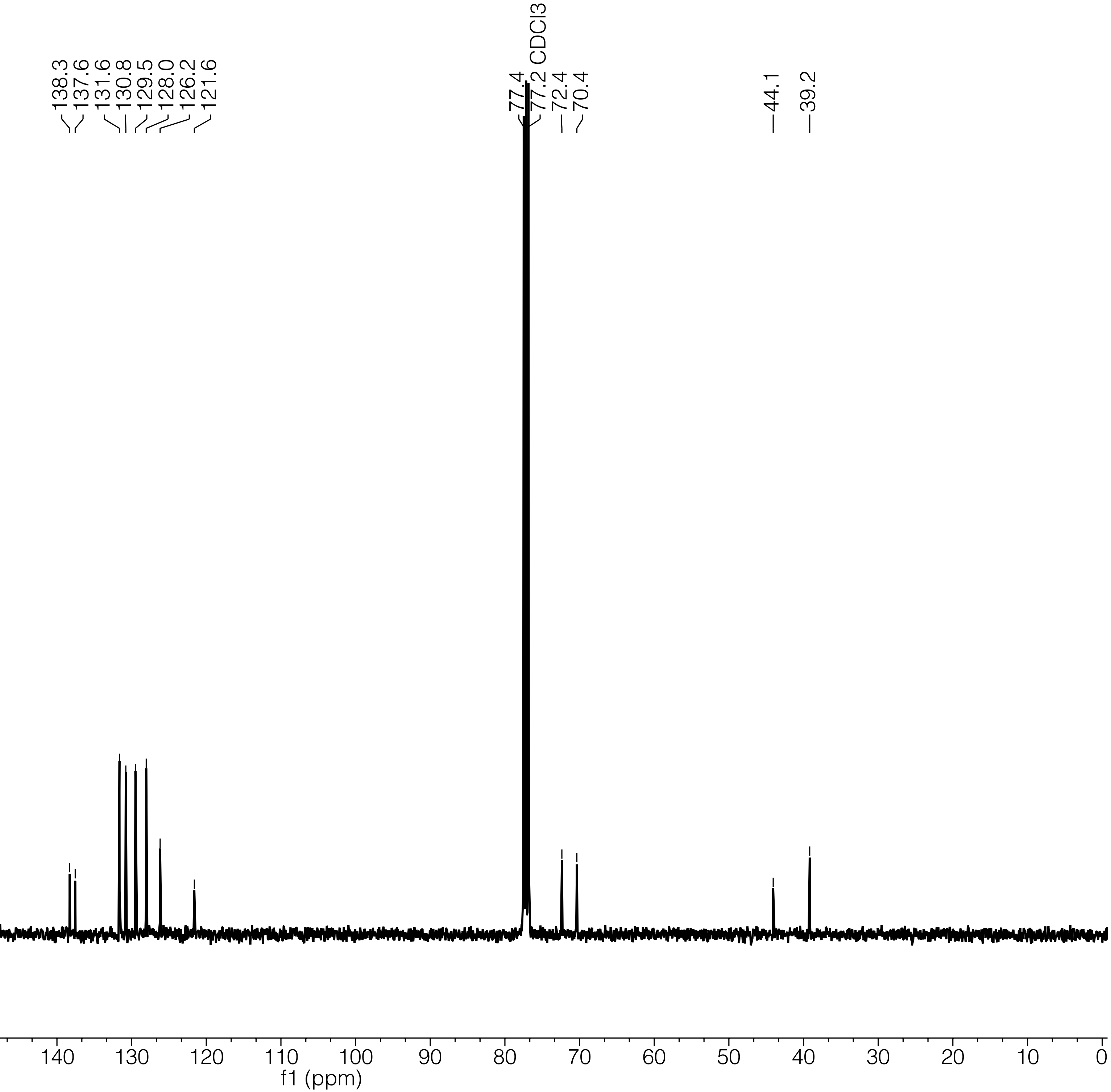

KS-4-55-fr
1H

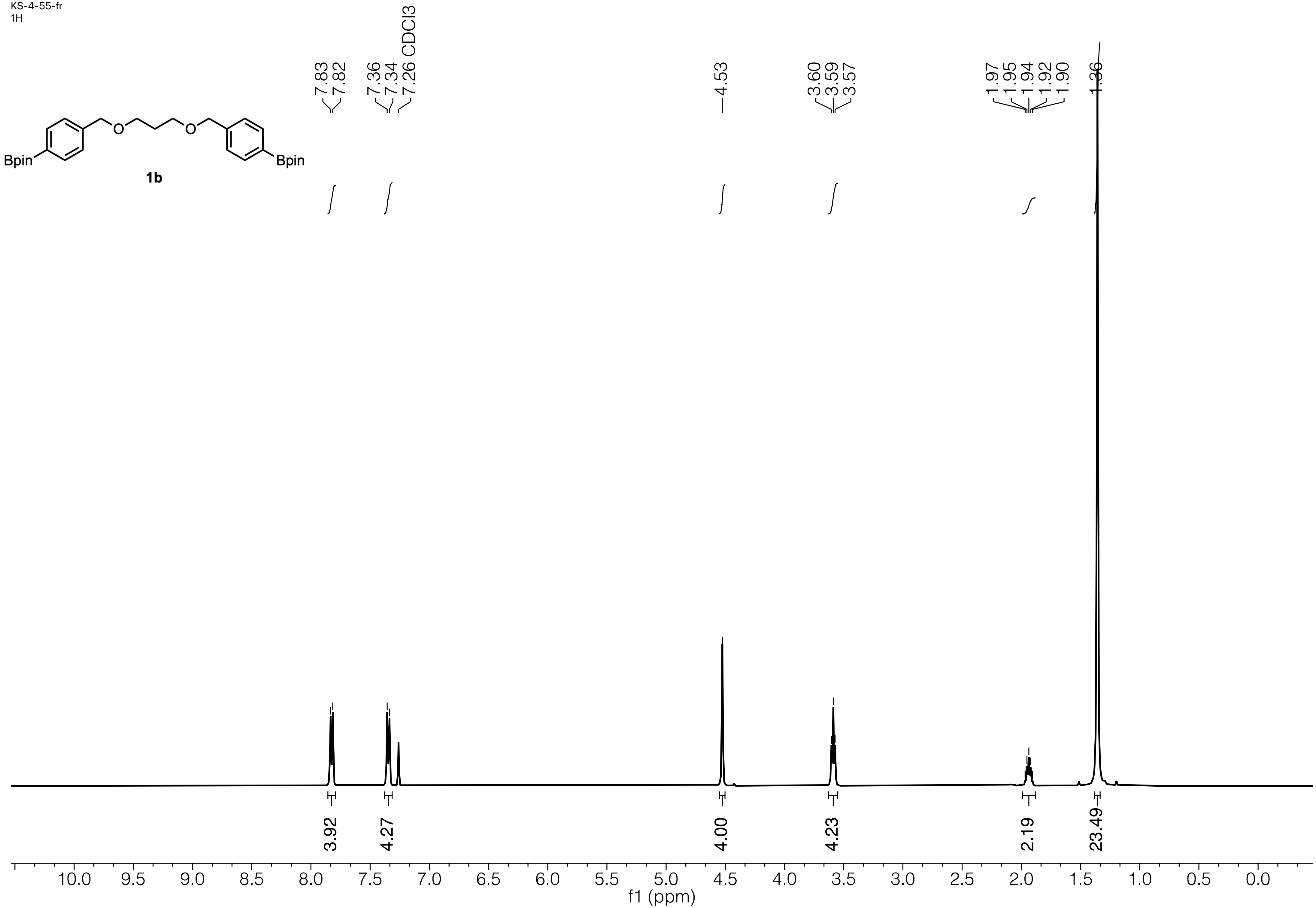

Bpin
Bpin
1b
7.83
7.82
7.36
7.34
7.26 CDCl3
4.53
3.60
3.59
3.57
1.97
1.95
1.94
1.92
1.90
1.36
3.92
4.27
4.00
4.23
2.19
23.49
10.0 9.5 9.0 8.5 8.0 7.5 7.0 6.5 6.0 5.5 5.0 4.5 4.0 3.5 3.0 2.5 2.0 1.5 1.0 0.5 0.0
f1 (ppm)

KS-4-55-fr
13C

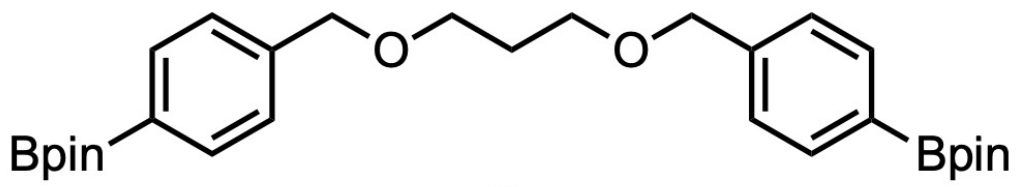

1b

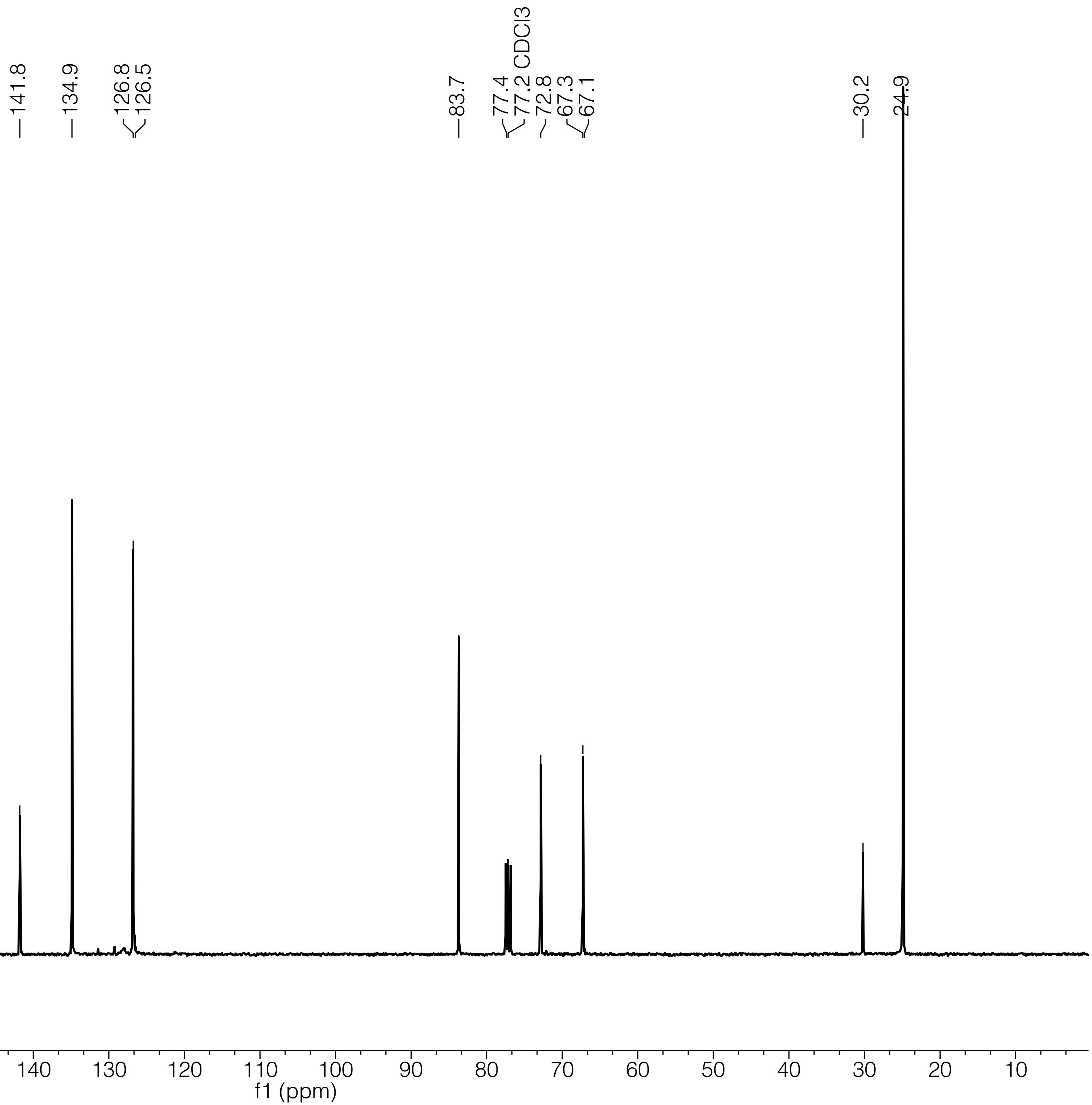

KS-4-55-11B
11B_No irr

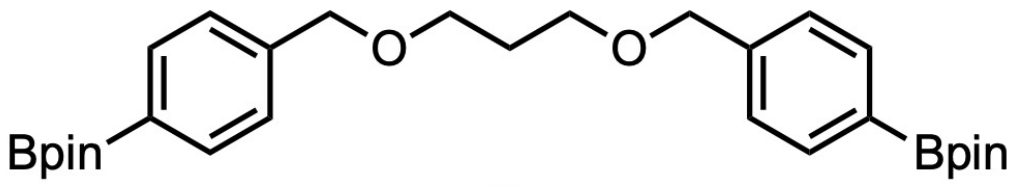

1b

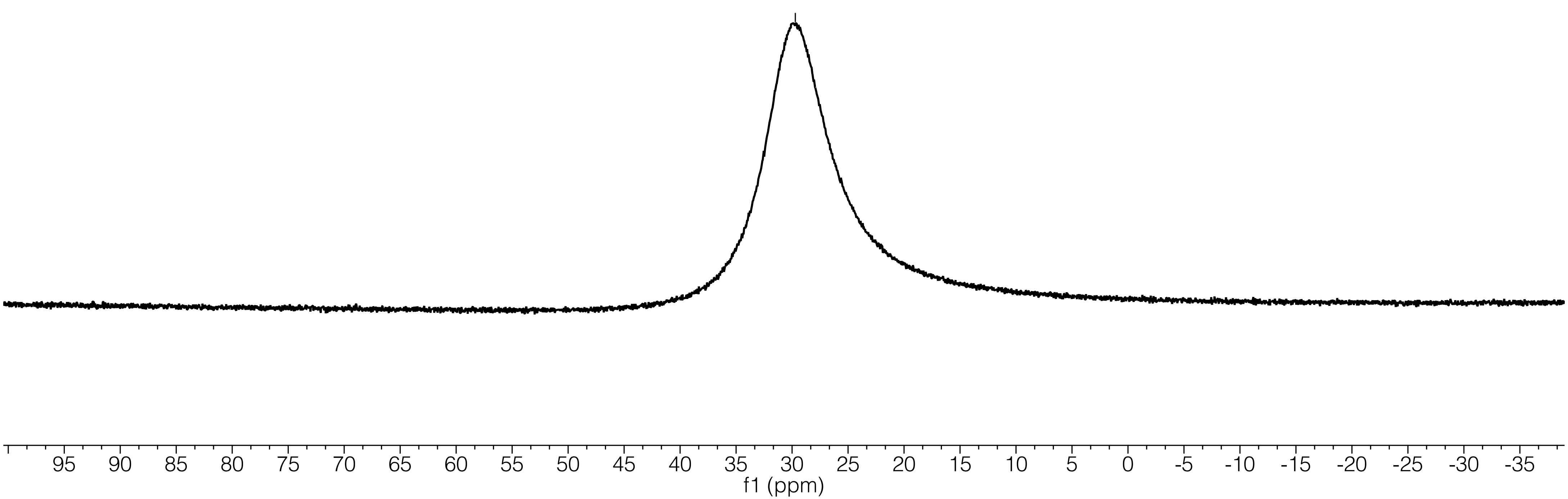

KS-4-54-fr
1H

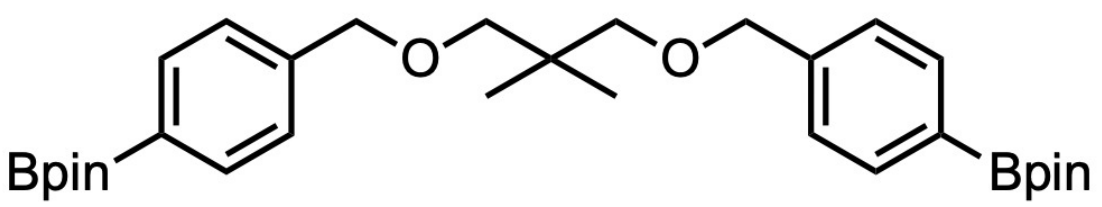

2b

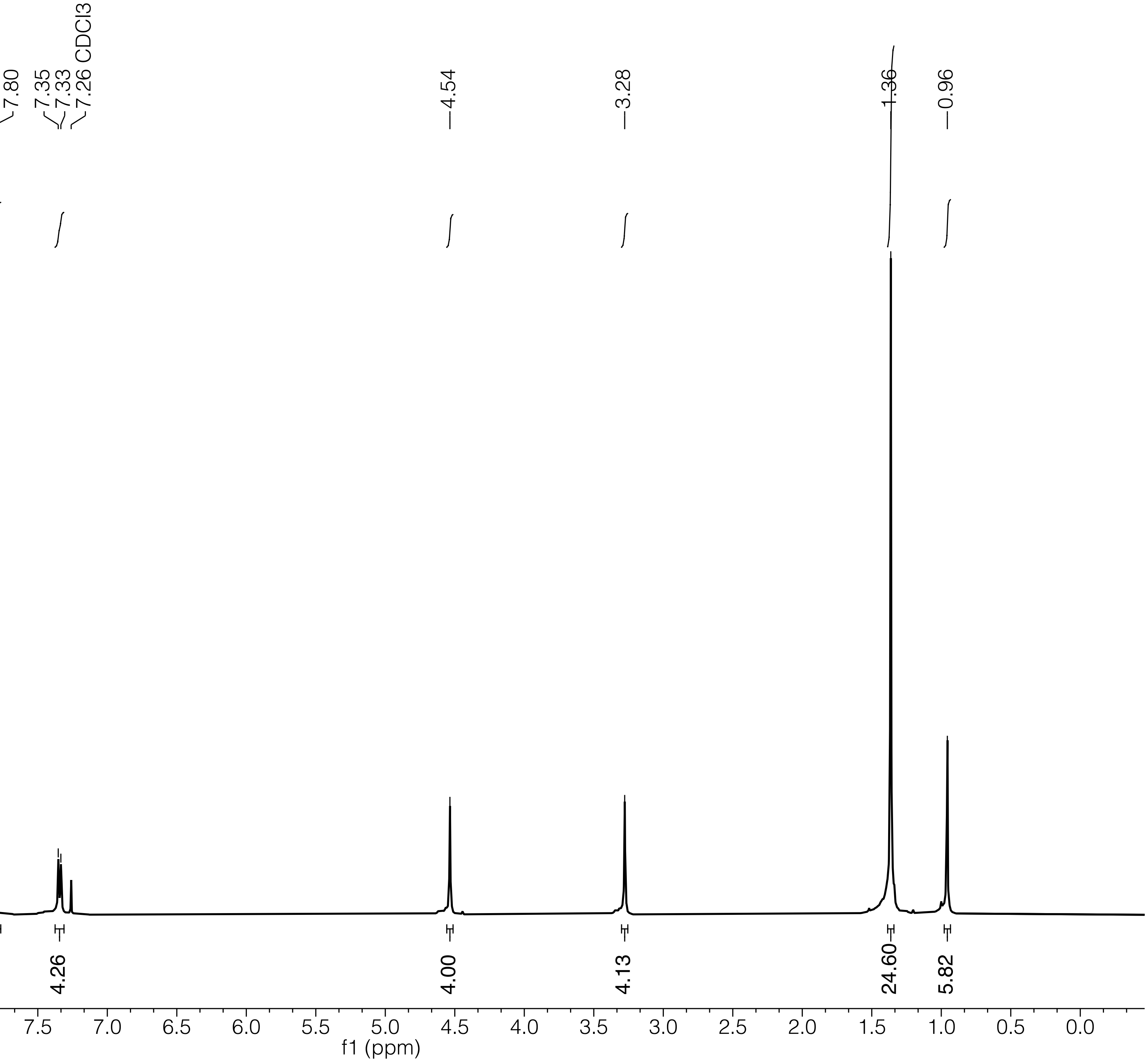

KS-4-54-fr
13C

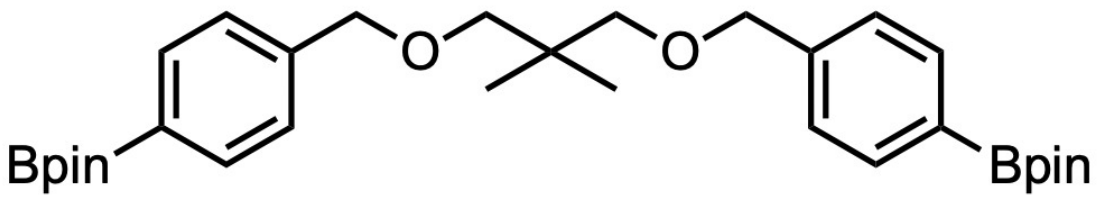

2b

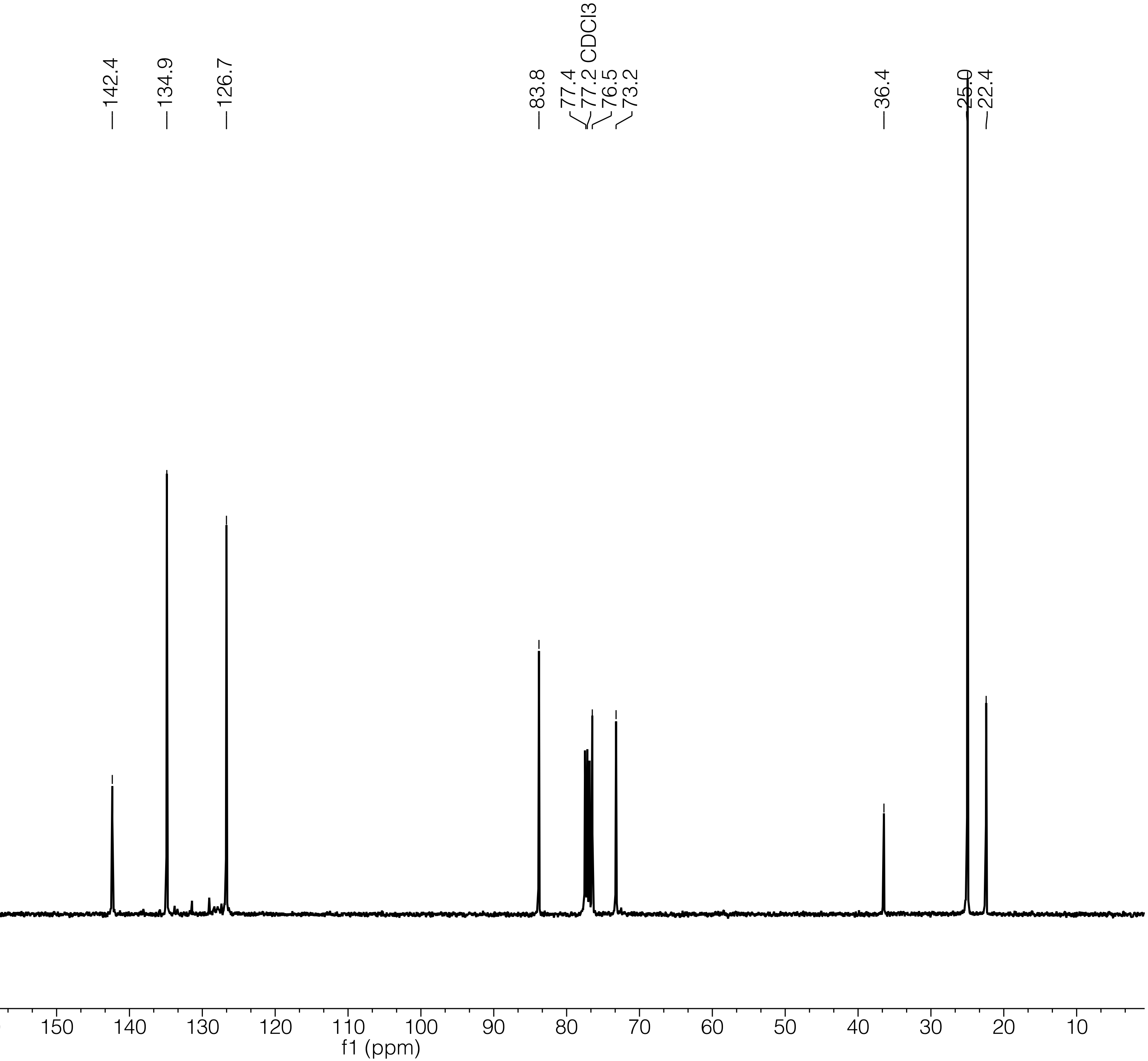

KS-4-54-11B
11B_No irr

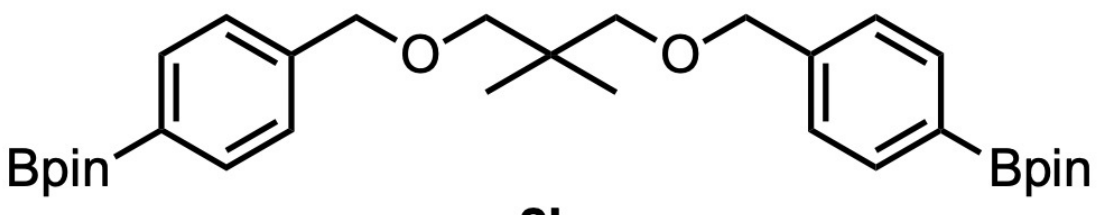

2b

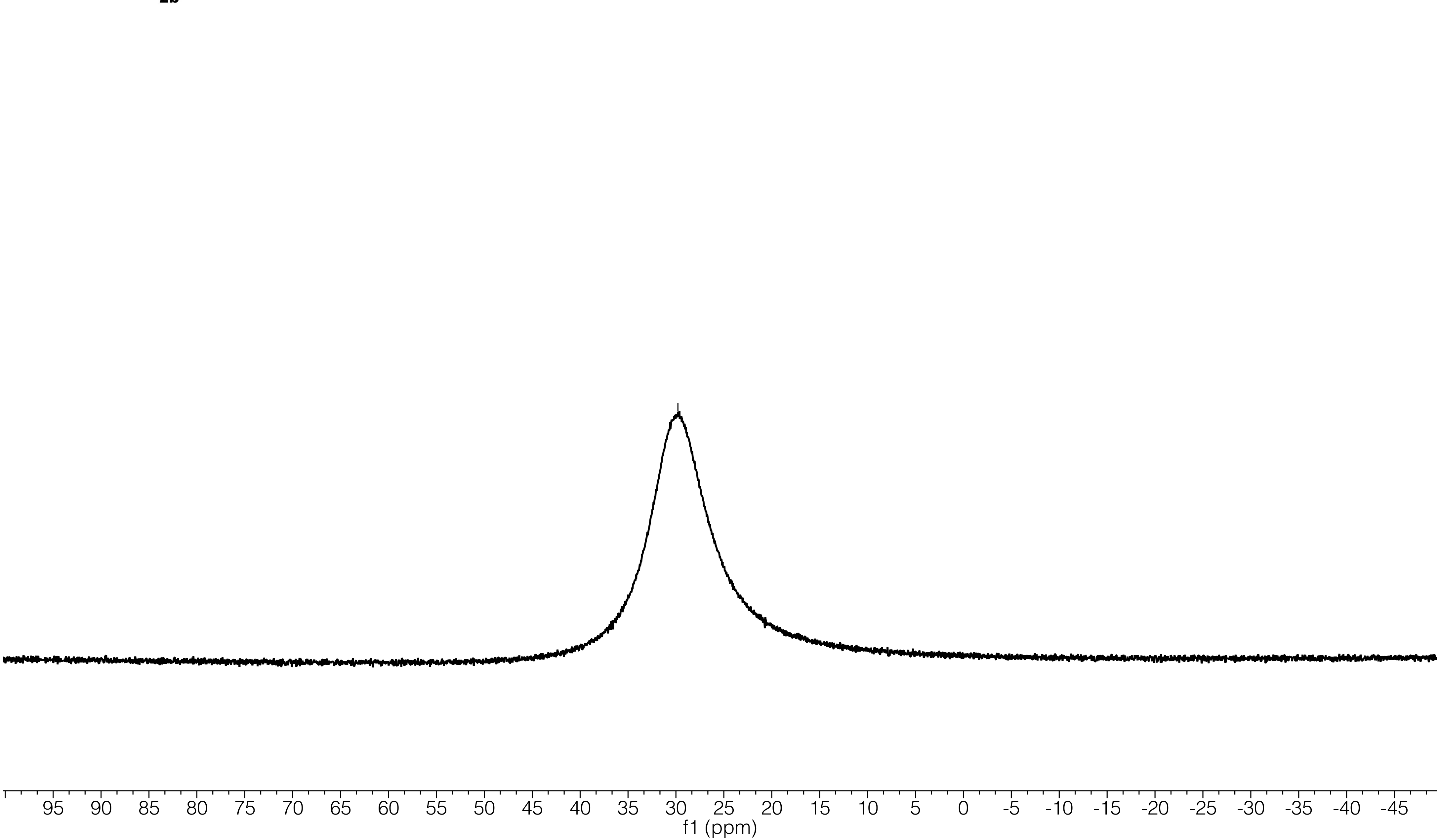

KS-4-58-fr
1H

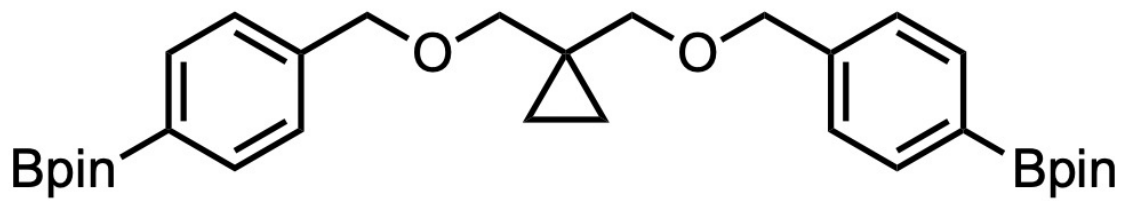

3b

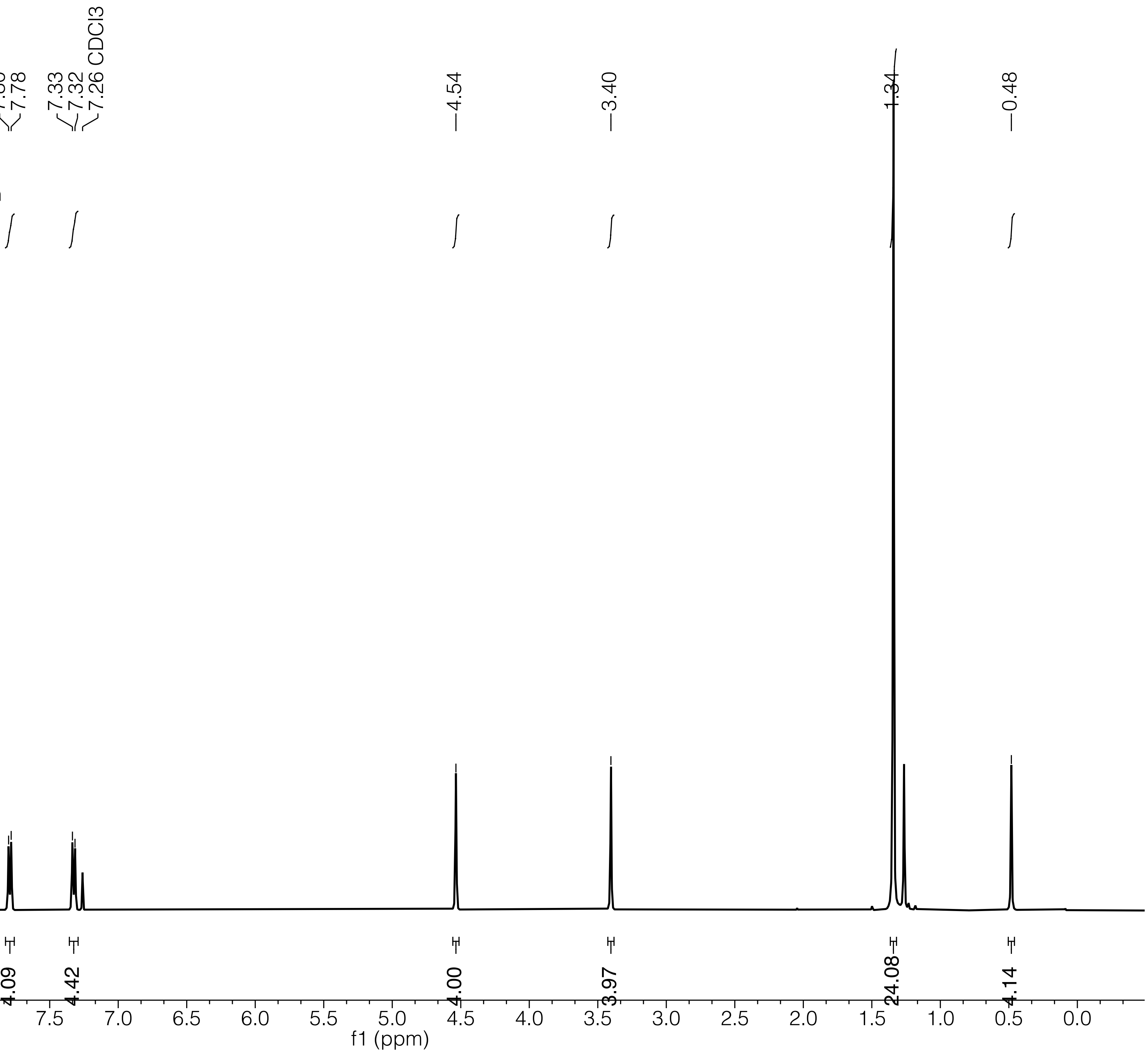

KS-4-58-fr
13C

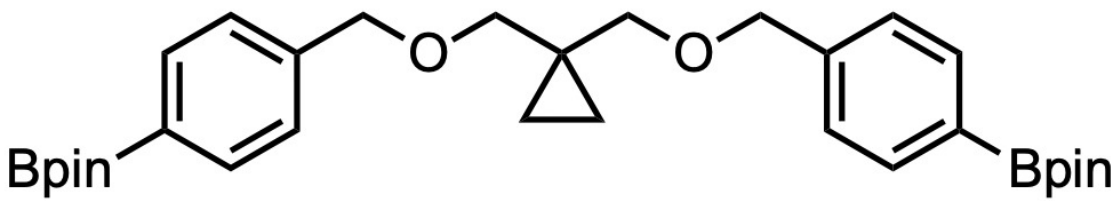

3b

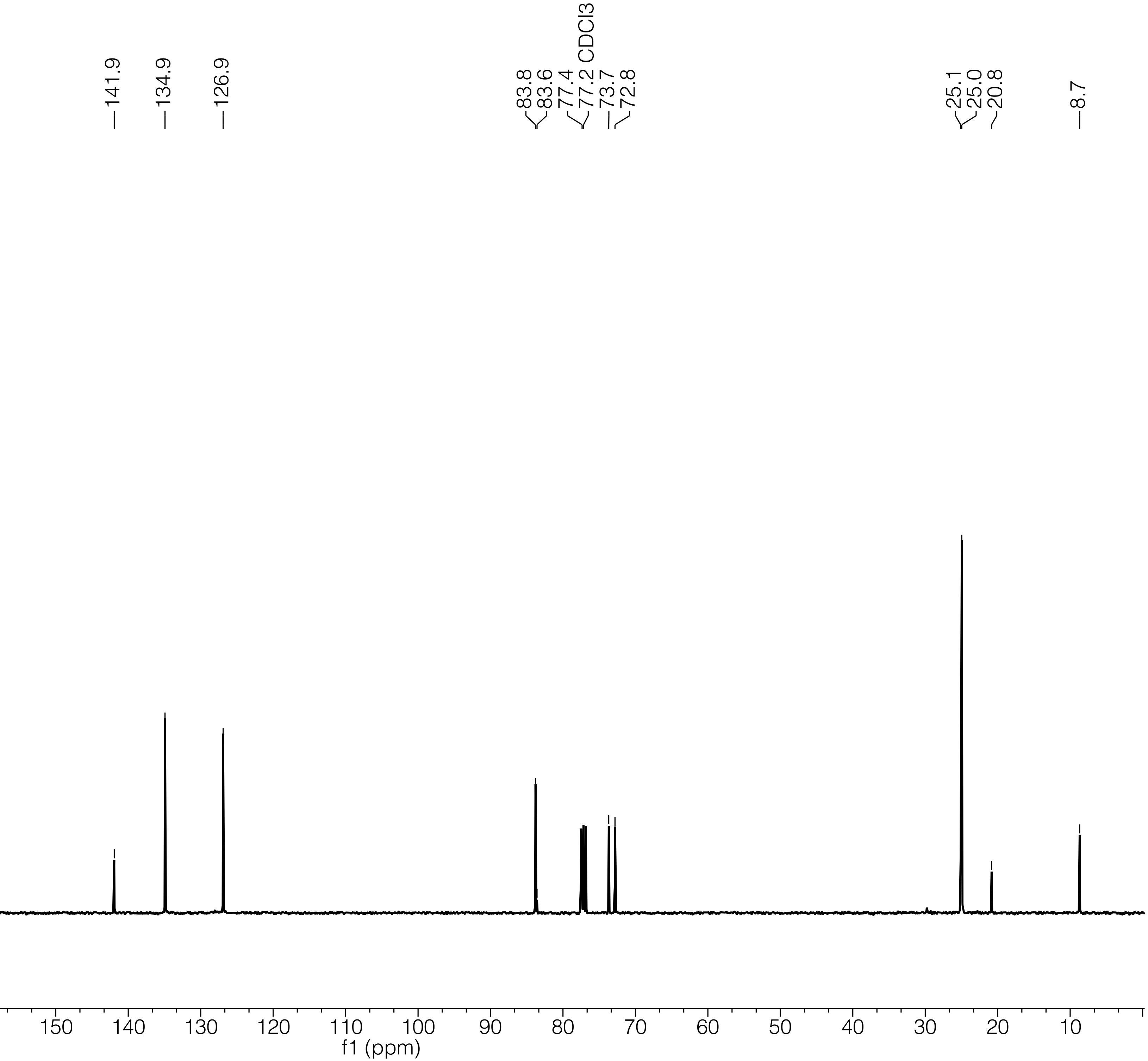

KS-4-58-E11B
11B_No irr

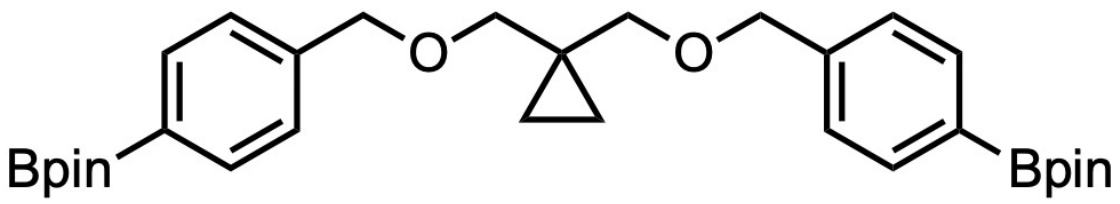

3b

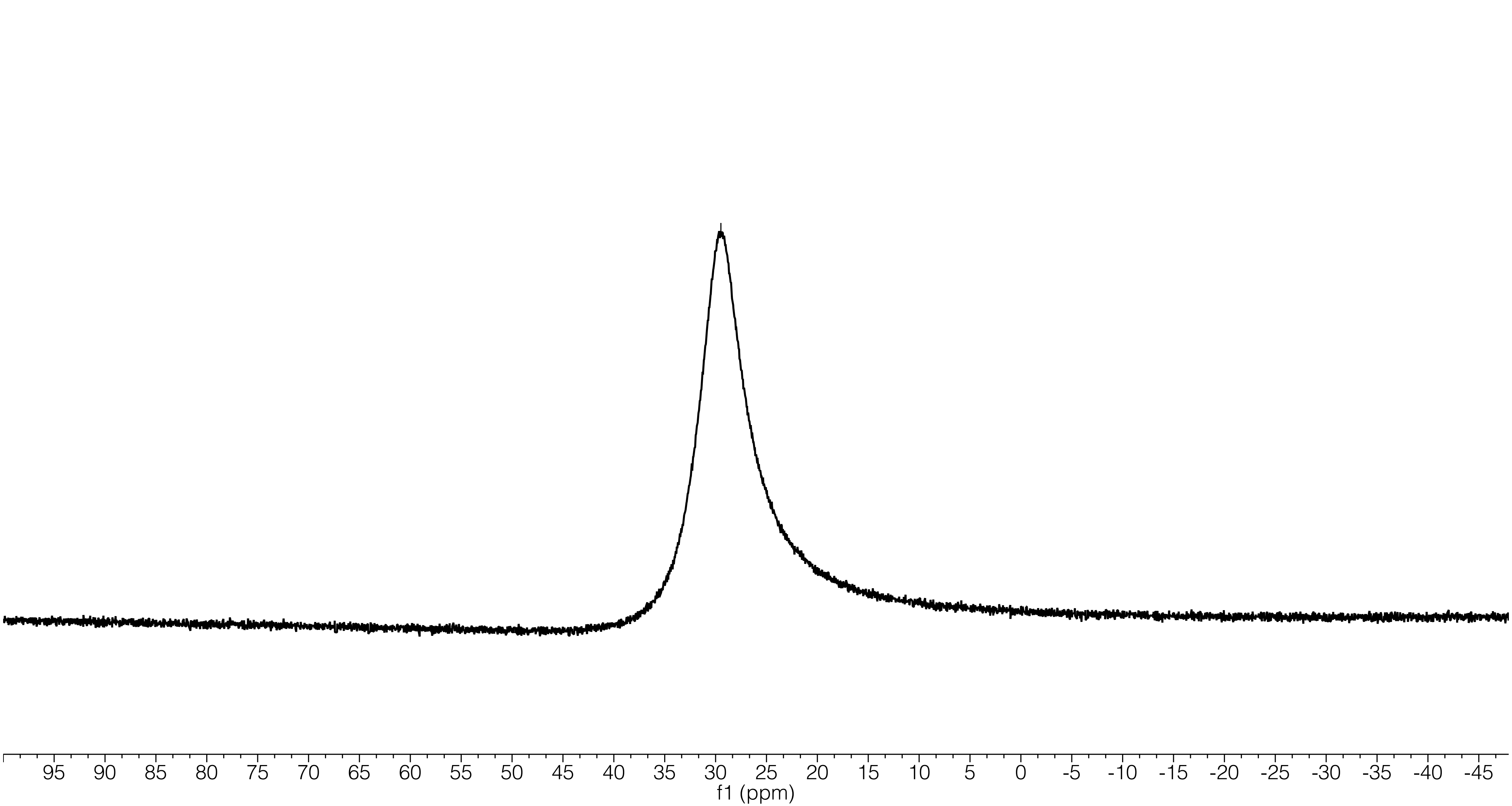

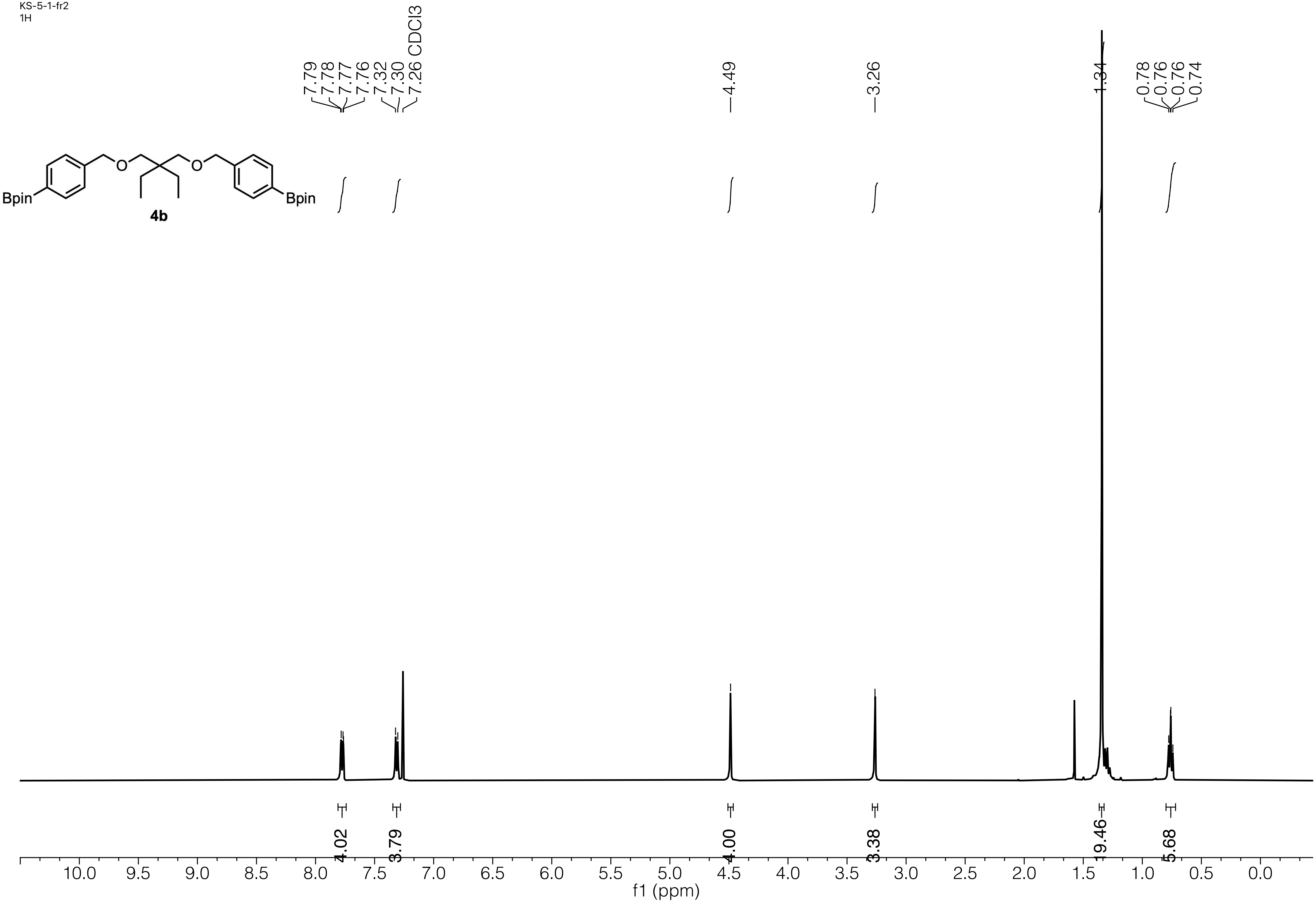
KS-5-1-fr2
1H
7.79
7.78
7.77
7.76
7.32
7.30
7.26 CDCl3
4.49
3.26
1.34
0.78
0.76
0.76
0.74
Bpin
Bpin
4b
4.02
3.79
4.00
3.38
19.46
5.68
10.0 9.5 9.0 8.5 8.0 7.5 7.0 6.5 6.0 5.5 5.0 4.5 4.0 3.5 3.0 2.5 2.0 1.5 1.0 0.5 0.0
f1 (ppm)

KS-5-1
13C

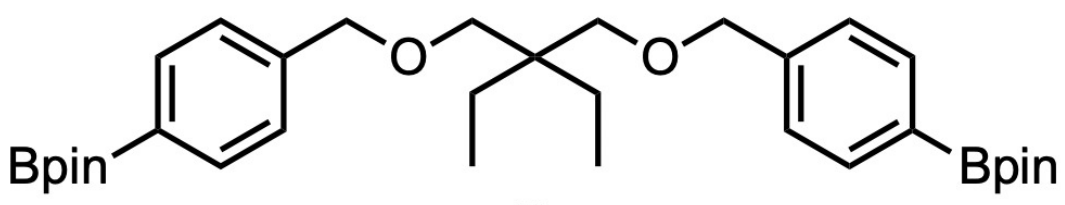
Bpin
O
O
Bpin

4b

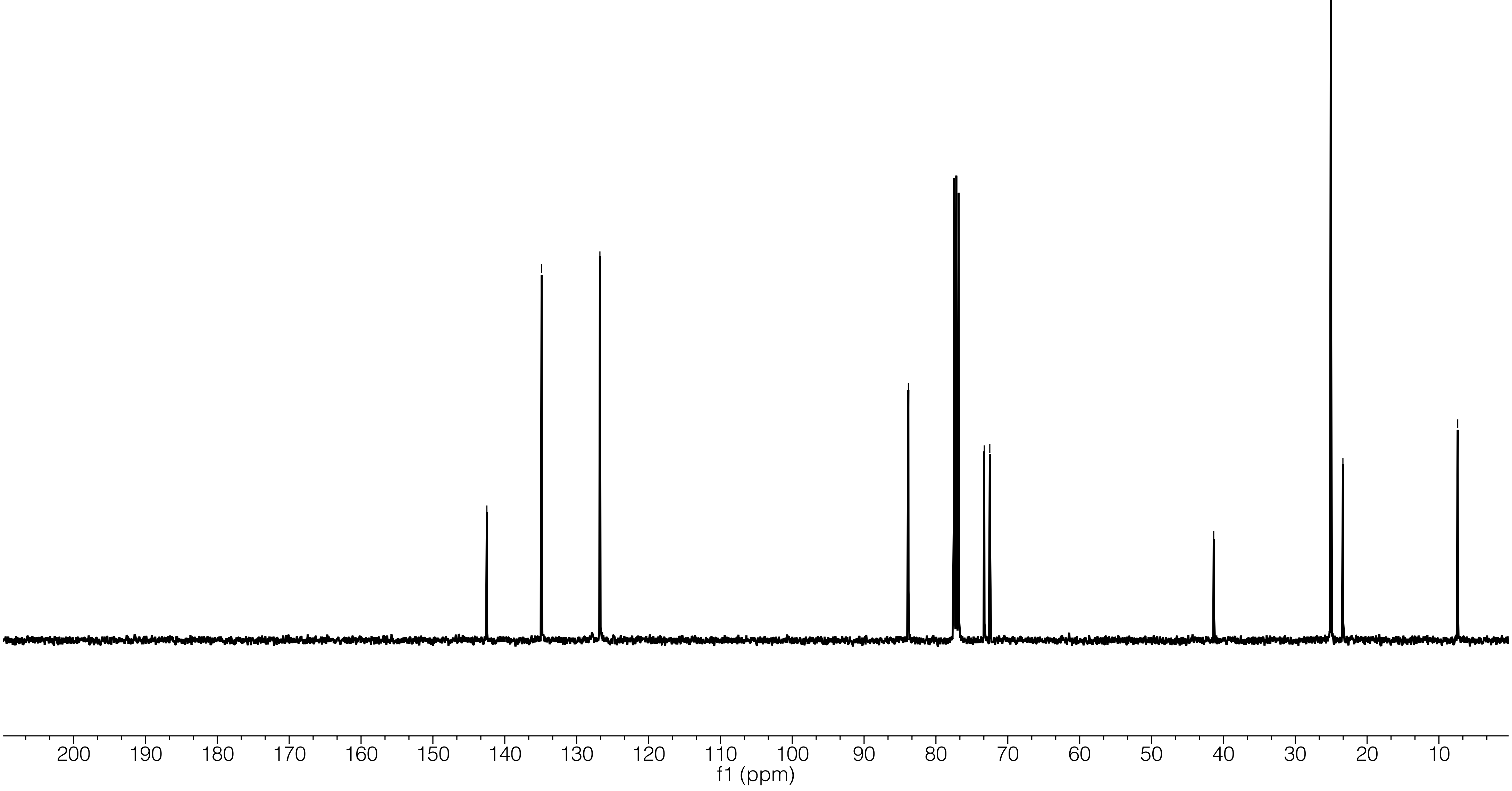
142.5
134.9
126.8
83.8
77.4
77.2 CDCl3
73.3
72.5
41.3
25.0
23.4
7.4
200 190 180 170 160 150 140 130 120 110 100 90 80 70 60 50 40 30 20 10
f1 (ppm)

KS-5-1
11B_No irr

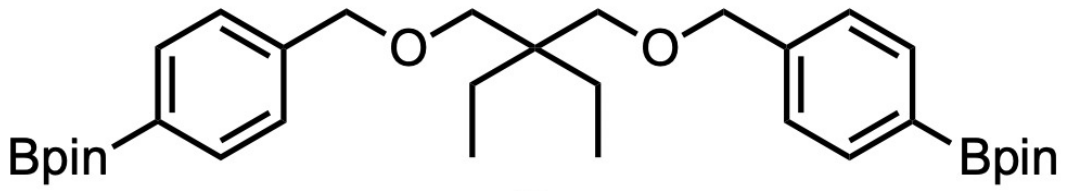

4b

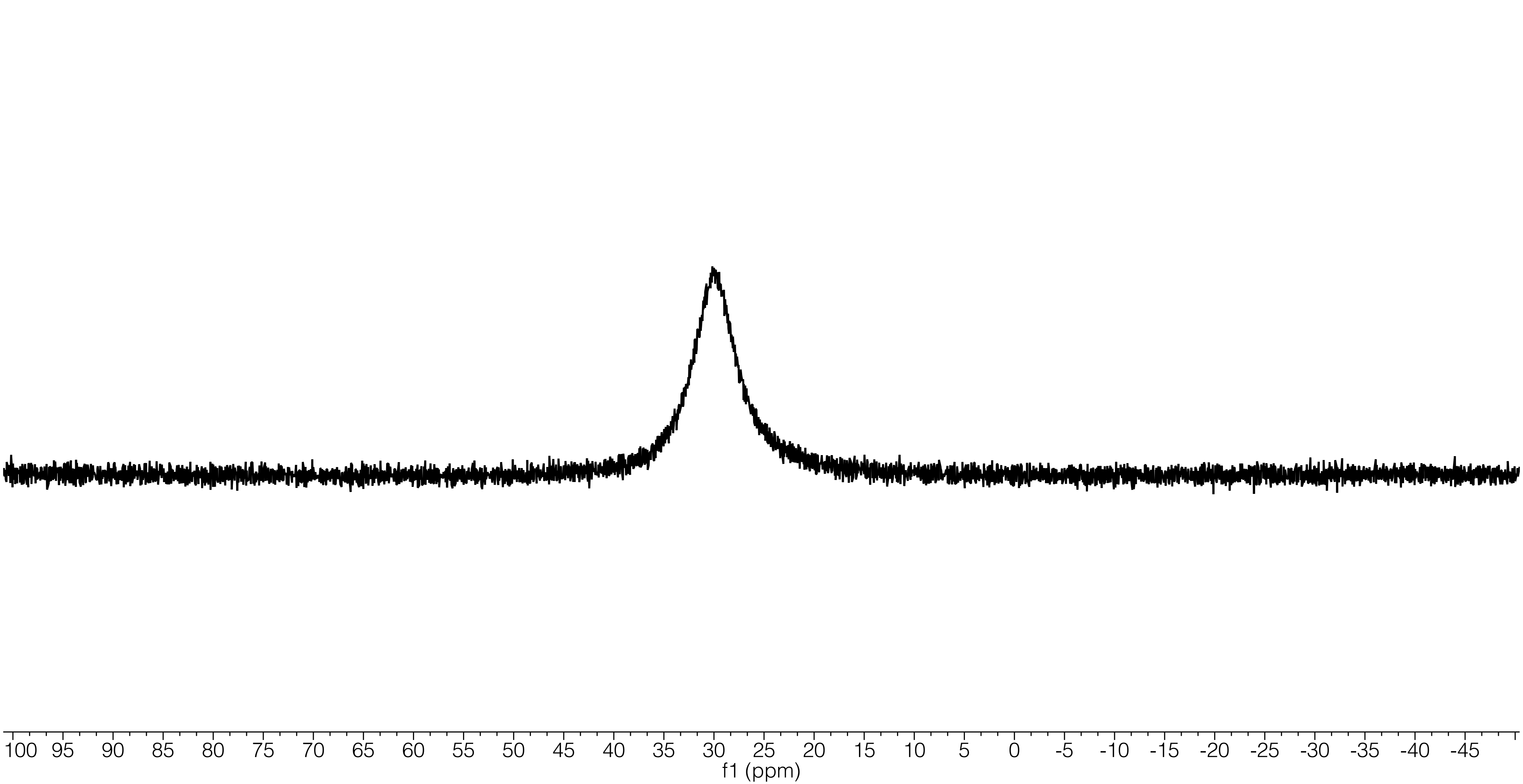

KS-5-11-fr1
1H

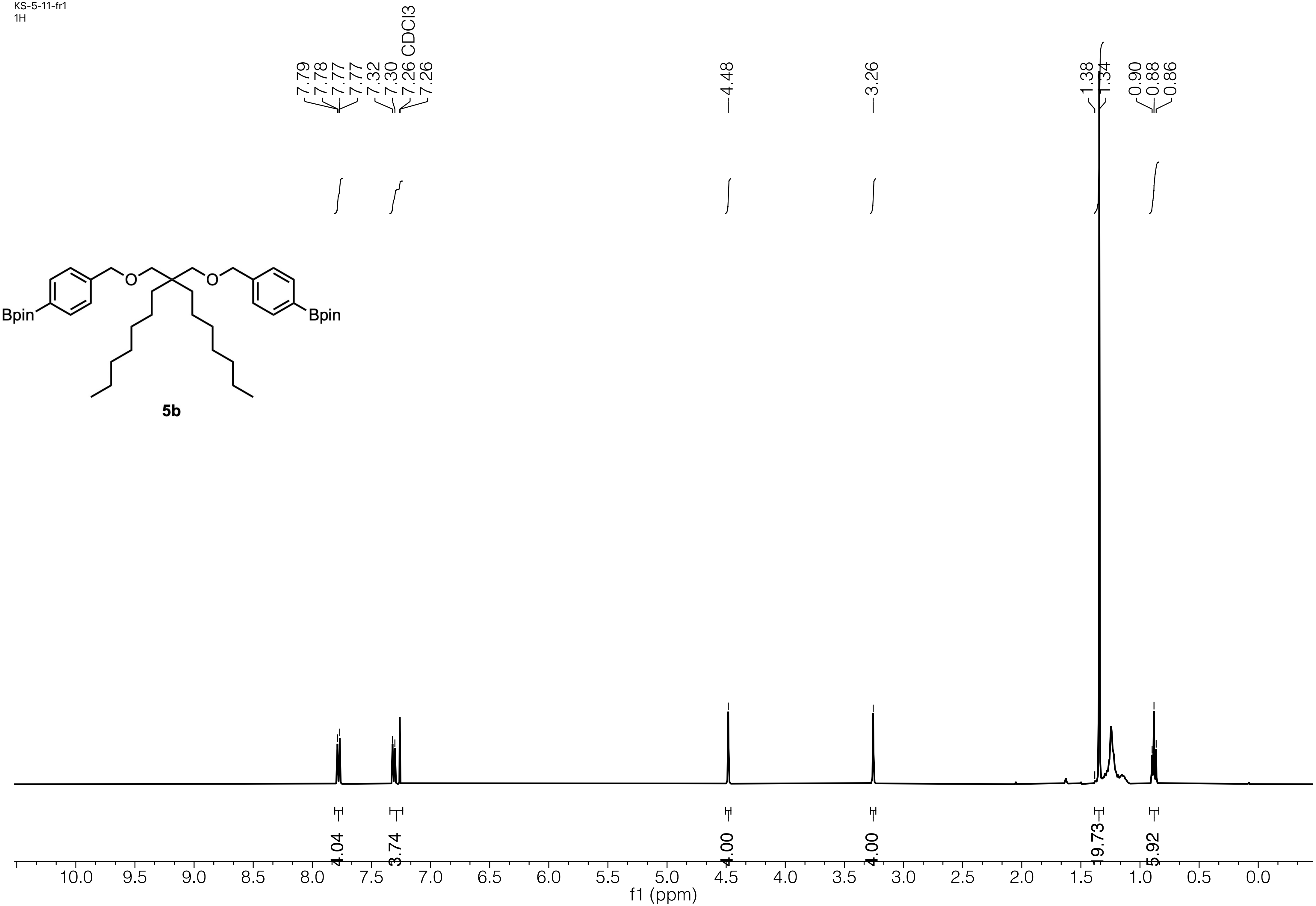

5b

KS-5-11-fr
13C

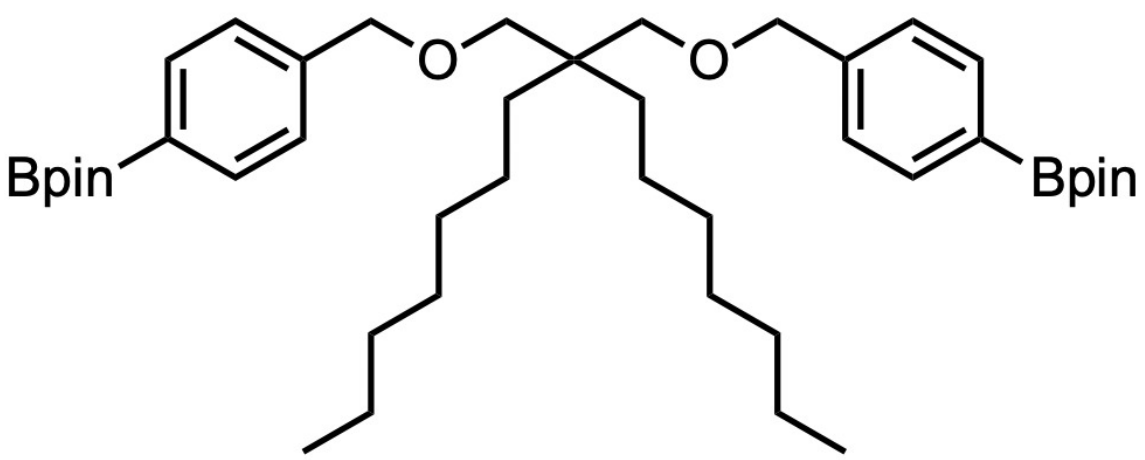

5b

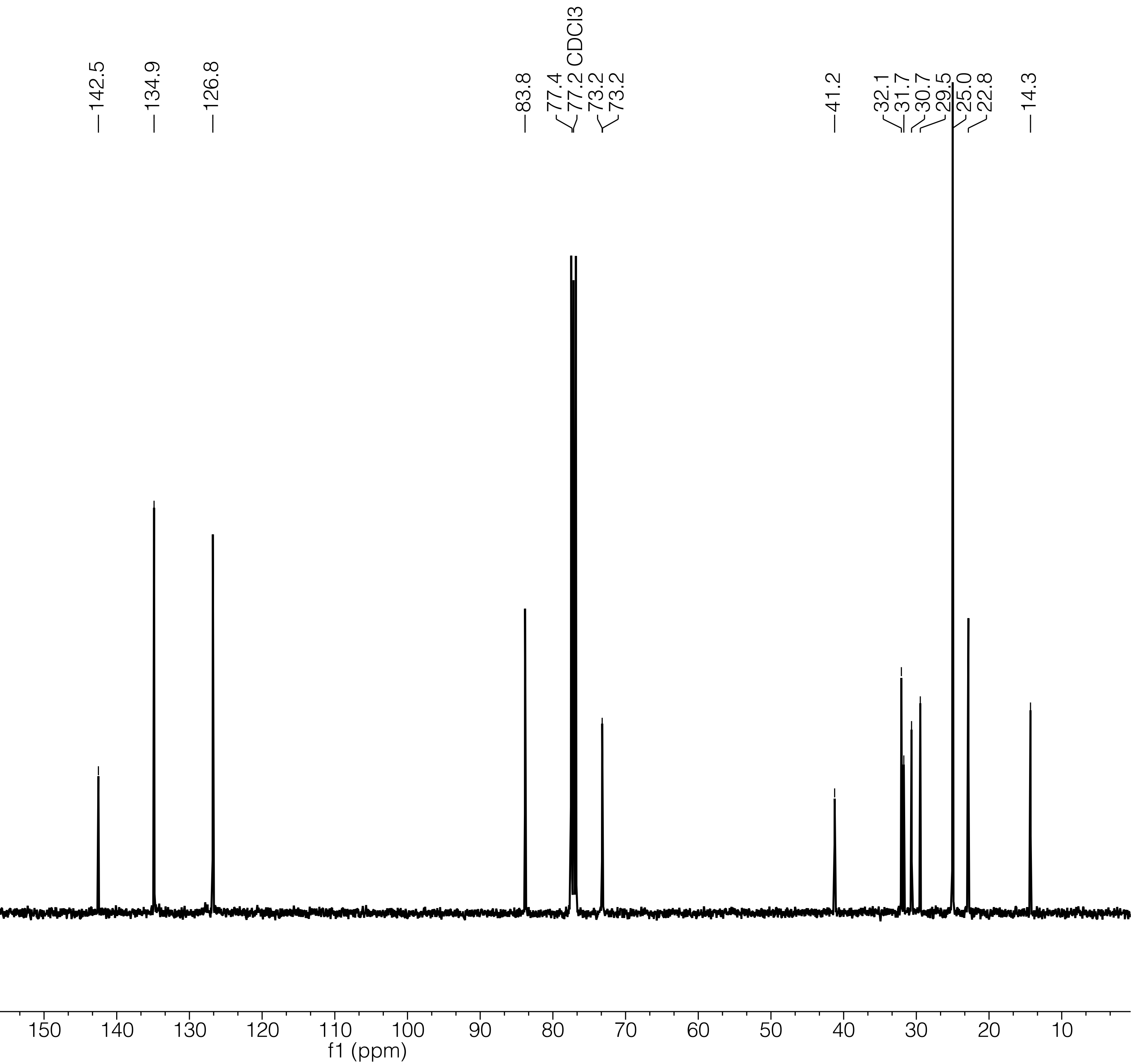

KS-5-11-fr
11B_No irr

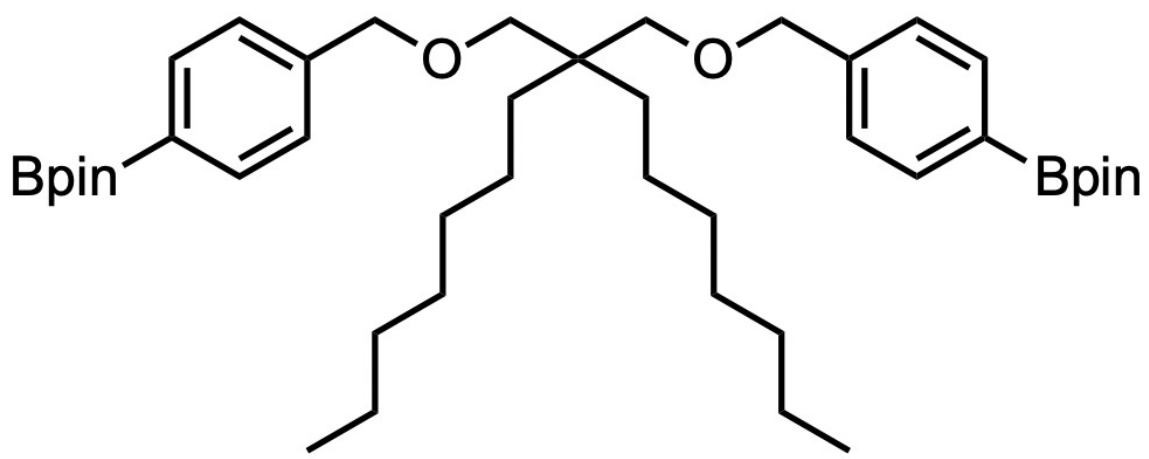

5b

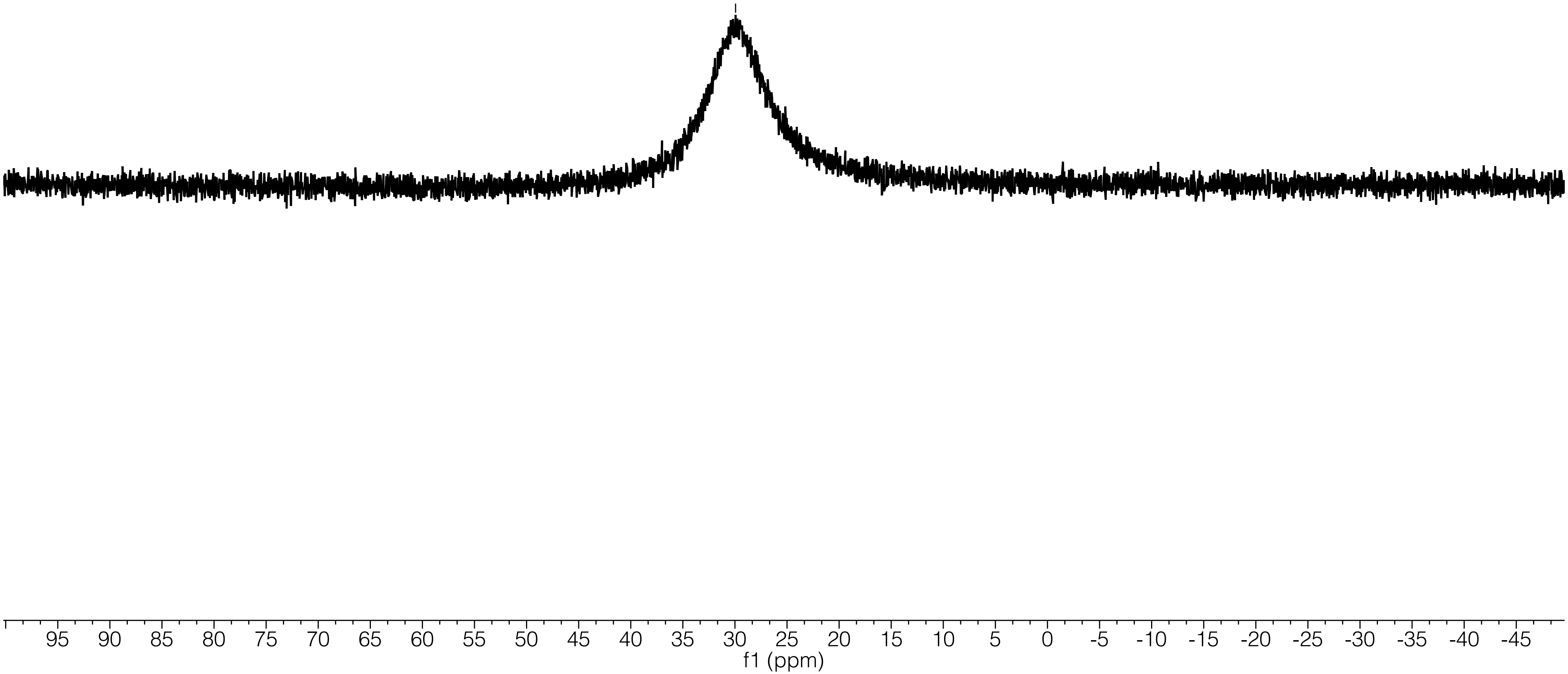

KS-5-10-fr
1H

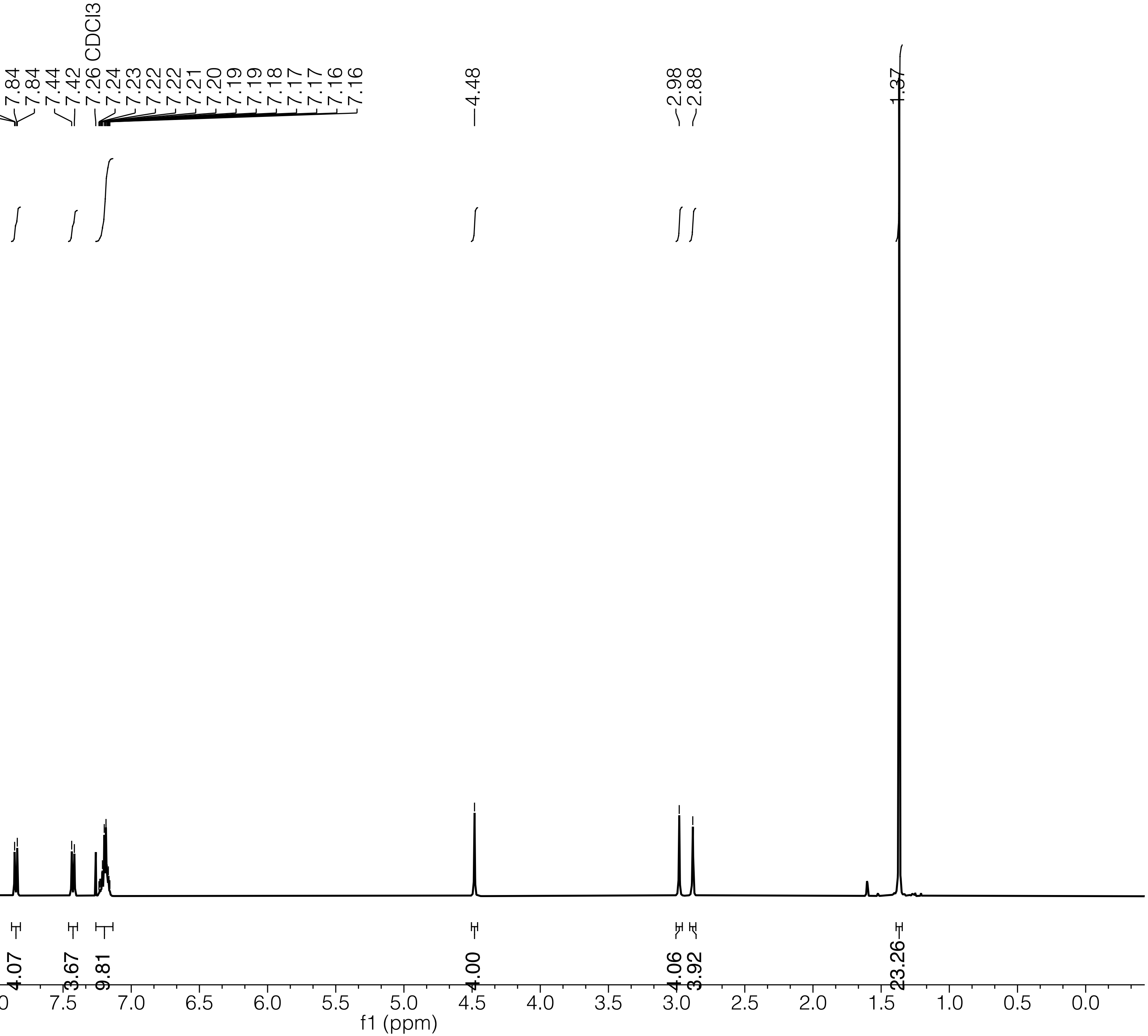

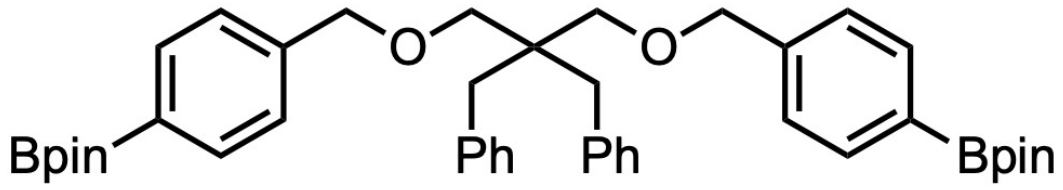

6b

KS-5-10-fr
13C

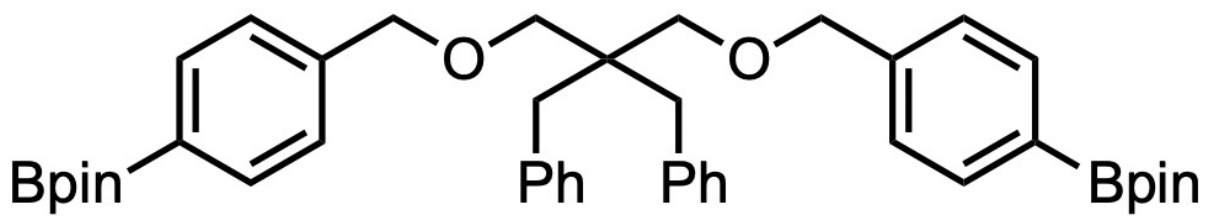

6b

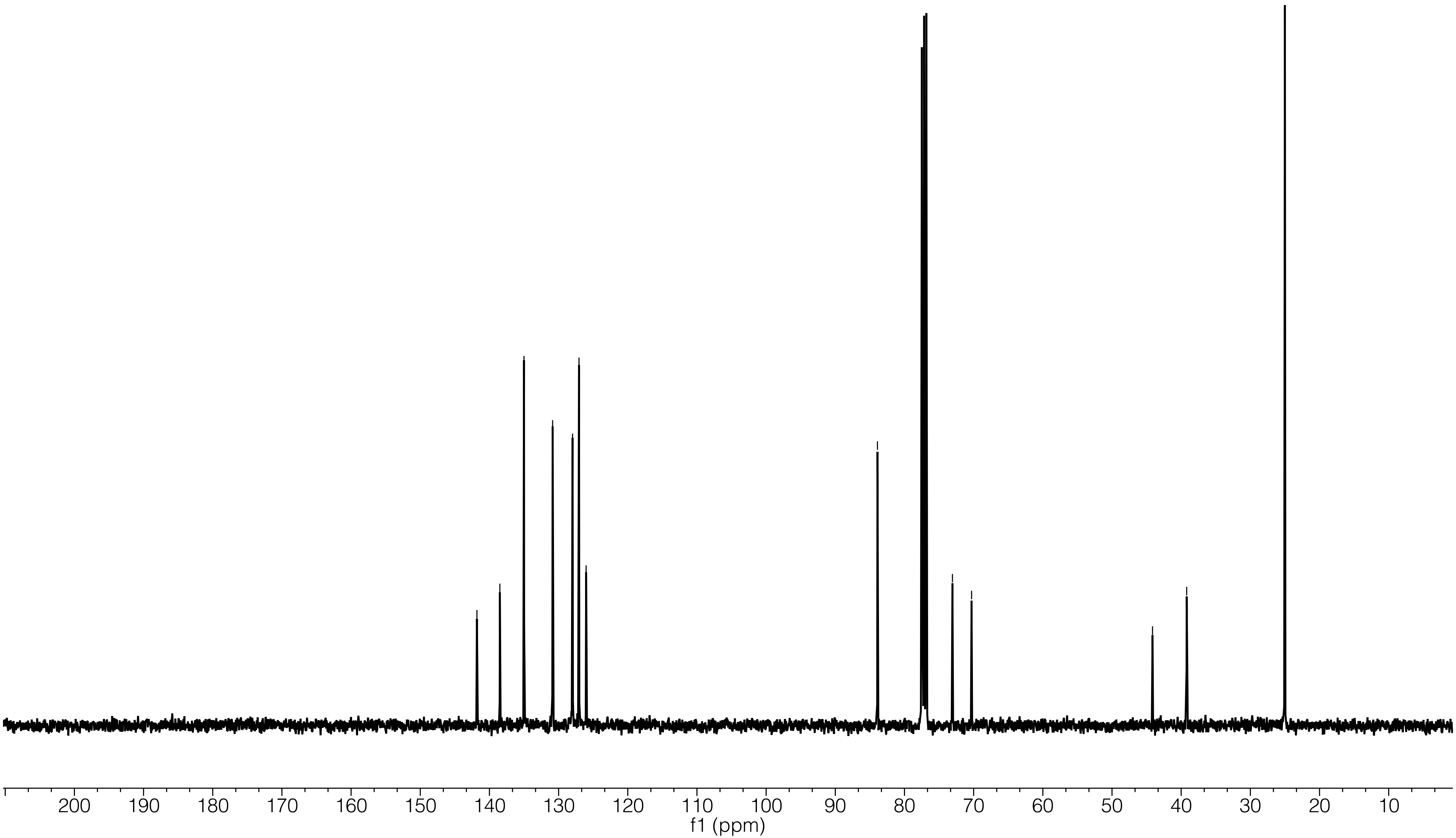

KS-5-10-fr
11B_No irr

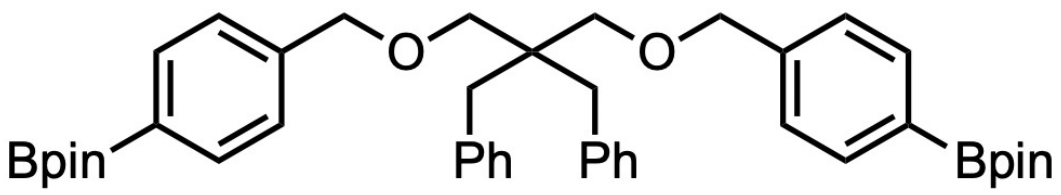

6b

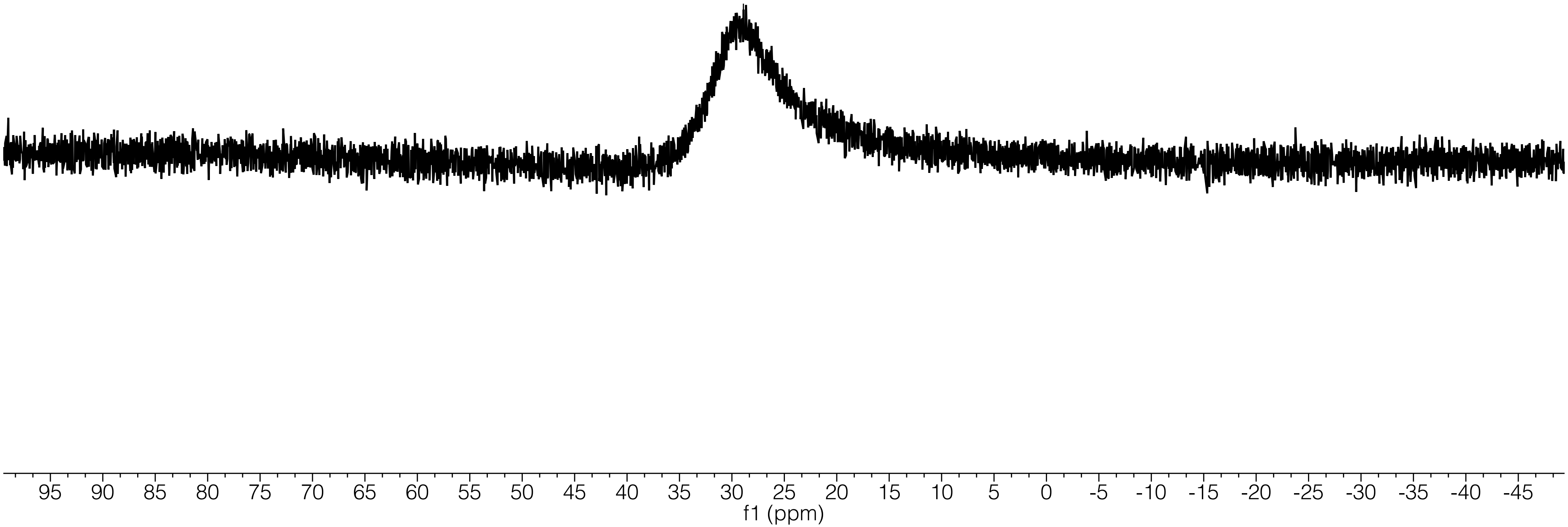

KS-2-68-GPC2
1H

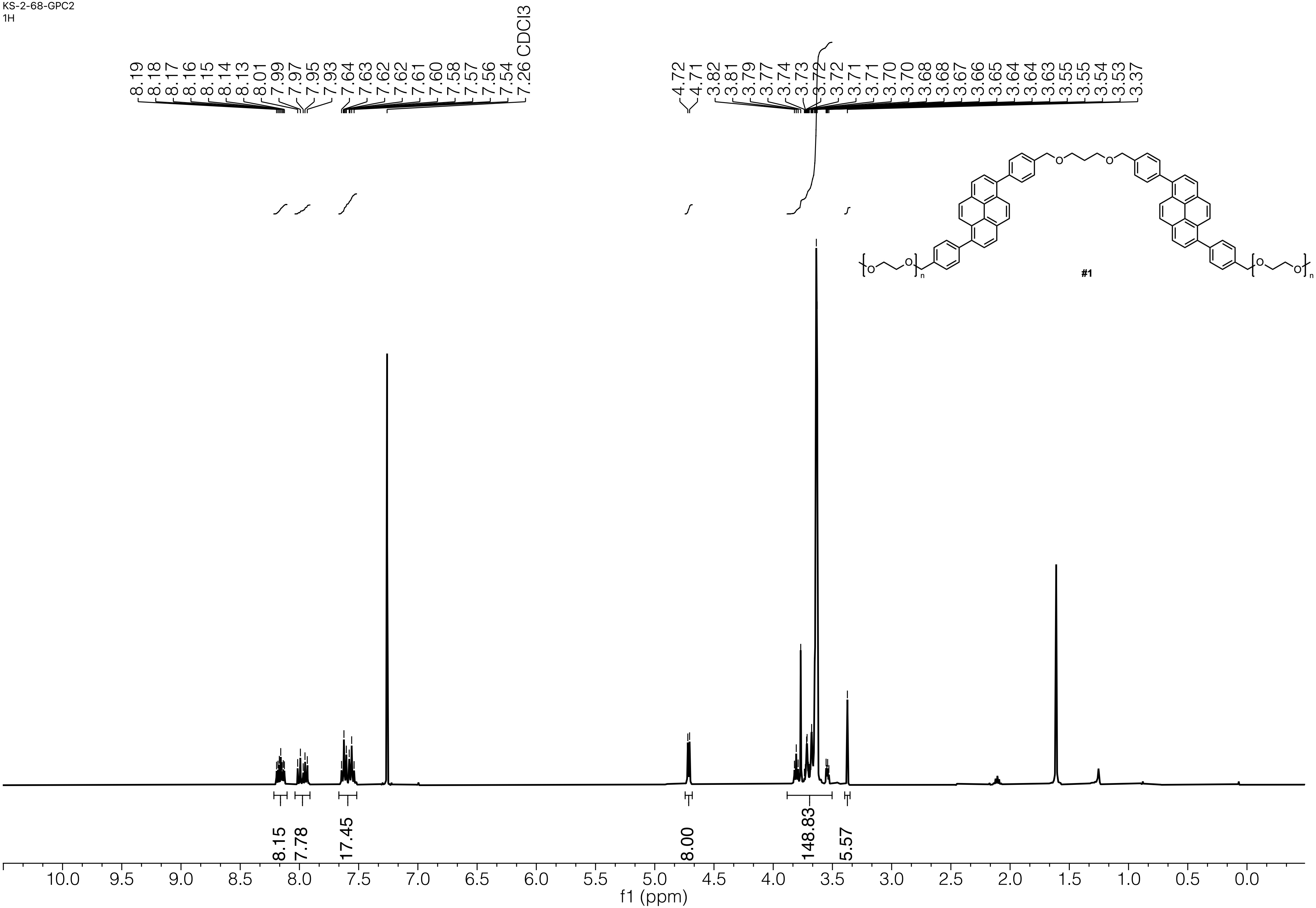

KS-2-68 13C
13C

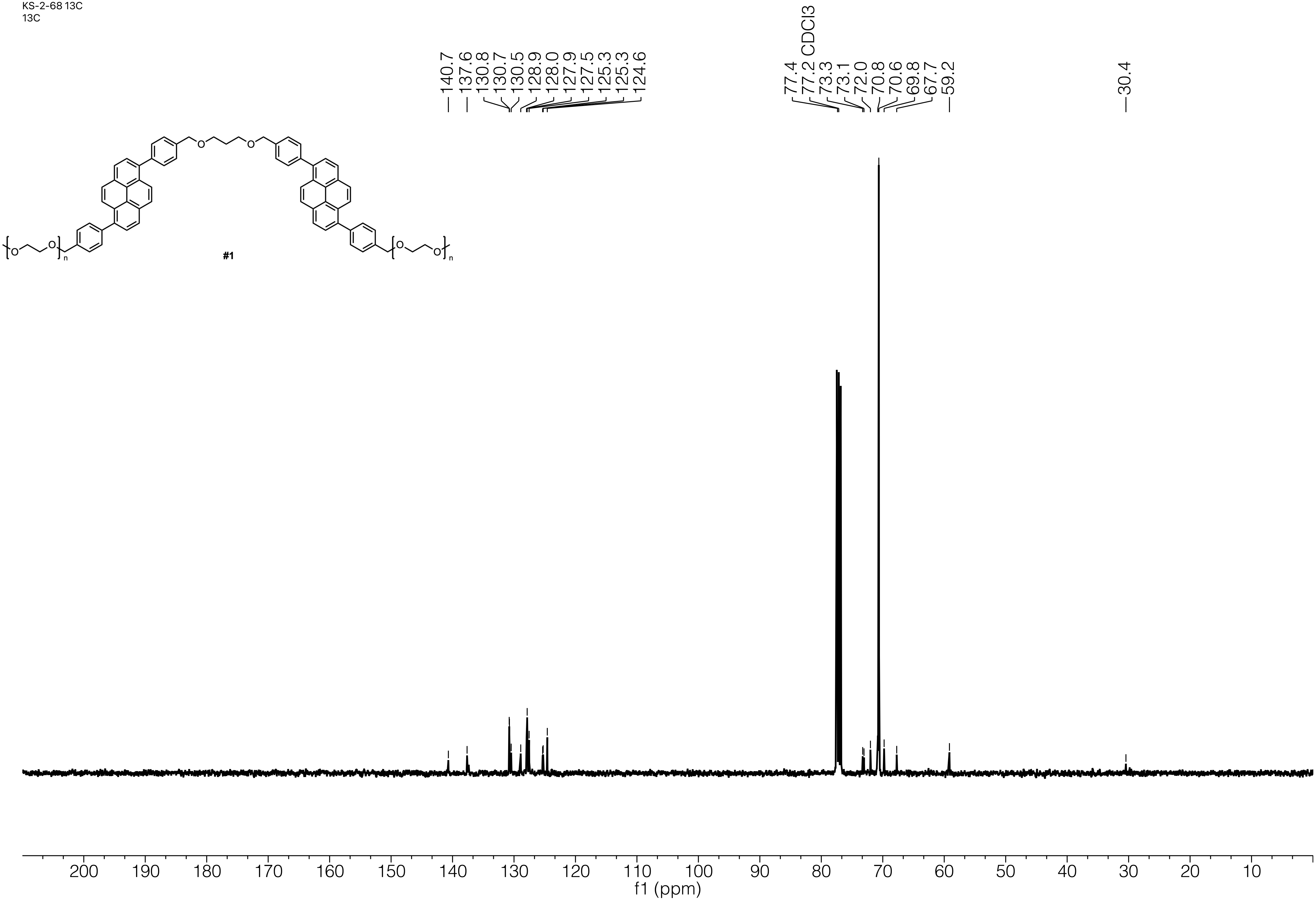

KS-4-86-GPC
1H

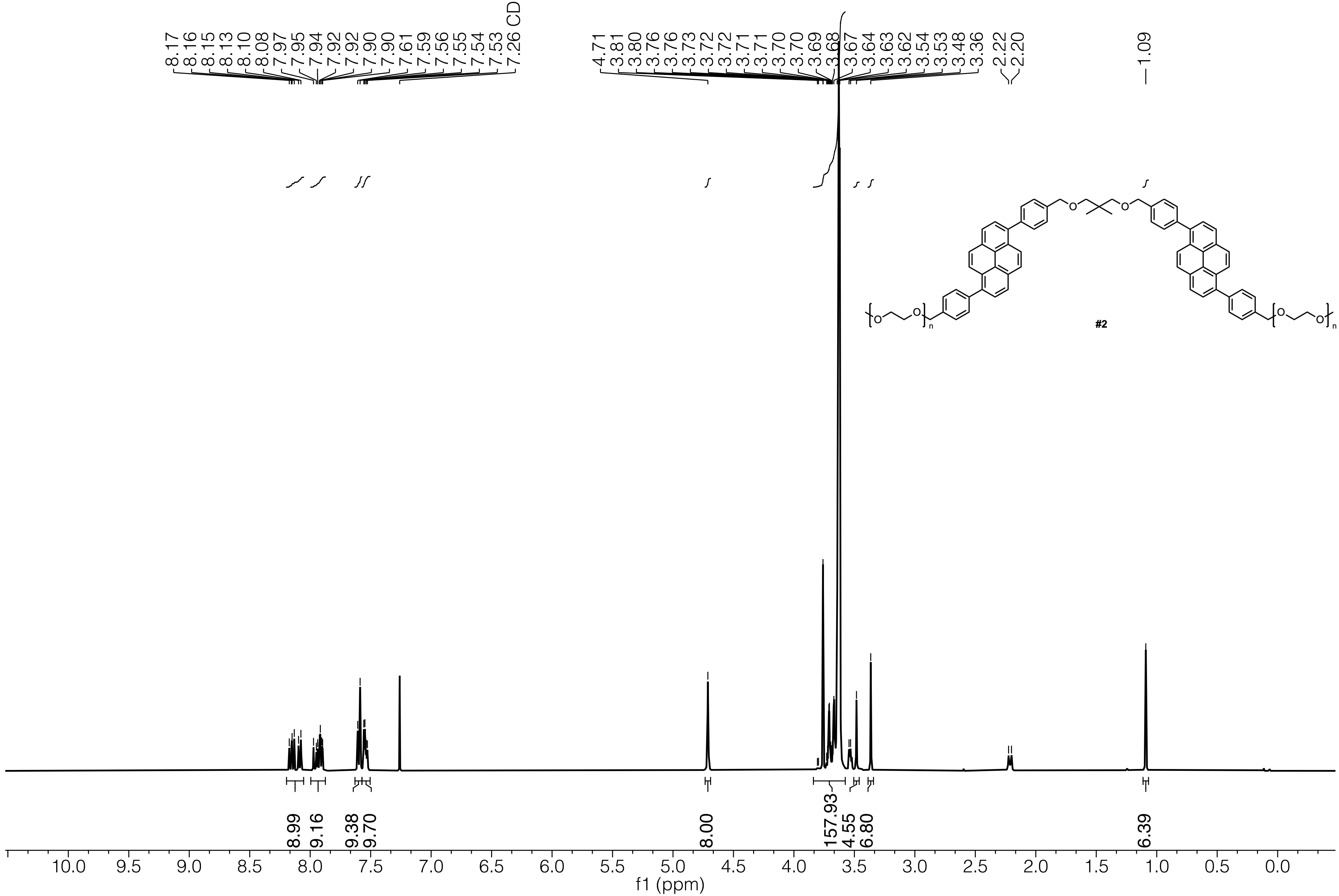
8.17 8.16 8.15 8.13 8.10 8.08 7.97 7.95 7.94 7.92 7.92 7.90 7.90 7.61 7.59 7.56 7.55 7.54 7.53 7.26 CDCl3
4.71 3.81 3.80 3.76 3.76 3.73 3.72 3.72 3.71 3.71 3.70 3.70 3.69 3.68 3.67 3.64 3.63 3.62 3.54 3.53 3.48 3.36 2.22 2.20
1.09
8.99 9.16 9.38 9.70 8.00 157.93 4.55 6.80 6.39
#2
O
n
10.0 9.5 9.0 8.5 8.0 7.5 7.0 6.5 6.0 5.5 5.0 4.5 4.0 3.5 3.0 2.5 2.0 1.5 1.0 0.5 0.0
f1 (ppm)

KS-4-86-GPC
13C

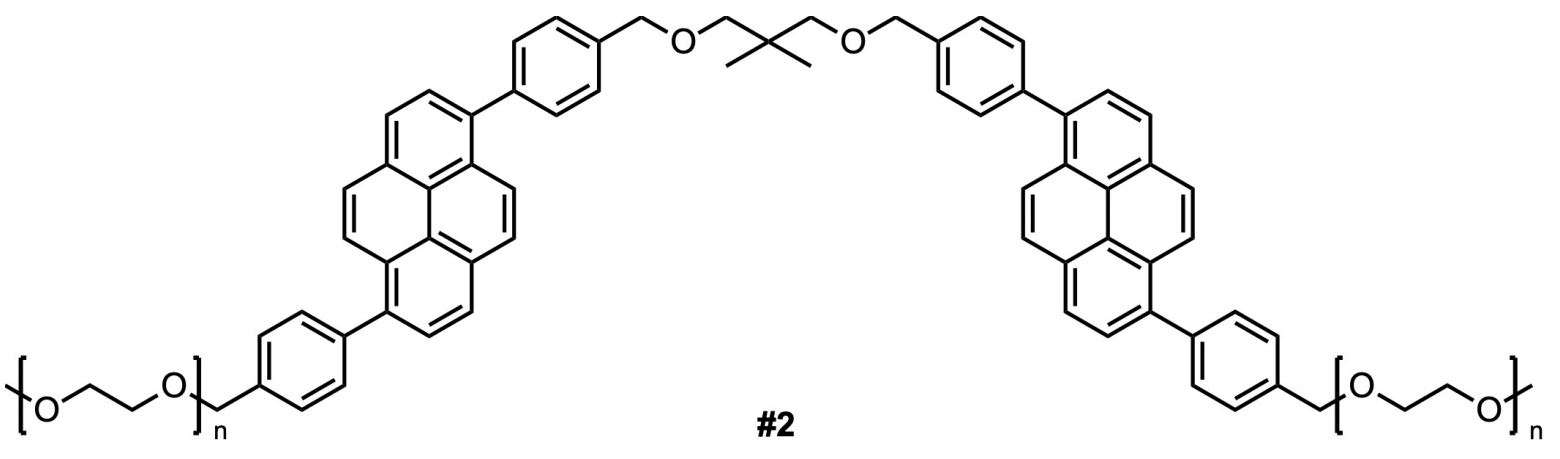

#2

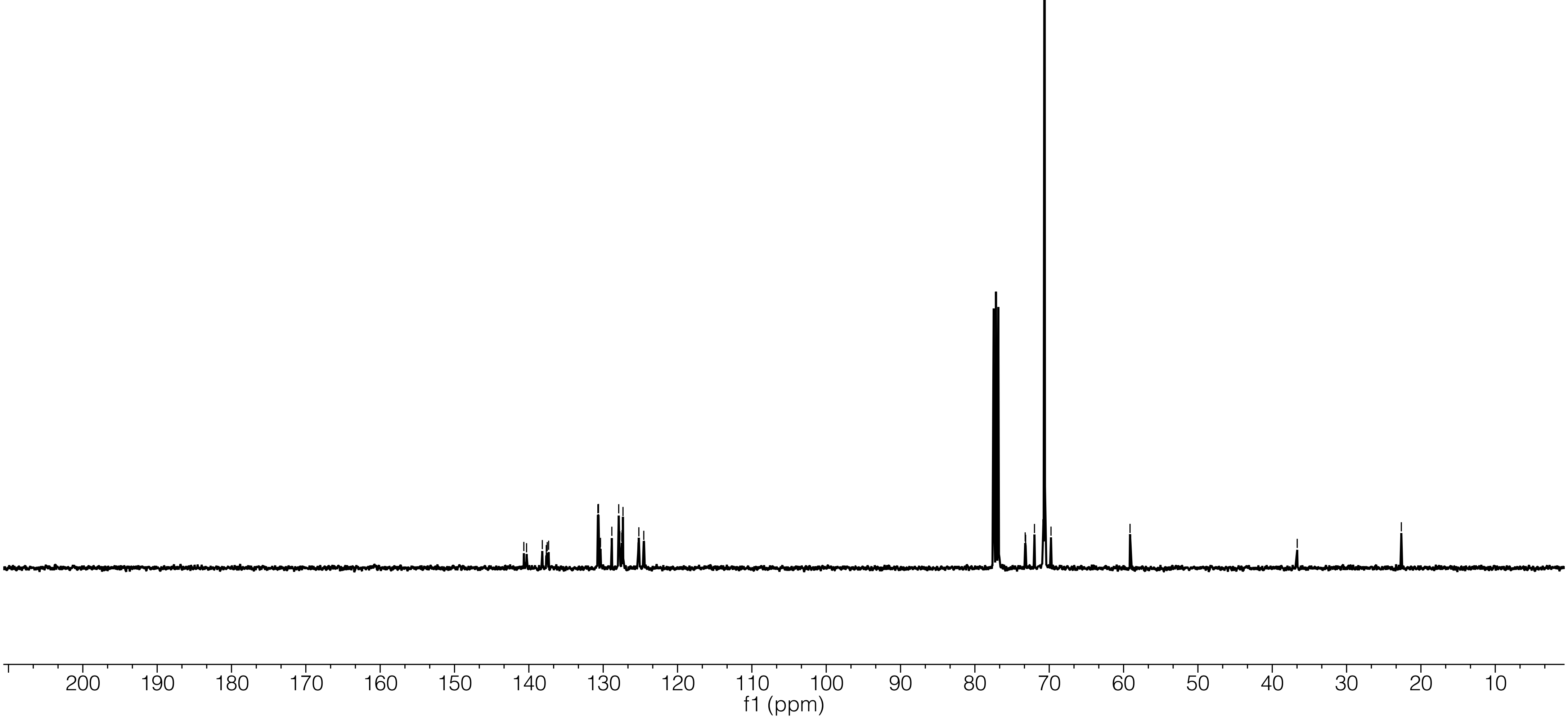

KS-2-20-GPC4
1H

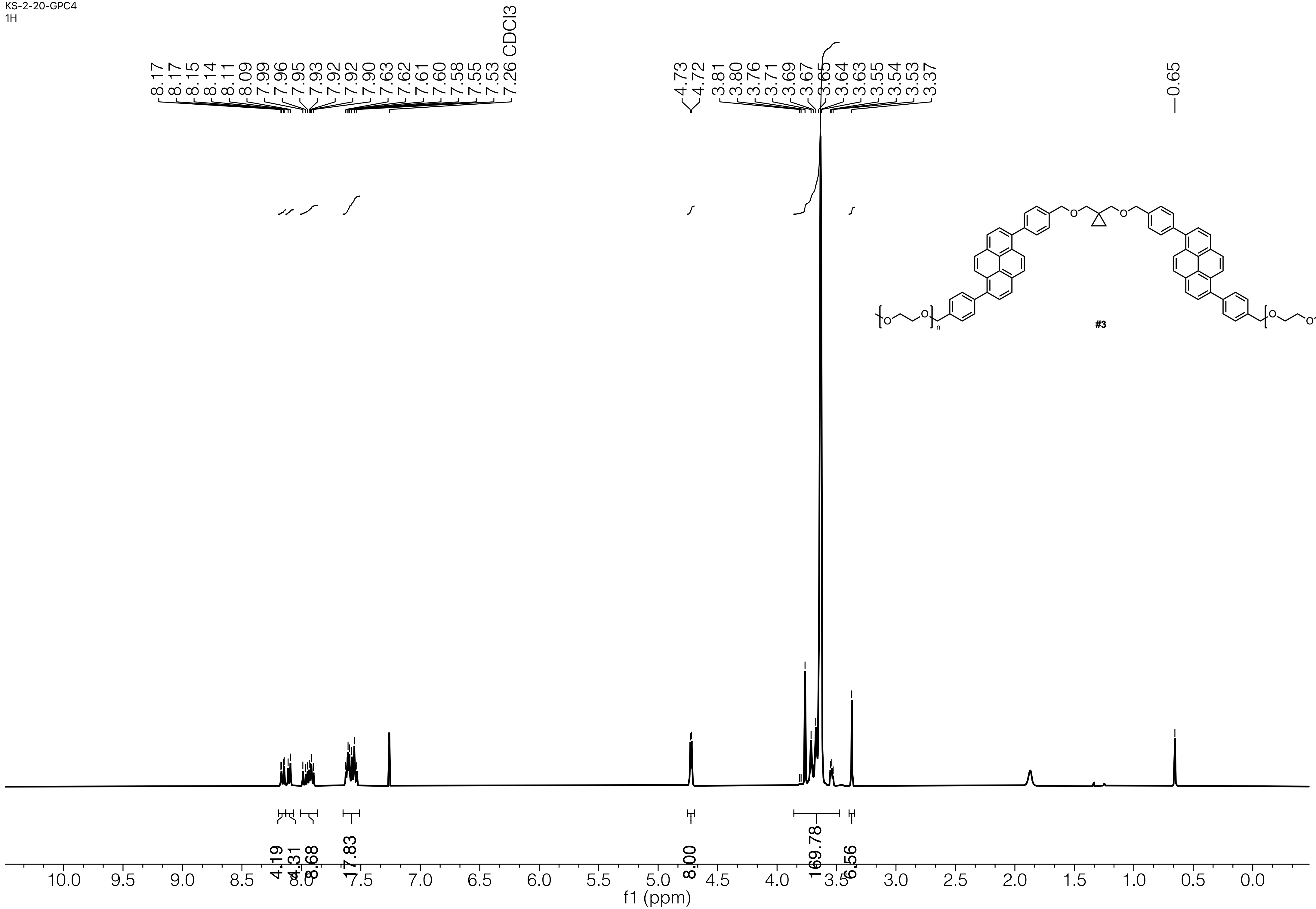

KS-2-20 13C
13C

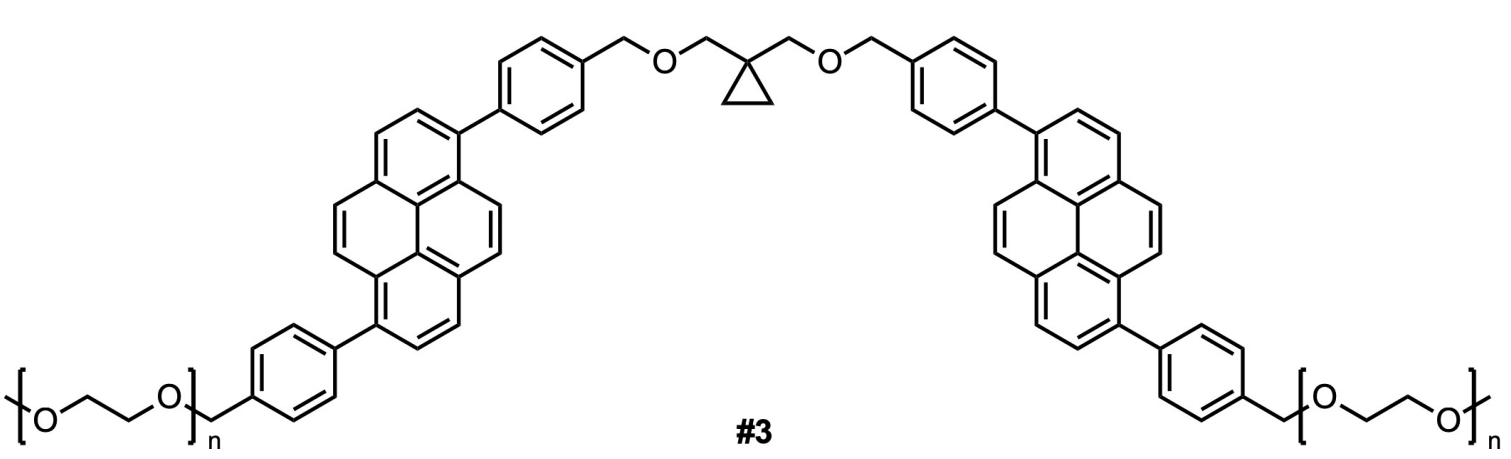

#3

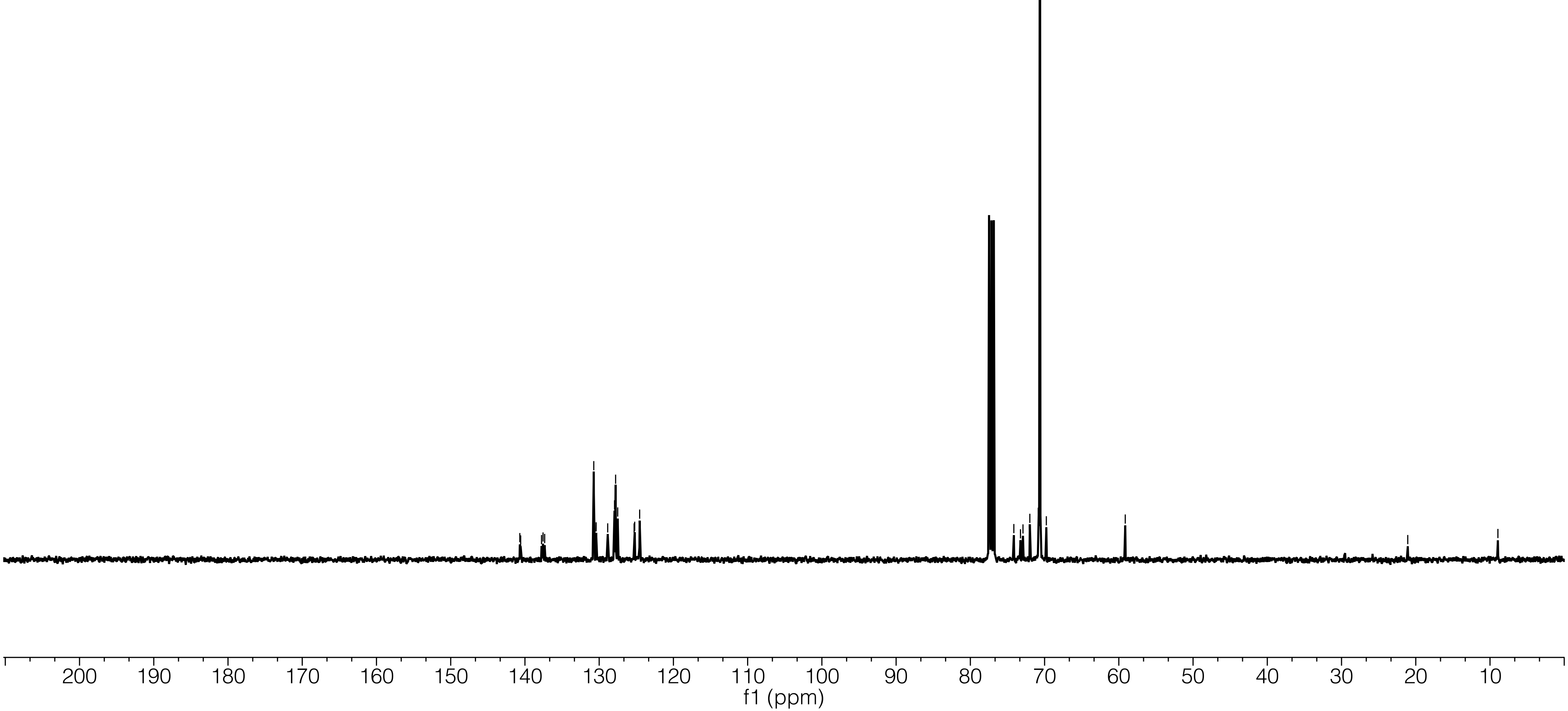

KS-5-5-GPC3
1H

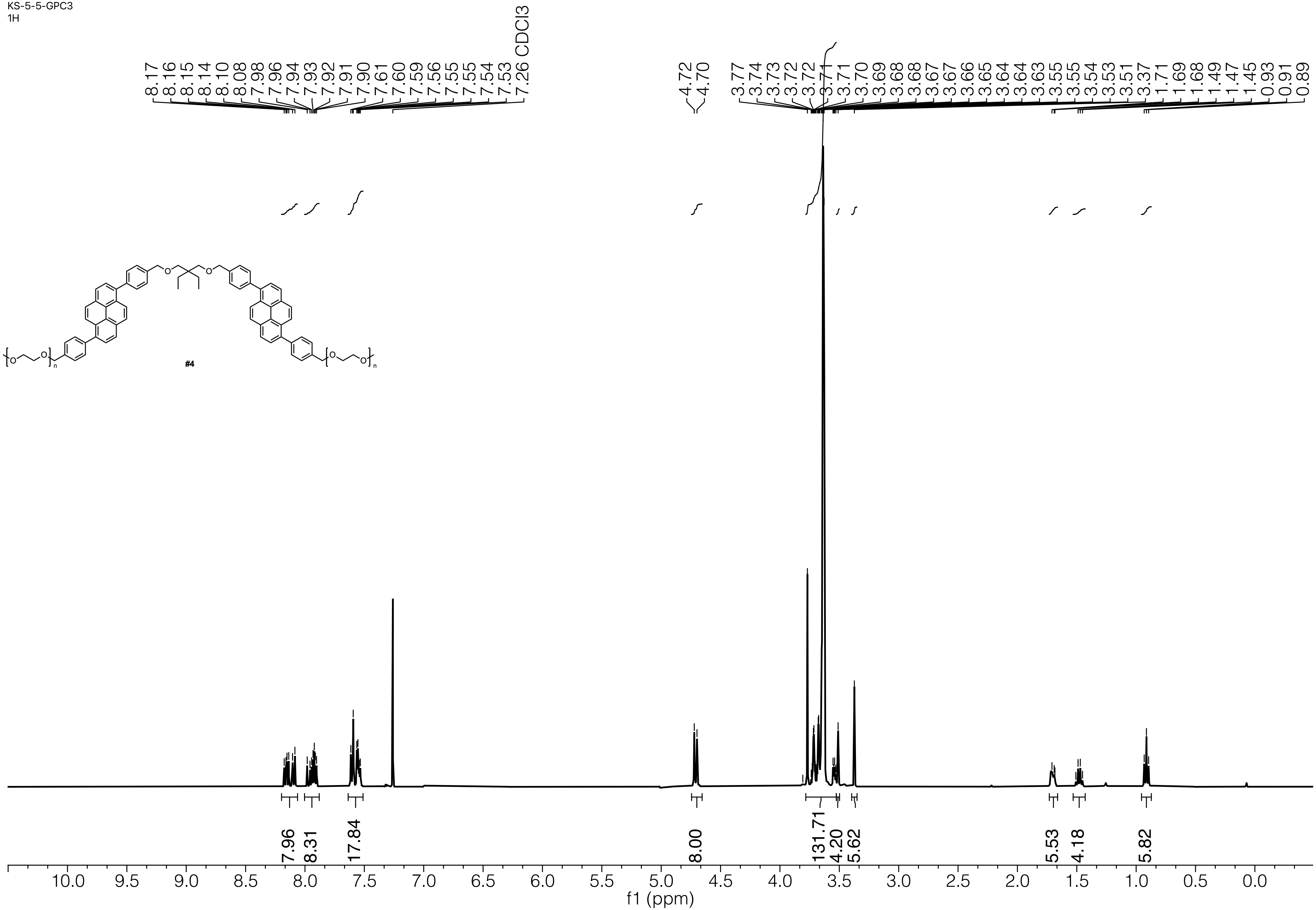

8.17 8.16 8.15 8.14 8.10 8.08 7.98 7.96 7.94 7.93 7.92 7.91 7.90 7.61 7.60 7.59 7.56 7.55 7.55 7.54 7.53 7.26 CDCl3
4.72 4.70
3.77 3.74 3.73 3.72 3.72 3.71 3.71 3.70 3.69 3.68 3.68 3.67 3.67 3.66 3.65 3.64 3.64 3.63 3.55 3.55 3.54 3.53 3.51 3.37 1.71 1.69 1.68 1.49 1.47 1.45 0.93 0.91 0.89
#4
7.96 8.31 17.84 8.00 131.71 4.20 5.62 5.53 4.18 5.82
10.0 9.5 9.0 8.5 8.0 7.5 7.0 6.5 6.0 5.5 5.0 4.5 4.0 3.5 3.0 2.5 2.0 1.5 1.0 0.5 0.0
f1 (ppm)

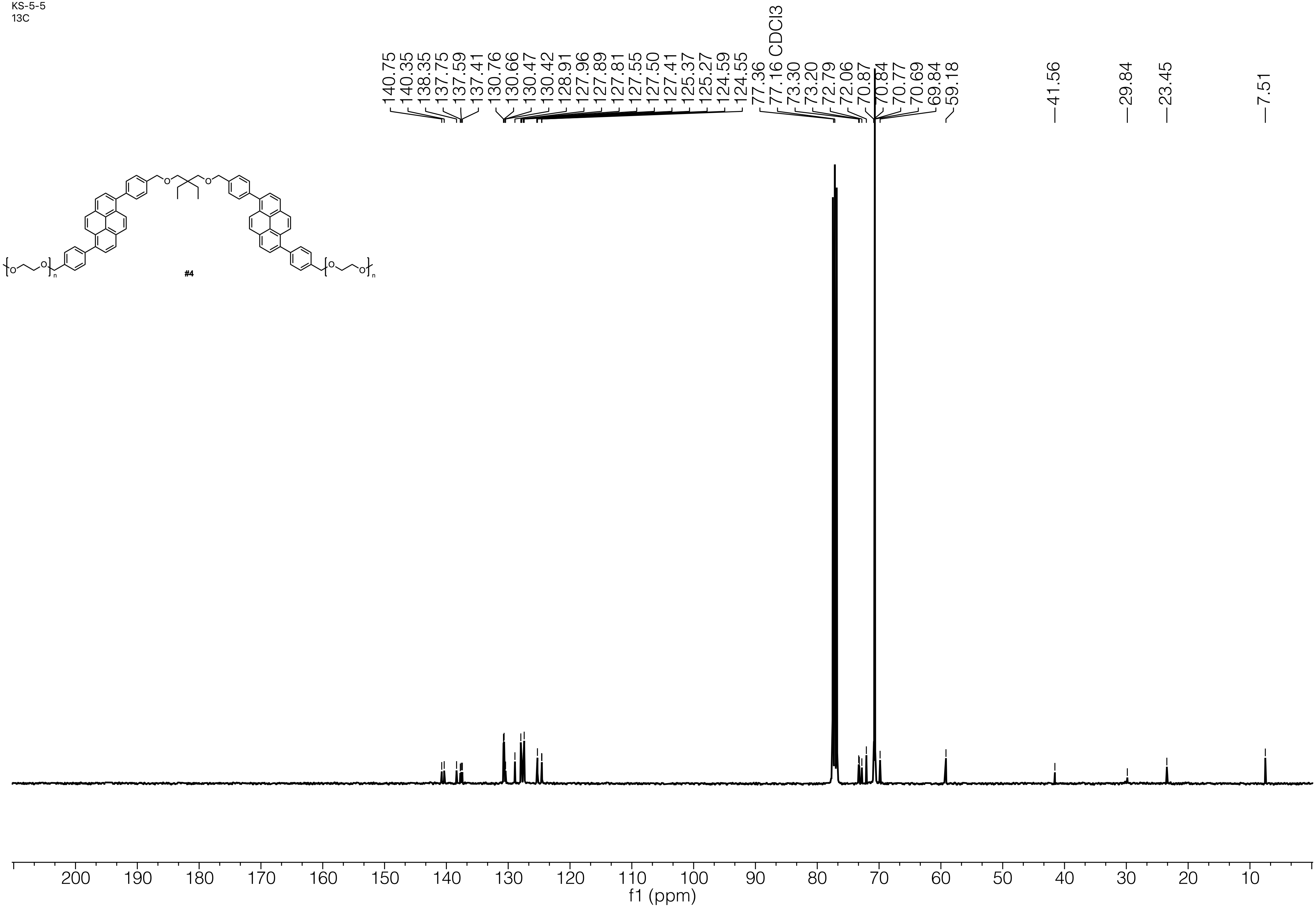
KS-5-5
13C
140.75
140.35
138.35
137.75
137.59
137.41
130.76
130.66
130.47
130.42
128.91
127.96
127.89
127.81
127.55
127.50
127.41
125.37
125.27
124.59
124.55
77.36
77.16 CDCl3
73.30
73.20
72.79
72.06
70.87
70.84
70.77
70.69
69.84
59.18
41.56
29.84
23.45
7.51
#4
200
190
180
170
160
150
140
130
120
110
100
90
80
70
60
50
40
30
20
10
f1 (ppm)

KS-5-15-GPC3
1H

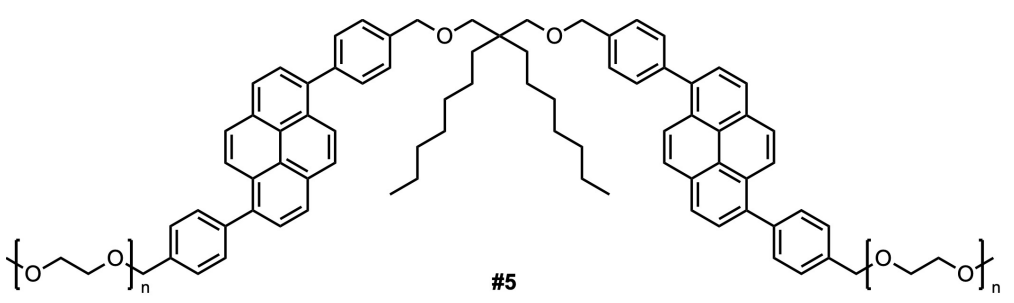

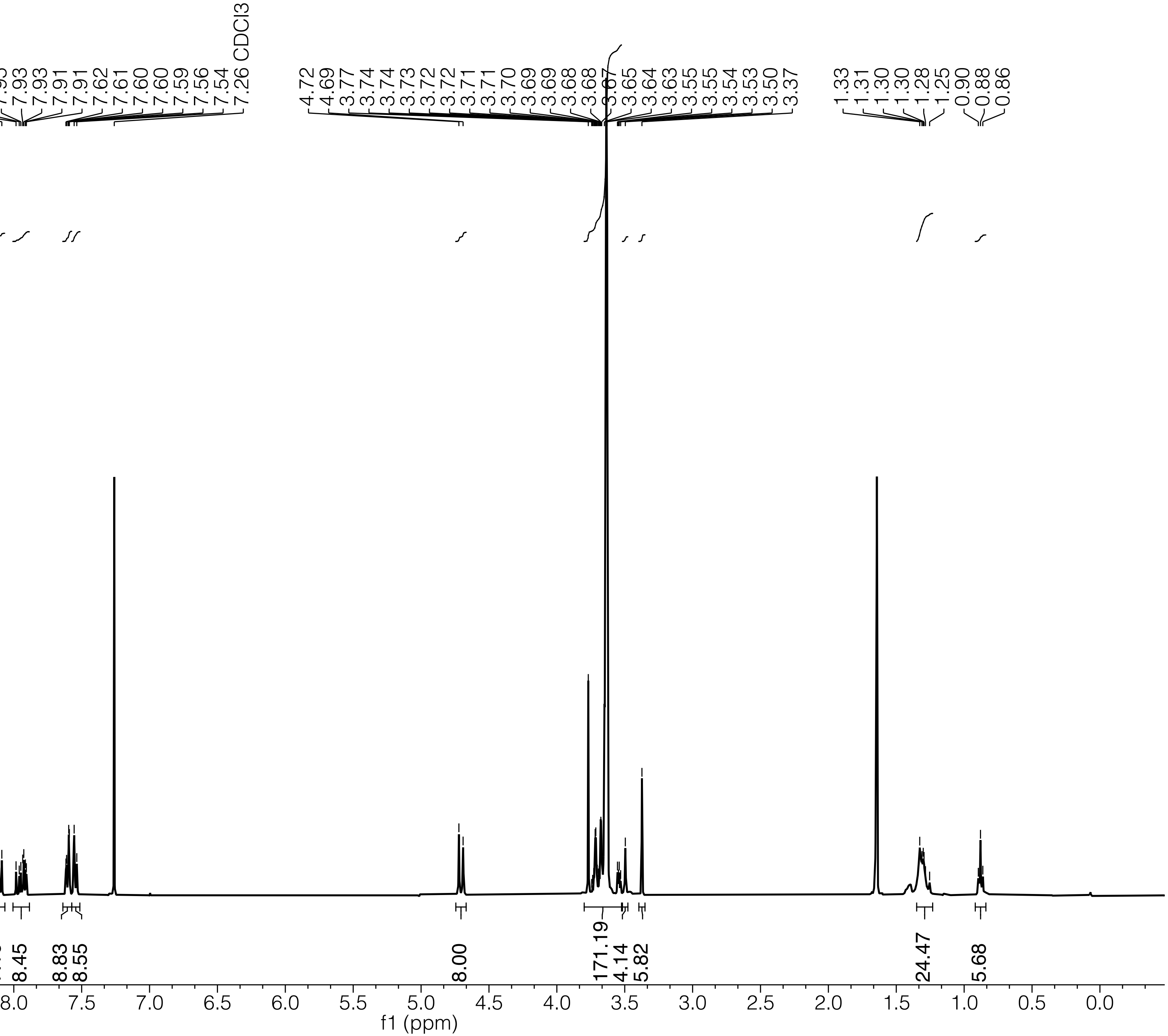

KS-5-5
13C

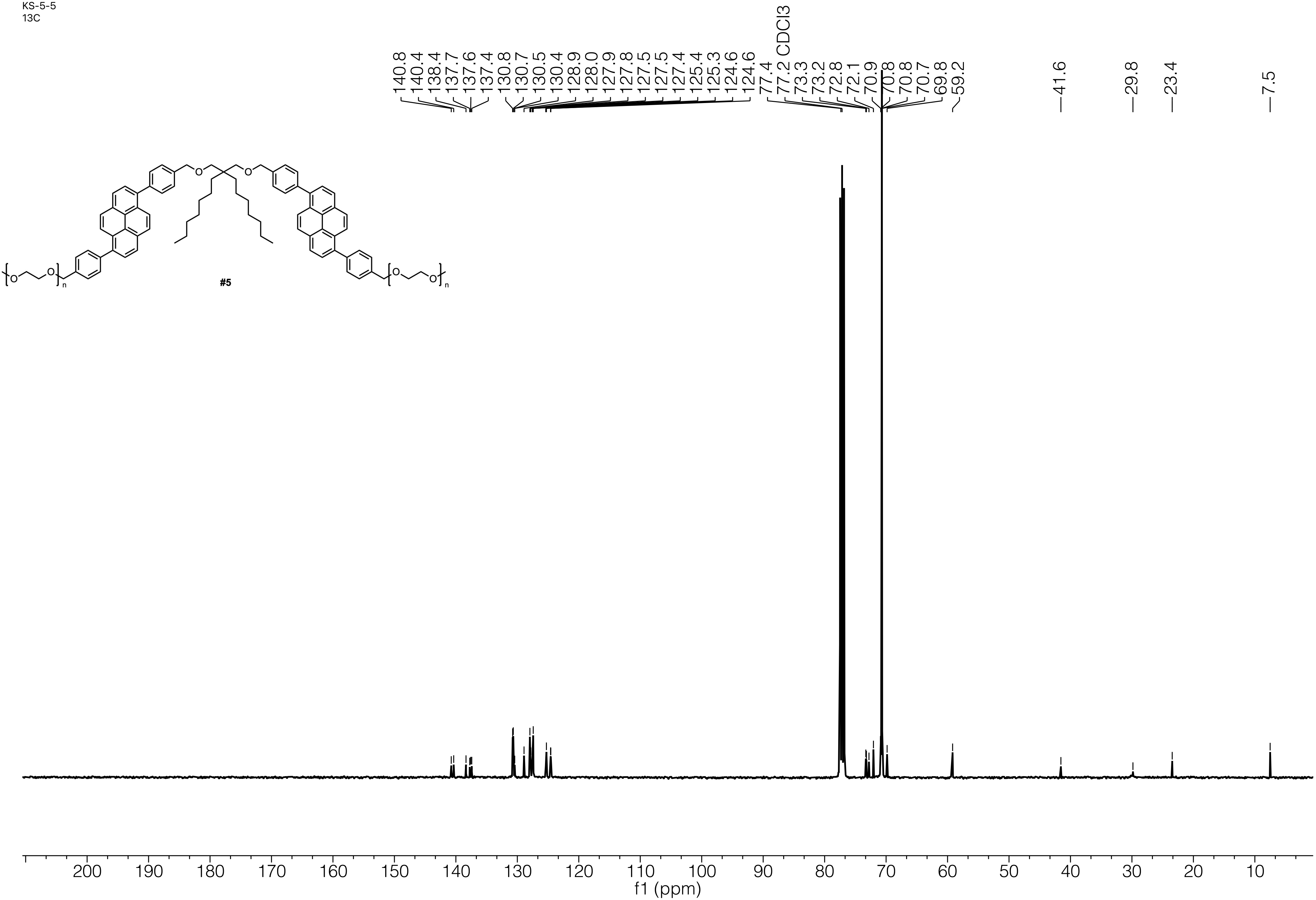

140.8
140.4
138.4
137.7
137.6
137.4
130.8
130.7
130.5
130.4
128.9
128.0
127.9
127.8
127.5
127.5
127.4
125.4
125.3
124.6
124.6
77.4
77.2 CDCl3
73.3
73.2
72.8
72.1
70.9
70.8
70.8
70.7
69.8
59.2
41.6
29.8
23.4
7.5
200 190 180 170 160 150 140 130 120 110 100 90 80 70 60 50 40 30 20 10
f1 (ppm)

#5

KS-5-5-GPC3
1H

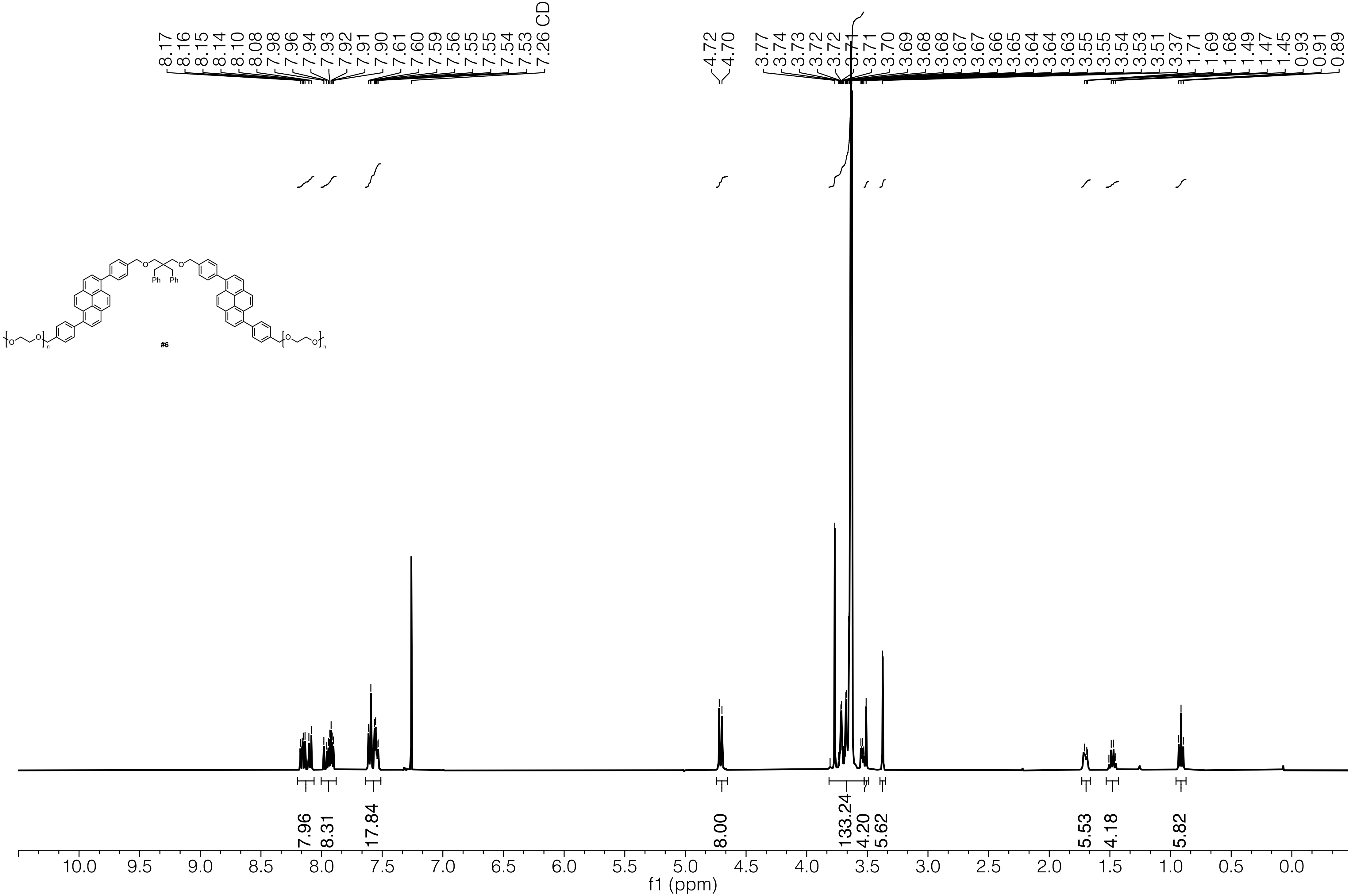
8.17 8.16 8.15 8.14 8.10 8.08 7.98 7.96 7.94 7.93 7.92 7.91 7.90 7.61 7.60 7.59 7.56 7.55 7.55 7.54 7.53 7.26 CDCl3
4.72 4.70
3.77 3.74 3.73 3.72 3.72 3.71 3.71 3.70 3.69 3.68 3.68 3.67 3.67 3.66 3.65 3.64 3.64 3.63 3.55 3.55 3.54 3.53 3.51 3.37 1.71 1.69 1.68 1.49 1.47 1.45 0.93 0.91 0.89
Ph
Ph
O
n
#6
7.96
8.31
17.84
8.00
133.24
4.20
5.62
5.53
4.18
5.82
10.0 9.5 9.0 8.5 8.0 7.5 7.0 6.5 6.0 5.5 5.0 4.5 4.0 3.5 3.0 2.5 2.0 1.5 1.0 0.5 0.0
f1 (ppm)

KS-5-15
13C

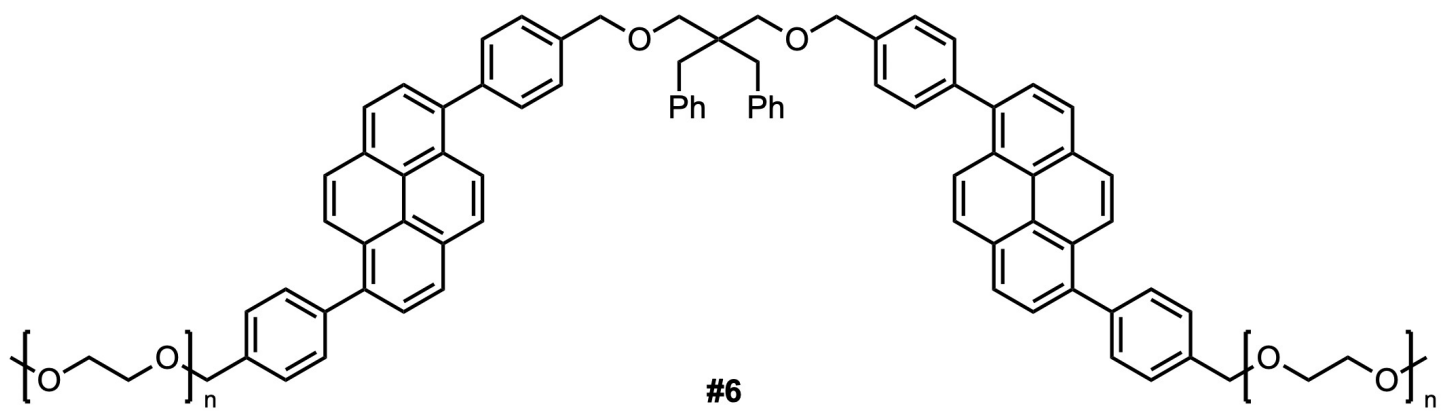

Ph
Ph
O
O
n

#6

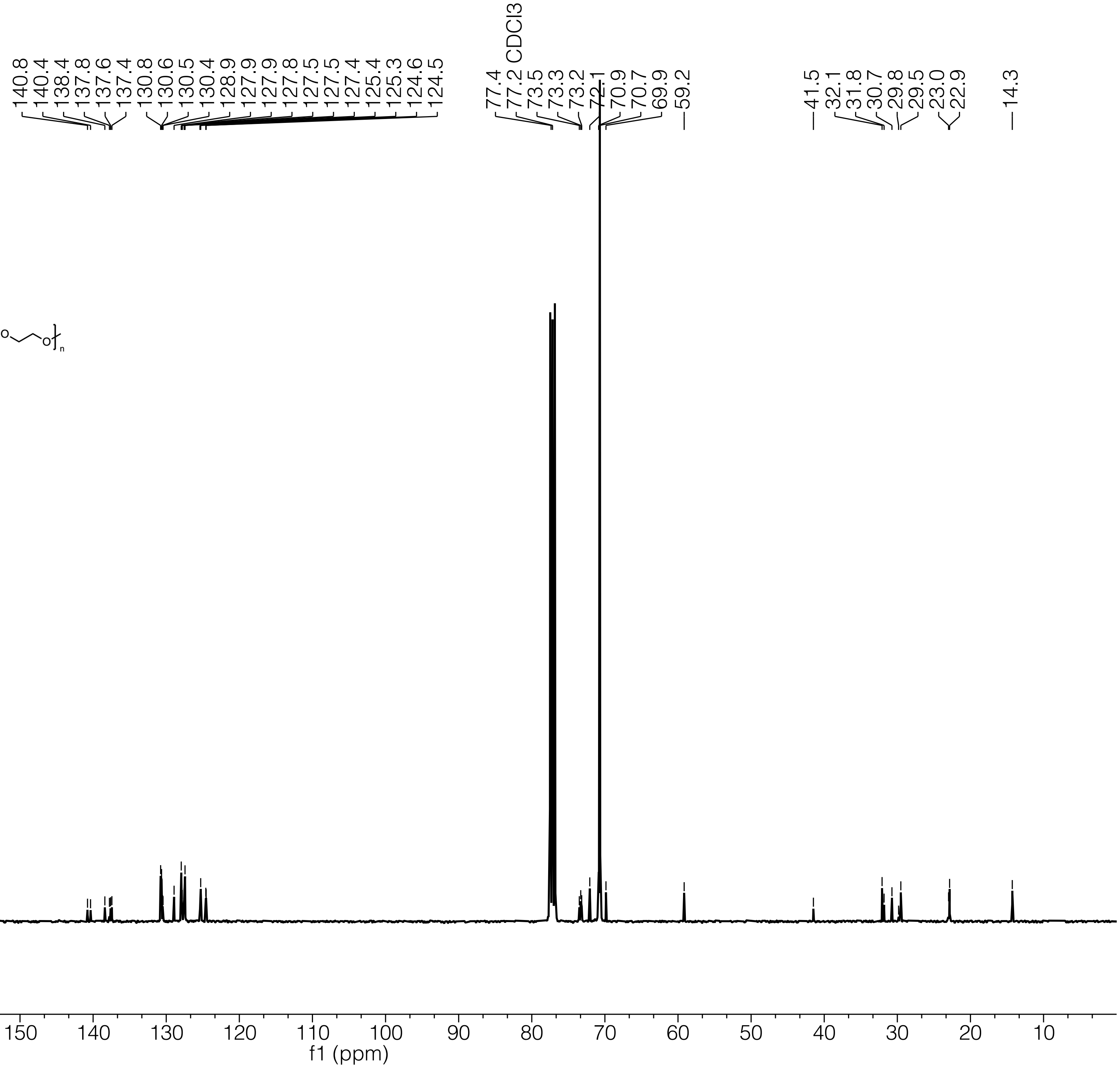

140.8
140.4
138.4
137.8
137.6
137.4
130.8
130.6
130.5
130.4
128.9
127.9
127.9
127.8
127.5
127.5
127.4
125.4
125.3
124.6
124.5
77.4
77.2 CDCl3
73.5
73.3
73.2
72.1
70.9
70.7
69.9
59.2
41.5
32.1
31.8
30.7
29.8
29.5
23.0
22.9
14.3
200
190
180
170
160
150
140
130
120
110
100
90
80
70
60
50
40
30
20
10
f1 (ppm)

260512-PyM-1H-CDCl3-2-ok.1.fid

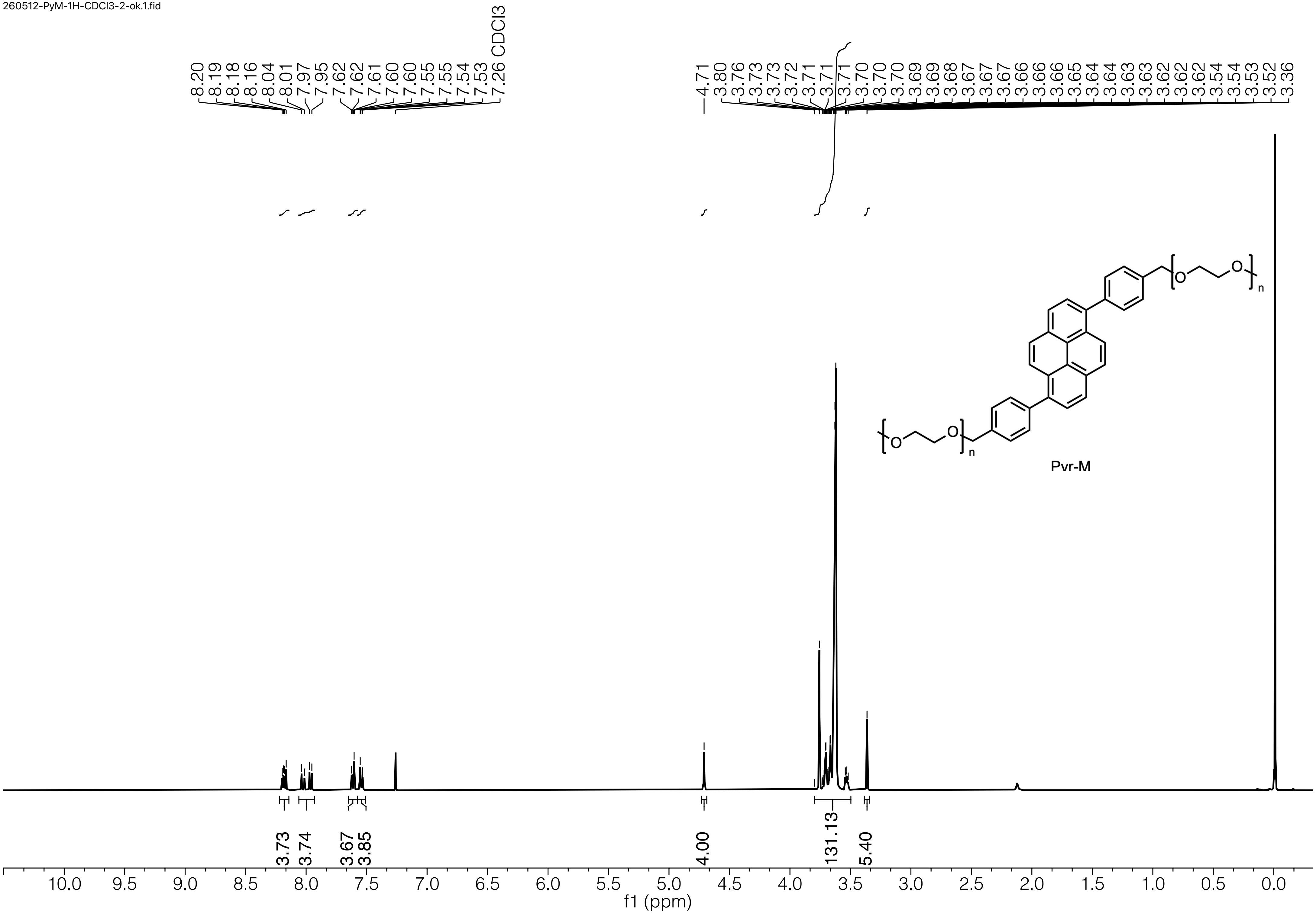

20260512-Pyr-M-13C-CDCl3-ok 3.1.fid

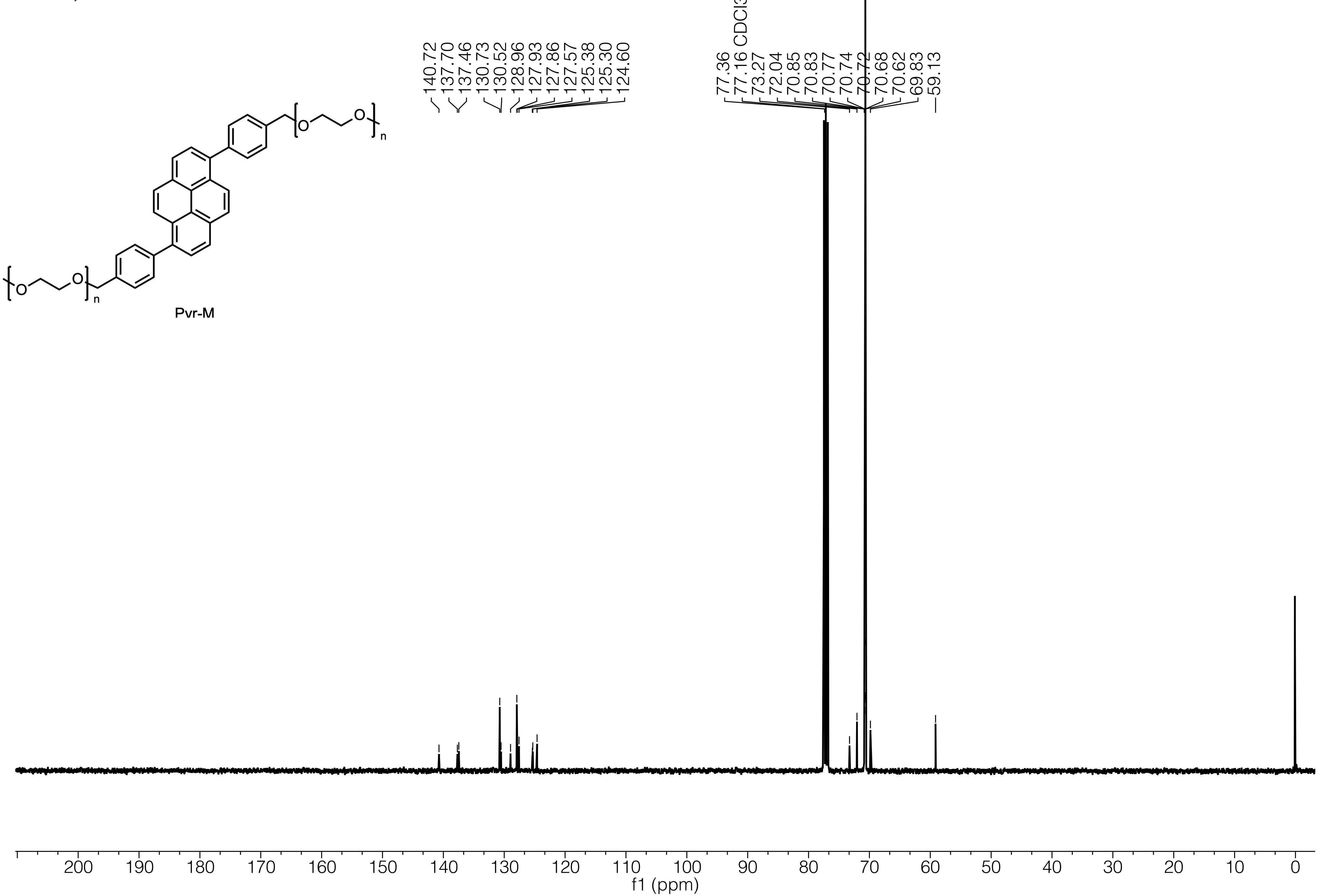

Supplementary Figure S2. NMR spectra of synthesized compounds.

^1H, ^13C, and, where applicable, ^11B NMR spectra of the synthesized dibromo precursors **1a–6a**, bis(pinacol boronate) derivatives **1b–6b**, polymers **#1–#6**, and **Pyr-M**. Spectra were recorded in CDCl3 unless otherwise noted; the ^1H NMR spectrum of **6a** was recorded in acetone-d6. Residual solvent peaks are indicated in the spectra.
