# Supplementary material for: A Bright Ratiometric Dipyrene Probe for Functional Imaging of Condensate Microenvironments in Living Cells
Source: Adv Sci (Weinh). 2026 Jul 16:e76419. Online ahead of print. doi: 10.1002/advs.76419 (PMC13374536; doi:10.1002/advs.76419)
Supplement: Supplementary file 1 — Supporting File 1: advs76419‐sup‐0001‐SuppMat.docx. [file ADVS-9999-e76419-s002.docx]

A Bright Ratiometric Dipyrene Probe for Functional Imaging of Condensate Microenvironments in Living Cells

Koki Matsumoto‡^1^ , Sora Kitai‡^2^ ,Yoshio Nishiyama^2^ ,Shogo Amemori^2-4^, Kei Makiyama^1^, Dini Kurnia Ikliptikawati^3^, Kentaro Ohira^5^, Yuta Kozuka^6^, Maho Tobita^6^, Shih-Cheng Chen^7,8^, Chien-Hung Yu^8,9^, Wei-Min Liu^10^, De-Chen Lin^11, 12^, Motohiro Mizuno^2-4^, Kazuma Ogawa^13^, Koichi Ogami^14^, Kenji Takahashi^6^, Hiroshi I Suzuki^14-17^, Richard W. Wong*^3,4,18^, Takahiro Soeta*^2^, Masaharu Hazawa*^3,4,18^

1. Division of Transdisciplinary Sciences, Graduate School of Frontier Science Initiative, Kanazawa University, Kakuma-machi, Kanazawa, Ishikawa 920-1192, Japan.
2. Division of Material Sciences, Graduate School of Natural Science and Technology, Kanazawa University, Kakuma, Kanazawa 920-1192, Japan.
3. Institute for Frontier Science Initiative, Kanazawa University, Kakuma-machi, Kanazawa, Ishikawa 920-1192, Japan.
4. NanoMaterials Research Institute, Kanazawa University, Kanazawa 920-1192, Japan.
5. School of Chemistry, College of Science and Engineering, Kanazawa
6. University, Kakuma, Kanazawa, 920-1192, Japan
7. Division of Biological Science and Technology, Graduate School of Natural Science and Technology
8. National Institute of Cancer Research, National Health Research Institutes, Tainan City, Taiwan.
9. Department of Biochemistry and Molecular Biology, College of Medicine, National Cheng Kung University, Tainan City, Taiwan.
10. Institute of Basic Medical Sciences, College of Medicine, National Cheng Kung University, Tainan City, Taiwan.
11. Department of Chemistry, Fu Jen Catholic University, New Taipei City, Taiwan.
12. Center for Craniofacial Molecular Biology, Herman Ostrow School of Dentistry, University of Southern California, Los Angeles, CA, USA
13. Norris Comprehensive Cancer Center, University of Southern California, Los Angeles, CA, USA.
14. Graduate School of Medical Sciences, Kanazawa University, Kakuma-machi, Kanazawa, Ishikawa 920-1192, Japan.
15. Division of Molecular Oncology, Center for Neurological Diseases and Cancer, Nagoya University Graduate School of Medicine, 65 Tsurumai-cho, Showa-ku, Nagoya, Aichi 466-8550, Japan.
16. Institute for Glyco-Core Research (iGCORE), Nagoya University, 65 Tsurumai-cho, Showa-ku, Nagoya, Aichi 464-8601, Japan.
17. Center for One Medicine Innovative Translational Research (COMIT), Nagoya University, 65 Tsurumai-cho, Showa-ku, Nagoya, Aichi 464-8601, Japan.
18. Inamori Research Institute for Science (InaRIS); Kyoto, 600-8411, Japan
19. WPI Nano Life Science Institute, Kanazawa University, Kanazawa, Ishikawa 920-1192, Japan.

‡These authors contributed equally, *Corresponding authors.

This file includes Figure S1, and S3-S12

***Experimental section***

**Synthetic procedures and characterization**

**General**

^1^H NMR was recorded on a JEOL ECS 400 (399.78 MHz) and Bruker Avance neo 400 (400.23 MHz) NMR spectrometer. Chemical shifts are reported in ppm using TMS as an internal standard. Data are reported as follows: Chemical shift, multiplicity (s = singlet, d = doublet, t = triplet, q = quartet, m = multiplet), coupling constant (*J*) and integration. ^13^C NMR spectra were recorded on JEOL ECS 400　(100.52 MHz)　and Bruker Avance neo 400 (100.63 MHz) NMR spectrometer. The chemical shifts were determined in the d-scale relative to CDCl3 (δ = 77.16 ppm). The IR spectra were measured on JASCO FT/IR-230 spectrometers. The MS spectrum was recorded with JEOL SX-102A mass spectrometer, JMS-T100TD, and Bruker microtof II. Dehydrated solvents were purchased for the reactions and used without further desiccation. Flash column chromatography was performed by using silica gel (PSQ60B, Fuji Silysia). All reactions were carried out under an argon atmosphere unless otherwise noted. Thin-layer chromatography (TLC) was performed on silica gel 60 F254 plates and visualized under UV light. Some carbon signals corresponding to PEG chains were broadened or overlapped and could not be fully assigned.

**General procedure for the synthesis of (4-(6-bromopyren-1-yl)phenyl)methanol**^1^ **(7) (4.0 mmol scale)**


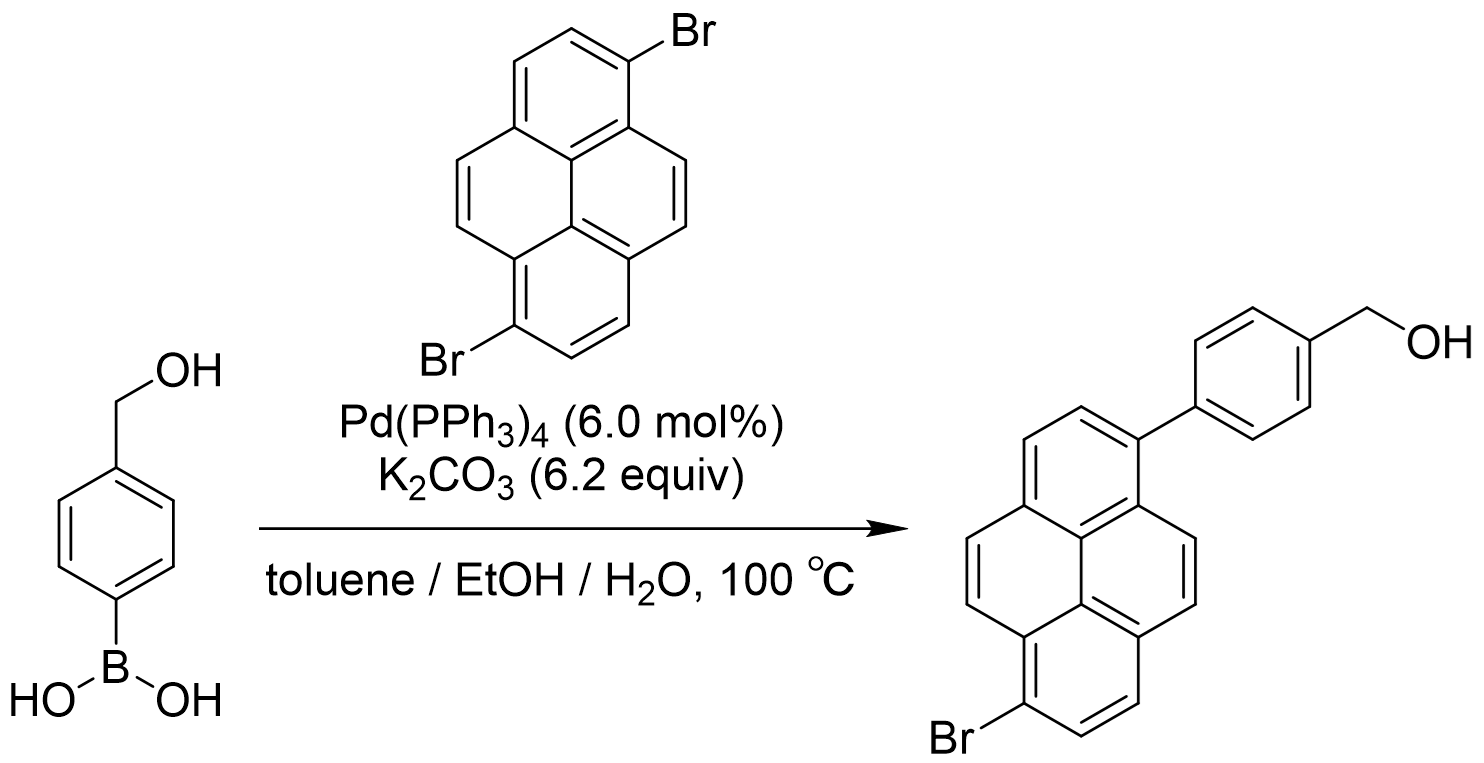


Under Ar atmosphere, the mixture of 4-(hydroxymethyl)phenylbronicacid (608 mg, 4.0 mmol), 1,6-dibromopyrene (1.73 g, 5.0 mmol, 1.2 equiv), and potassium carbonate (3.59 g, 26.0 mmol, 6.2 equiv in 10 mL water), in 80 mL of toluene and 10 mL of EtOH was added to Pd(PPh_3_)_4_ (300.5 mg, 6.0 mol%). The mixture was stirred at 100^◦^C (oil bath) and the reaction was monitored by TLC. After the reaction was completed, the reaction mixture was cooled to room temperature and concentration. The residue was dissolved in CH_2_Cl_2_ and filtered. Then CH_2_Cl_2_ filtrate was washed by brine, dried over Na_2_SO_4_, and concentration. The crude mixture was purified by silica gel flash chromatography (CHCl_3_). The solid product was crystallized from CHCl_3_ and hexane to give **7** (704.5 mg, 45% yield) as a white solid. mp 172-174^◦^C. ^1^H NMR (400 MHz, CDCl_3_) δ 8.48 (d, *J* = 9.2 Hz, 1H) 8.27 (d, *J* = 7.9 Hz ,1H), 8.25 (d, *J* = 8.3 Hz ,1H), 8.21 (d, *J* = 9.2 Hz ,1H), 8.18 (d, *J* = 9.3 Hz ,1H), 8.01 (d, *J* = 7.9 Hz ,1H), 8.00 (d, *J* = 8.3 Hz ,1H), 7.98 (d, *J* = 9.3 Hz ,1H), 7.63 (d, *J* = 8.3 Hz ,2H), 7.58 (d, *J* = 8.3 Hz ,2H), 4.86 (t, *J* = 5.9 Hz, 2H), 1.77 (t, *J* = 5.9 Hz ,1H). ^13^C NMR (101 MHz, CDCl_3_) δ 140.4, 140.0, 138.2, 130.8, 130.4, 130.3, 130.0 129.0, 128.6, 128.2, 127.2, 127.1, 126.1, 126.0, 125.5, 125.4, 125.2, 124.4, 120.0, 65.2. IR (neat): 3270, 3048, 2904, 2855, 1601, 1579, 1479, 1455, 1431, 1412, 1366, 1322, 1309, 1277, 1234, 1211, 1195, 1180, 1161, 1132, 1107, 1041, 1025, 1003, 969, 946, 871, 856, 837, 817, 792, 757, 713, 678 cm^–1^. HRMS-FAB (m/z): Calcd for C_23_H_15_BrO [M]^+^: 386.0306. found: 386.0311.

The data are in agreement with those reported in the literature^1^.

**-General procedure for the synthesis of bis 4-bromobenzyl derivatives**


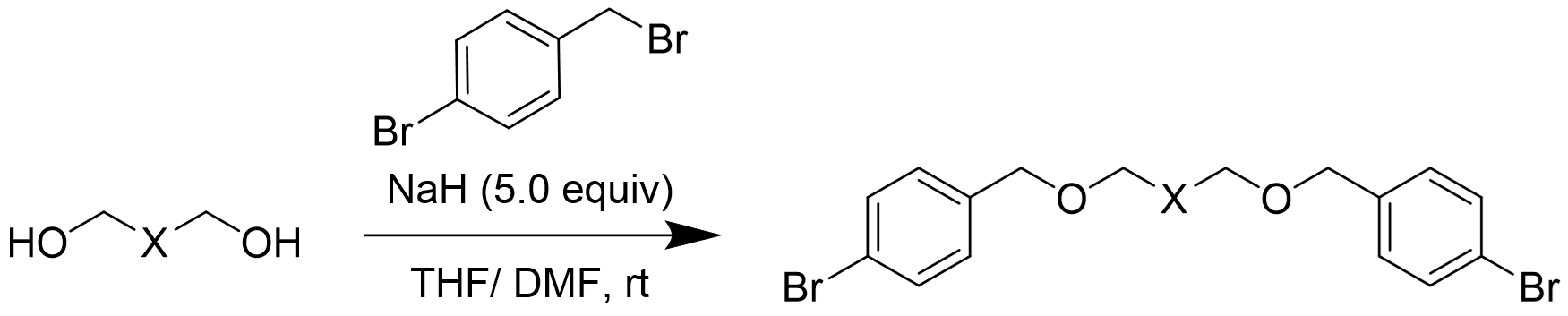


Under Ar atmosphere, to a stirring solution of diol (1.0 equiv) in THF and DMF was added NaH (60% dispersion in oil, 5.0 equiv) at 0^◦^C. After 1 hour, 4-bromobenzyl bromide (3.0 equiv) in THF was slowly added. The resulting mixture was warmed up to room temperature, and the reaction was monitored by TLC. After the reaction was completed, the reaction mixture was cooled to 0^◦^C and poured into water. The aqueous layer was separated and extracted with a mixture of hexane and ethyl acetate (3:1). The combined organic layers were washed with brine and dried over Na_2_SO_4_. Concentration and purified by silica gel column chromatography to give the products.

**1,3-Bis((4-bromobenzyl)oxy)propane**^2^ **(1a)**

Silica gel column chromatography (hexane /ethyl acetate = 20/1) gave **1a** (825 mg, 99% yield, 2 mmol scale) as colorless oil. ^1^H NMR (400 MHz, CDCl_3_) δ 7.45 (d, *J* = 8.2 Hz, 4H), 7.17 (d, *J* = 8.2 Hz, 4H), 4.43 (s, 4H), 3.55 (t, *J* = 6.4 Hz, 4H), 1.94-1.87 (m, 2H). ^13^C NMR (101 MHz, CDCl_3_) δ 137.6, 131.6, 129.3 121.5, 72.3, 67.4, 30.2. IR (neat): 2922, 2859, 1592, 1489, 1395, 1360, 1093, 1070, 1040, 1011, 836, 802, cm^–1^. HRMS-FAB (m/z): Calcd for C_17_H_19_Br_2_O_2_ 414.9726. Found: 414.9737.

The data are in agreement with those reported in the literature^2^.

**4,4'-(((2,2-Dimethylpropane-1,3-diyl)bis(oxy))bis(methylene))bis(bromobenzene) (2a)**

Silica gel column chromatography (hexane /ethyl acetate = 20/1) gave **2a** (760 mg, 85% yield, 2.0 mmol scale) as colorless oil. ^1^H NMR (400 MHz, CDCl_3_) δ 7.43 (d, *J* = 8.2 Hz, 4H), 7.16 (d, *J* = 8.7 Hz, 4H), 4.42 (s, 4H), 3.23 (s, 4H), 0.92 (s, 6H). ^13^C NMR (101 MHz, CDCl_3_) δ 138.0, 131.5, 129.0, 121.2, 76.4, 72.5, 36.4, 22.4. IR (neat): 2957, 2854, 1894, 1592, 1486, 1456, 1398, 1374, 1356, 1297, 1282, 1243, 1201, 1092, 1070, 1011, 963, 933, 914, 830, 803, 757, 644, 629 cm^–1^. HRMS-FAB (m/z): Calcd for C_19_H_23_Br_2_O_2_ [M+H]^+^: 443.0044. Found: 443.0036.

**4,4'-(((Cyclopropane-1,1-diylbis(methylene))bis(oxy))bis(methylene))bis(bromobenzene) (3a)**

Silica gel column chromatography (hexane /ethyl acetate = 20/1) gave **3a**(791 mg, 89% yield, 2 mmol scale) as colorless oil. ^1^H NMR (400 MHz, CDCl_3_) δ 7.44 (d, *J* = 8.7 Hz, 4H), 7.16 (d, *J* = 8.2 Hz, 4H), 4.44 (s, 4H), 3.38 (s, 4H), 0.49 (s, 4H). ^13^C NMR (101 MHz, CDCl_3_) δ 137.7, 131.5, 129.3, 121.4, 73.8, 72.2, 20.8, 8.7. IR (neat): 3077, 3004, 2857, 1897, 1593, 1487, 1397, 1361, 1324, 1298, 1276, 1252, 1200, 1174, 1091, 1041, 1011, 964, 932, 907, 838, 803, 794, 756, 733, 644, 629 cm^–1^. HRMS-FAB (m/z): Calcd for C_19_H_21_Br_2_O_2_ [M+H]^+^: 440.9888. Found: 440.9890.

**4,4'-(((2,2-Diethylpropane-1,3-diyl)bis(oxy))bis(methylene))bis(bromobenzene) (4a)**

Silica gel column chromatography (hexane /CHCl_3_ = 19/1) gave **4a** (344 mg, 48% yield, 1.5 mmol scale) as a white solid. mp 97-98^◦^C. ^1^H NMR (400 MHz, CDCl_3_) δ 7.42 (d, *J* = 8.0 Hz, 4H), 7.15 (d, *J* = 8.4 Hz, 4H), 4.39 (s, 4H), 3.24 (s, 4H), 1.29 (q, *J* = 7.5 Hz, 4H), 0.76 (t, *J* = 7.6 Hz, 6H). ^13^C NMR (101 MHz, CDCl_3_) δ 138.1, 131.4, 129.1, 121.2, 72.48, 72.40, 41.2, 23.3, 7.3. IR (neat): 2964, 2932, 2860, 1895, 1724, 1592, 1486, 1461, 1397, 1360, 1385, 1345, 1294, 1274, 1260, 1201, 1174, 1094, 1070, 1011, 957, 875, 835, 804, 764, 629 cm^–1^. HRMS-FAB (m/z): Calcd for C_21_H_27_Br_2_O_2_ [M+H]^+^: 471.0357. Found: 471.0353.

**4,4'-(((2,2-Diheptylpropane-1,3-diyl)bis(oxy))bis(methylene))bis(bromobenzene) (5a)**

Silica gel column chromatography (hexane /CHCl_3_ = 4/1) gave **5a** (213 mg, 17% yield, 2.0 mmol scale) as colorless oil. ^1^H NMR (400 MHz, CDCl_3_) δ 7.42 (d, *J* = 8.0 Hz, 4H), 7.14 (d, *J* = 8.4 Hz, 4H), 4.39 (s, 4H), 3.23 (s, 4H), 1.32-1.12 (m, 24H), 0.87 (t, *J* = 6.8 Hz, 6H). ^13^C NMR (101 MHz, CDCl_3_) δ 138.1, 131.4, 129.1, 121.2, 73.0, 72.4, 41.2, 32.0, 31.5, 30.6, 29.4, 22.8, 14.2. Due to signal overlap and symmetry, the number of observed carbon resonances is fewer than the total number of carbons in the molecular formula. IR (neat): 2928, 2856, 1593, 1487, 1404, 1359, 1092, 1071, 1012, 907, 831, 794, 733, 648 cm^–1^. HRMS-FAB (m/z): Calcd for C_31_H_47_Br_2_O_2_ [M+H]^+^: 611.1922. Found: 611.1930.

**4,4'-(((2,2-Dibenzylpropane-1,3-diyl)bis(oxy))bis(methylene))bis(bromobenzene) (6a)**

Silica gel column chromatography (hexane /CHCl_3_ = 4/1) gave **6a** (721 mg, 81% yield, 1.5 mmol scale) as a white solid. mp 97-98^◦^C. ^1^H NMR (400 MHz, Acetone-*d6*) δ 7.55 (d, *J* = 8.0 Hz, 4H), 7.39 (d, *J* = 8.0 Hz, 4H), 7.23-7.13 (m, 10H), 4.45 (s, 4H), 2.97 (s, 4H), 2.83 (s, 4H). ^13^C NMR (101 MHz, CDCl_3_) δ 138.2, 137.5, 131.6, 130.7, 129.4, 128.0, 126.1, 121.5, 72.3, 70.3, 44.0f, 39.1. IR (KBr): 3083, 3065, 3051, 3025, 2946, 2928, 2875, 2820, 1944, 1891, 1809, 1780, 1764, 1598, 1484, 1455, 1420, 1398, 1366, 1338, 1295, 1280, 1252, 1207, 1158, 1109, 1083, 1041, 1010, 920, 897, 852, 829, 798, 748, 731, 701, 647, 605 cm^–1^. HRMS-FAB (m/z): Calcd for C_31_H_31_Br_2_O_2_ [M+H]^+^: 595.0670. Found: 595.0664.

**General procedure for the synthesis of bis dioxaborolane species**


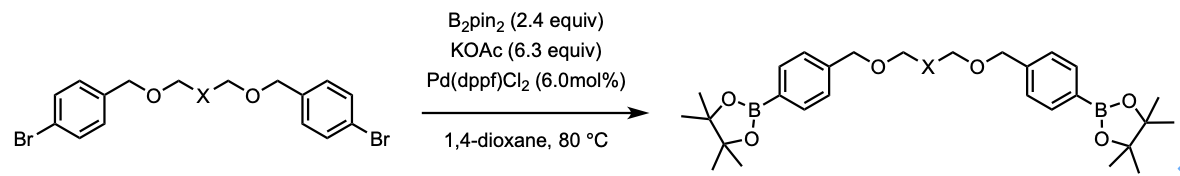


Under Ar atmosphere, the mixture of compound **a** (1.0 equiv) and bis(pinacolato)diboron (2.4 equiv) in 1,4-dioxane was added to potassium acetate (6.3 equiv). The mixture was thoroughly degassed by freeze-pump-thaw before Pd(dppf)Cl_2_ (6.0 mol%) was added. The mixture was stirred at 80^◦^C and the reaction was monitored by TLC. After the reaction was completed, the reaction mixture was filtered off through celite and the filtrate was concentrated under reduced pressure. The residue was purified by silica gel column chromatography.

**1,3-Bis((4-(4,4,5,5-tetramethyl-1,3,2-dioxaborolan-2-yl)benzyl)oxy)propane (1b)**

Silica gel column chromatography (hexane /ethyl acetate = 10/1) gave **1b** (266 mg, 65% yield, 0.8 mmol scale) as colorless oil. ^1^H NMR (400 MHz, CDCl_3_) δ 7.81 (d, *J* = 7.3 Hz, 4H), 7.34 (d, *J* = 7.8 Hz, 4H), 4.51 (s, 4H), 3.58 (t, *J* = 6.2 Hz, 4H), 1.96-1.90 (m, 2H), 1.35 (s, 24H). ^13^C NMR (101, MHz, CDCl_3_) δ 141.9, 135.0, 129.9, 83.8, 73.0, 67.4, 30.3, 25.0. ^11^B NMR (128 MHz, CDCl_3_) δ 30.1. IR (neat): 2978, 2929, 2860, 1614, 1517, 1468, 1399, 1360, 1321, 1272, 1214, 1145, 1089, 1021, 962, 859, 822, 756, 730, 658 cm^–1^. HRMS-FAB (m/z): Calcd for C_29_H_43_B_2_O_6_ [M+H]^+^: 509.3245. Found: 509.3242.

**2,2'-((((2,2-Dimethylpropane-1,3-diyl)bis(oxy))bis(methylene))bis(4,1-phenylene))bis(4,4,5,5-tetramethyl-1,3,2-dioxaborolane) (2b)**

Silica gel column chromatography (hexane /ethyl acetate = 10/1) gave **2b** (183 mg, 42% yield, 0.8 mmol scale) as white solid. mp. 116-118^◦^C. ^1^H NMR (400 MHz, CDCl_3_) δ 7.80 (d, *J* = 7.3 Hz, 4H), 7.33 (d, *J* = 7.3 Hz, 4H), 4.52 (s, 4H), 3.27 (s, 4H), 1.35 (s, 24H), 0.94 (s, 6H). ^13^C NMR (101 MHz, CDCl_3_) δ 142.4, 134.9, 126.7, 83.8, 76.5, 73.2, 36.5, 25.0, 22.4. ^11^B NMR (128 MHz, CDCl_3_) δ 30.6. IR (KBr): 2975, 2852, 1615, 1518, 1471, 1401, 1361, 1322, 1275, 1214, 1142, 1099, 1039, 1020, 962, 859, 824, 660 cm^–1^. HRMS-FAB (m/z): Calcd for C_31_H_47_B_2_O_6_ [M+H]^+^: 537.3558. Found: 537.3553.

**2,2'-((((Cyclopropane-1,1-diylbis(methylene))bis(oxy))bis(methylene))bis(4,1-phenylene))bis(4,4,5,5-tetramethyl-1,3,2-dioxaborolane) (3b)**

Silica gel column chromatography (hexane /ethyl acetate = 10/1) gave **3b** (85 mg, 69% yield, 0.2 mmol scale) as white solid. mp. 100-102^◦^C. ^1^H NMR (400 MHz, CDCl_3_) δ 7.78 (d, *J* = 7.8 Hz, 4H), 7.31 (d, *J* = 7.8 Hz, 4H), 4.53 (s, 4H), 3.39 (s, 4H), 1.33 (s, 24H), 0.47 (s, 4H). 13C NMR (101 MHz, CDCl_3_) δ 142.0, 134.9, 126.9, 83.8, 73.7, 72.8, 25.0, 20.9. ^11^B NMR (128 MHz CDCl_3_) δ 30.1. IR (KBr): 2978, 2937, 2870, 1615, 1518, 1483, 1469, 1401, 1360, 1321, 1273, 1214, 1142, 1089, 1020, 962, 860, 823, 739, 674, 658 cm^–1^. HRMS-FAB (m/z): Calcd for C_31_H_45_B_2_O_6_ [M+H]^+^: 535.3402. Found: 535.3407.

**2,2'-((((2,2-Diethylpropane-1,3-diyl)bis(oxy))bis(methylene))bis(4,1-phenylene))bis(4,4,5,5-tetramethyl-1,3,2-dioxaborolane) (4b)**

Silica gel column chromatography (hexane /ethyl acetate = 19/1) gave **4b** (286 mg, 79% yield, 0.64 mmol scale) as a white solid. mp. 100-101^◦^C. ^1^H NMR (400 MHz, CDCl_3_) δ 7.76 (d, *J* = 6.4 Hz, 4H), 7.30 (d, *J* = 6.8 Hz, 4H), 4.46 (s, 4H), 3.25 (s, 4H), 1.35-1.27 (m, 28H), 0.75 (t, *J* = 6.8 Hz, 6H). ^13^C NMR (101 MHz, CDCl_3_) δ 142.4, 134.8, 126.7, 83.8, 73.2, 72.5, 41.3, 25.0, 23.3, 7.3. ^11^B NMR (128 MHz, CDCl_3_) δ 31.6. IR (KBr): 2971, 2935, 2861, 1613, 1517,1482, 1463, 1395, 1360, 1322, 1270, 1213, 1143, 1114, 1098, 1020, 964, 859, 819, 728, 671, 656 cm^–1^. HRMS-FAB (m/z): Calcd for C_33_H_51_B_2_O_6_ [M+H]^+^: 565.3871. Found: 565.3883.

**2,2'-((((2,2-Diheptylpropane-1,3-diyl)bis(oxy))bis(methylene))bis(4,1-phenylene))bis(4,4,5,5-tetramethyl-1,3,2-dioxaborolane) (4d)**

Silica gel column chromatography (hexane /ethyl acetate = 19/1) gave **4d** (200 mg, 81% yield, 0.35 mmol scale) as colorless oil. ^1^H NMR (400 MHz, CDCl_3_) δ 7.77 (d, *J* = 8.0 Hz, 4H), 7.30 (d, *J* = 8.0 Hz, 4H), 4.47 (s, 4H), 3.25 (s, 4H), 1.33 (s, 24H), 1.30-1.12 (m, 24H) 0.87 (t, *J* = 7.0 Hz, 6H). ^13^C NMR (101 MHz, CDCl_3_) δ 142.5, 134.8, 126.7, 83.8, 73.1, 73.2, 41.2, 32.0, 31.7, 30.6, 29.4, 24.9, 22.8, 14.2. Due to signal overlap and symmetry, the number of observed carbon resonances is fewer than the total number of carbons in the molecular formula.  ^11^B NMR (128 MHz, CDCl_3_) δ 27.0. IR (neat) 2977, 2928, 2856, 1614, 1517, 1466, 1399, 1360, 1320, 1272, 1213, 1145, 1089, 1022, 962, 860, 822, 731, 658 cm^–1^. HRMS-FAB (m/z): Calcd for C_43_H_71_B_2_O_6_ [M+H]^+^: 705.5436. Found: 705.5447.

**2,2'-((((2,2-Dibenzylpropane-1,3-diyl)bis(oxy))bis(methylene))bis(4,1-phenylene))bis(4,4,5,5-tetramethyl-1,3,2-dioxaborolane) (6b)**

Silica gel column chromatography (hexane /ethyl acetate = 19/1) gave **6b** (574 mg, 83% yield, 1.0 mmol scale) as a white solid. mp. 170-172^◦^C. ^1^H NMR (400 MHz, CDCl_3_) δ 7.83 (d, *J* = 7.6 Hz, 4H), 7.41 (d, *J* = 7.6 Hz, 4H), 7.23-7.15 (m, 10H), 4.47 (s, 4H), 2.97 (s, 4H), 2.87 (s, 4H), 1.35 (s, 24H). ^13^C NMR (101 MHz CDCl_3_) δ 141.8, 138.5, 135.0, 130.8, 128.0, 127.0, 126.0, 83.9, 73.0, 70.2, 44.1, 39.2, 25.0. ^11^B NMR (128 MHz CDCl_3_) δ 31.7. IR (neat) 3028, 2978, 2931, 2859, 1615, 1518, 1455, 1400, 1359, 1321, 1272, 1213, 1144, 1088, 1021, 962, 859, 822, 753, 731, 703, 657 cm^–1^. HRMS-FAB (m/z): Calcd for C_43_H_55_B_2_O_6_ [M+H]^+^: 689.4184. Found: 689.4191.

**General procedure for the synthesis of bis benzyl alcohol species**


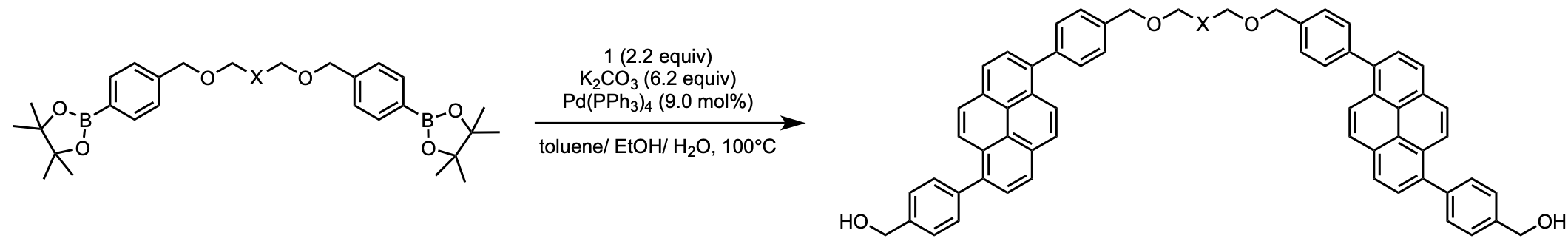


Under Ar atmosphere, the mixture of **compound b** (1.0 equiv), **7** (2.2 equiv), and potassium carbonate ( 6.2 equiv in water), in toluene and of EtOH was added to Pd(PPh _3_)_4_ (9.0 mol%). The mixture was stirred at 100^◦^C (oil bath) and the reaction was monitored by TLC. After the reaction was completed, the reaction mixture was filtered. The precipitate was washed by Water, MeOH, and CH_2_Cl_2_. The residue was dried under reduced pressure. Crystallization of crude product gave compound **c** and was used for the next reaction without further puriﬁcation.

**(((((Propane-1,3-diylbis(oxy))bis(methylene))bis(4,1-phenylene))bis(pyrene-6,1-diyl))bis(4,1-phenylene))dimethanol (1c).**

Crystallized from CHCl_3_ and hexane give **1c** as pale yellow solid (345 mg, 0.6 mmol scale).

HRMS-FAB (m/z): Calcd for C_63_H_49_O_4_ [M+H]^+^: 869.3631. Found: 869.3623.

**((((((2,2-Dimethylpropane-1,3-diyl)bis(oxy))bis(methylene))bis(4,1-phenylene))bis(pyrene-6,1-diyl))bis(4,1-phenylene))dimethanol (2c).**

Crystallized from CHCl_3_ and hexane give **2c** as pale yellow solid (290 mg, 0.6 mmol scale).

HRMS-FAB (m/z): Calcd for C_65_H_53_O_4_ [M+H]^+^: 897.3944. Found: 897.3948.

**((((((Cyclopropane-1,1-diylbis(methylene))bis(oxy))bis(methylene))bis(4,1-phenylene))bis(pyrene-6,1-diyl))bis(4,1-phenylene))dimethanol (3c).**

Crystallized from CHCl_3_ and hexane give **3c** as pale yellow solid (488 mg, 0.8 mmol scale).

HRMS-FAB (m/z): Calcd for C_65_H_51_O_4_ [M+H]^+^: 895.3787. Found: 895.3774.

**((((((2,2-Diethylpropane-1,3-diyl)bis(oxy))bis(methylene))bis(4,1-phenylene))bis(pyrene-6,1-diyl))bis(4,1-phenylene))dimethanol (4c).**

Crystallized from CHCl_3_ and hexane give **4c** as pale yellow solid (280 mg, 0.35 mmol scale).

HRMS-FAB (m/z): Calcd for C_67_H_56_O_4_ [M]^+^: 924.4179. Found: 924.4162.

**((((((2,2-Diheptylpropane-1,3-diyl)bis(oxy))bis(methylene))bis(4,1-phenylene))bis(pyrene-6,1-diyl))bis(4,1-phenylene))dimethanol (5c).**

Crystallized from CHCl_3_ and hexane give **5c** as pale yellow solid (118 mg, 0.23 mmol scale).

HRMS-FAB (m/z): Calcd for C_77_H_76_O_4_ [M]^+^: 1064.5744. Found: 1064.5753.

**((((((2,2-Dibenzylpropane-1,3-diyl)bis(oxy))bis(methylene))bis(4,1-phenylene))bis(pyrene-6,1-diyl))bis(4,1-phenylene))dimethanol (6c).**

Crystallized from CHCl_3_ and hexane gave **6c** as pale yellow solid (311 mg, 0.35 mmol scale).

HRMS-FAB (m/z): Calcd for C_77_H_60_O_4_ [M]^+^: 1048.4492. Found: 1048.4519.

**General procedure for the synthesis of Pyr-A derivatives**

Under Ar atmosphere, to a stirring solution of compound **c** (1.0 equiv) in THF and DMF was added NaH (60% dispersion in oil, 13.8 equiv) at 0^◦^C. After 1 hour, poly(ethylene glycol) methyl ether tosylate (Mn 900 g/mol) (3.0 equiv) was slowly added. The resulting mixture was warmed up to room temperature and the reaction was monitored by TLC. After the reaction was completed, the reaction mixture was cooled to 0^◦^C and poured into water. The aqueous layer was separated and extracted with CH_2_Cl_2_. The combined organic layers were washed with Brine and dried over Na_2_SO_4_. Concentration and purified by Silica gel column chromatography to crude products. Crude product was separated by size exclusion chromatography (CHCl_3_) to afford Pyr-A derivative.

**1,3-Bis((4-(6-(4-((2-methoxyethoxy)methyl)phenyl)pyren-1-yl)benzyl)oxy)propane (#1)**

Silica gel column chromatography (CHCl_3_ /MeOH = 80/1~9/1) gave **#1** (12 mg, 5% yield, 0.1 mmol scale) as pale yellow solid. M p= 35-37^◦^C. ^1^H NMR (400 MHz, CDCl_3_) δ 8.18-8.08 (m, 8H), 7.86-7.80 (m, 8H), 7.65-7.51 (m, 16H), 4.70 (d, *J* = 6.4 Hz, 8H), 3.88 (t, *J* = 6.4 Hz, 4H), 3.75-3.44 (m, –OCH₂CH₂–), 3.36 (s, 6H), 2.12-2.06 (m, 2H). The integral values of the poly(ethylene glycol) methylene protons were not explicitly assigned due to severe signal overlap. ^13^C NMR (101 MHz, CDCl_3_) δ 140.70, 140.67, 137.6, 137.4, 130.77, 130.73, 130.4, 128.9, 127.9, 127.8, 127.5, 125.3, 125.2, 124.6, 73.3, 73.0, 72.0, 70.8, 70.6, 69.8, 67.7, 59.1, 30.4. Due to signal overlap and symmetry, the number of observed carbon resonances is fewer than the total number of carbons in the molecular formula. IR (KBr) 3433, 2884, 1638, 1466, 1346, 1280, 1242, 1117, 951, 843, 728, 682, cm^–1^.

**6,6'-((((2,2-Dimethylpropane-1,3-diyl)bis(oxy))bis(methylene))bis(4,1-phenylene))bis(1-(4-((2-methoxyethoxy)methyl)phenyl)pyrene) (#2)**

Silica gel column chromatography (CHCl_3_ /MeOH = 80/1~9/1) gave **#2** (12 mg, 4% yield, 0.1 mmol scale) as pale yellow solid. mp 37-39^◦^C. ^1^H NMR (400 MHz, CDCl_3_) δ 8.17-8.13 (m, 4H), 8.09 (d, *J* = 7.8 Hz, 4H), 7.97 (s, 1H), 7.94 (d, *J* = 3.2 Hz, 2H), 7.92 (d, *J* = 2.3 Hz, 3H), 7.90 (d, *J* = 2.3 Hz, 2H), 7.60-7.58 (m, 8H), 7.55-7.52 (m, 8H), 4.71 (s, 8H), 3.79-3.52 (m, –OCH₂CH₂–), 3.48 (s, 4H), 3.36 (s, 6H), 1.08 (s, 6H). The integral values of the poly(ethylene glycol) methylene protons were not explicitly assigned due to severe signal overlap. ^13^C NMR (101 MHz, CDCl_3_) δ 140.7, 140.3, 138.2, 137.6, 137.5, 137.3, 130.7, 130.6, 130.41, 130.37, 128.8, 127.92, 127.85, 127.77, 127.50, 127.46, 127.35, 125.3, 125.2, 124.56, 124.52, 73.2, 73.1, 72.0, 70.82, 70.78, 70.6, 69.8, 67.7, 59.1, 36.6, 22.6. Due to signal overlap and symmetry, the number of observed carbon resonances is fewer than the total number of carbons in the molecular formula. IR (KBr) 3434, 2884, 1368, 1466, 1345, 1280, 1241, 1112, 963, 843, 727, 682 cm^–1^.

**6,6'-((((Cyclopropane-1,1-diylbis(methylene))bis(oxy))bis(methylene))bis(4,1-phenylene))bis(1-(4-((2-methoxyethoxy)methyl)phenyl)pyrene) (#3).**

Silica gel column chromatography (CHCl_3_ /MeOH = 80/1~9/1) gave **#3** (12 mg, 4% yield, 0.1 mmol scale) as pale yellow solid. mp 37-39^◦^C. ^1^H NMR (400 MHz, CDCl_3_) δ 8.16 (dd, *J* = 9.1, 2.0 Hz, 4H), 8.10 (d, *J* = 7.8 Hz, 4H), 8.01-7.87 (m, 8H), 7.62-7.52 (m, 16H), 4.71 (d, 8H, *J* = 4.6 Hz), 3.80-3.52 (m, –OCH₂CH₂–), 3.36 (s, 6H), 0.64 (s, 4H).^13^C NMR (101 MHz, CDCl_3_) δ 140.7, 140.6, 137.8, 137.6, 137.6, 137.4, 130.7, 130.4, 128.6, 127.9, 127.8, 127.5, 125.3, 125.2, 124.6, 74.1, 73.3, 72.9, 72.0, 70.8, 70.8, 70.6, 69.8, 59.1, 21.1, 8.9. IR (KBr) 3504, 2871, 1710, 1638, 1458, 1361, 1290, 1222, 1104, 951, 848, 729, 683 cm^–1^.

**6,6'-((((2,2-Diethylpropane-1,3-diyl)bis(oxy))bis(methylene))bis(4,1-phenylene))bis(1-(4-((2-methoxyethoxy)methyl)phenyl)pyrene) (#4)**

Silica gel column chromatography (CHCl_3_ /MeOH = 80/1~9/1) gave **#4** (18 mg, 14% yield, 0.05 mmol scale) as pale yellow solid. mp 37-39^◦^C. ^1^H NMR (400 MHz, CDCl_3_) δ 8.16-8.13 (dd, *J* = 6.0, 6.0 Hz, 4H), 8.08 (d, *J* = 8.0 Hz, 4H), 7.97 (s, 1H), 7.95-7.89 (m, 7H), 7.59 (d, *J* = 8.4 Hz, 8H), 7.55-7.52 (dd, *J* = 3.6, 4.0 Hz, 8H), 4.70 (d, *J* = 9.2 Hz, 8H), 3.79-3.52 (m, –OCH₂CH₂–), 3.50 (s, 4H), 3.36 (s, 6H), 1.47 (q, *J* = 7.5 Hz, 4H), 0.90 (t, *J* = 7.6 Hz, 6H). The integral values of the poly(ethylene glycol) methylene protons were not explicitly assigned due to severe signal overlap. ^13^C NMR (101 MHz, CDCl_3_) δ 140.7, 140.3, 138.3, 137.7, 137.5, 137.4, 130.7, 130.6, 130.43, 130.38, 128.9, 127.9, 127.8, 127.7, 127.50, 127.46, 127.3, 125.3, 125.2, 124.54, 124.51, 73.3, 73.2, 72.7, 72.0, 70.82, 70.79, 70.6, 69.8, 59.1, 41.5, 29.8, 23.4. Due to signal overlap and symmetry, the number of observed carbon resonances is fewer than the total number of carbons in the molecular formula. IR (neat) 2883, 1466, 1359, 1344, 1280, 1241, 1110, 962, 843, 725, 681 cm^–1^.

**6,6'-((((2,2-Diheptylpropane-1,3-diyl)bis(oxy))bis(methylene))bis(4,1-phenylene))bis(1-(4-((2-methoxyethoxy)methyl)phenyl)pyrene) (#5).**

Silica gel column chromatography (CHCl_3_ /MeOH = 80/1~9/1) gave **#5** (20 mg, 15% yield, 0.05 mmol scale) as pale yellow solid. Mp = 47-49^◦^C. ^1^H NMR (400 MHz, CDCl_3_) δ 8.17-8.13 (dd, *J* = 8.4, 8.4 Hz, 4H) 8.08 (d, *J* = 8.0 Hz, 4H), 7.97 (s, 1H), 7.95-7.90 (ddd, *J* = 4.4, 3.2, 3.2 Hz, 7H), 7.61-7.58 (dd, *J* = 2.0, 1.6 Hz, 8H), 7.53 (d, *J* = 7.6 Hz, 8H), 4.71 (s, 4H), 4.68 (s, 4H), 3.79-3.52 (m, –OCH₂CH₂–), 3.49 (s, 4H), 3.36 (s, 6H), 1.38-1.24 (m, 24H), 0.87 (t, *J* = 6.8 Hz, 6H). The integral values of the poly(ethylene glycol) methylene protons were not explicitly assigned due to severe signal overlap. ^13^C NMR (101 MHz, CDCl_3_) δ 140.7, 140.3, 138.3, 137.7, 137.6, 137.4, 130.7, 130.6, 130.44, 130.38, 128.9, 127.9, 127.84, 127.75, 127.5, 127.44, 127.38, 125.3, 125.2, 124.54, 124.50, 73.4, 73.2, 73.1, 72.0, 70.82, 70.79, 70.6, 69.8, 59.1, 41.4, 32.0, 31.7, 30.6, 29.4, 22.9, 22.7, 14.2. Due to signal overlap and symmetry, the number of observed carbon resonances is fewer than the total number of carbons in the molecular formula. IR (KBr) 2883, 1465, 1344, 1280, 1242, 1107, 962, 839 cm^–1^.

**6,6'-((((2,2-Dibenzylpropane-1,3-diyl)bis(oxy))bis(methylene))bis(4,1-phenylene))bis(1-(4-((2-methoxyethoxy)methyl)phenyl)pyrene) (#6)**

Silica gel column chromatography (CHCl_3_ /MeOH = 80/1~9/1) gave **#6** (15 mg, 5% yield, 0.05 mmol scale) as pale yellow solid. Mp = 29-31^◦^C. ^1^H NMR (400 MHz, CDCl_3_) δ 8.23 (d, *J* = 9.2 Hz, 4H), 8.19-8.15 (m, 4H), 8.03-7.94 (m, 8H), 7.69-7.60 (m, 12H), 7.54 (d, *J* = 9.2 Hz, 4H), 7.32-7.21 (m, 10H), 4.71 (s, 4H), 4.65 (s, 4H), 3.79-3.52 (m, –OCH₂CH₂–), 3.36 (s, 6H), 3.14 (s, 4H), 3.01 (s, 4H). The integral values of the poly(ethylene glycol) methylene protons were not explicitly assigned due to severe signal overlap.^13^C NMR (101 MHz, CDCl_3_) δ 140.7, 138.5, 137.6, 137.4, 130.9, 130.7, 130.5, 128.9, 128.0, 127.9, 127.5, 126.1, 125.3, 125.2, 124.6, 73.2, 72.9, 72.0, 71.0, 70.82, 70.79, 70.6, 70.2, 69.8, 59.1, 44.2, 39.2. Due to signal overlap and symmetry, the number of observed carbon resonances is fewer than the total number of carbons in the molecular formula. IR (KBr) 3466, 2885, 1466, 1344, 1279, 1241, 1108, 961, 841 cm^–1^.

**1,6-Bis(4-((2-methoxyethoxy)methyl)phenyl)pyrene (Pyr-M)**

To a solution of (pyrene-1,6-diylbis(4,1-phenylene))dimethanol (30 mg, 0.07 mmol) in dry THF (0.5 mL) and dry DMF (0.6 mL) was slowly added NaH (60% dispersion in oil, 25 mg, 0.63 mmol) at 0 °C under N_2_. After stirring for 2 h, poly(ethylene glycol) [methyl ether](https://www.sciencedirect.com/topics/pharmacology-toxicology-and-pharmaceutical-science/dimethyl-ether) tosylate (*M*_n_ = 900 gmol^-1^, 260 mg, 0.29 mmol) was added. The reaction mixture was allowed to warm to room temperature and stirred for 37 h. The resulting mixture was quenched with saturated NH_4_Cl aq. and then extracted with CH_2_Cl_2_. The organic layer was concentrated under reduced pressure. The residue was purified by silica gel [column chromatography](https://www.sciencedirect.com/topics/biochemistry-genetics-and-molecular-biology/column-chromatography) and [size exclusion chromatography](https://www.sciencedirect.com/topics/agricultural-and-biological-sciences/size-exclusion-chromatography) to obtain **Pyr-M** (23 mg, 0.012 mmol, 16%). ^1^H NMR (400 MHz, CDCl_3_) δ 8.20 (d, *J* = 8.0 Hz, 2H), 8.19 (d, *J* = 9.2 Hz, 2H), 8.04 (d, *J* = 9.4 Hz, 2H), 7.97 (d, *J* = 7.8 Hz, 2H), 7.62 (d, *J* = 8.1 Hz, 4H), 7.55 (d, *J* = 8.1 Hz, 4H), 4.72 (s, 4H), 3.82-3.46 (m, –OCH₂CH₂–), 3.37 (s, 6H). The integral values of the poly(ethylene glycol) methylene protons were not explicitly assigned due to severe signal overlap.^13^C NMR (101 MHz, CDCl_3_) δ 140.7, 137.7, 137.5, 130.7, 130.5, 129.0, 127.9, 127.9, 127.6, 125.4, 125.3, 124.6, 73.3, 72.0, 70.9, 70.8, 70.8, 70.7, 70.7, 70.7, 70.6, 69.8, 59.1. IR (KBr) 3448, 2871, 1643, 1458, 1351, 1299, 1251, 1105, 952, 849 cm^–1^.

**Cell viability assay**

The effects of the dipyrene-based probes on cell viability were evaluated using an MTT assay. HeLa cells were treated with each dipyrene-based probe at a final concentration of 10 μM for 48 h under standard culture conditions, and cell viability was measured according to the previously described protocol.

**Mono-pyrene control measurement**

To evaluate possible intermolecular excimer formation, fluorescence emission spectra of a mono-pyrene control probe were measured under the same conditions used for PyLUMI characterization. Because the mono-pyrene control contains only one pyrene chromophore, any excimer-like emission would reflect intermolecular pyrene–pyrene encounters. The spectra were compared with those of PyLUMI to assess whether intermolecular excimer formation contributed detectably under the tested concentration range.

**Polarity-dependent fluorescence measurements**

Pyr-A and PyLUMI (#2) were measured in water/THF mixtures with different water:THF volume ratios at a final probe concentration of 1 μM. Fluorescence emission spectra were acquired at 25 ± 1 °C with excitation at 358 nm. E/M ratios were calculated from the corresponding excimer and monomer emission intensities and used for the dimensionless polarity-sensitivity analysis.

**Excitation-induced DNA damage assay**

To evaluate potential DNA damage caused by short-wavelength excitation, cells were seeded on glass-bottom dishes and irradiated at the center of the dish using 350-nm illumination for 20, 200, or 400 ms. The surrounding area of the dish was shielded with aluminum foil to generate irradiated and non-irradiated regions within the same dish. Three hours after illumination, cells were fixed and subjected to immunofluorescence staining using an anti-γH2AX antibody. Nuclei were counterstained with DAPI. Nuclear γH2AX intensity was quantified from irradiated and shielded regions under identical image-acquisition settings.

Reference

1. Thomas, B.; David C, M.; Markus, G.; Klaus, M.; Oskar, N. Crosslinkable Hole-Transporting Polymers by Palladium Catalyzed C N-Coupling Reaction. *Macromol. Rapid Commun*. **2000**, *21,* 583-589.
2. Hazawa, M.; Amemori, S.; Nishiyama, Y.; Iga, Y.; Iwashima, Y.; Kobayashi, A.; Nagatani, H.; Mizuno, M.; Takhashi, K.; Richard W, W. A light-switching pyrene probe to detect phase-separated biomolecules. *iScience.* **2021**, *24*, 102865.


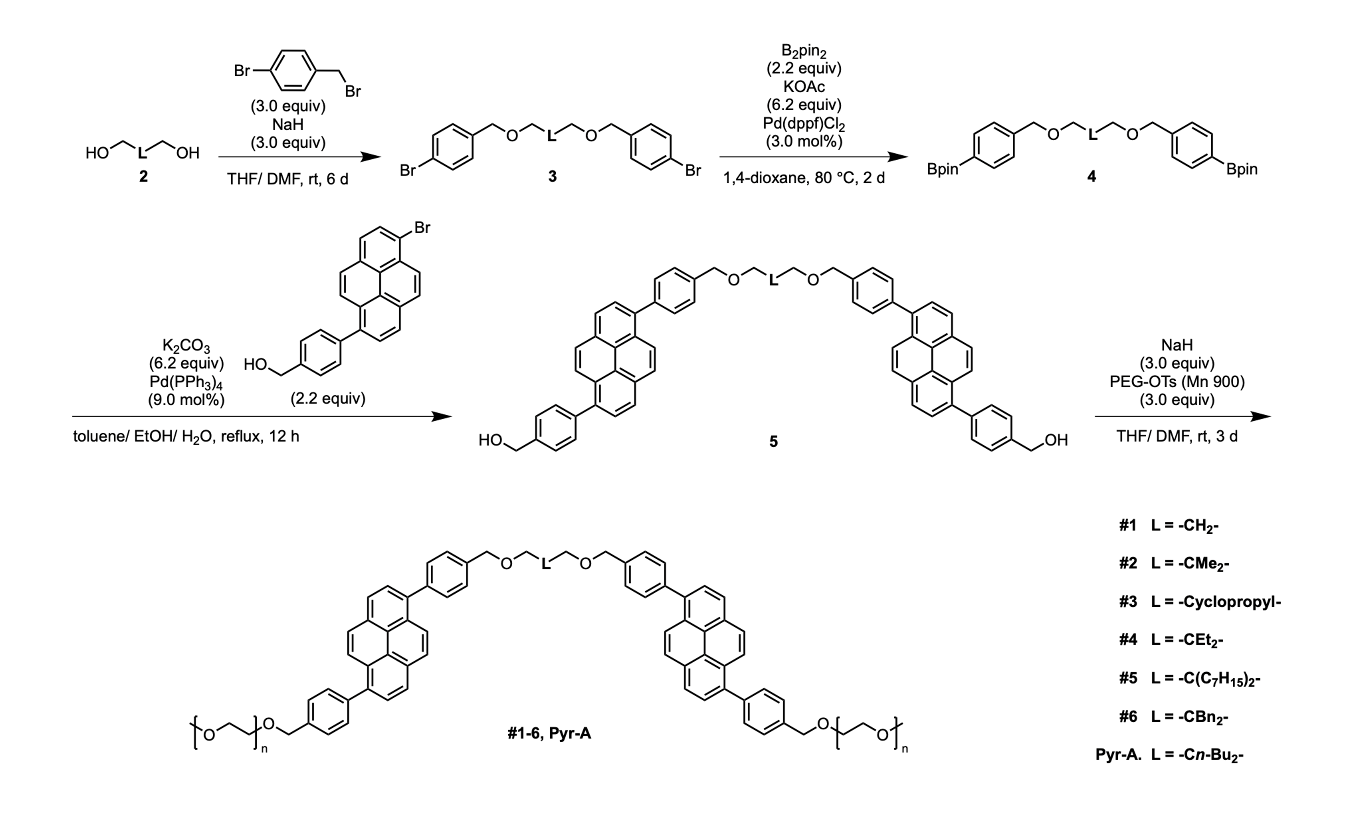


**Supplementary Figure S1. Synthetic route overview for pyrene-based rheological probes with various linkers.**

Schematic illustration of the synthesis of PEGylated bis-pyrene derivatives with tunable linker structures (L). Starting from ethylene glycol derivatives (2), the key intermediate 5 was synthesized via etherification and Suzuki–Miyaura coupling. Final PEG conjugation yielded the amphiphilic probes. The linker L was systematically varied as 1,3-propanediol (#1), 2,2-dimethyl-1,3-propanediol (#2), 1,1-bis(hydroxymethyl)cyclopropane (#3), 2,2-diethyl-1,3-propanediol (#4), 2,2-diheptyl-1,3-propanediol (#5), and 2,2-dibenzyl-1,3-propanediol (#6), and tert-butyl (Pyr-A).


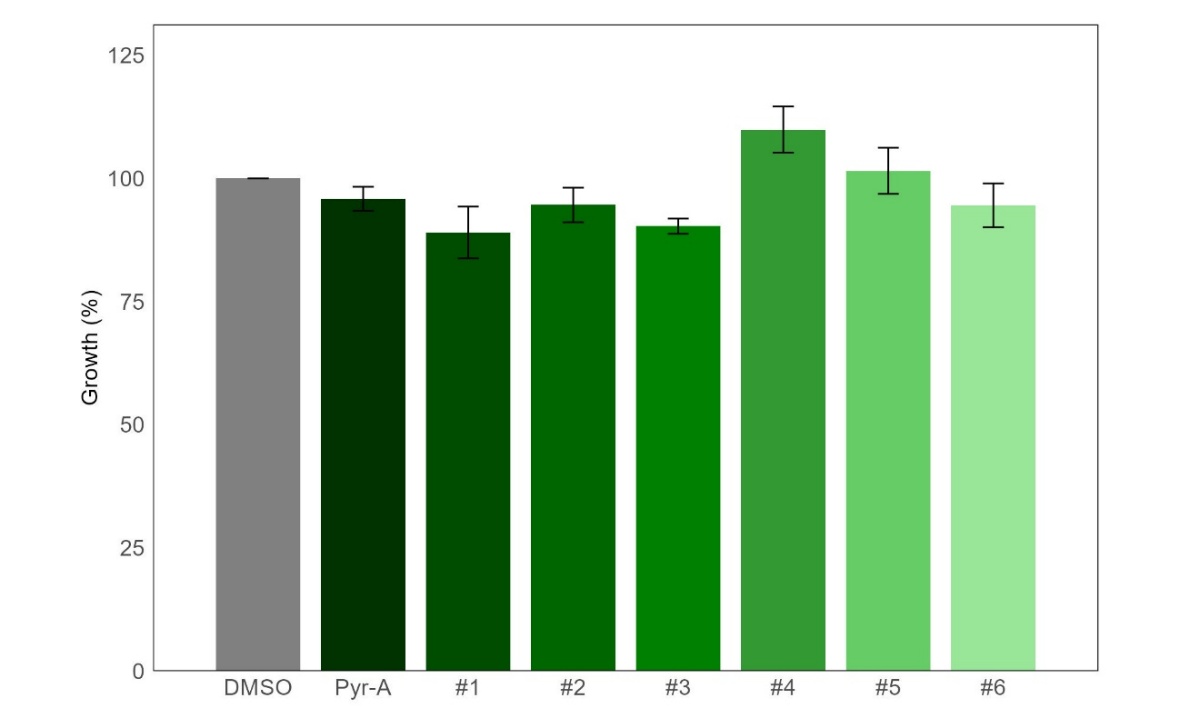


**Supplementary Figure S3. The effects of dipyrene-based probes on cell viability.**

The effects of dipyrene-based probes (10μM, 48 h) on cell viability, evaluated using the MTT assay (n = 3, mean ± SEM).


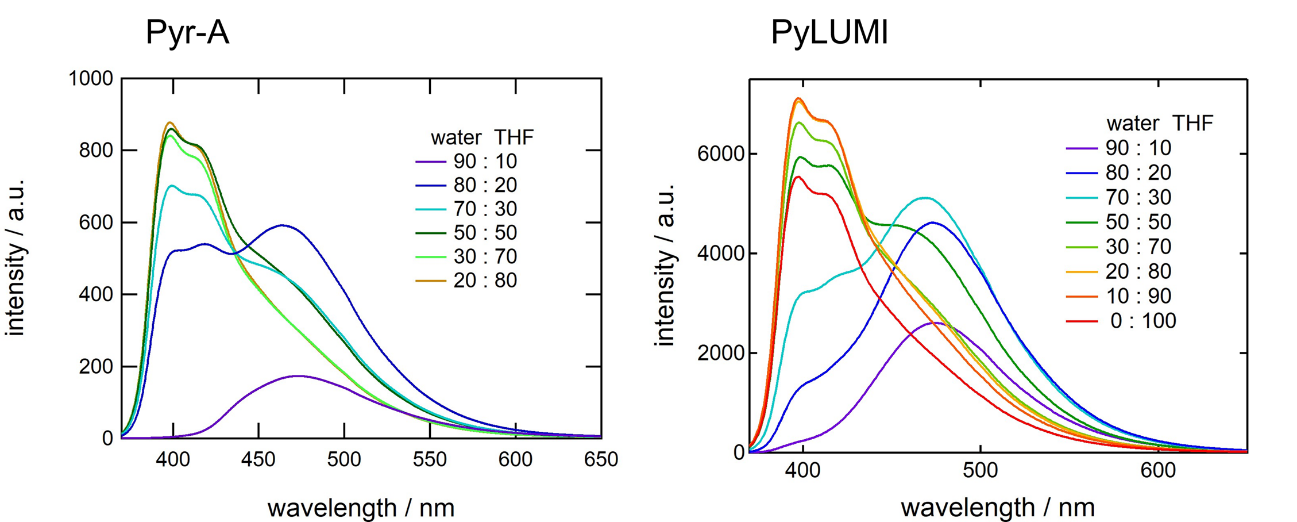


**Supplementary Figure S4.** Polarity-dependent fluorescence emission spectra of Pyr-A and PyLUMI. Steady-state fluorescence emission spectra of Pyr-A and PyLUMI measured in water/THF mixtures with the indicated water:THF ratios. Probe concentration was 1 μM, and the excitation wavelength was 358 nm. The spectra show solvent polarity-dependent modulation of the monomer and excimer emission bands. In water-rich, higher-polarity mixtures, the relative contribution of the excimer emission band increased, demonstrating that polarity/solvation affects the E/M ratio


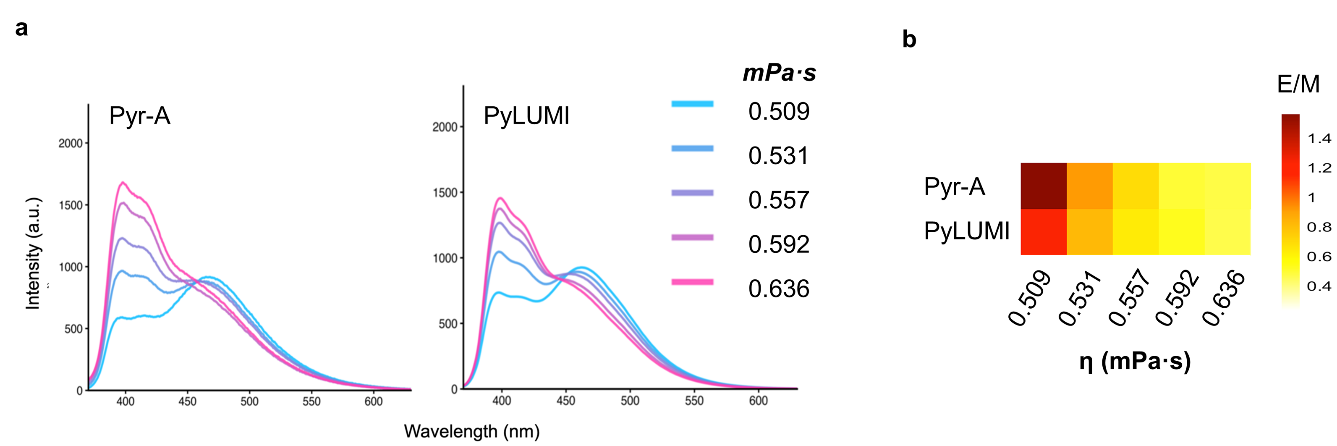


**Supplementary Figure S5. Viscosity-indexed fluorescence responses of Pyr-A and PyLUMI in MeOH/PEG 600 mixtures.**

**a) Steady-state fluorescence emission spectra of Pyr-A and PyLUMI were measured in MeOH/PEG 600 mixtures with the indicated bulk viscosities: 0.509, 0.531, 0.557, 0.592, and 0.636 mPa·s. Probe concentration was 1 μM, and the excitation wavelength was 358 nm. b) The heatmap summarizes E/M ratios calculated from the spectra. These data show viscosity-indexed modulation of the monomer and excimer emission profiles and were used for the dimensionless sensitivity analysis of viscosity-weighted E/M responsiveness.**


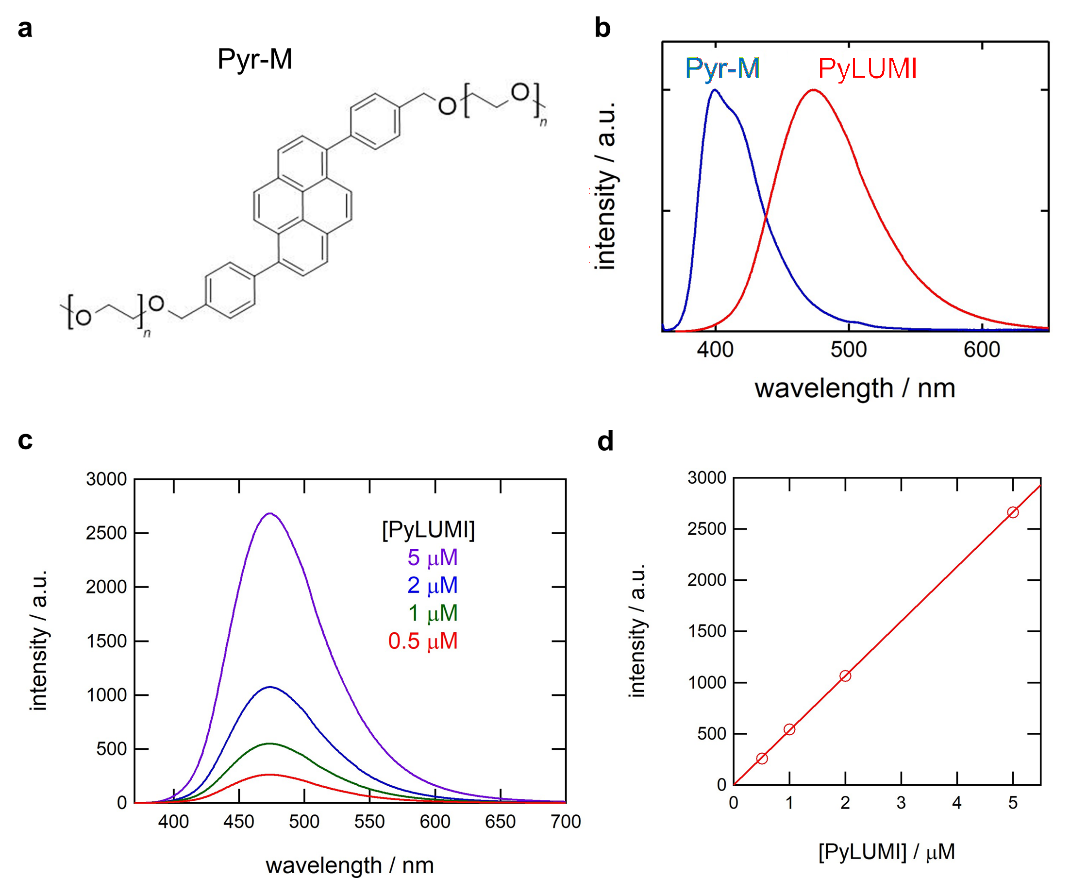


**Supplementary Figure S6. Mono-pyrene control analysis supporting intramolecular excimer formation in PyLUMI.** a) Chemical structure of the mono-pyrene control probe, Pyr-M. Pyr-M contains only one pyrene chromophore b) Fluorescence emission spectra of Pyr-M and PyLUMI at 1 μM measured in aqueous solution. c) Concentration-dependent fluorescence emission spectra of PyLUMI measured in aqueous solution at 0.5, 1, 2, and 5 μM.　d) Relationship between PyLUMI concentration and fluorescence intensity at the excimer emission maximum. The red line indicates a linear fit.


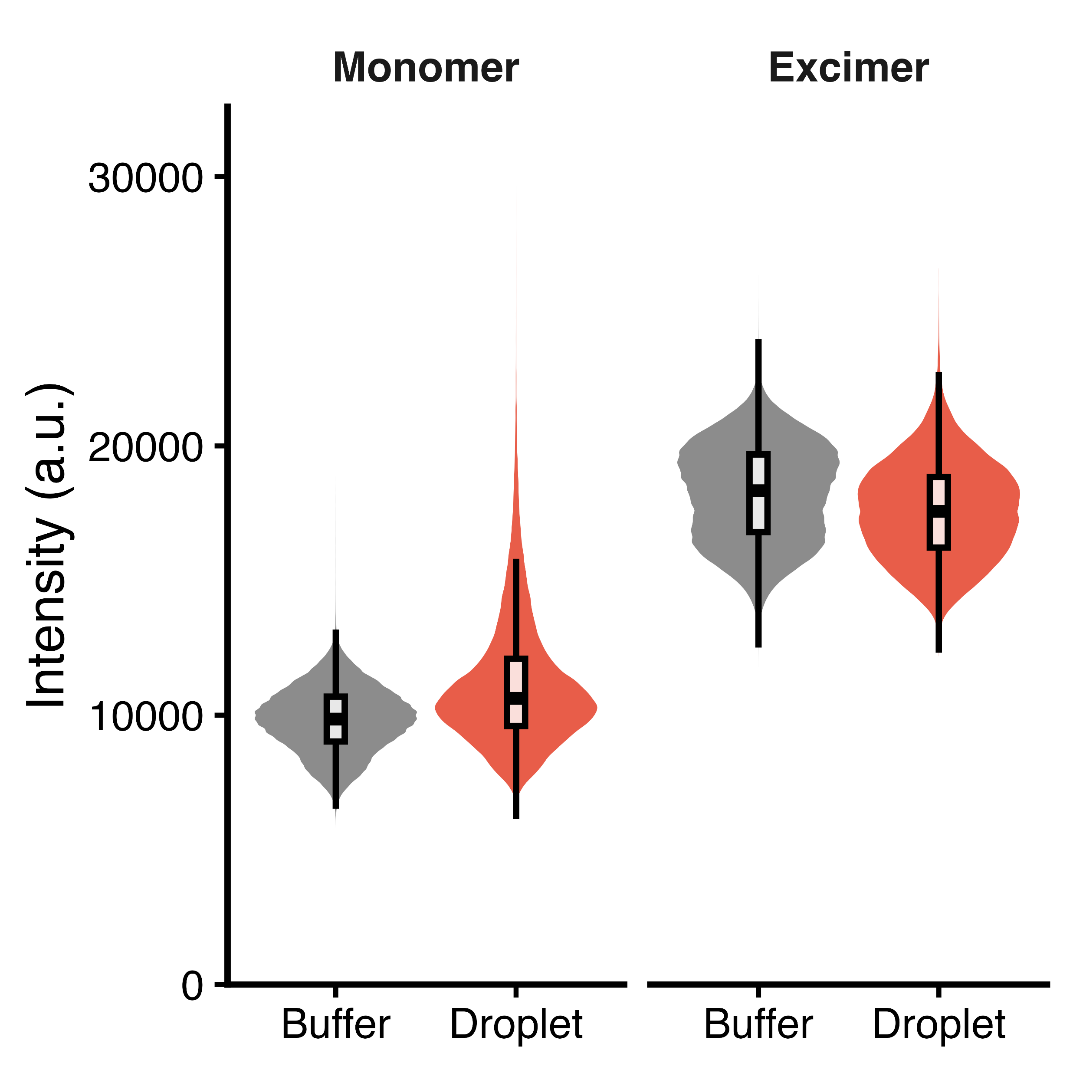


**Supplementary Figure S7. Monomer and excimer fluorescence intensities in droplets and the surrounding dilute phase**

Fluorescence intensities were quantified for the monomer and excimer emission channels inside droplets and in the surrounding dilute medium (buffer). Violin plots show the distribution of intensity values (a.u.), with embedded box plots indicating the median and interquartile range (whiskers, 1.5×IQR). Grey indicates buffer, and red indicates droplet.


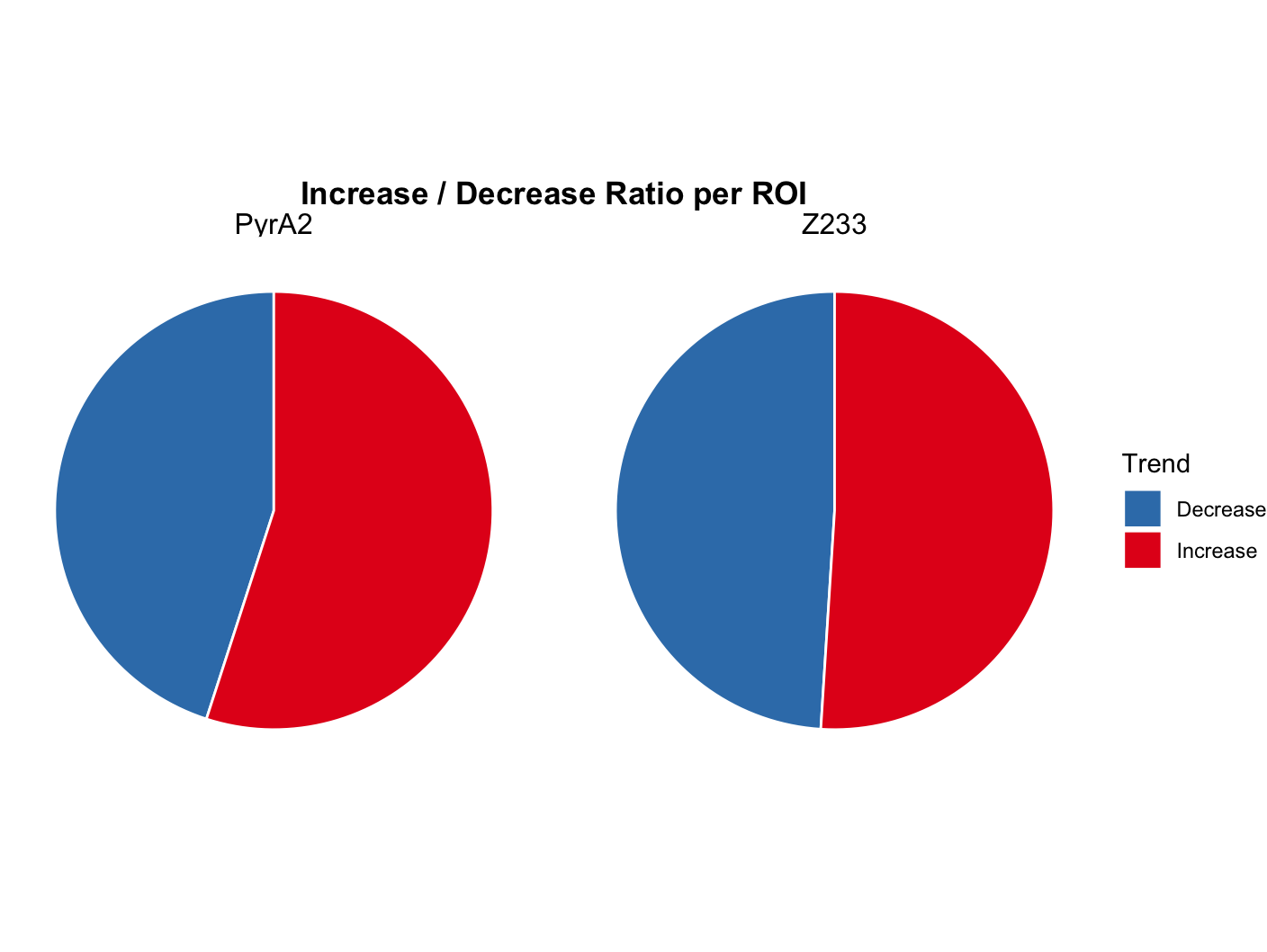


Pearson correlation (r > 0)

Pearson correlation (r < 0)

51.0%

(127 droplets)

49.0%

(122 droplets)

**Supplementary Figure S8. Droplets split into positive and negative pixel-wise correlations between E/M and mCherry intensity.**

For each droplet ROI, we calculated the Pearson correlation coefficient (r) between pixel-wise E/M ratio values and pixel-wise mCherry intensity. Droplets were classified by the sign of r (r > 0, red; r < 0, blue), and the pie chart shows the fraction of droplets in each category. Numbers indicate the percentage and droplet count (N = 249 droplets).


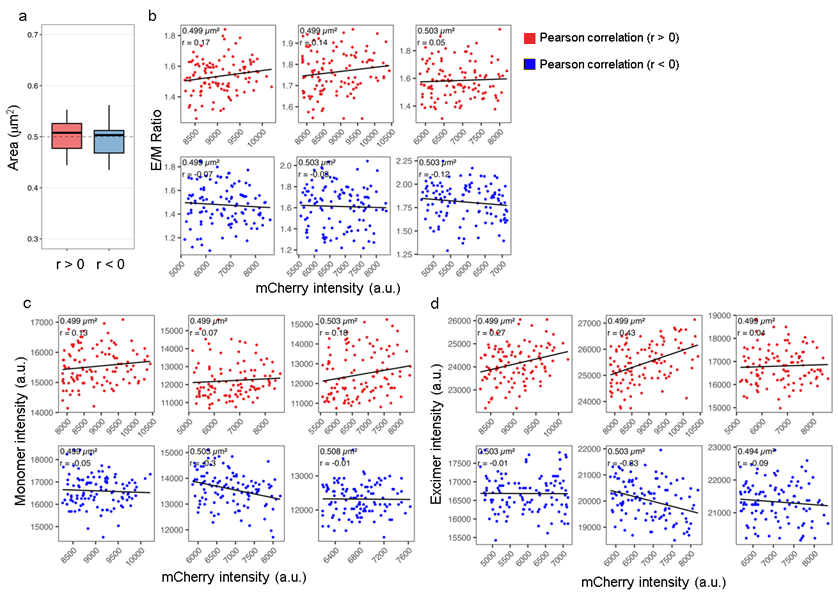


**Supplementary Figure S9. Size-matched droplets exhibit both positive and negative pixel-wise correlations between mCherry intensity and probe readouts.**

(a) Droplet areas (µm²) for the size-matched droplet cohort classified by the sign of the Pearson correlation coefficient (r) between pixel-wise E/M ratio and pixel-wise mCherry intensity (r > 0, red; r < 0, blue; n = 20 droplets per group). Box plots show the median and interquartile range (whiskers, 1.5×IQR). (b–d) Representative examples (three droplets per class) showing pixel-wise scatter plots of mCherry intensity (x-axis, a.u.) versus (b) E/M ratio, (c) monomer intensity (a.u.), and (d) excimer intensity (a.u.). Each dot represents a single pixel within the droplet ROI. Solid lines indicate linear fits; the droplet area and Pearson r are annotated in each panel.


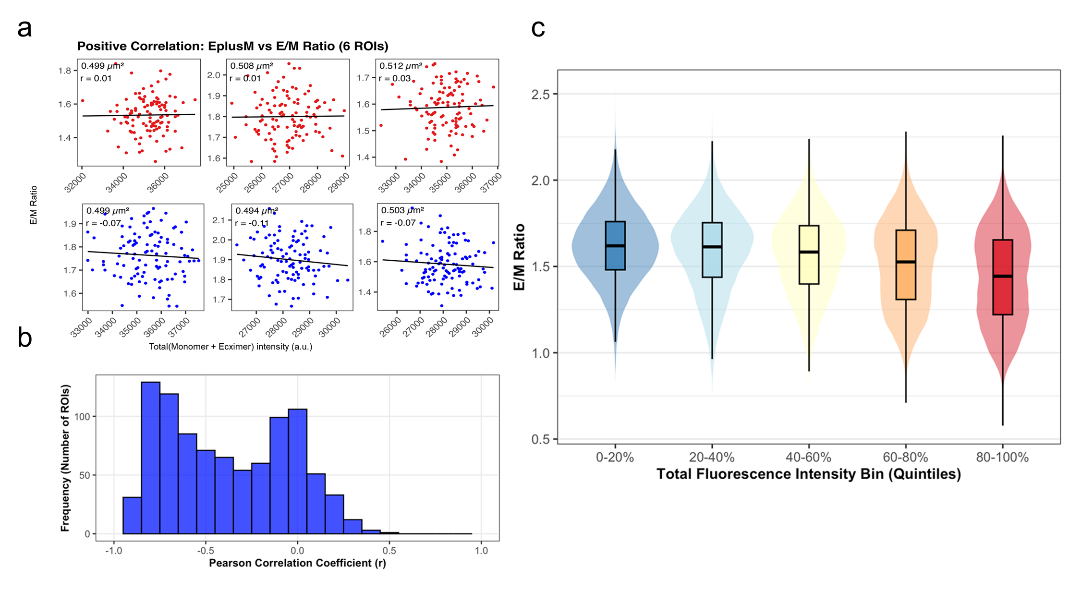


**Supplementary Figure S10. Total-intensity analysis of pixel-wise PyLUMI E/M ratios in BRD4-IDR condensates.** a) Representative pixel-wise scatter plots comparing total PyLUMI fluorescence intensity and E/M ratio within individual BRD4-IDR droplet ROIs. Total PyLUMI fluorescence intensity was calculated as the sum of excimer and monomer intensities. Each point represents a pixel within the droplet ROI. The black line indicates a linear fit, and the Pearson correlation coefficient, r, is shown in each plot. b) Distribution of Pearson correlation coefficients between total PyLUMI fluorescence intensity and E/M ratio across droplet ROIs (919 droplets). c) Intensity-binned analysis of E/M ratio distributions. Pixels were pooled and stratified into five quantile bins according to total PyLUMI fluorescence intensity after removal of outliers using the 1.5×IQR criterion. Violin plots with embedded box plots show the E/M ratio distribution in each intensity bin. The broad overlap of E/M distributions across intensity bins indicates that pixel-wise E/M values are not simply determined by total fluorescence intensity.


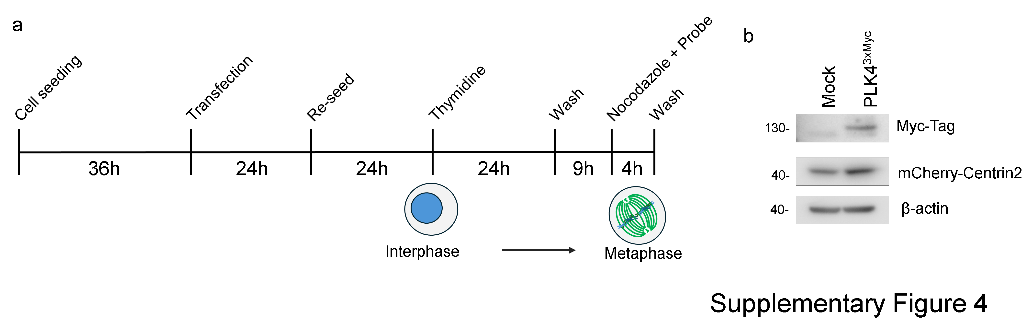


**Supplementary Figure S11. Experimental workflow and model of centrosome amplification induced by PLK4 overexpression.**

a) Schematic illustration of the working model and experimental design. b) Western blot analysis confirming the expression of exogenous centrin2-mCherry as well as PLK4.


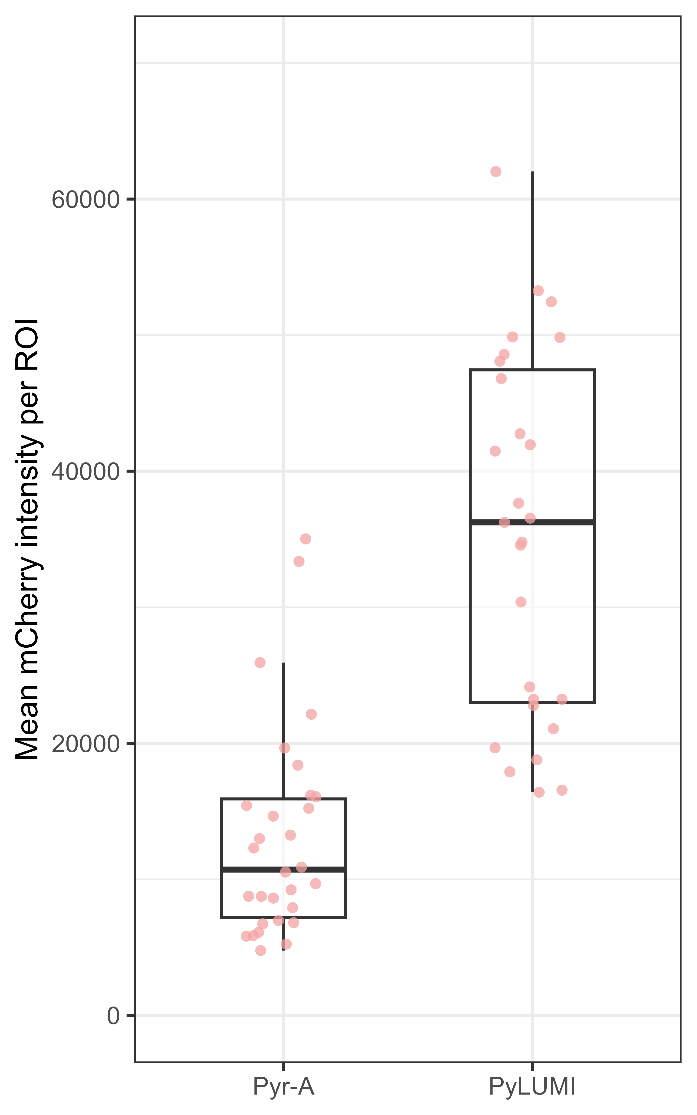


**Supplementary Figure S12. Pyr-A imaging conditions reduce interphase mCherry signal compared with PyLUMI**

Interphase cells expressing mCherry were imaged under the conditions used for centrosome E/M mapping with either Pyr-A (10 µM; 200 ms exposure per channel) or PyLUMI (1 µM; 20 ms exposure per channel). The plot shows the distribution of mean mCherry intensity per ROI for each condition. Box plots indicate the median and interquartile range (whiskers, 1.5×IQR), with individual points representing single ROIs.
