# Supplementary material for: A Bright Ratiometric Dipyrene Probe for Functional Imaging of Condensate Microenvironments in Living Cells
Source: Adv Sci (Weinh). 2026 Jul 16:e76419. Online ahead of print. doi: 10.1002/advs.76419 (PMC13374536; doi:10.1002/advs.76419)
Supplement: Supplementary file 2 — Supporting File 2: advs76419‐sup‐0002‐FigureS2_NMR.pdf. [file ADVS-9999-e76419-s001.pdf]

KS-4-45-fr
1H

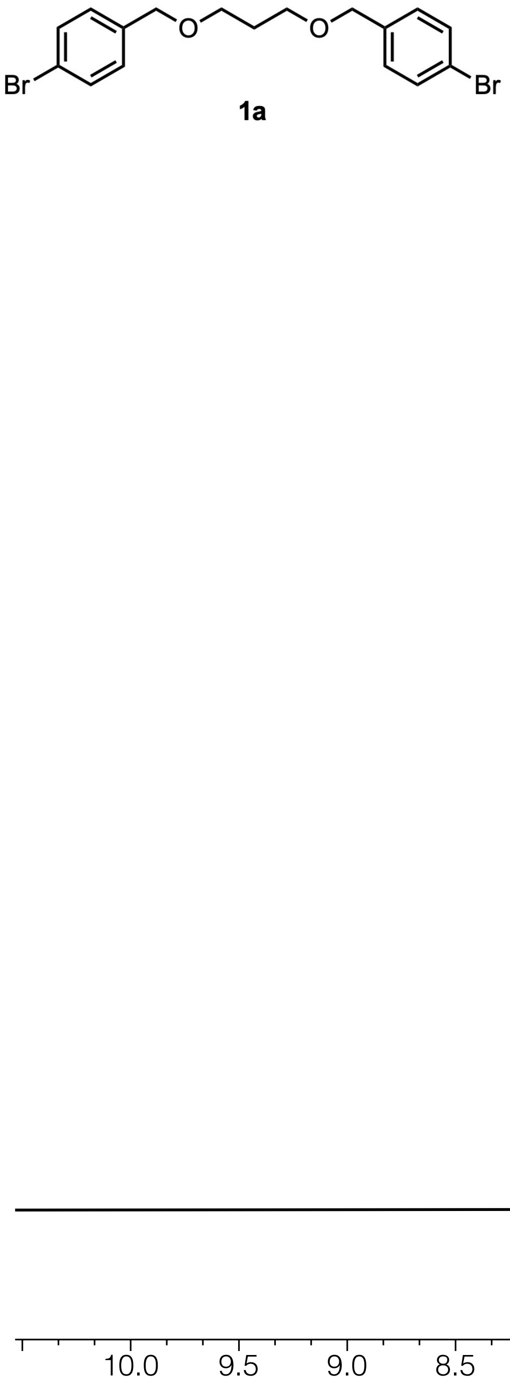

1a

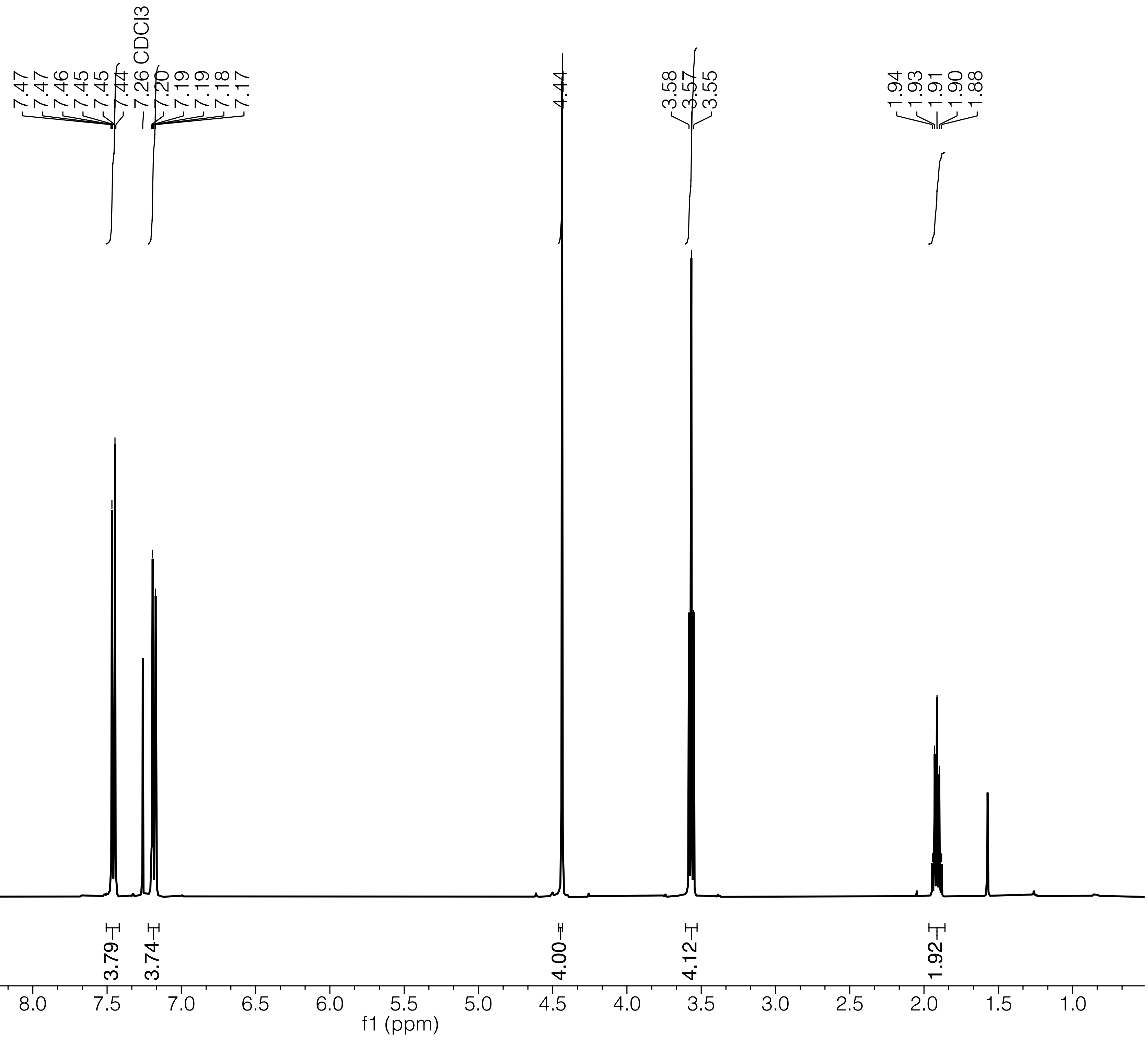

KS-4-45-fr
13C

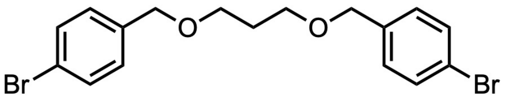

1a

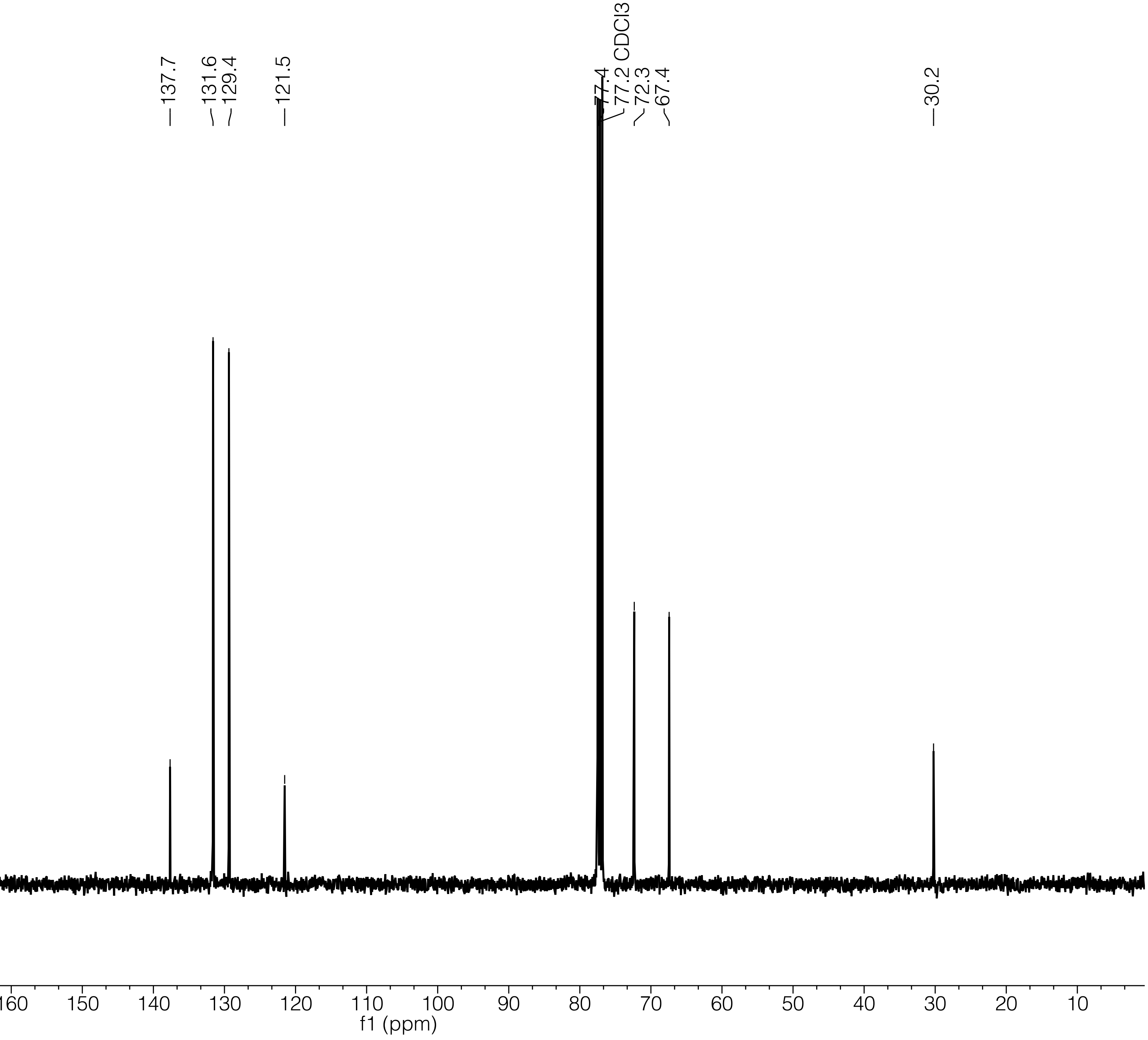

KS-4-44-fr
1H

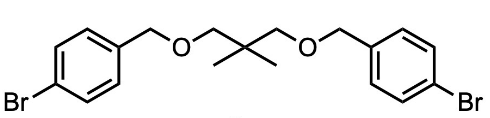

2a

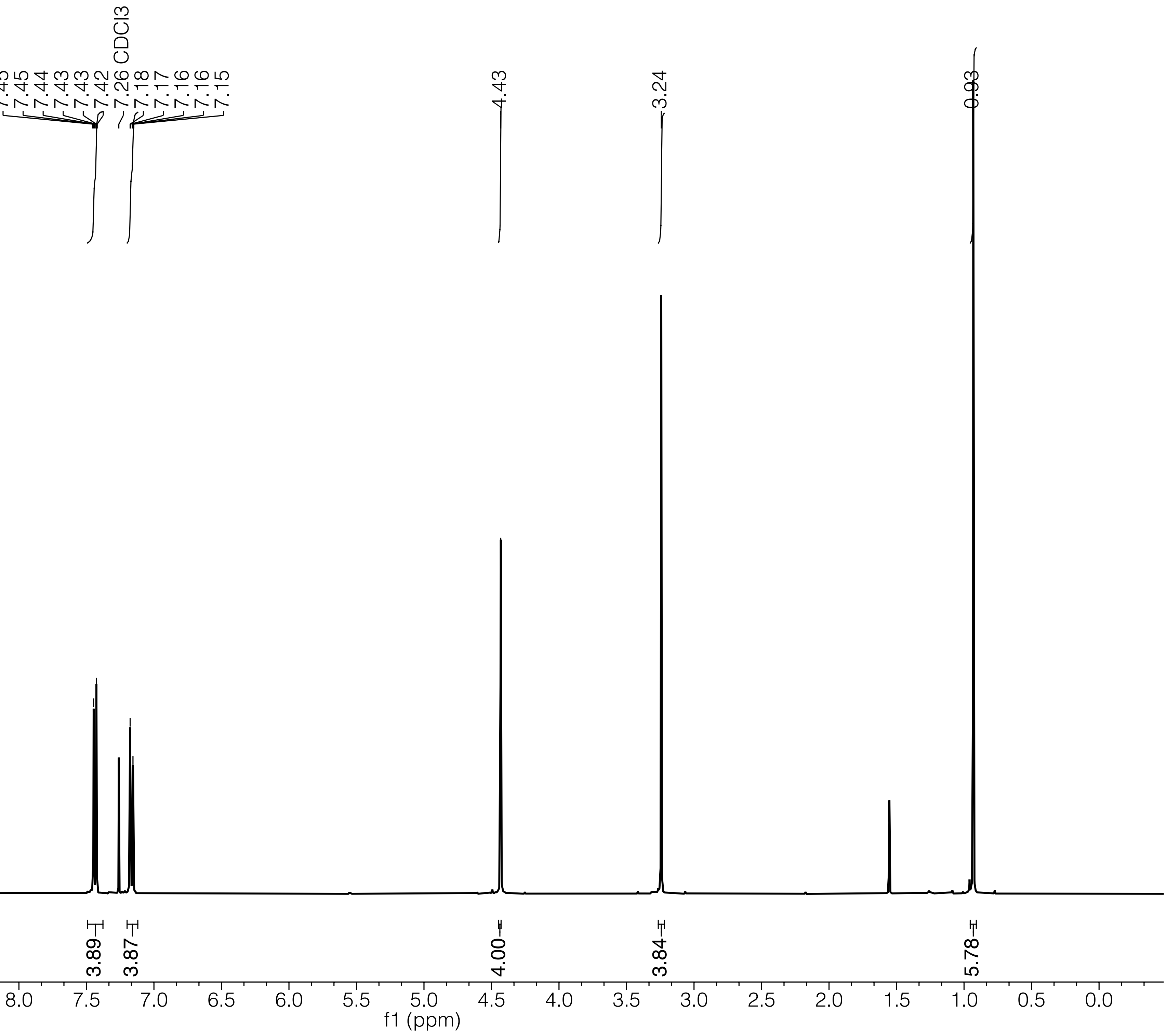

KS-4-44-fr
13C

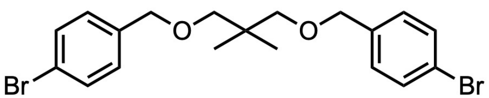

2a

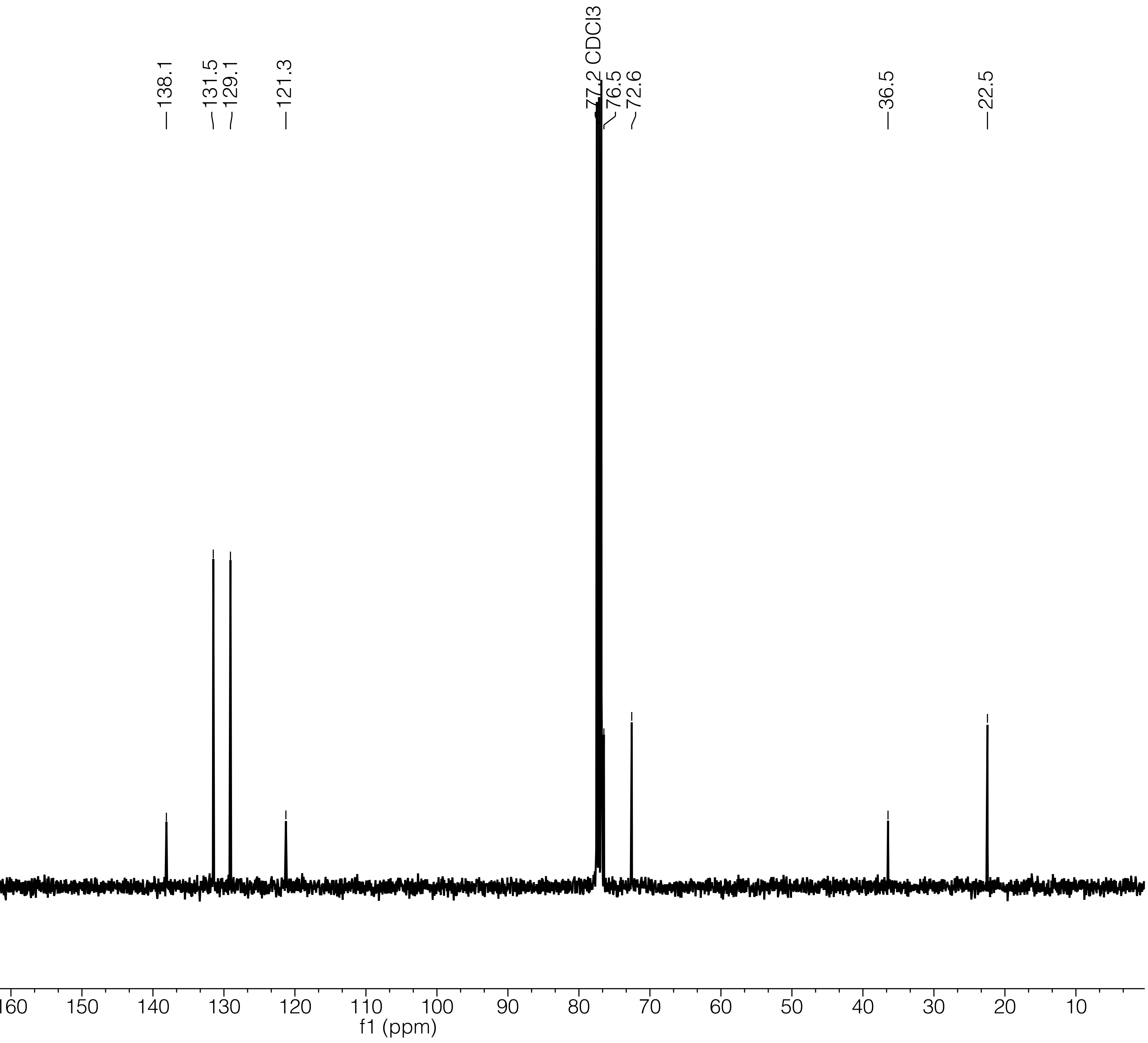

KS-4-42-fr
1H

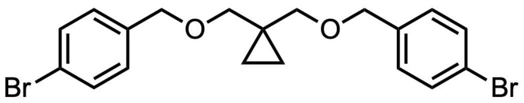

3a

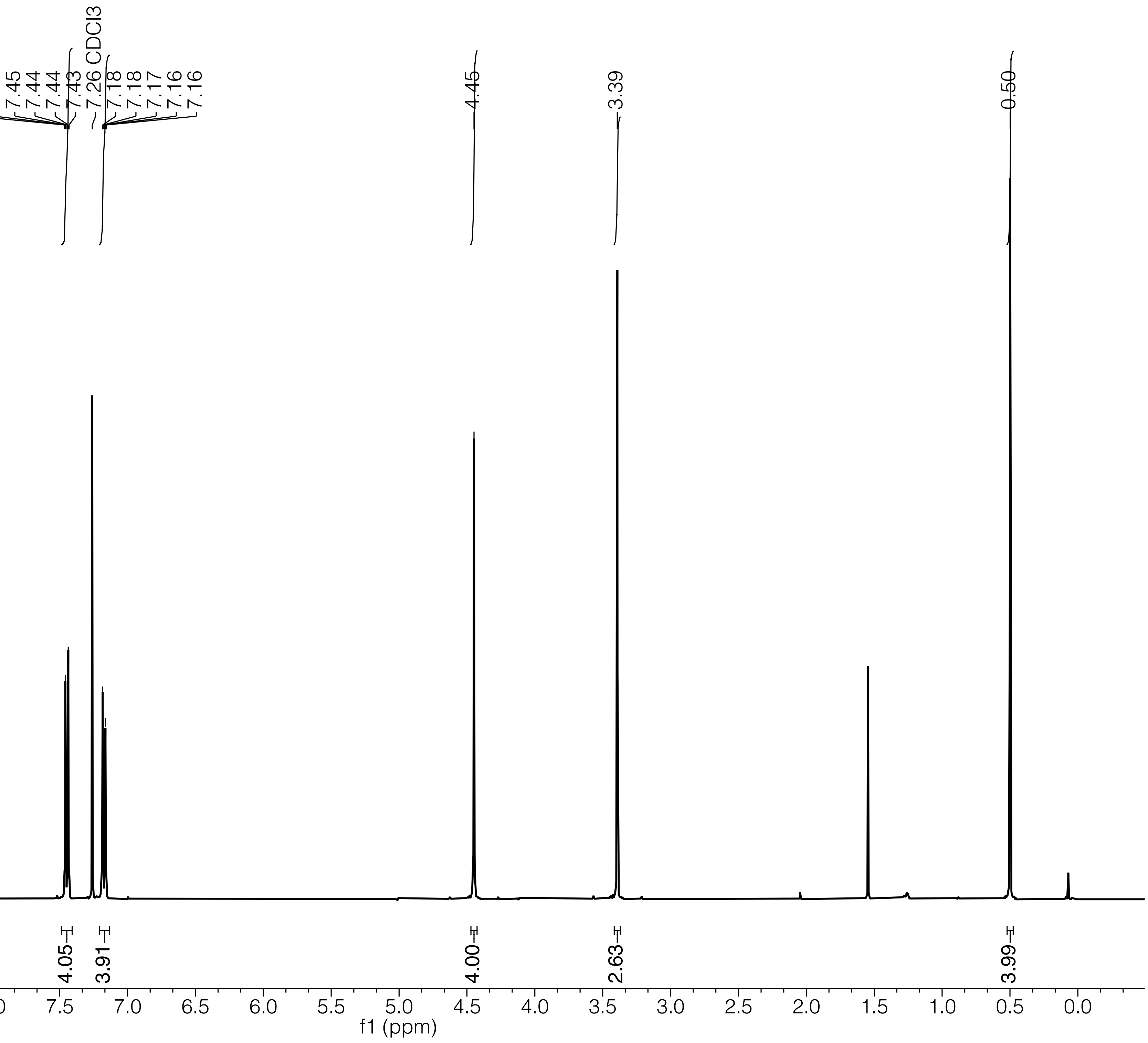

KS-4-42-fr
13C

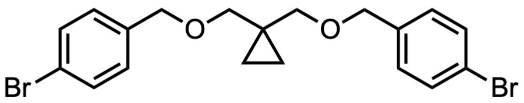

3a

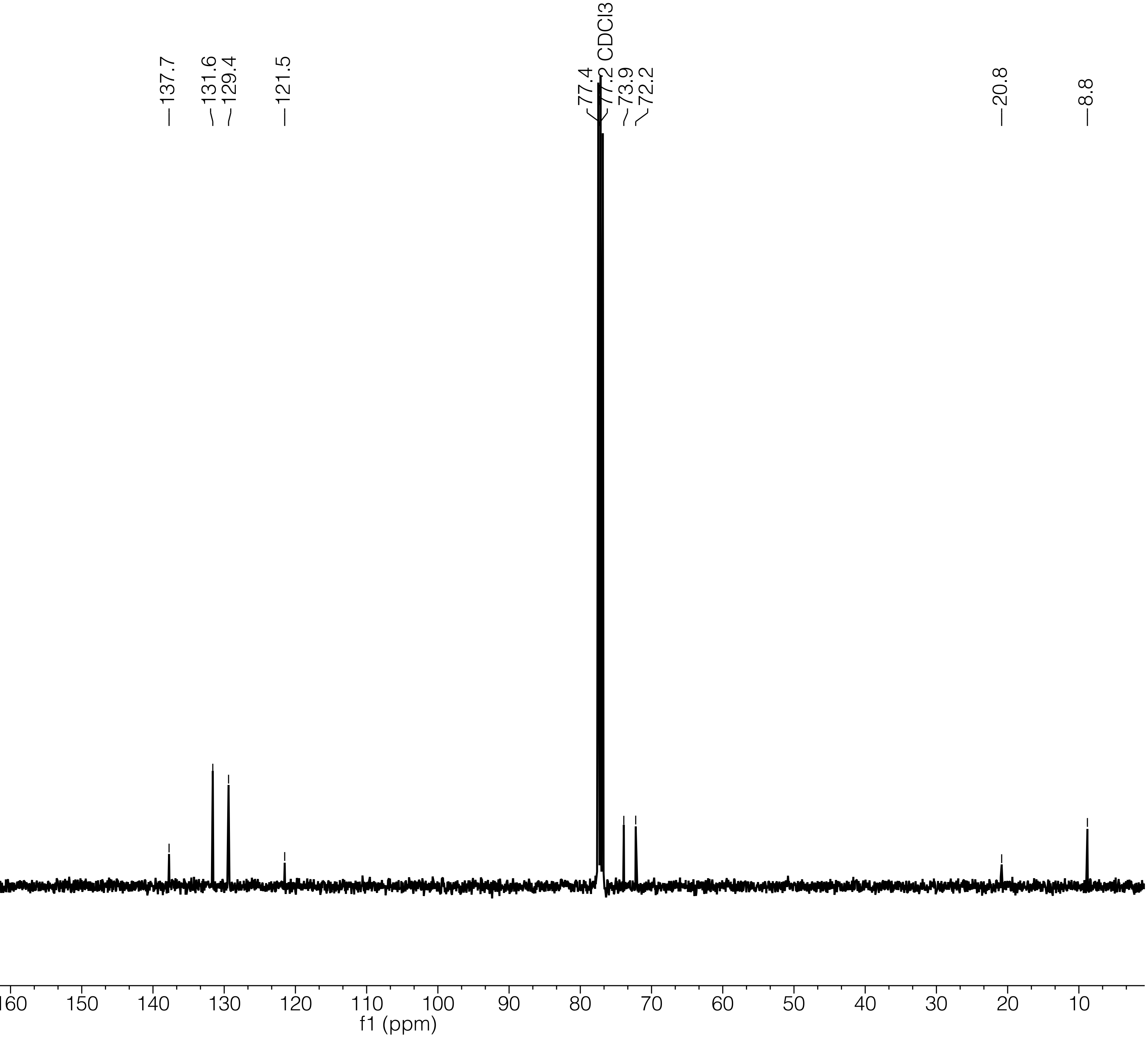

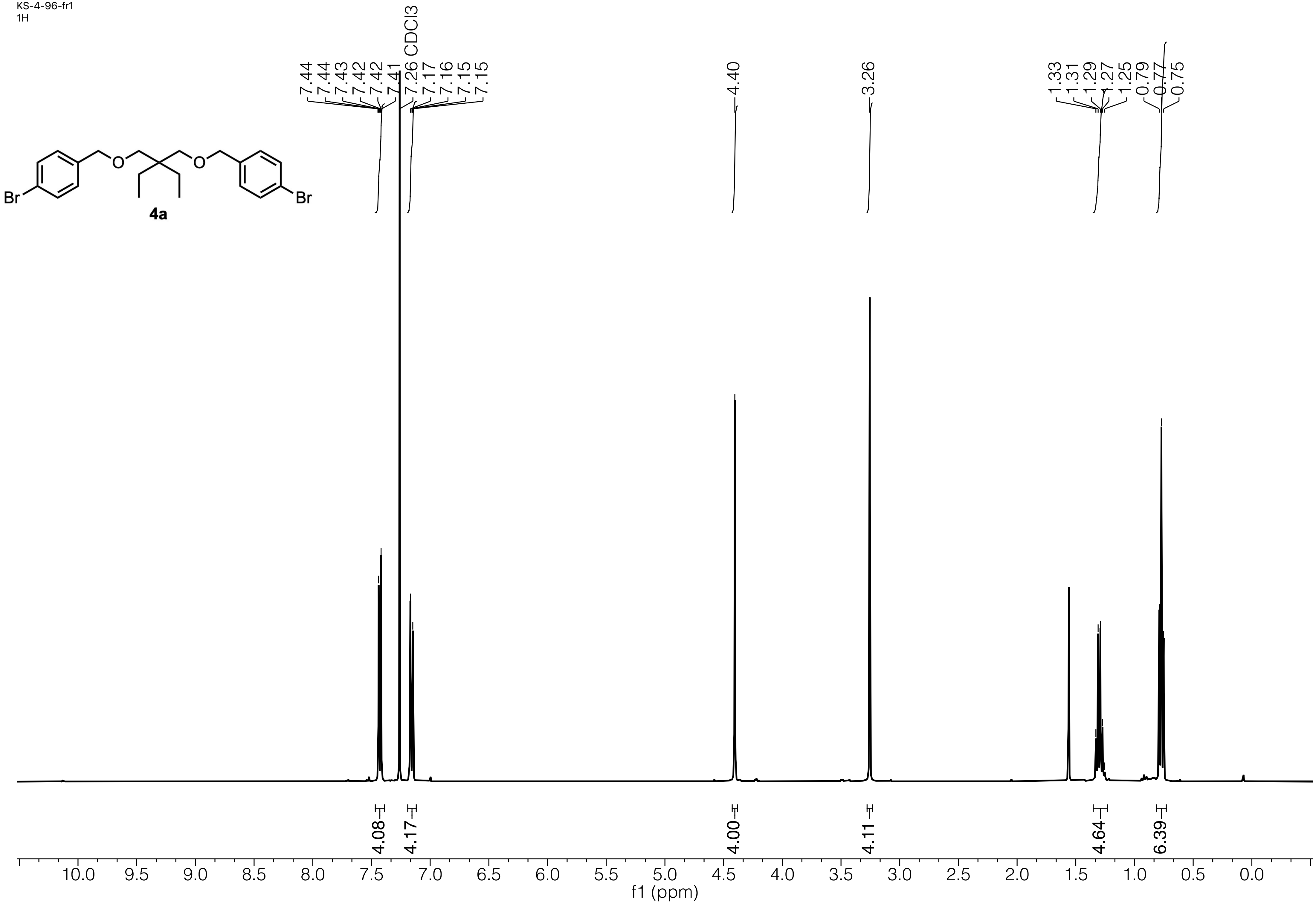
KS-4-96-fr1
1H
7.44
7.44
7.43
7.42
7.42
7.41
7.26 CDCl3
7.17
7.16
7.15
7.15
4.40
3.26
1.33
1.31
1.29
1.27
1.25
0.79
0.77
0.75
Br
O
O
Br
4a
4.08
4.17
4.00
4.11
4.64
6.39
10.0
9.5
9.0
8.5
8.0
7.5
7.0
6.5
6.0
5.5
5.0
4.5
4.0
3.5
3.0
2.5
2.0
1.5
1.0
0.5
0.0
f1 (ppm)

KS-4-96-13C
13C

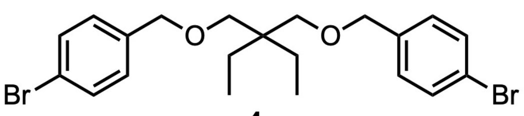

4a

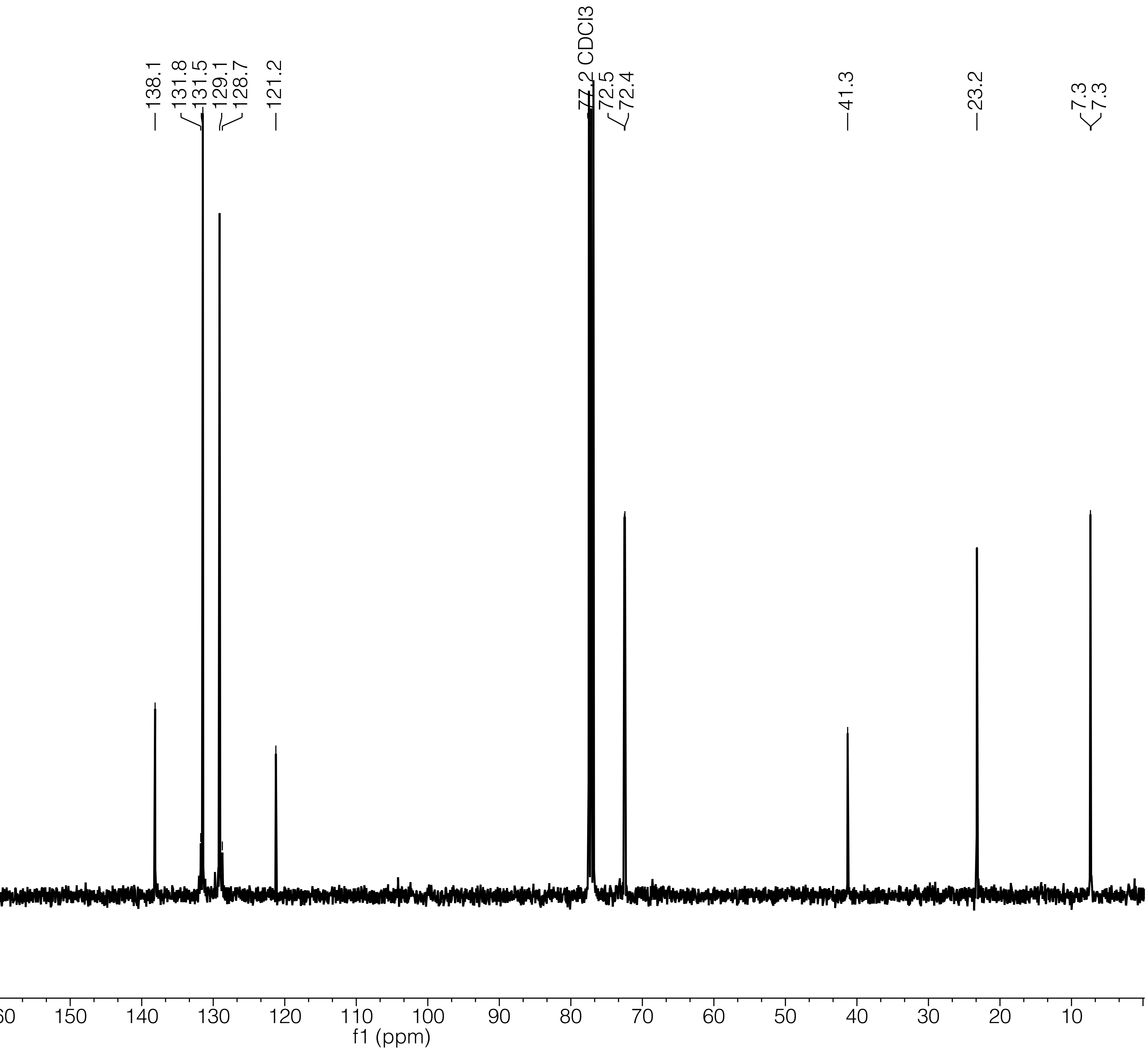

KS-5-7-fr4
1H

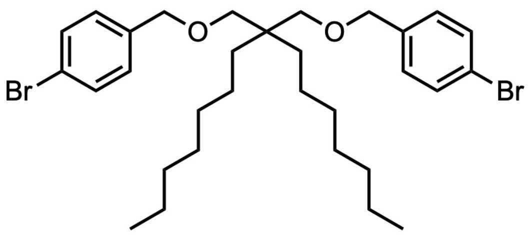

5a

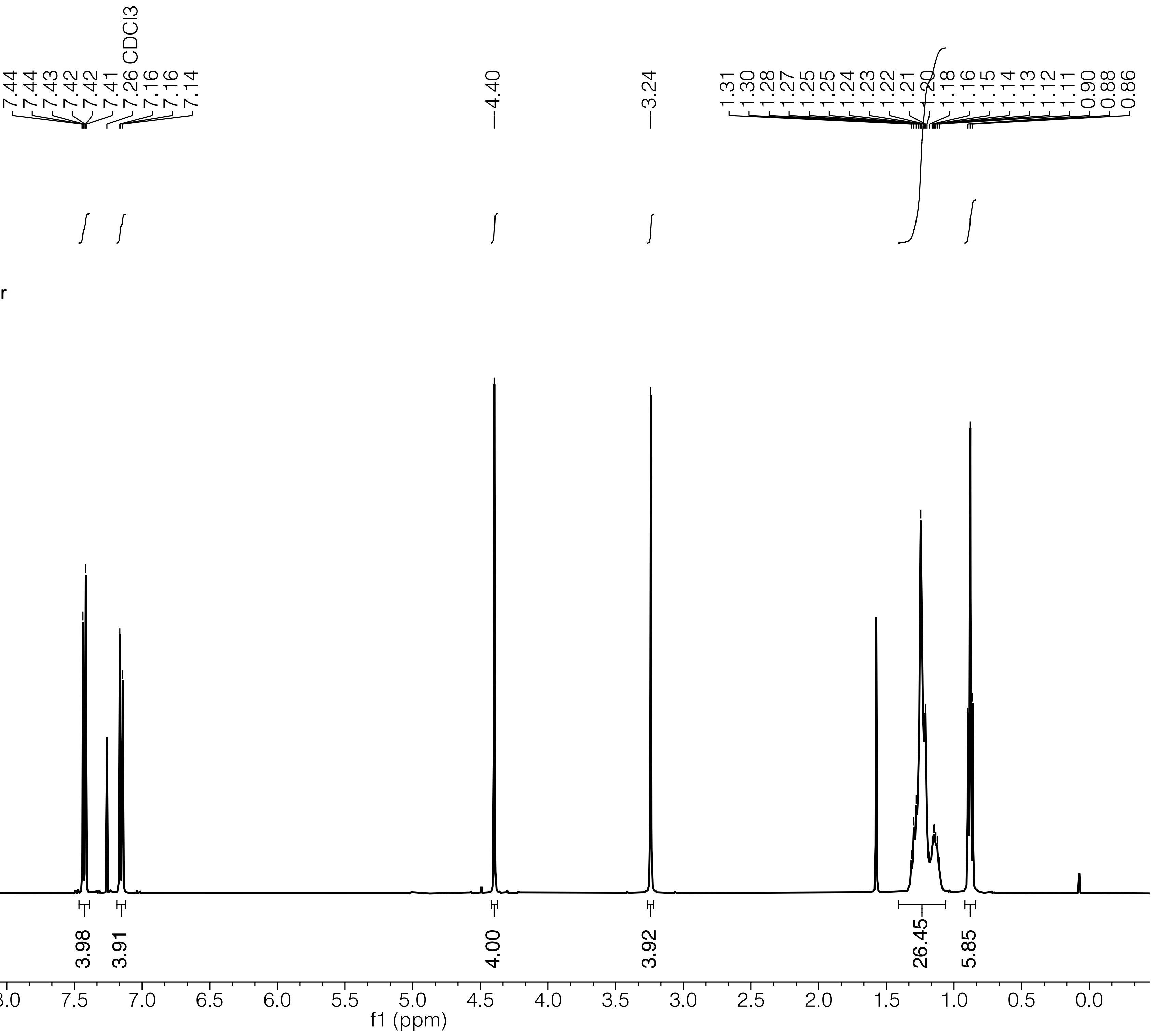

KS-5-7-fr4
13C

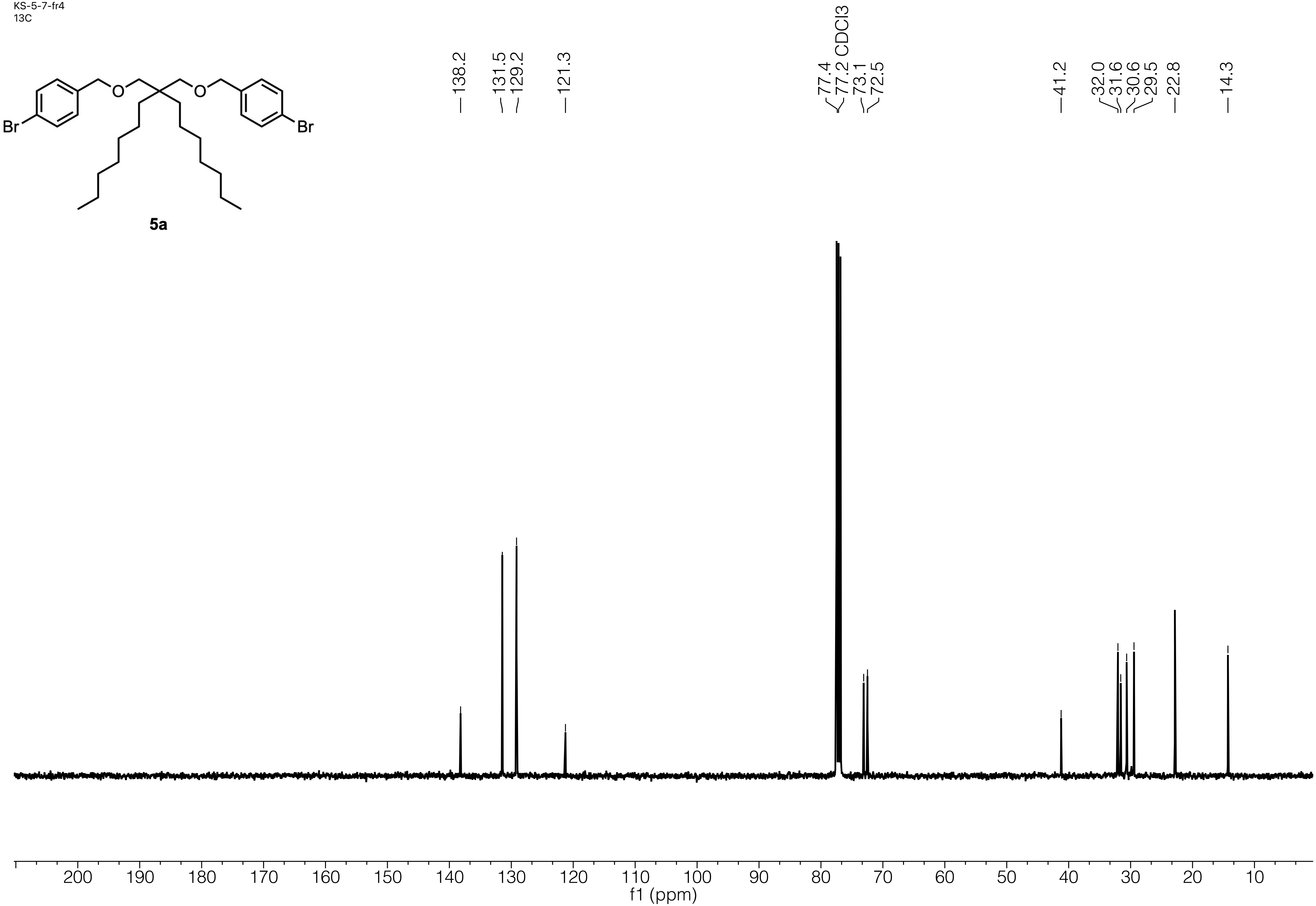

Br
O
O
Br
5a
138.2
131.5
129.2
121.3
77.4
77.2 CDCl3
73.1
72.5
41.2
32.0
31.6
30.6
29.5
22.8
14.3
200
190
180
170
160
150
140
130
120
110
100
90
80
70
60
50
40
30
20
10
f1 (ppm)

KS-5-8-Acetone-d6
1H

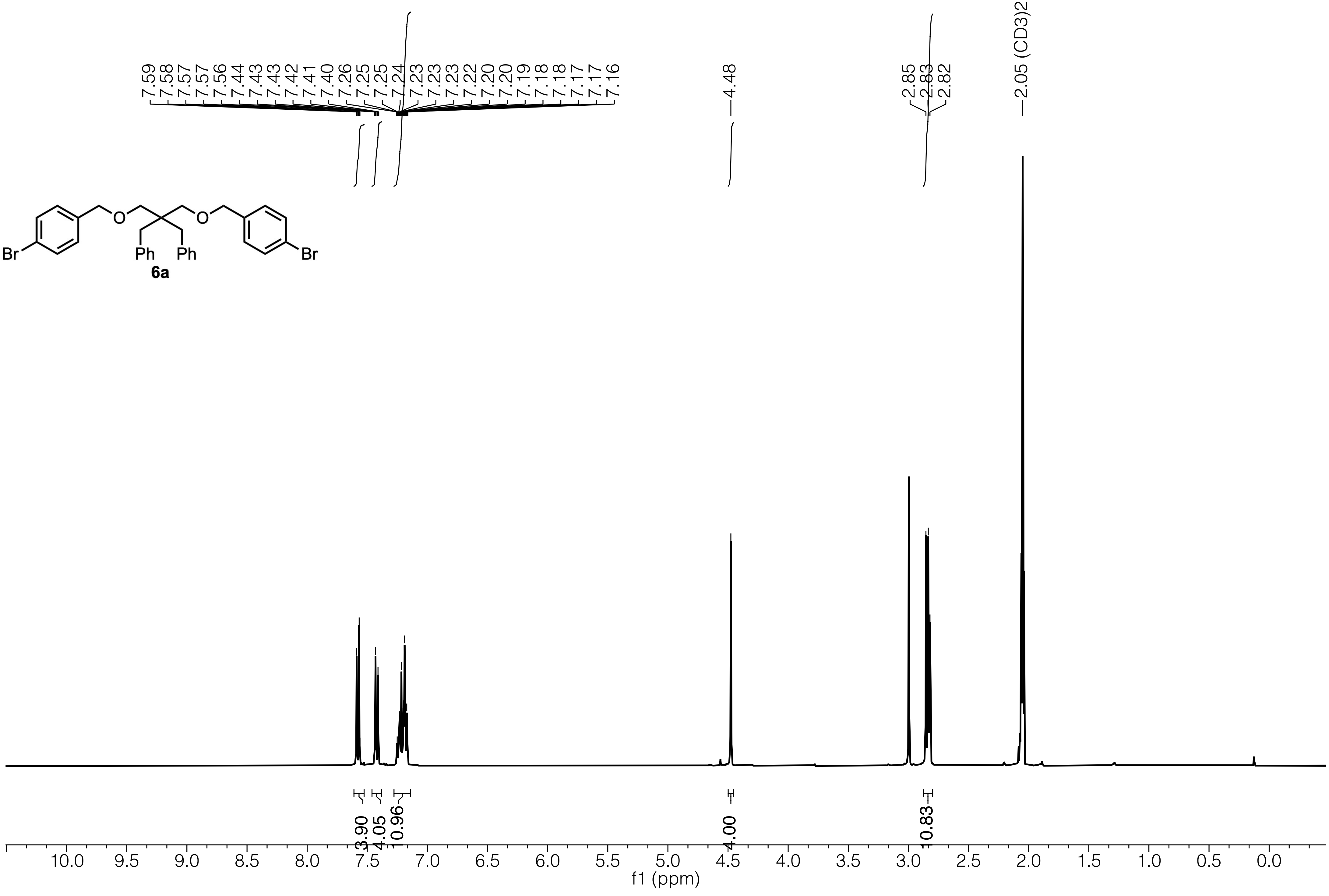

KS-5-8-fr1
13C

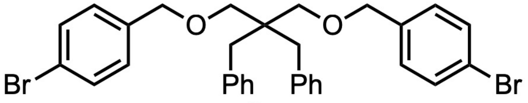

6a

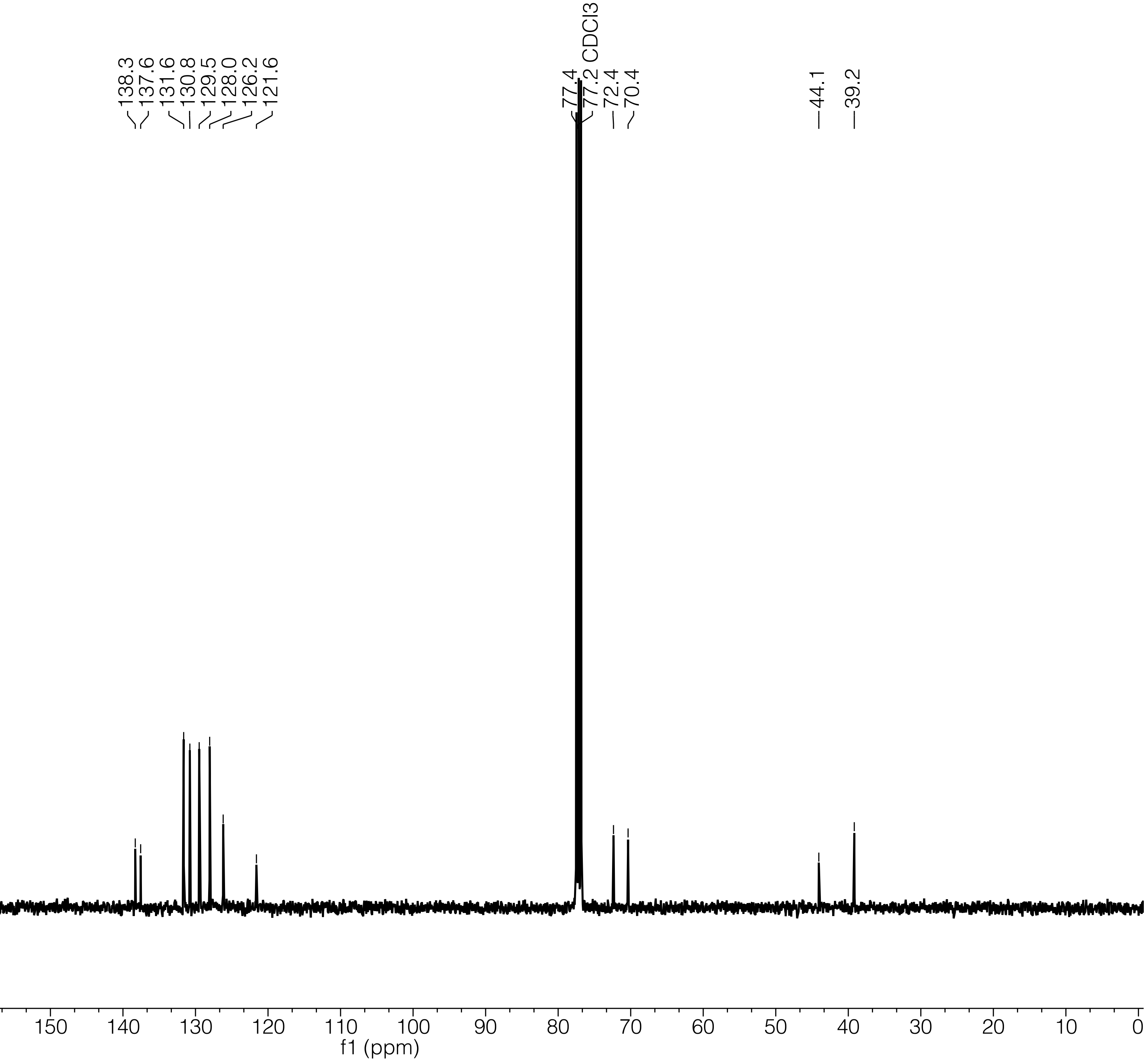

KS-4-55-fr
1H

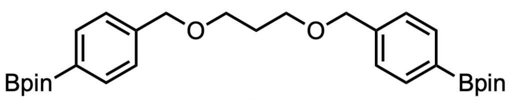

1b

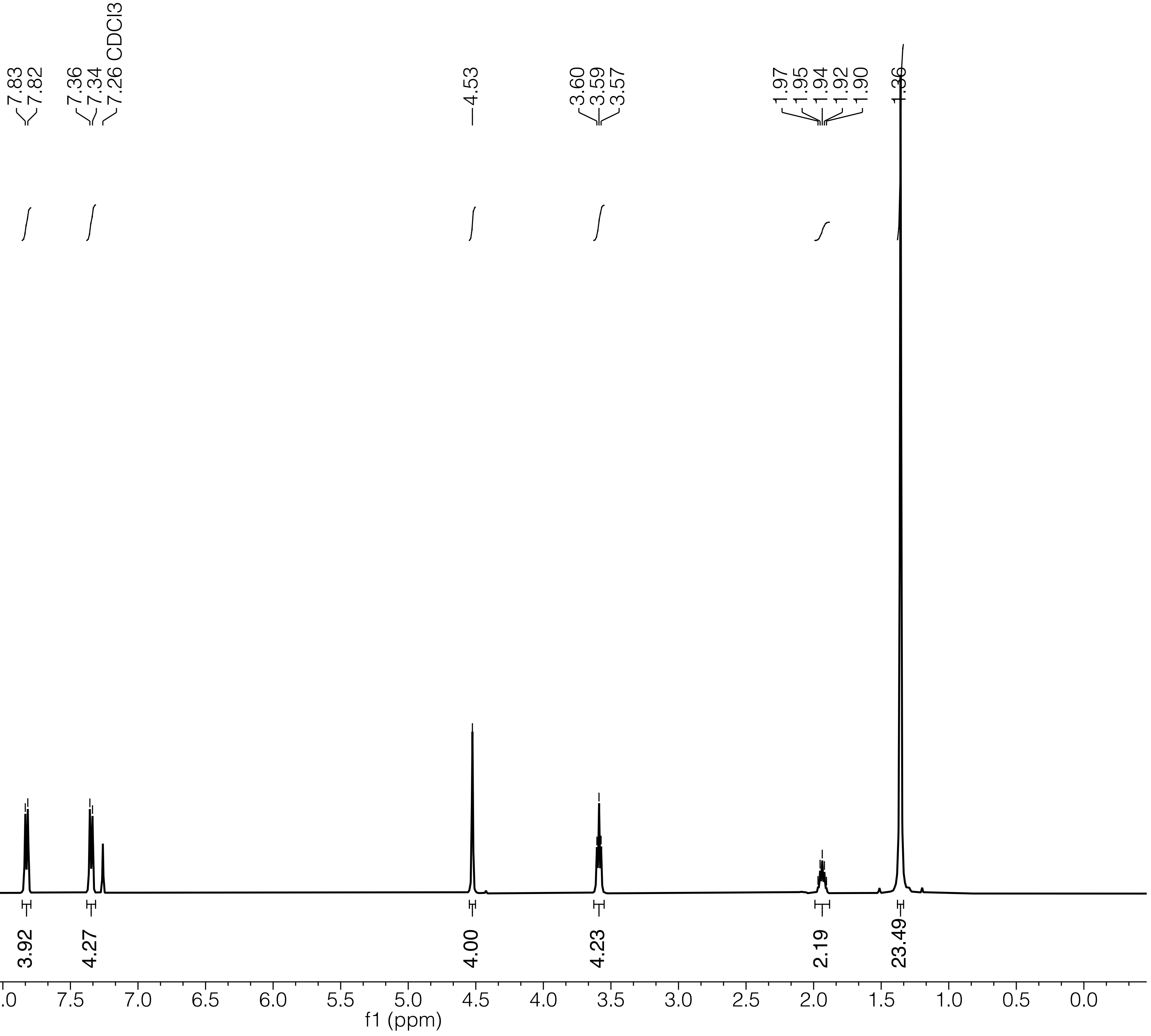

KS-4-55-fr
13C

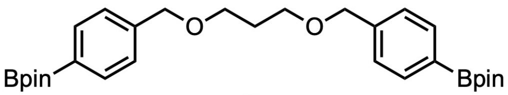

1b

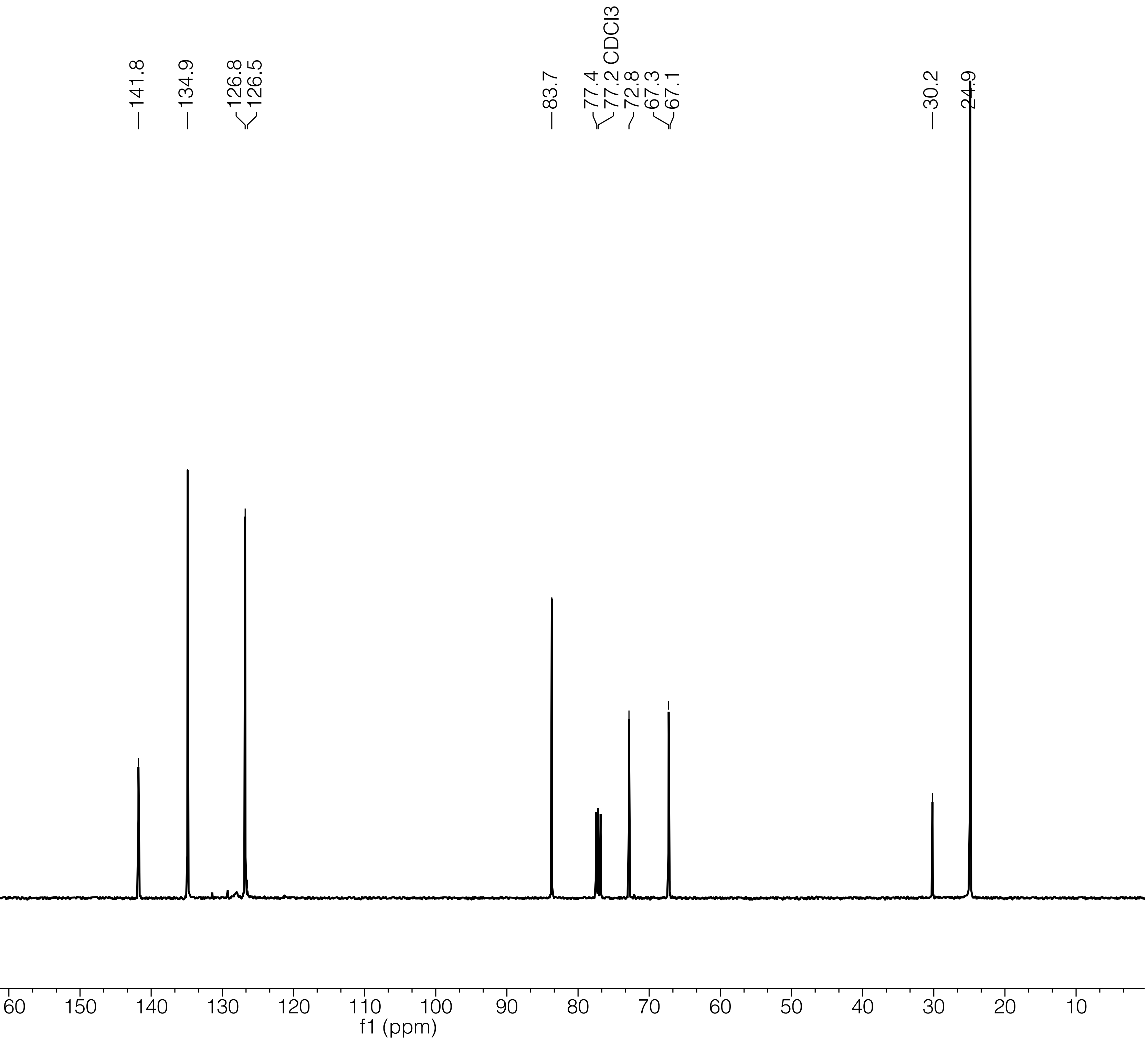

KS-4-55-11B
11B_No irr

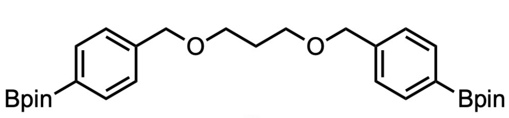

1b

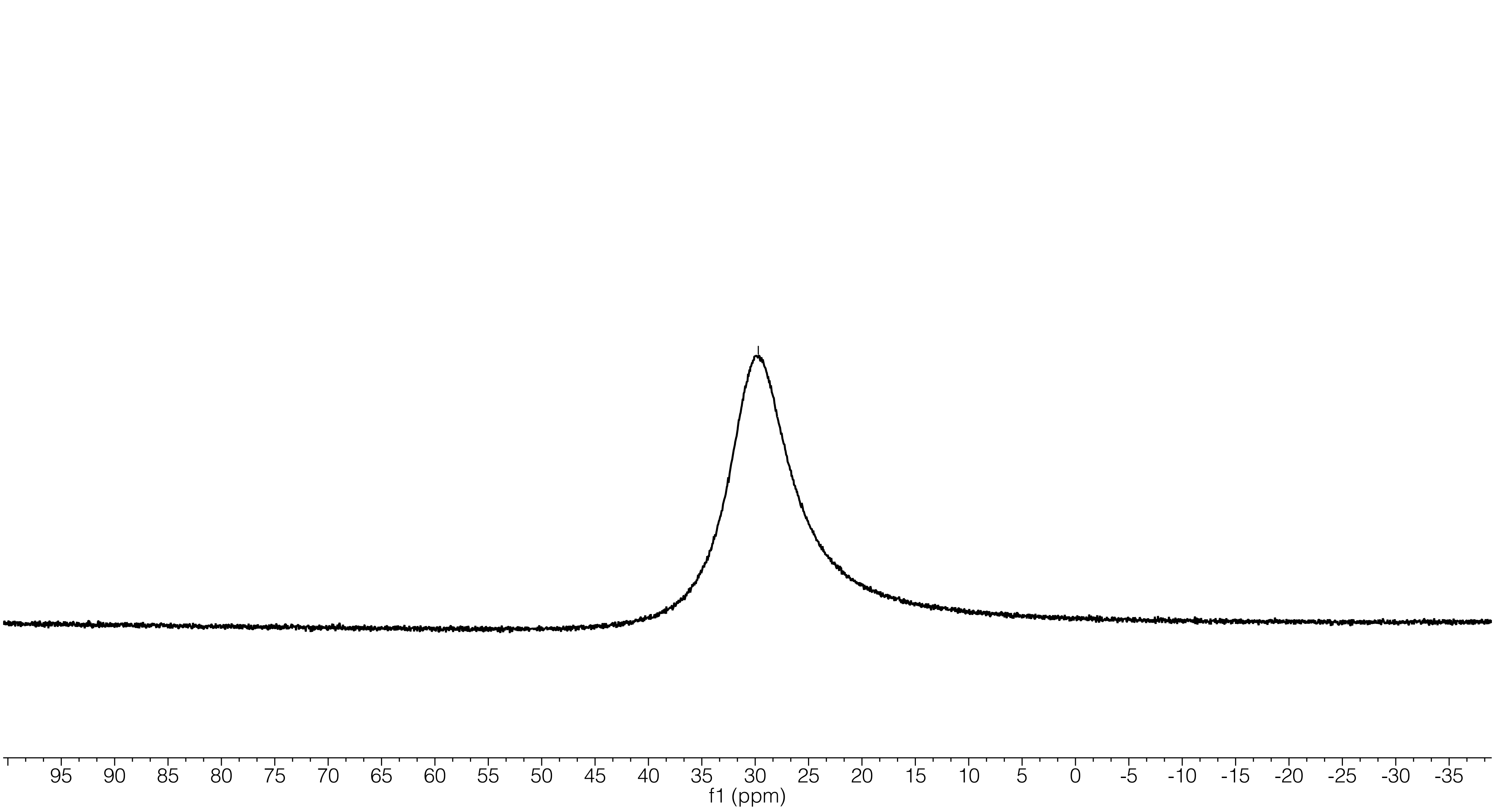

KS-4-54-fr
1H

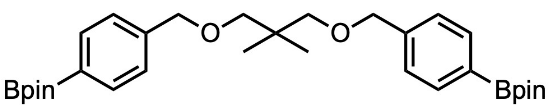

2b

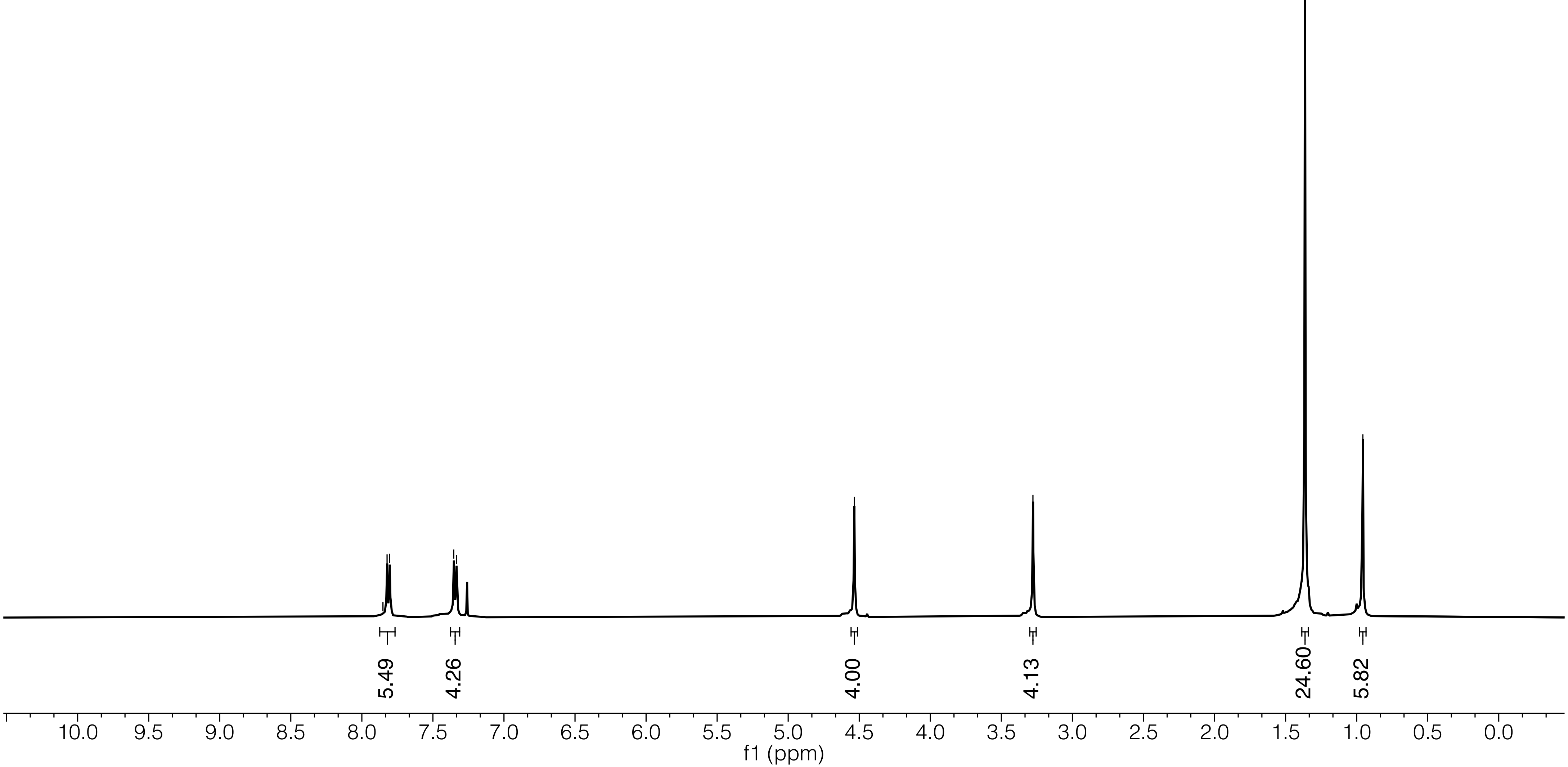

KS-4-54-fr
13C

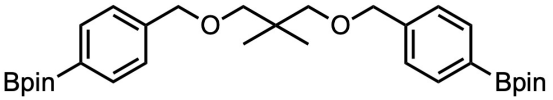

2b

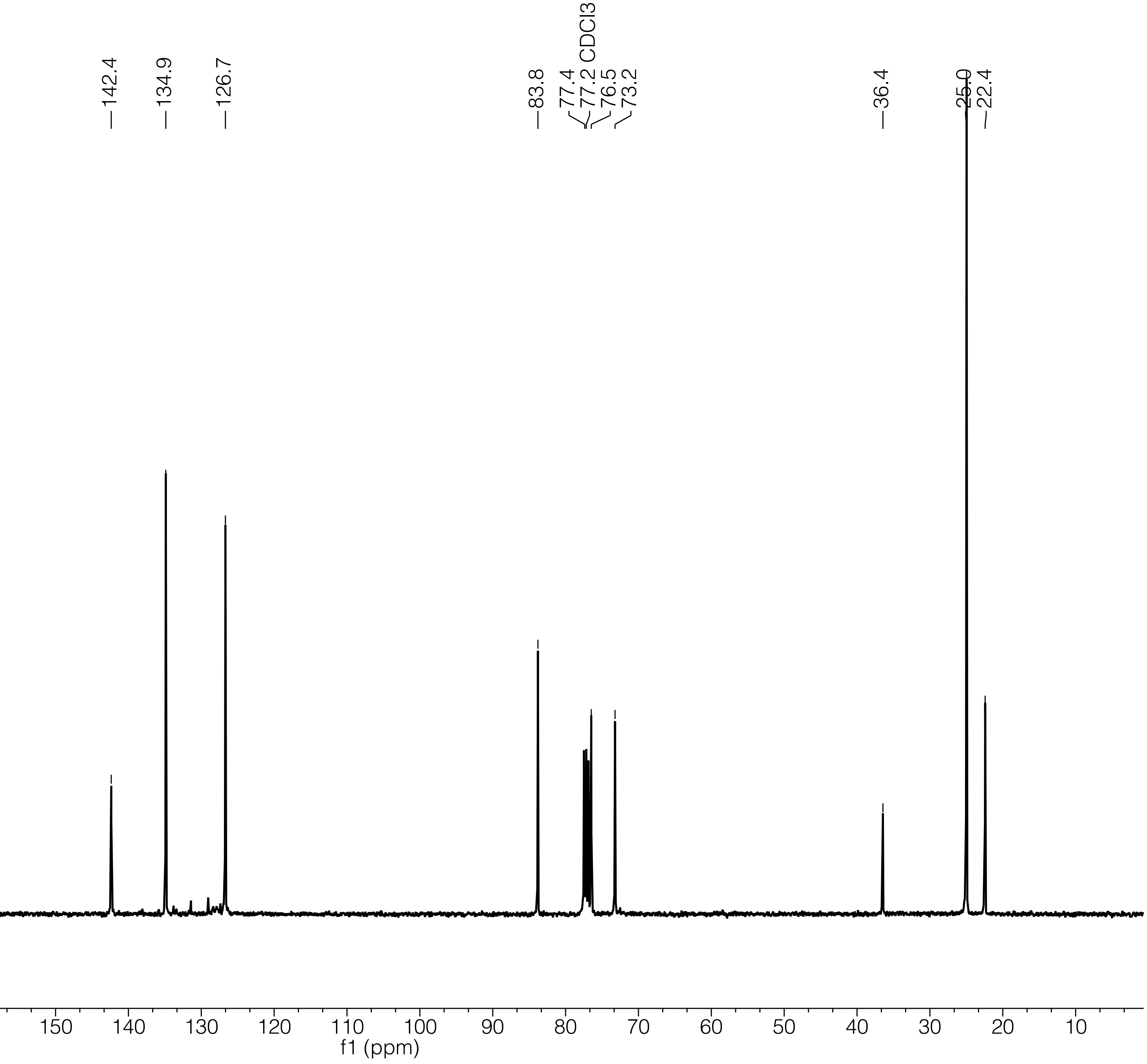

KS-4-54-11B
11B_No irr

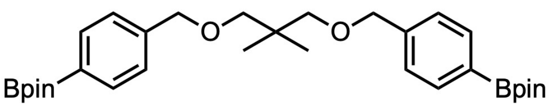

2b

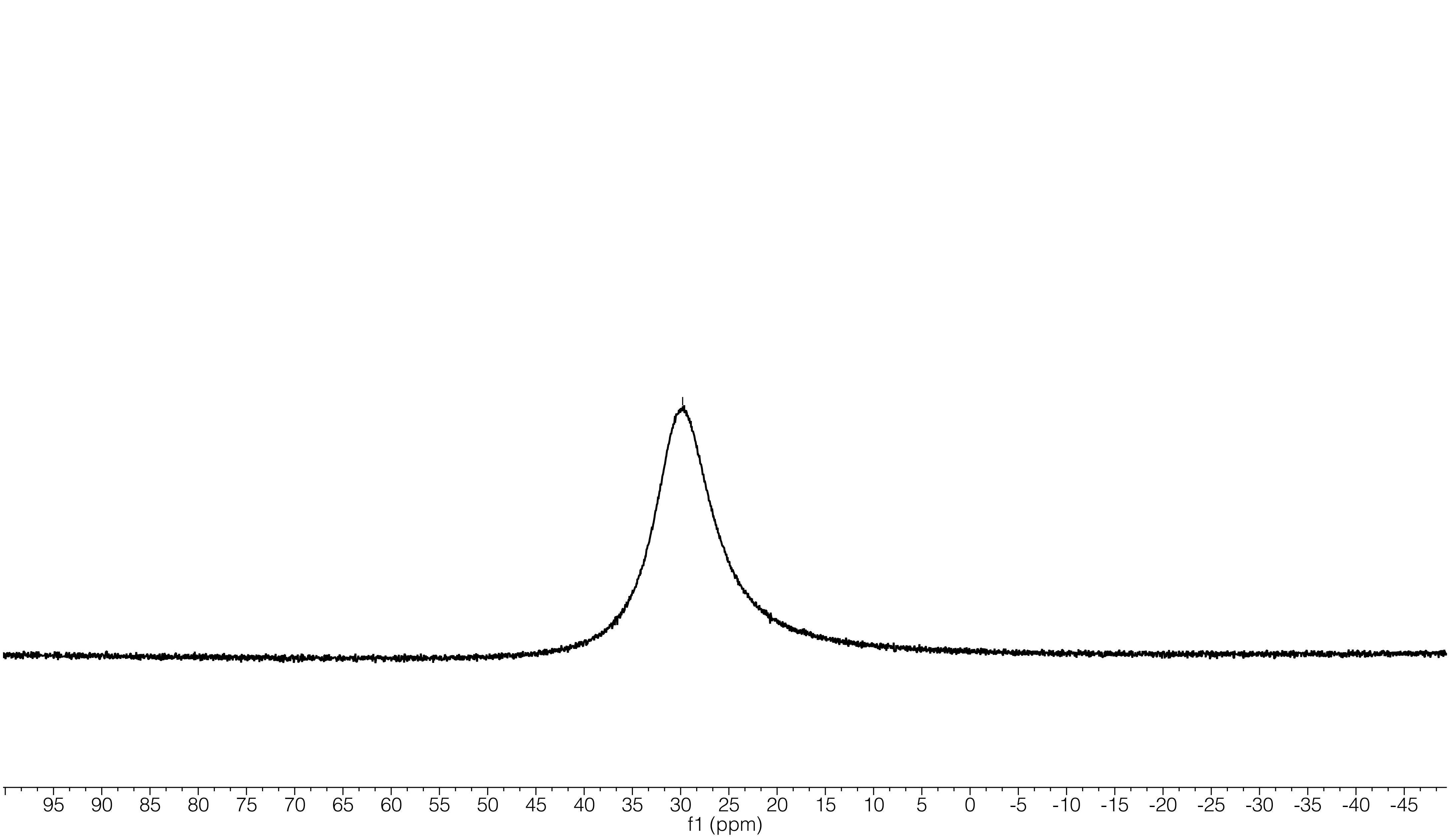

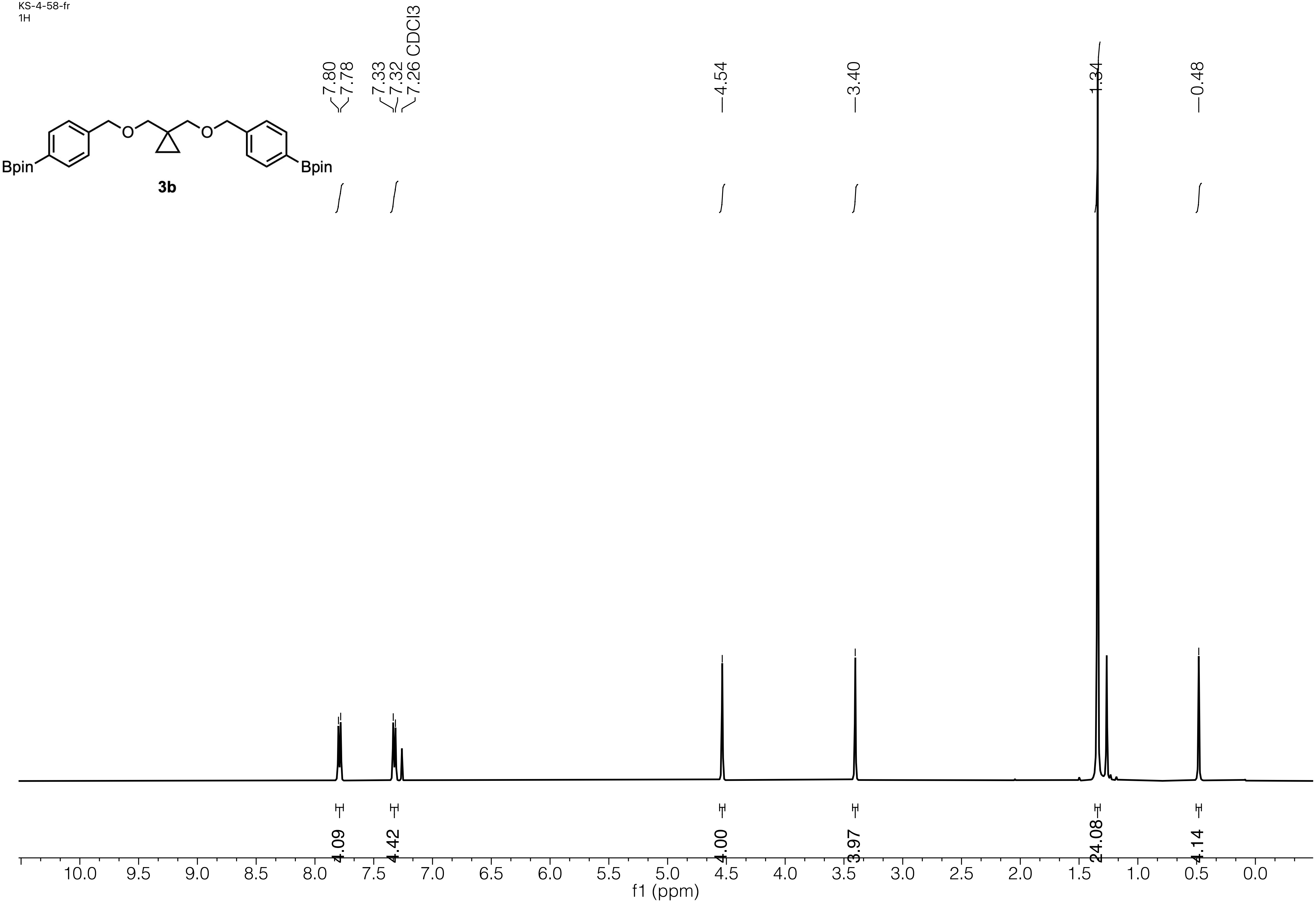
KS-4-58-fr
1H
7.80
7.78
7.33
7.32
7.26 CDCl3
4.54
3.40
1.34
0.48
Bpin
Bpin
O
O
3b
4.09
4.42
4.00
3.97
24.08
4.14
10.0 9.5 9.0 8.5 8.0 7.5 7.0 6.5 6.0 5.5 5.0 4.5 4.0 3.5 3.0 2.5 2.0 1.5 1.0 0.5 0.0
f1 (ppm)

KS-4-58-fr
13C

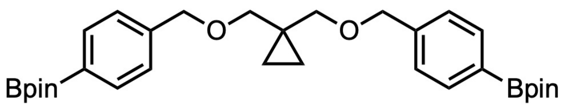

3b

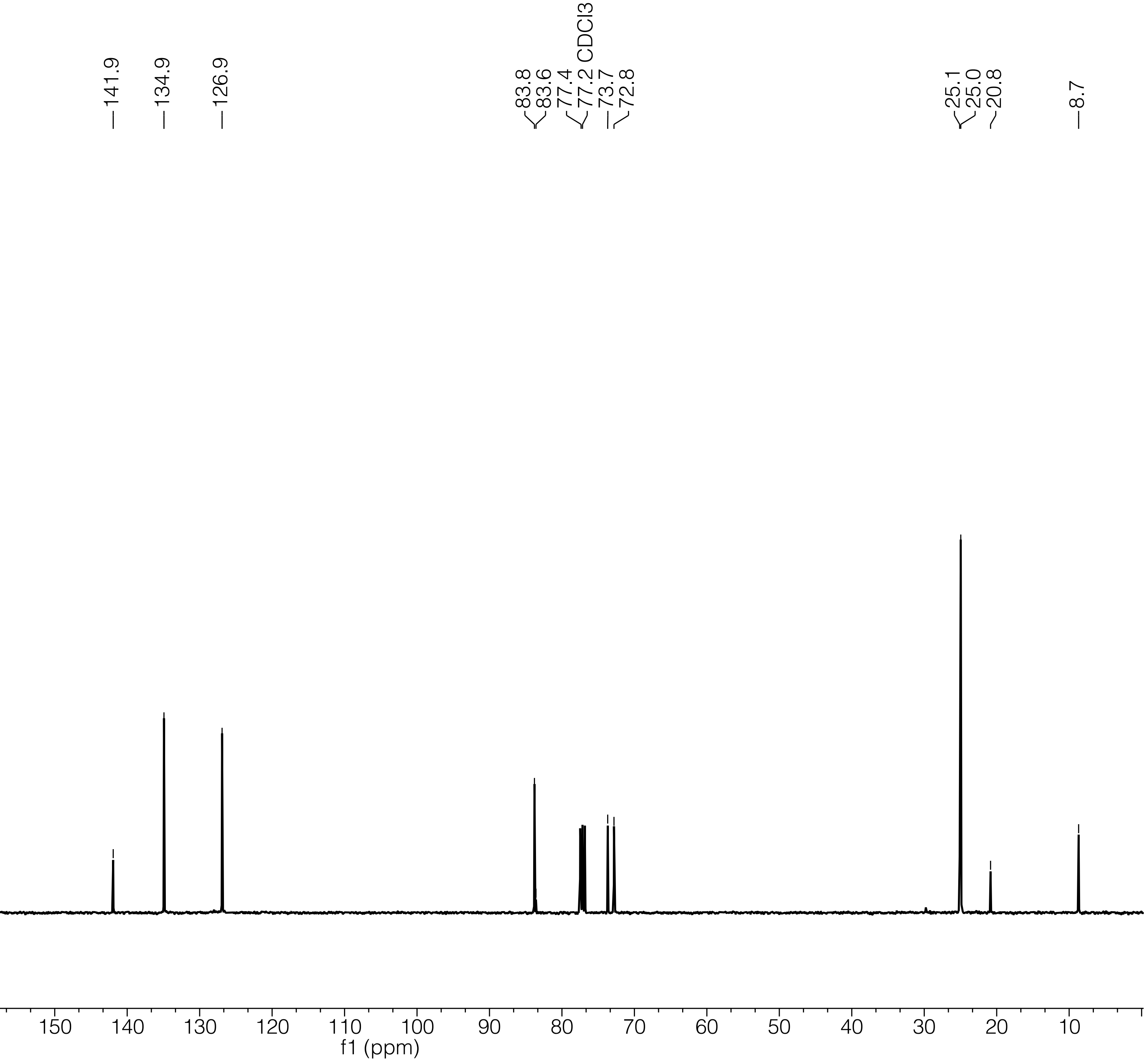

KS-4-58-E11B
11B_No irr

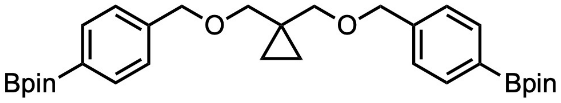

3b

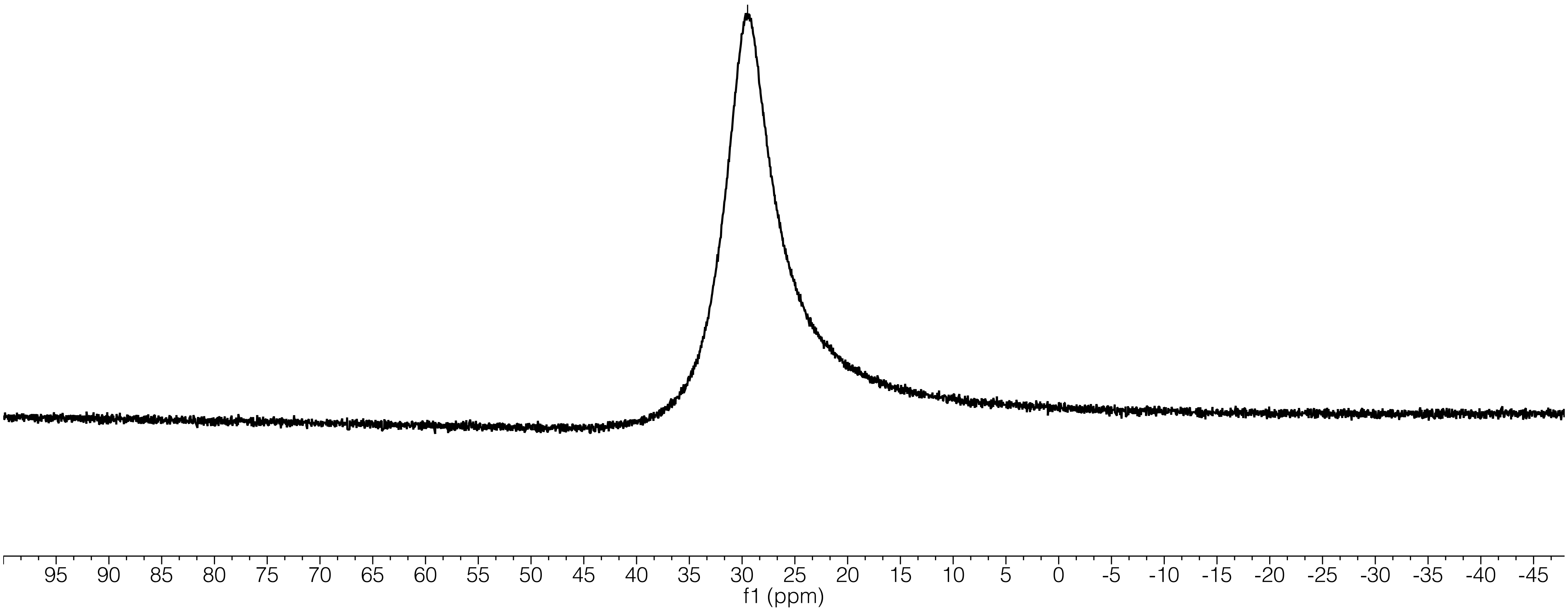

KS-5-1-fr2
1H

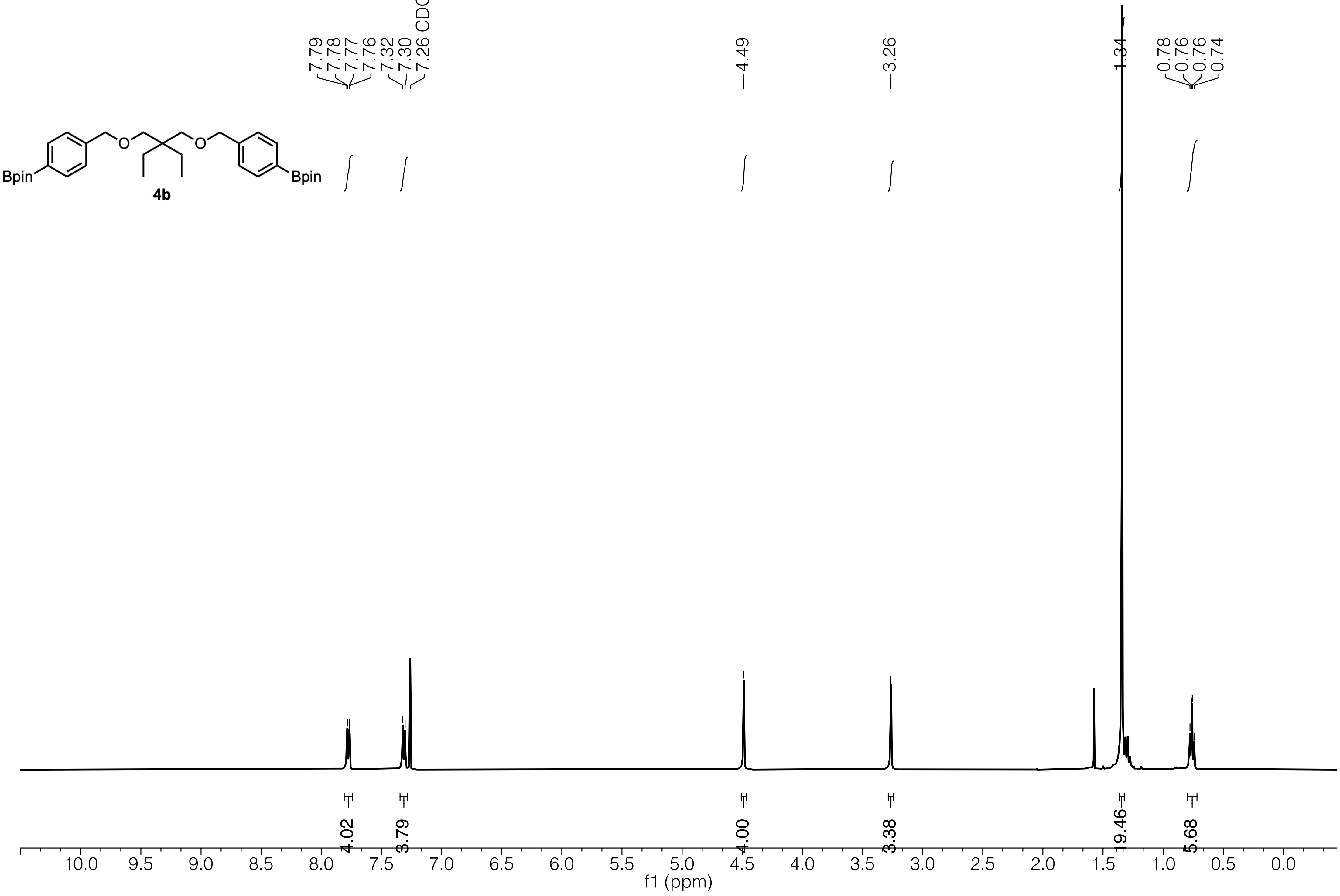
7.79
7.78
7.77
7.76
7.32
7.30
7.26 CDCl3
4.49
3.26
1.34
0.78
0.76
0.76
0.74
Bpin
O
O
Bpin
4b
4.02
3.79
4.00
3.38
19.46
5.68
10.0
9.5
9.0
8.5
8.0
7.5
7.0
6.5
6.0
5.5
5.0
4.5
4.0
3.5
3.0
2.5
2.0
1.5
1.0
0.5
0.0
f1 (ppm)

KS-5-1
13C

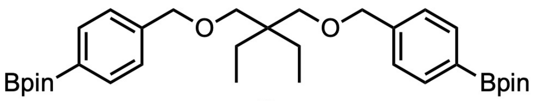

4b

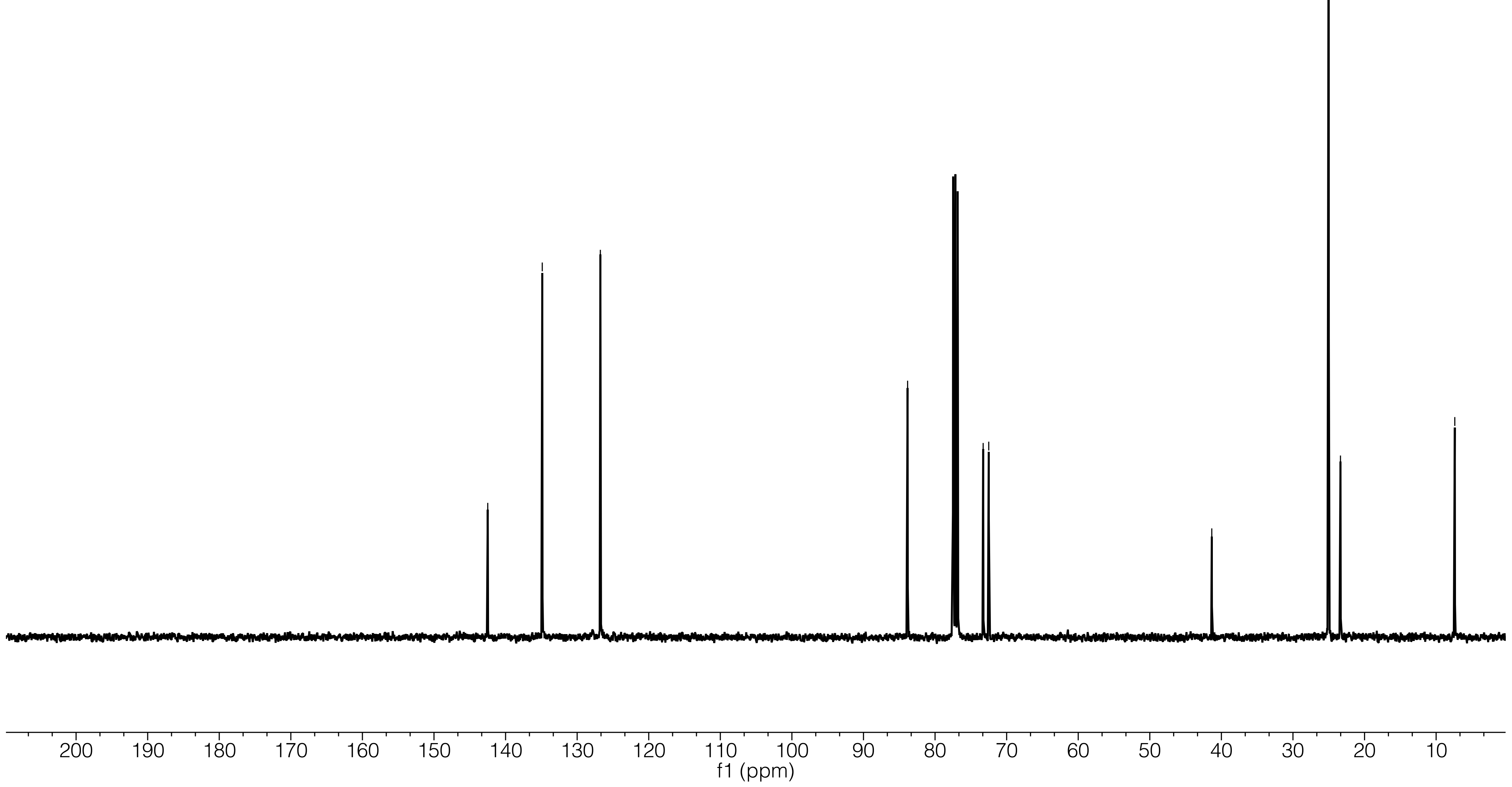
142.5
134.9
126.8
83.8
77.4
77.2 CDCl3
73.3
72.5
41.3
25.0
23.4
7.4
200 190 180 170 160 150 140 130 120 110 100 90 80 70 60 50 40 30 20 10
f1 (ppm)

KS-5-1
11B_No irr

—31.37

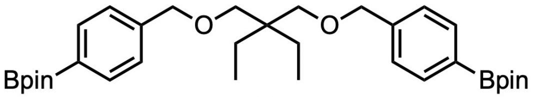

4b

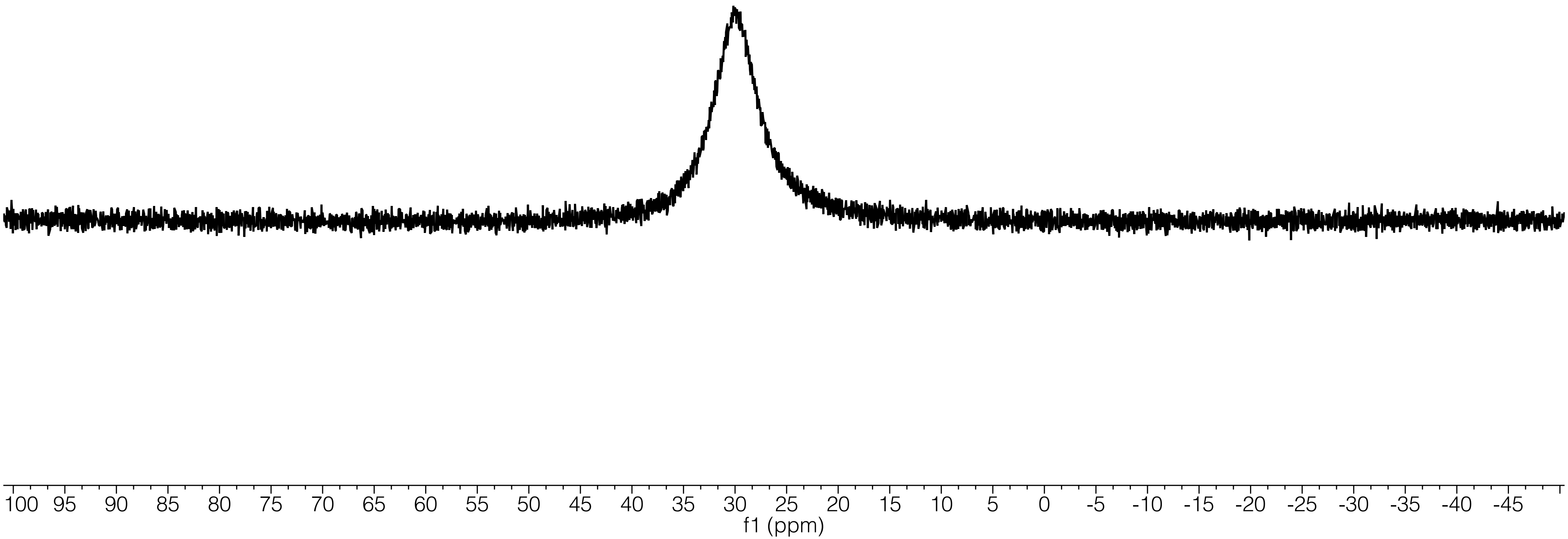

KS-5-11-fr1
1H

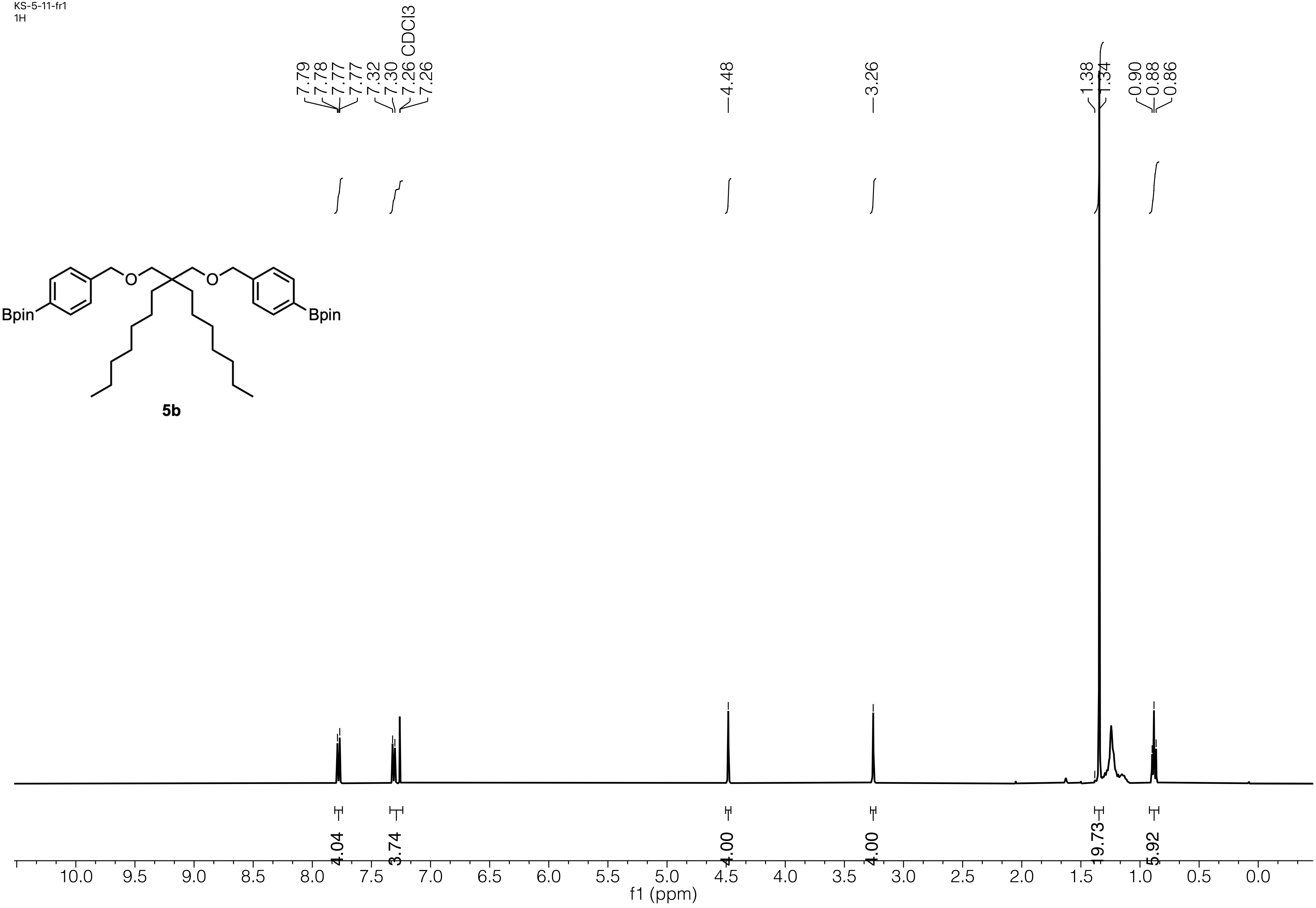

5b

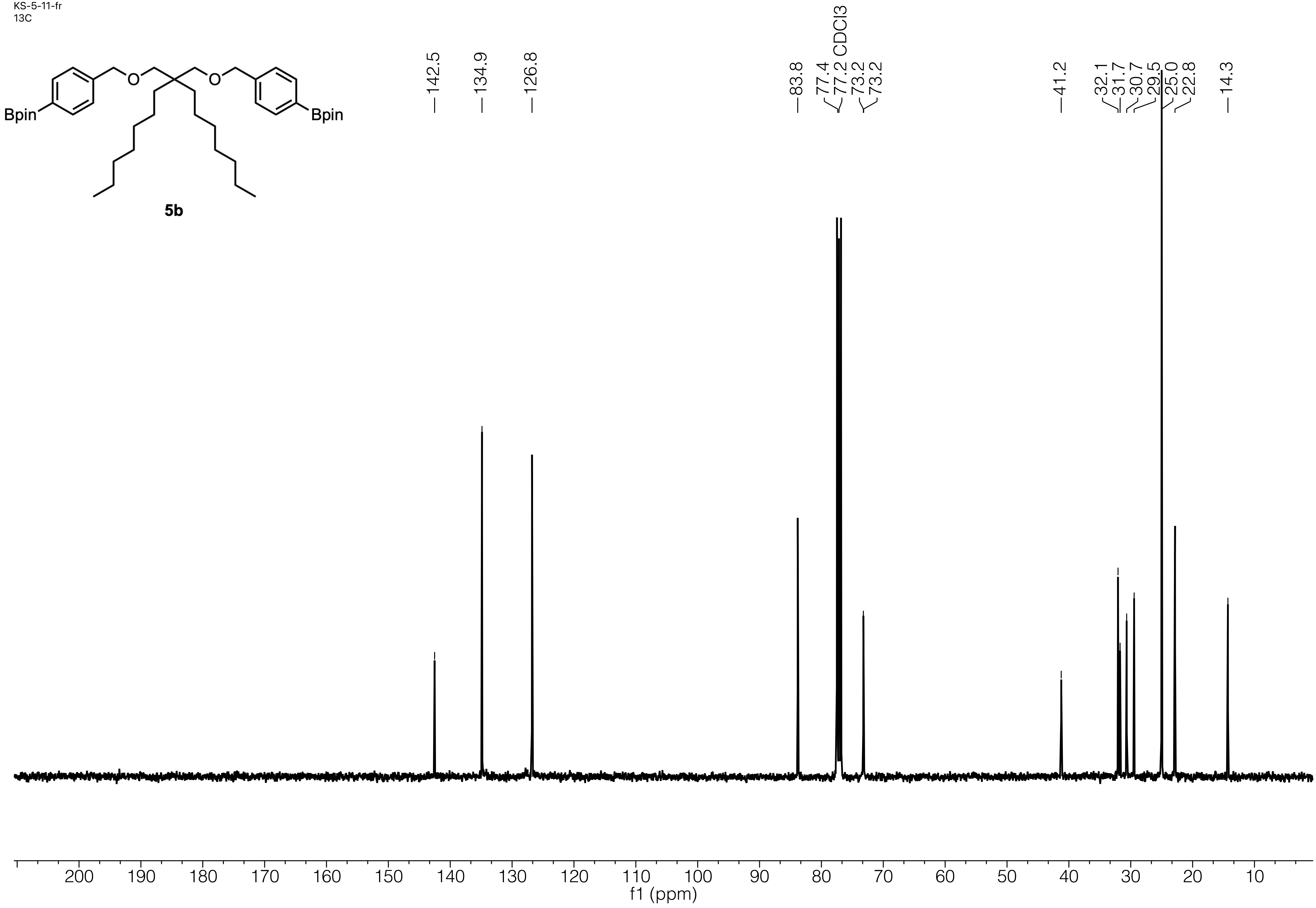
KS-5-11-fr
13C
Bpin
O
O
Bpin
5b
142.5
134.9
126.8
83.8
77.4
77.2 CDCl3
73.2
73.2
41.2
32.1
31.7
30.7
29.5
25.0
22.8
14.3
200 190 180 170 160 150 140 130 120 110 100 90 80 70 60 50 40 30 20 10
f1 (ppm)

KS-5-11-fr
11B_No irr

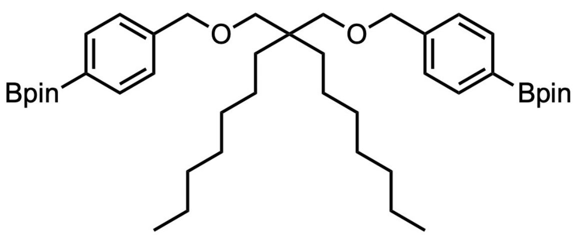

5b

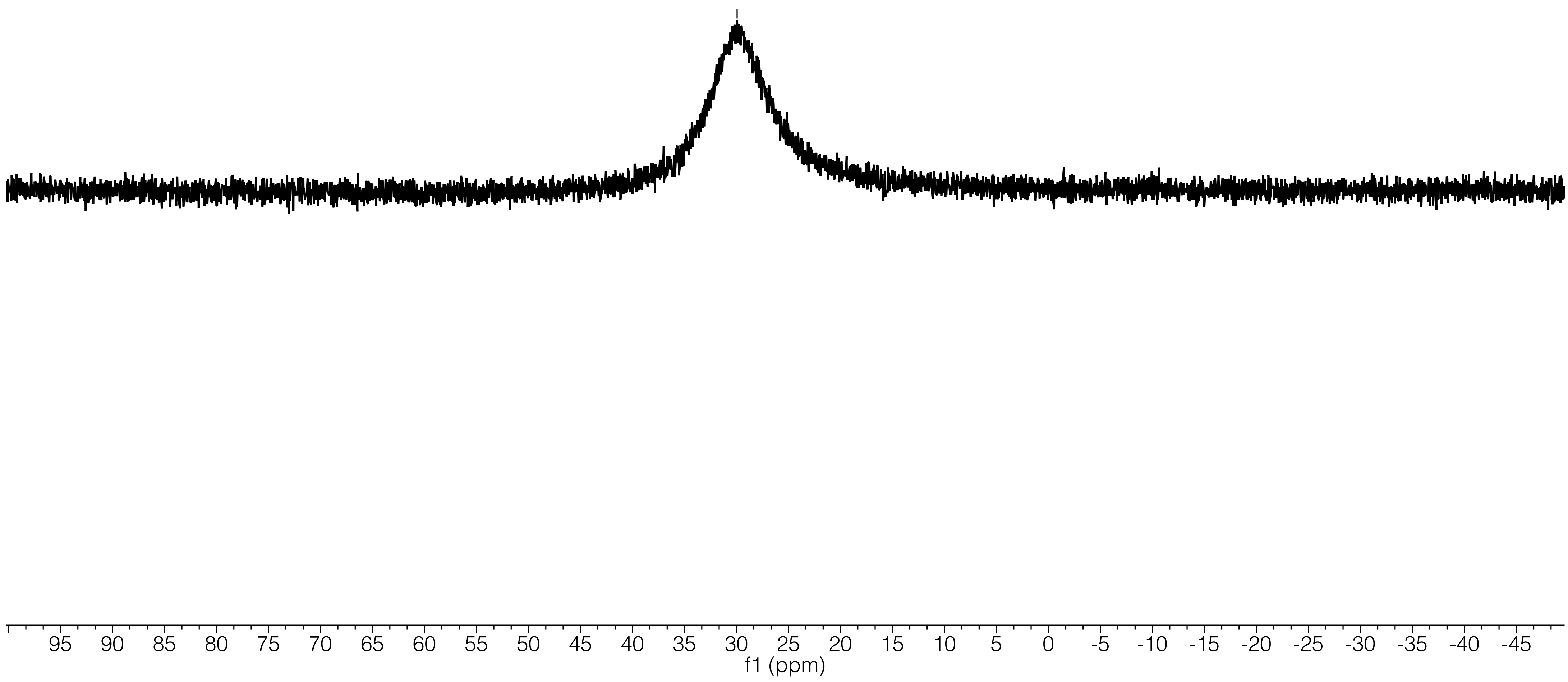

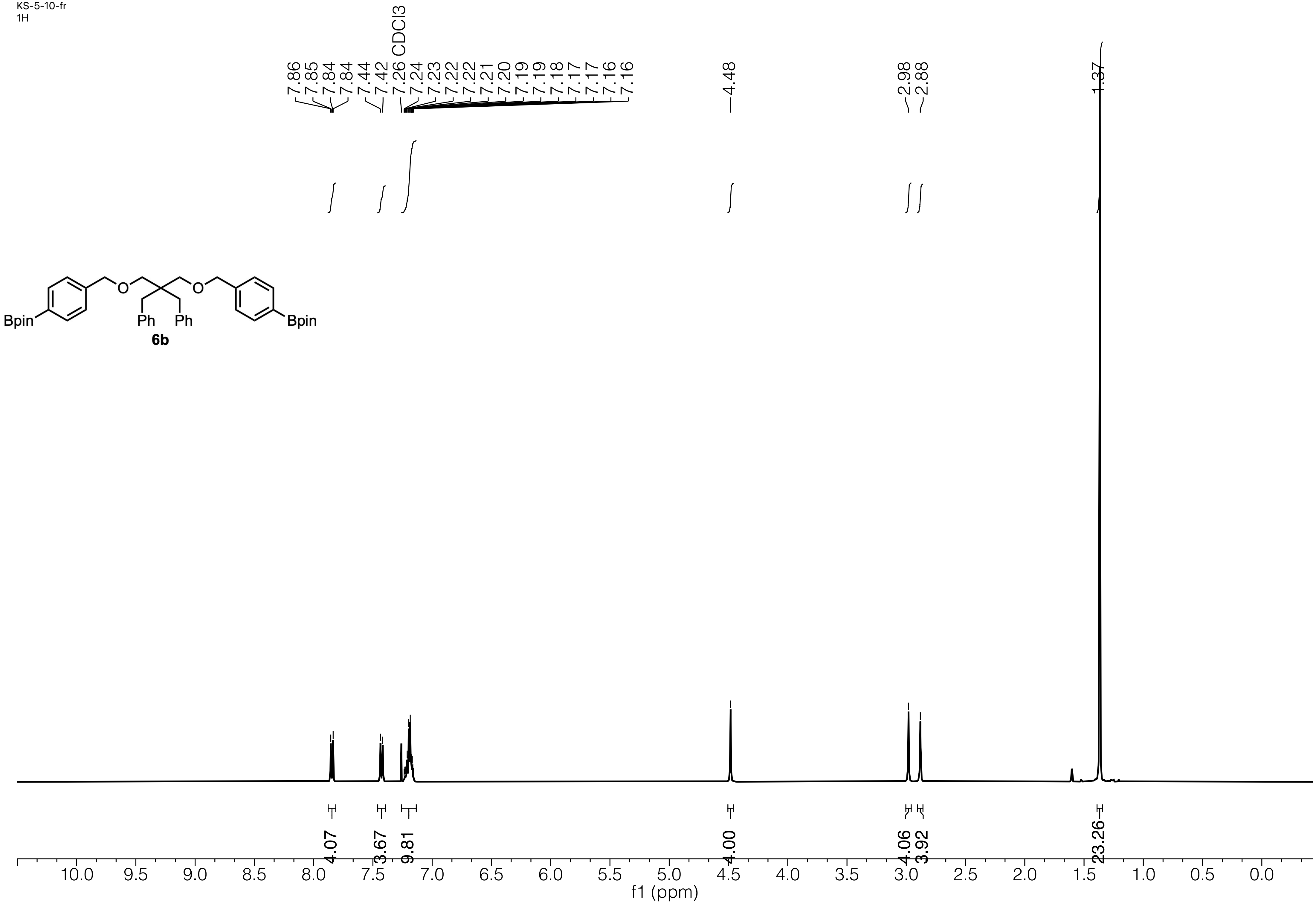
KS-5-10-fr
1H
7.86
7.85
7.84
7.84
7.44
7.42
7.26 CDCl3
7.24
7.23
7.22
7.22
7.21
7.20
7.19
7.19
7.18
7.17
7.17
7.16
7.16
4.48
2.98
2.88
1.37
Bpin
O
O
Ph
Ph
Bpin
6b
4.07
3.67
9.81
4.00
4.06
3.92
23.26
10.0
9.5
9.0
8.5
8.0
7.5
7.0
6.5
6.0
5.5
5.0
4.5
4.0
3.5
3.0
2.5
2.0
1.5
1.0
0.5
0.0
f1 (ppm)

KS-5-10-fr
13C

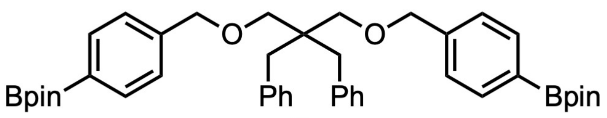

6b

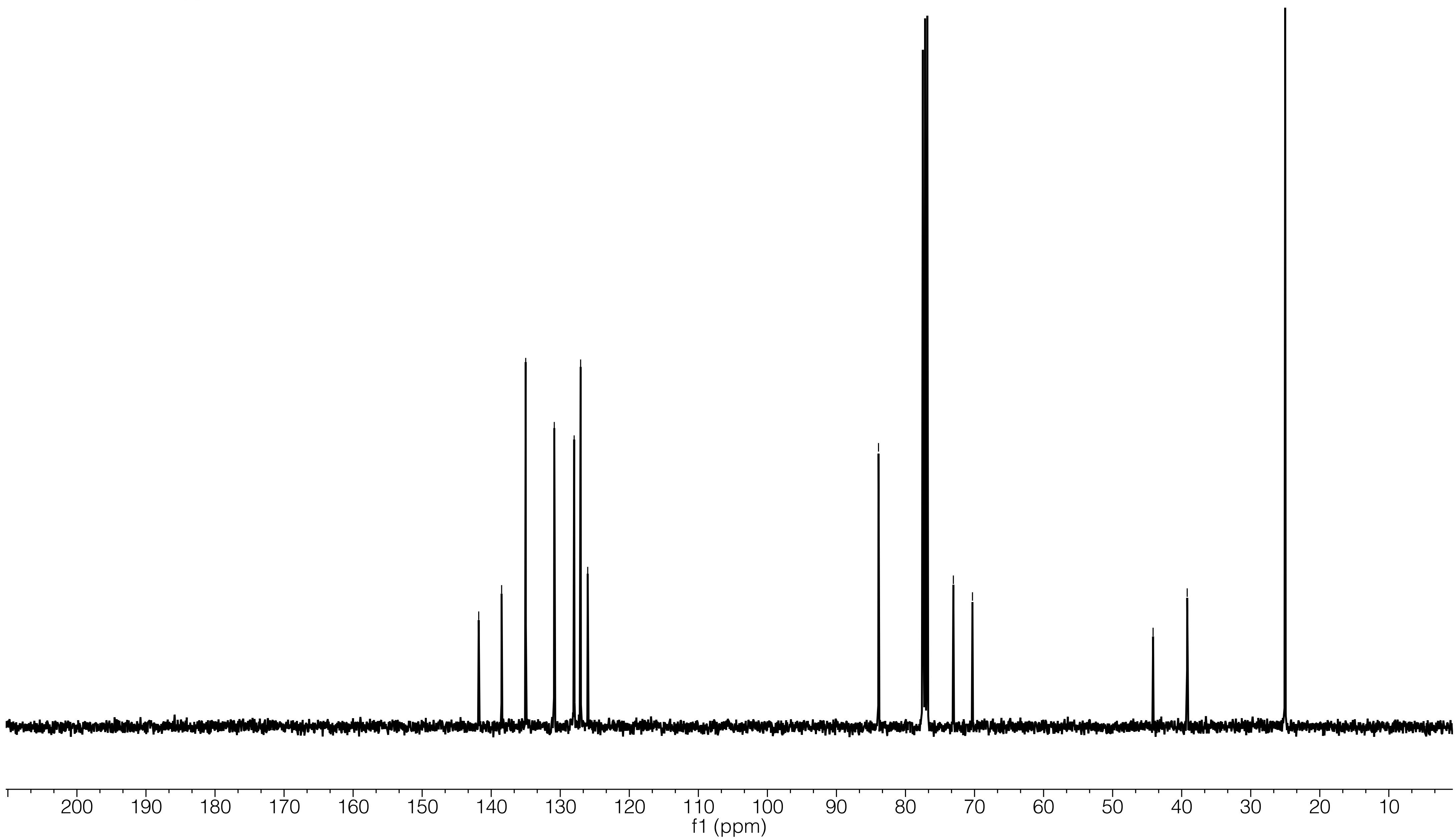

KS-5-10-fr
11B_No irr

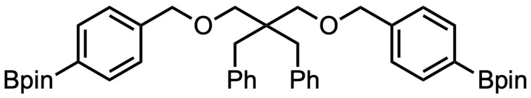

6b

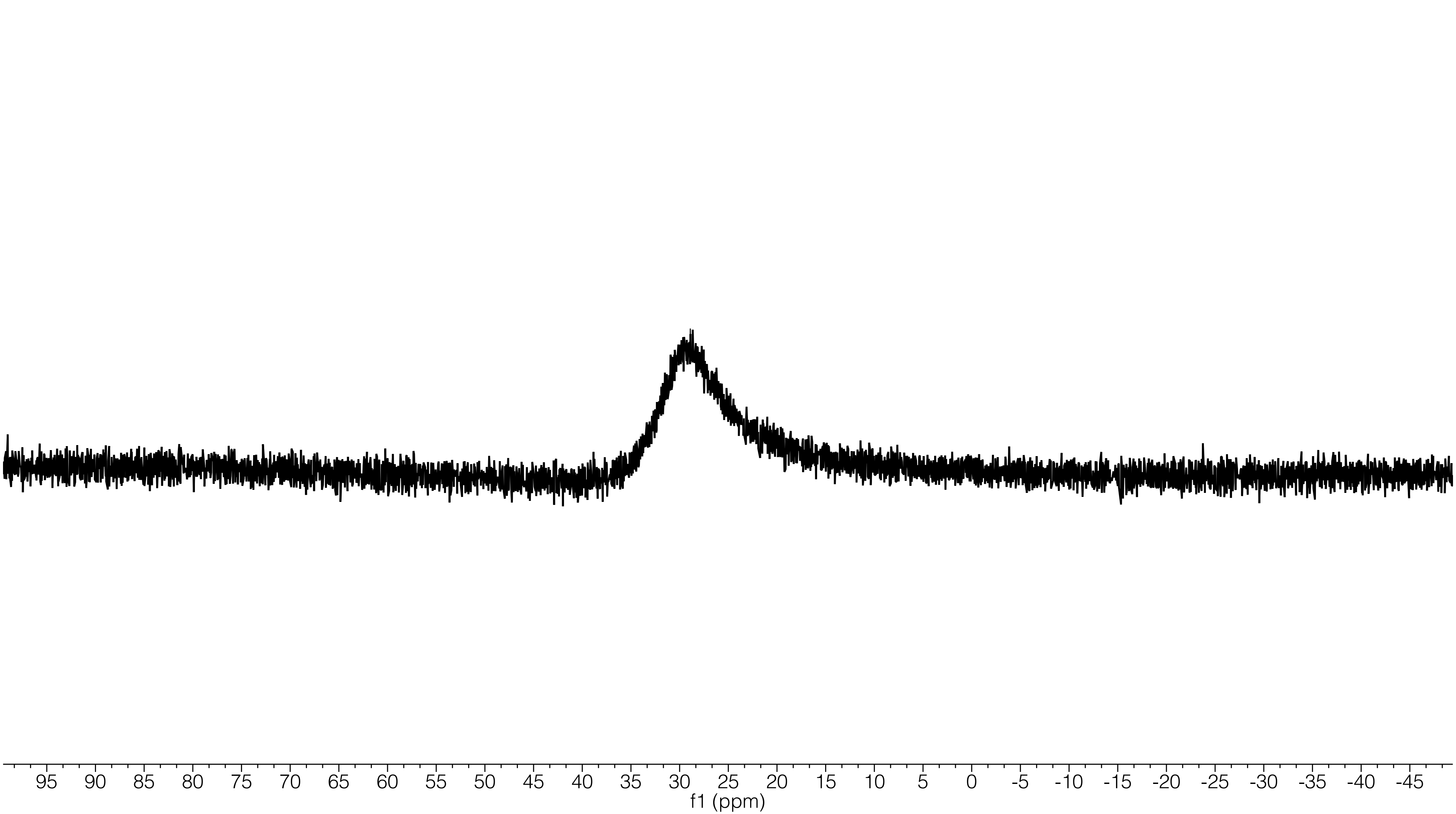

KS-2-68-GPC2
1H

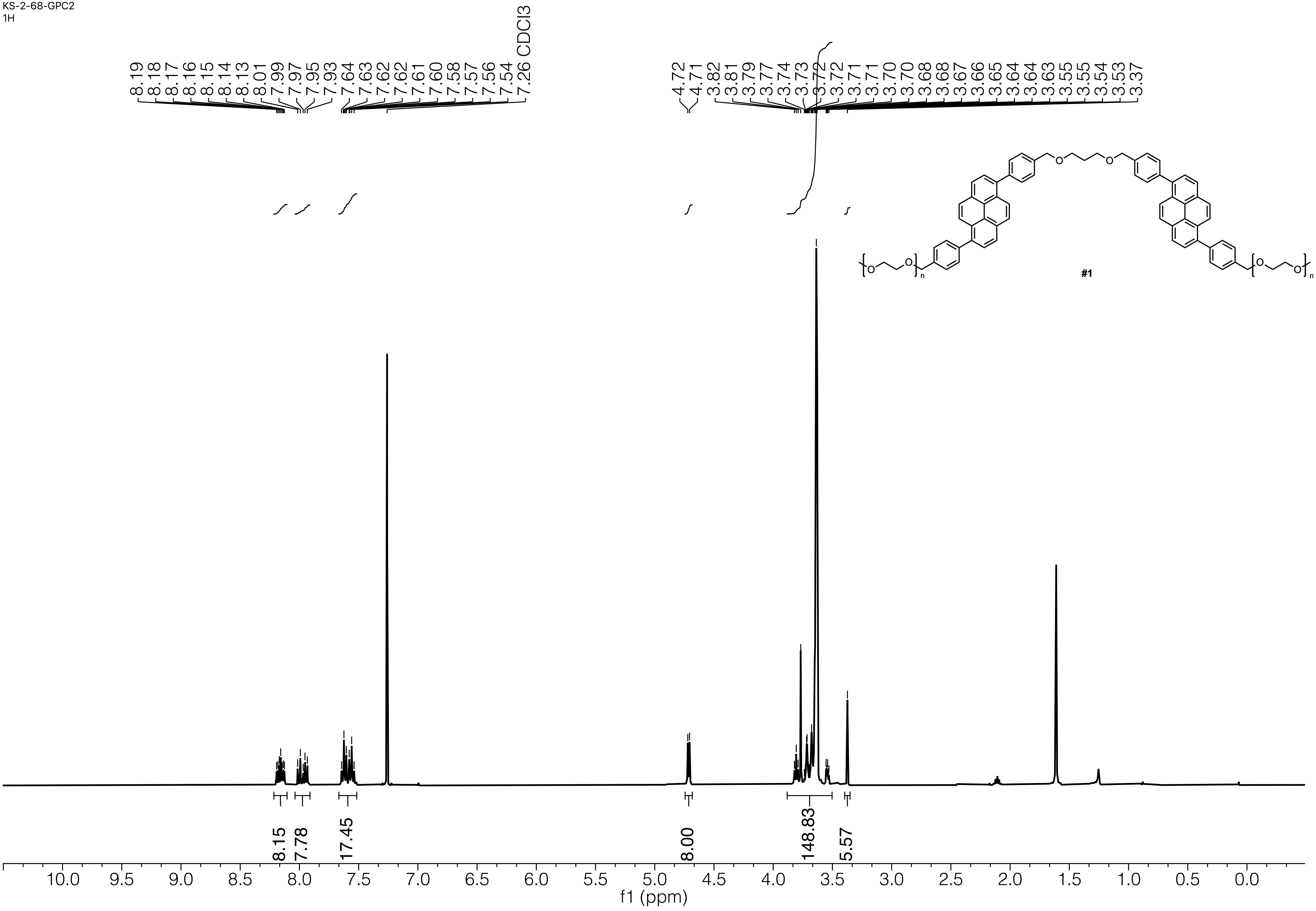

KS-2-68 13C
13C

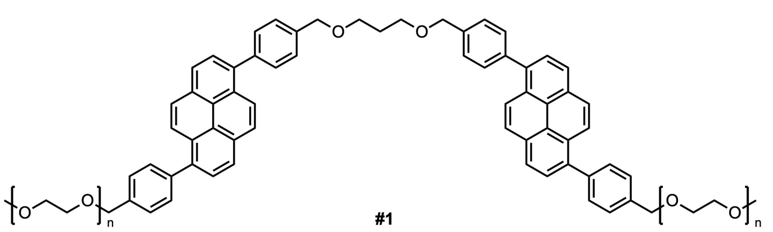

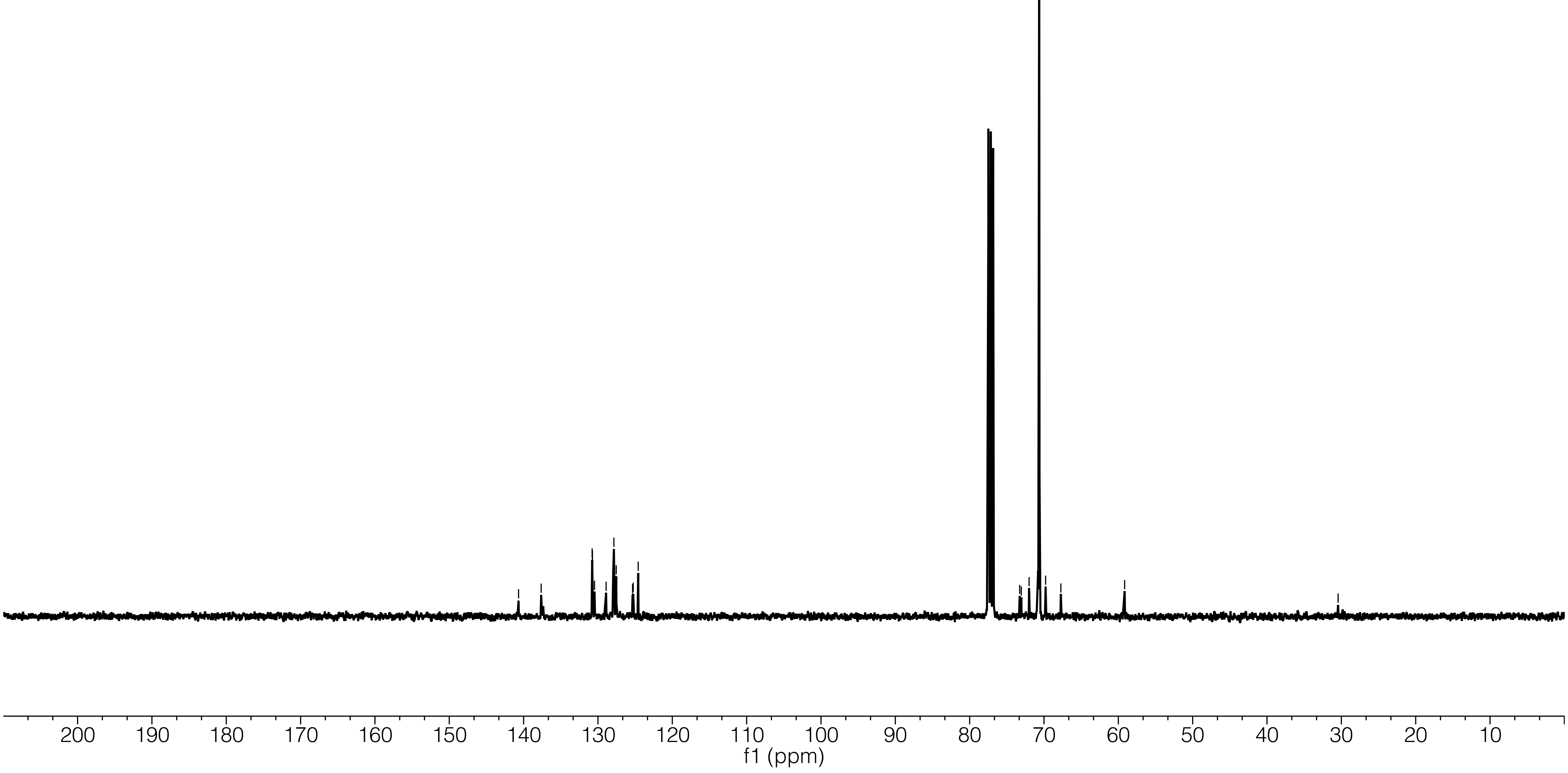

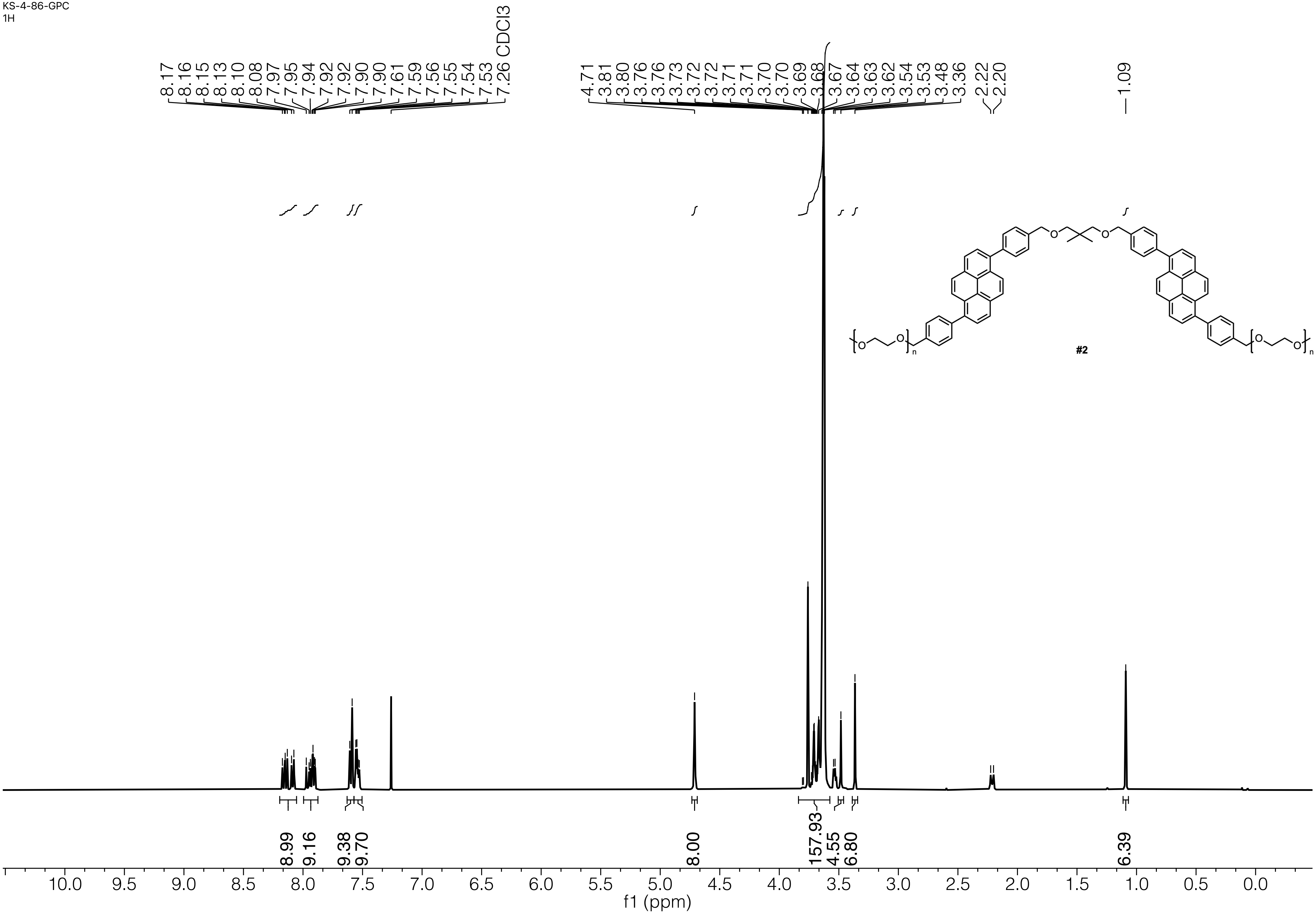

KS-4-86-GPC
1H
8.17 8.16 8.15 8.13 8.10 8.08 7.97 7.95 7.94 7.92 7.90 7.90 7.61 7.59 7.56 7.55 7.54 7.53 7.26 CDCl3
4.71 3.81 3.80 3.76 3.76 3.73 3.72 3.72 3.71 3.71 3.70 3.70 3.69 3.68 3.67 3.64 3.63 3.62 3.54 3.53 3.48 3.36 2.22 2.20
1.09
#2
n
8.99 9.16 9.38 9.70 8.00 157.93 4.55 6.80 6.39
10.0 9.5 9.0 8.5 8.0 7.5 7.0 6.5 6.0 5.5 5.0 4.5 4.0 3.5 3.0 2.5 2.0 1.5 1.0 0.5 0.0
f1 (ppm)

KS-4-86-GPC
13C

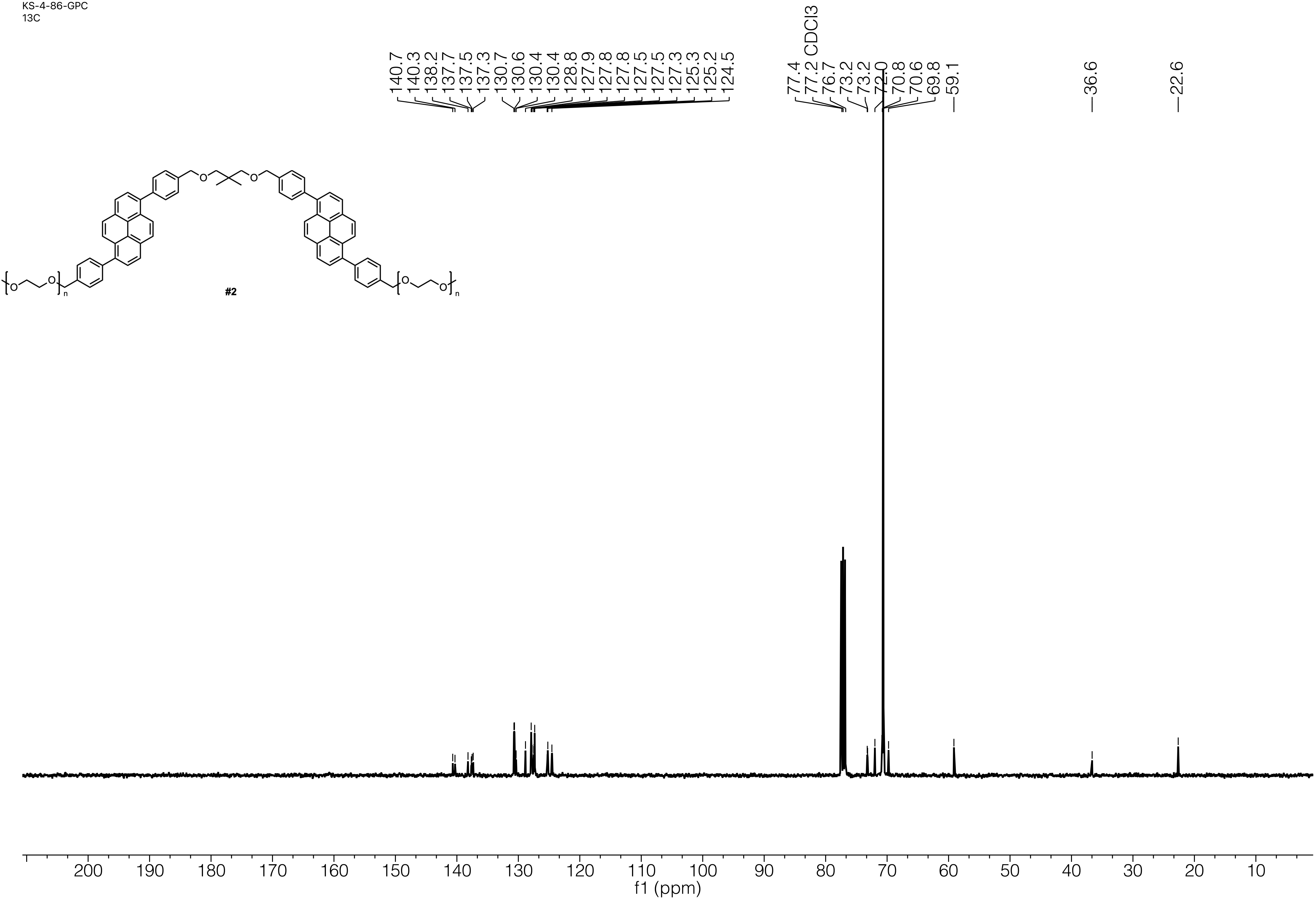

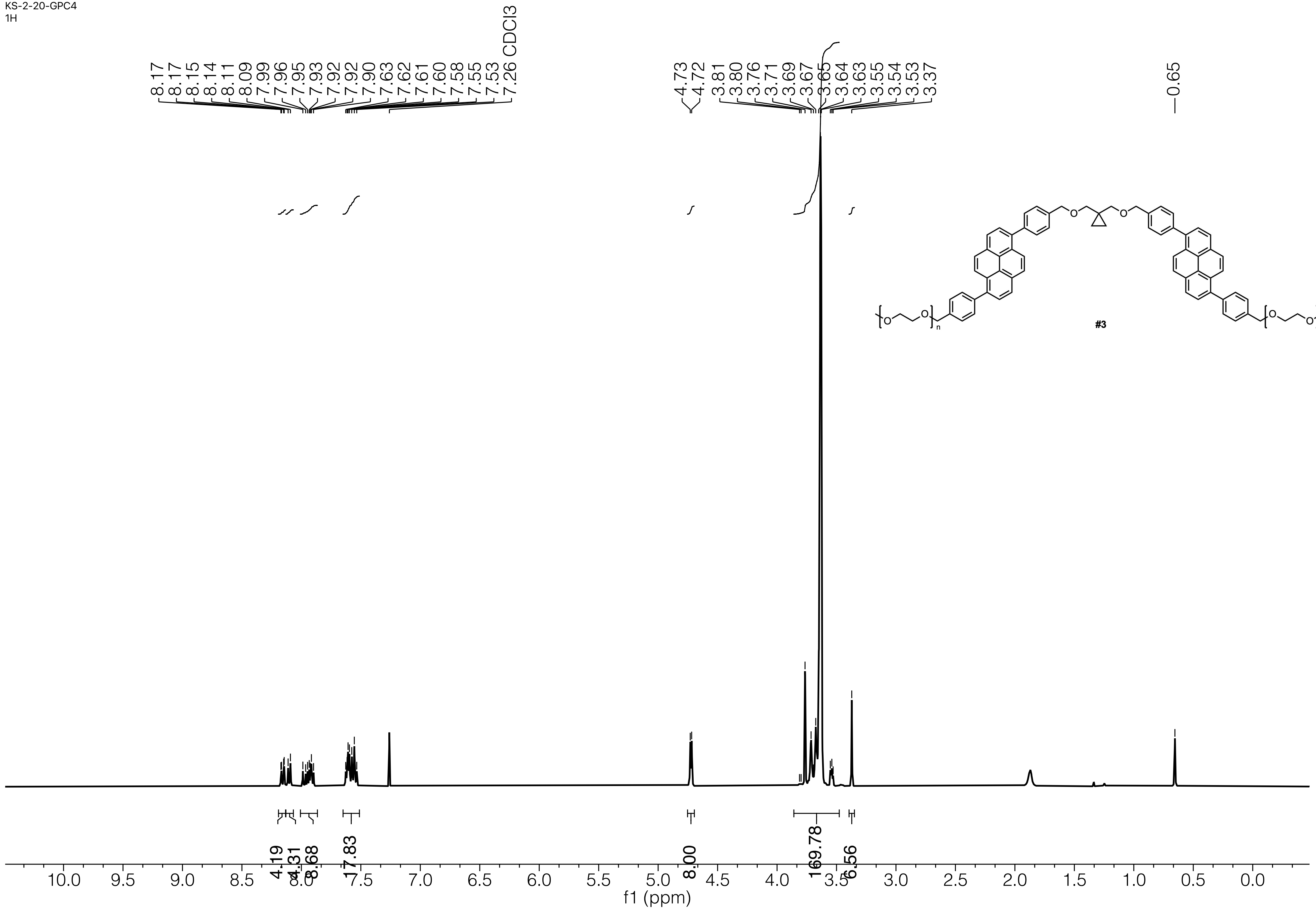
KS-2-20-GPC4
1H
8.17 8.17 8.15 8.14 8.11 8.09 7.99 7.96 7.95 7.93 7.92 7.92 7.90 7.63 7.62 7.61 7.60 7.58 7.55 7.53 7.26 CDCl3
4.73 4.72
3.81 3.80 3.76 3.71 3.69 3.67 3.65 3.64 3.63 3.55 3.54 3.53 3.37
0.65
#3
4.19
4.31
8.68
17.83
8.00
169.78
6.56
10.0 9.5 9.0 8.5 8.0 7.5 7.0 6.5 6.0 5.5 5.0 4.5 4.0 3.5 3.0 2.5 2.0 1.5 1.0 0.5 0.0
f1 (ppm)

KS-2-20 13C
13C

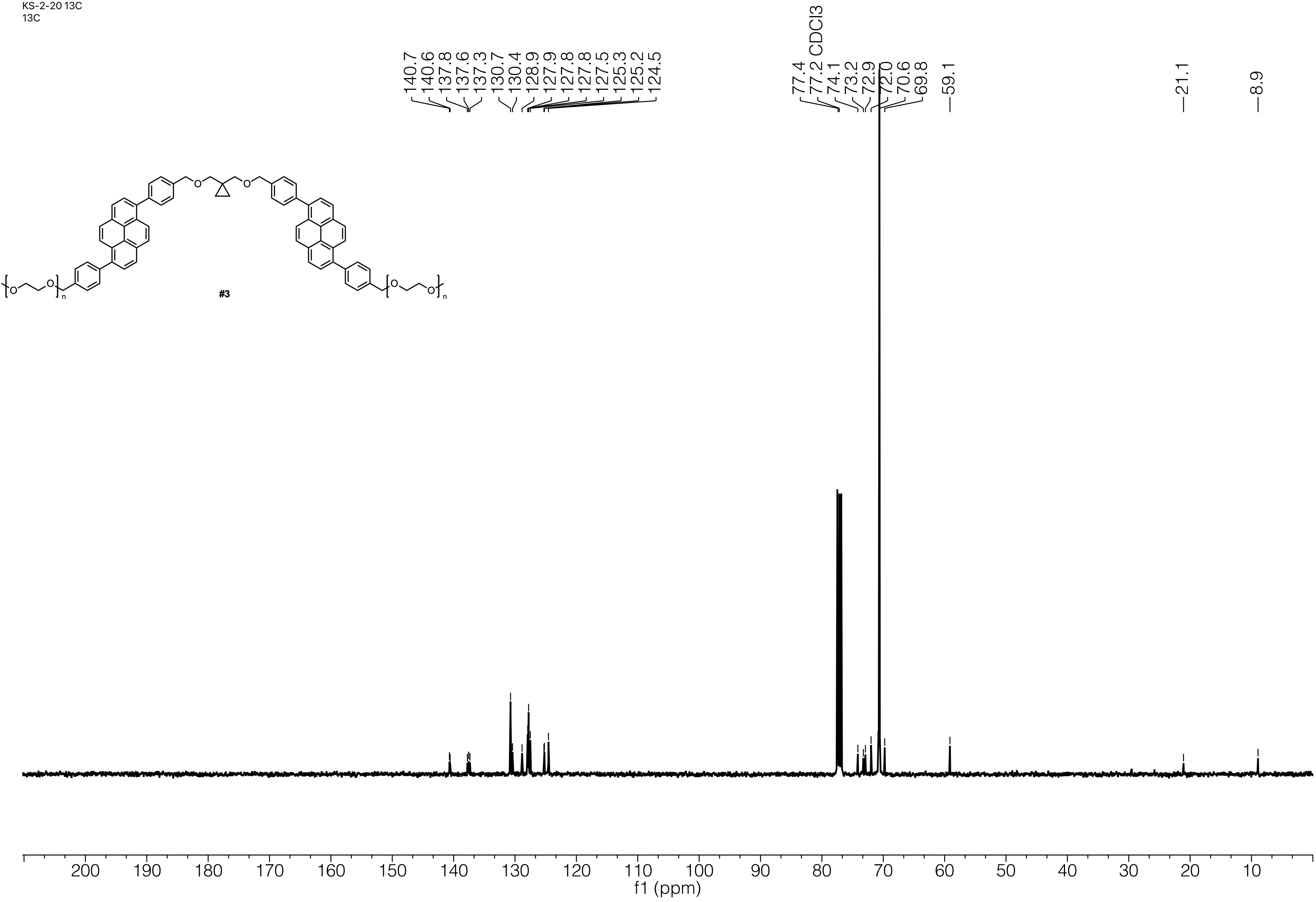

KS-5-5-GPC3
1H

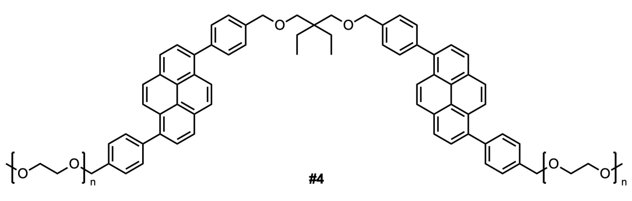
#4

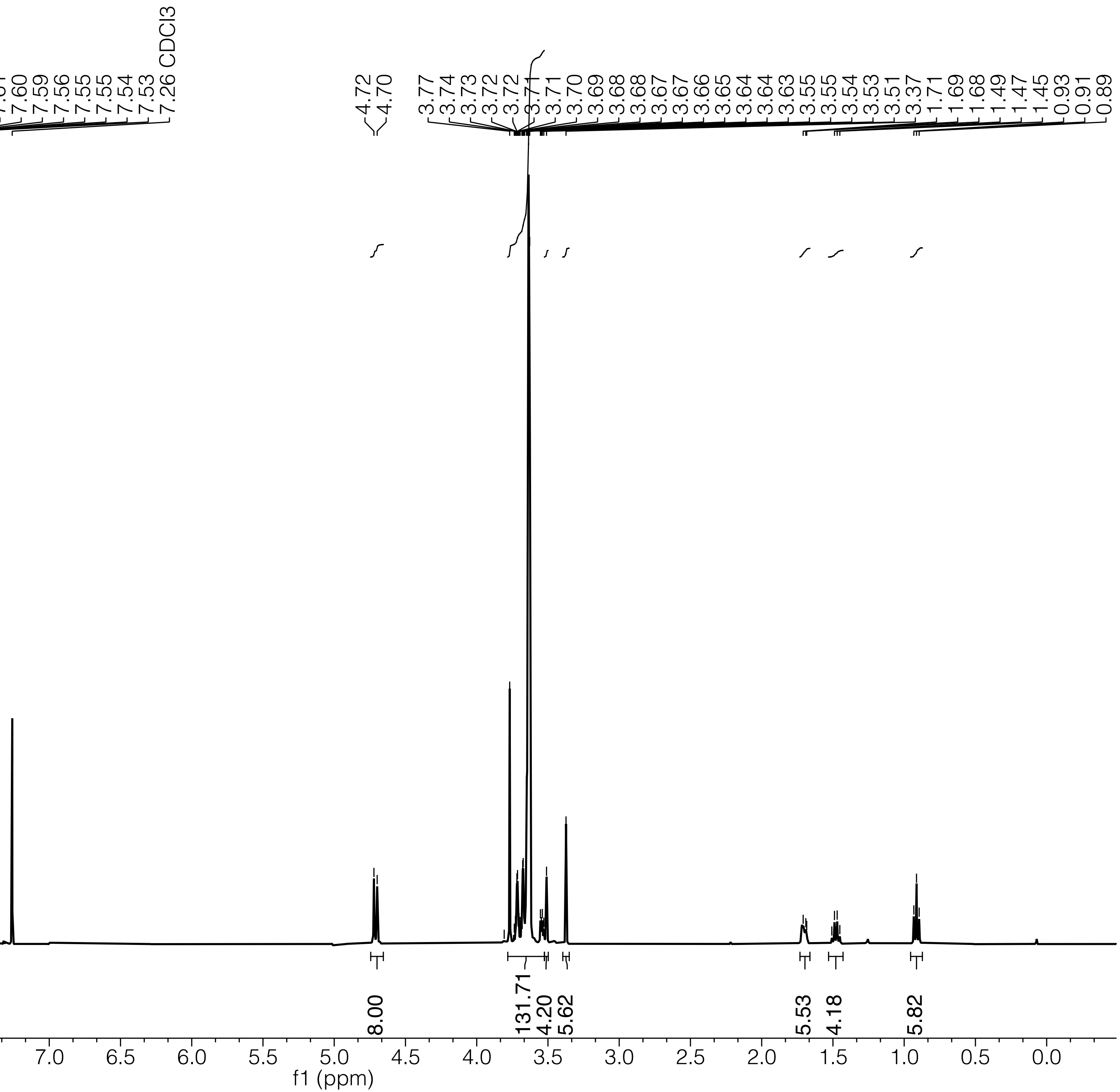

KS-5-5
13C

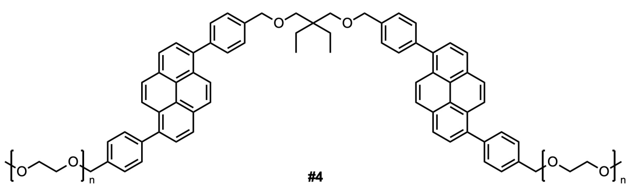
#4

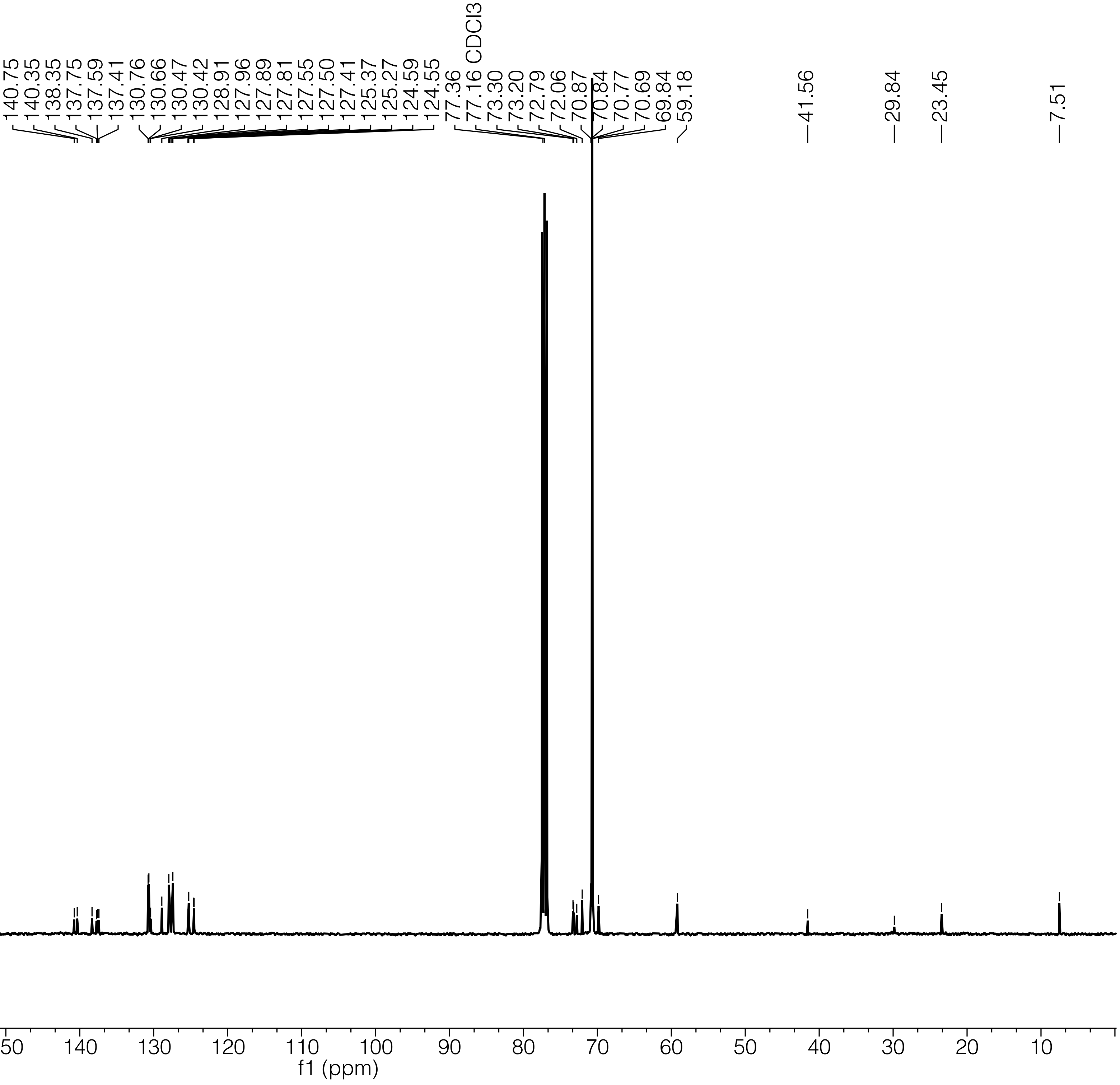
140.75
140.35
138.35
137.75
137.59
137.41
130.76
130.66
130.47
130.42
128.91
127.96
127.89
127.81
127.55
127.50
127.41
125.37
125.27
124.59
124.55
77.36
77.16 CDCl3
73.30
73.20
72.79
72.06
70.87
70.84
70.77
70.69
69.84
59.18
41.56
29.84
23.45
7.51
200
190
180
170
160
150
140
130
120
110
100
90
80
70
60
50
40
30
20
10
f1 (ppm)

KS-5-15-GPC3
1H

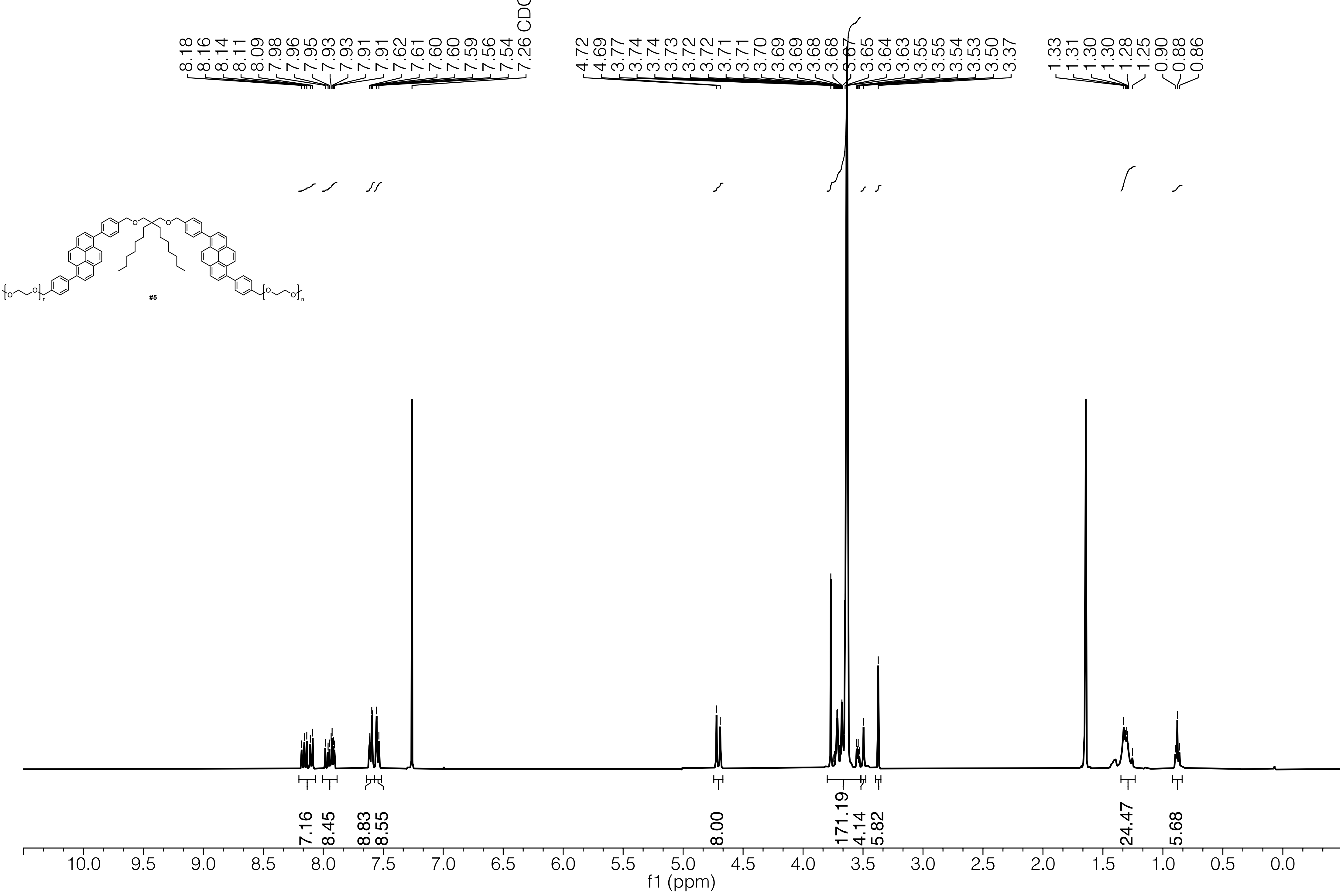

KS-5-5
13C

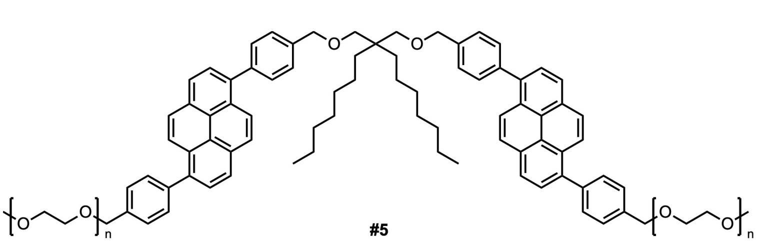

#5

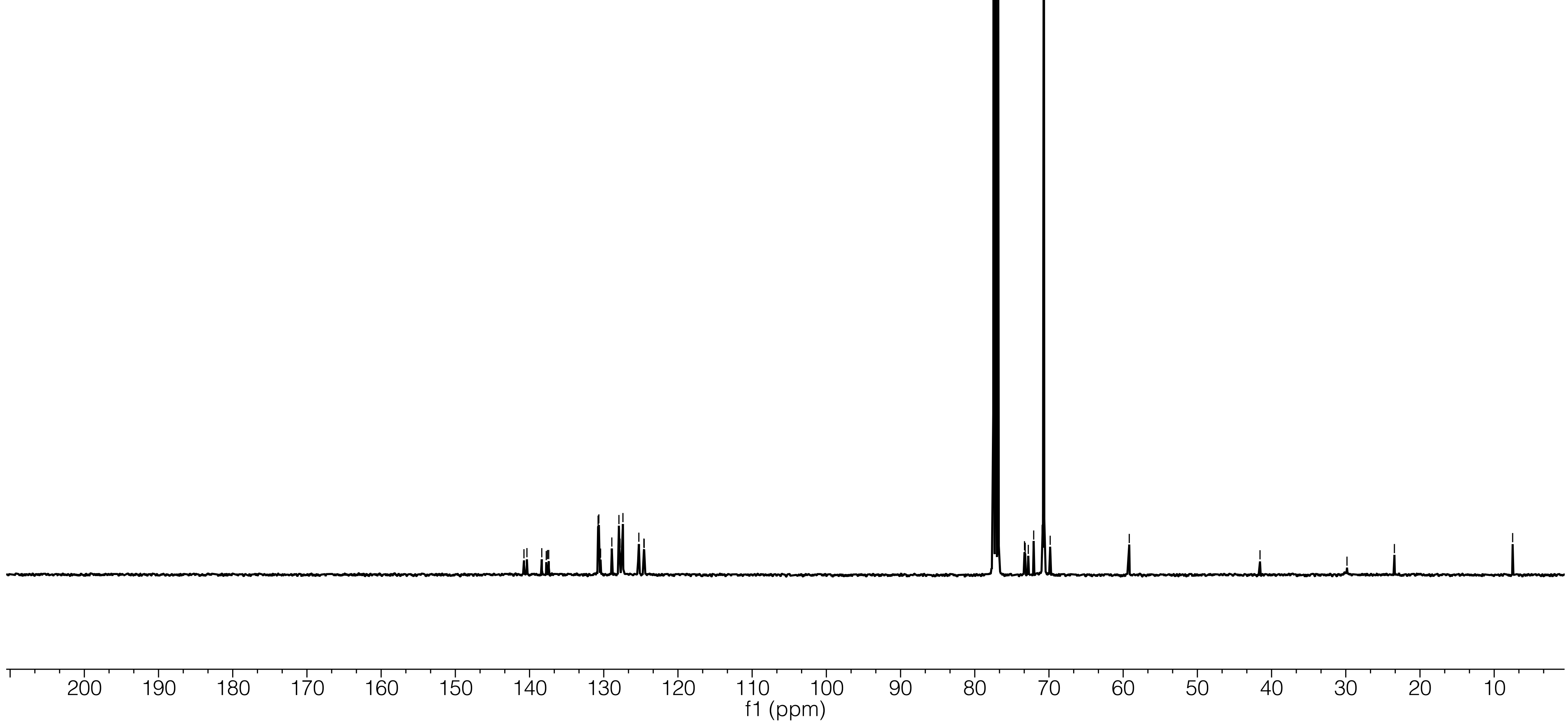

140.8
140.4
138.4
137.7
137.6
137.4
130.8
130.7
130.5
130.4
128.9
128.0
127.9
127.8
127.5
127.5
127.4
125.4
125.3
124.6
124.6
77.4
77.2 CDCl3
73.3
73.2
72.8
72.1
70.9
70.8
70.8
70.7
69.8
59.2
41.6
29.8
23.4
7.5
200 190 180 170 160 150 140 130 120 110 100 90 80 70 60 50 40 30 20 10
f1 (ppm)

KS-5-5-GPC3
1H

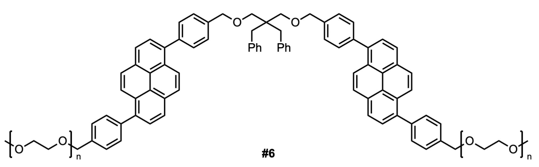
Ph
Ph
#6
n
n

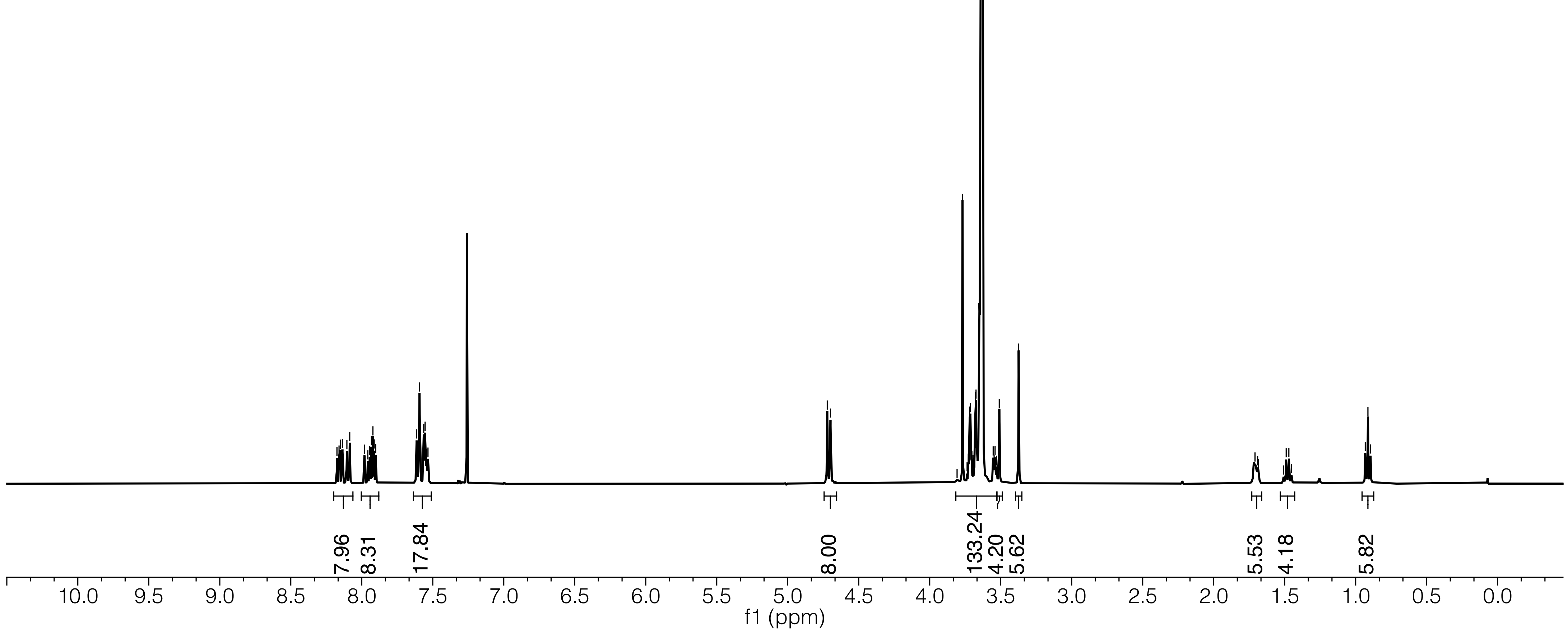
8.17
8.16
8.15
8.14
8.10
8.08
7.98
7.96
7.94
7.93
7.92
7.91
7.90
7.61
7.60
7.59
7.56
7.55
7.55
7.54
7.53
7.26 CDCl3
4.72
4.70
3.77
3.74
3.73
3.72
3.72
3.71
3.71
3.70
3.69
3.68
3.68
3.67
3.67
3.66
3.65
3.64
3.64
3.63
3.55
3.55
3.54
3.53
3.51
3.37
1.71
1.69
1.68
1.49
1.47
1.45
0.93
0.91
0.89
7.96
8.31
17.84
8.00
133.24
4.20
5.62
5.53
4.18
5.82
10.0
9.5
9.0
8.5
8.0
7.5
7.0
6.5
6.0
5.5
5.0
4.5
4.0
3.5
3.0
2.5
2.0
1.5
1.0
0.5
0.0
f1 (ppm)

KS-5-15
13C

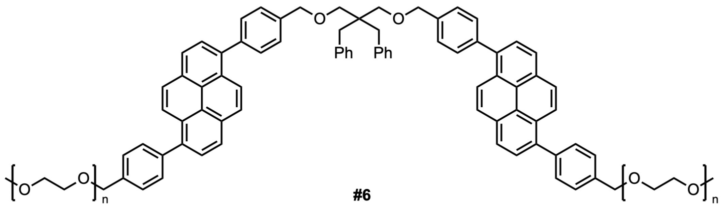

#6

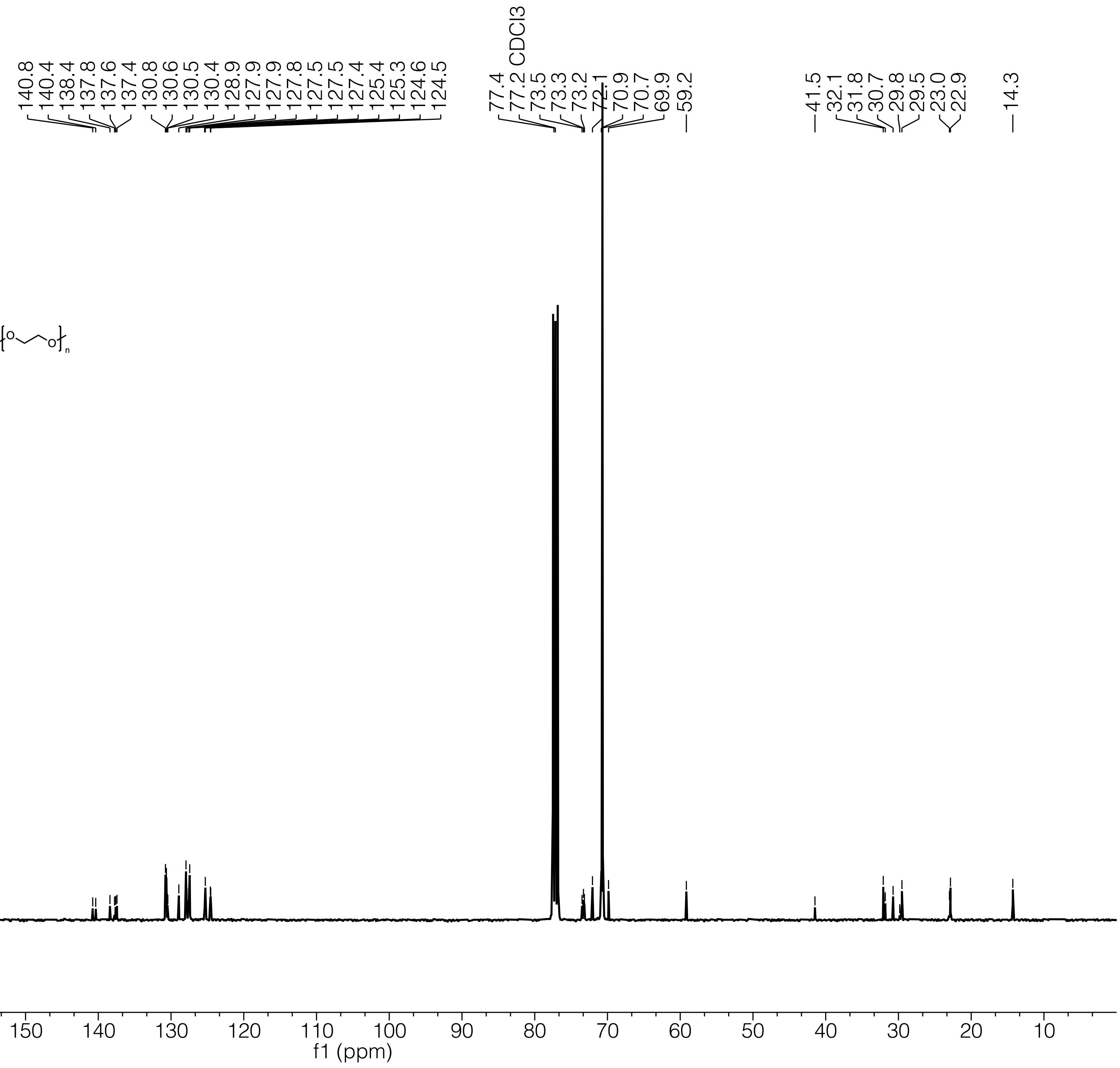
140.8
140.4
138.4
137.8
137.6
137.4
130.8
130.6
130.5
130.4
128.9
127.9
127.9
127.8
127.5
127.5
127.4
125.4
125.3
124.6
124.5
77.4
77.2 CDCl3
73.5
73.3
73.2
72.1
70.9
70.7
69.9
59.2
41.5
32.1
31.8
30.7
29.8
29.5
23.0
22.9
14.3
200 190 180 170 160 150 140 130 120 110 100 90 80 70 60 50 40 30 20 10
f1 (ppm)

260512-PyM-1H-CDCl3-2-ok.1.fid

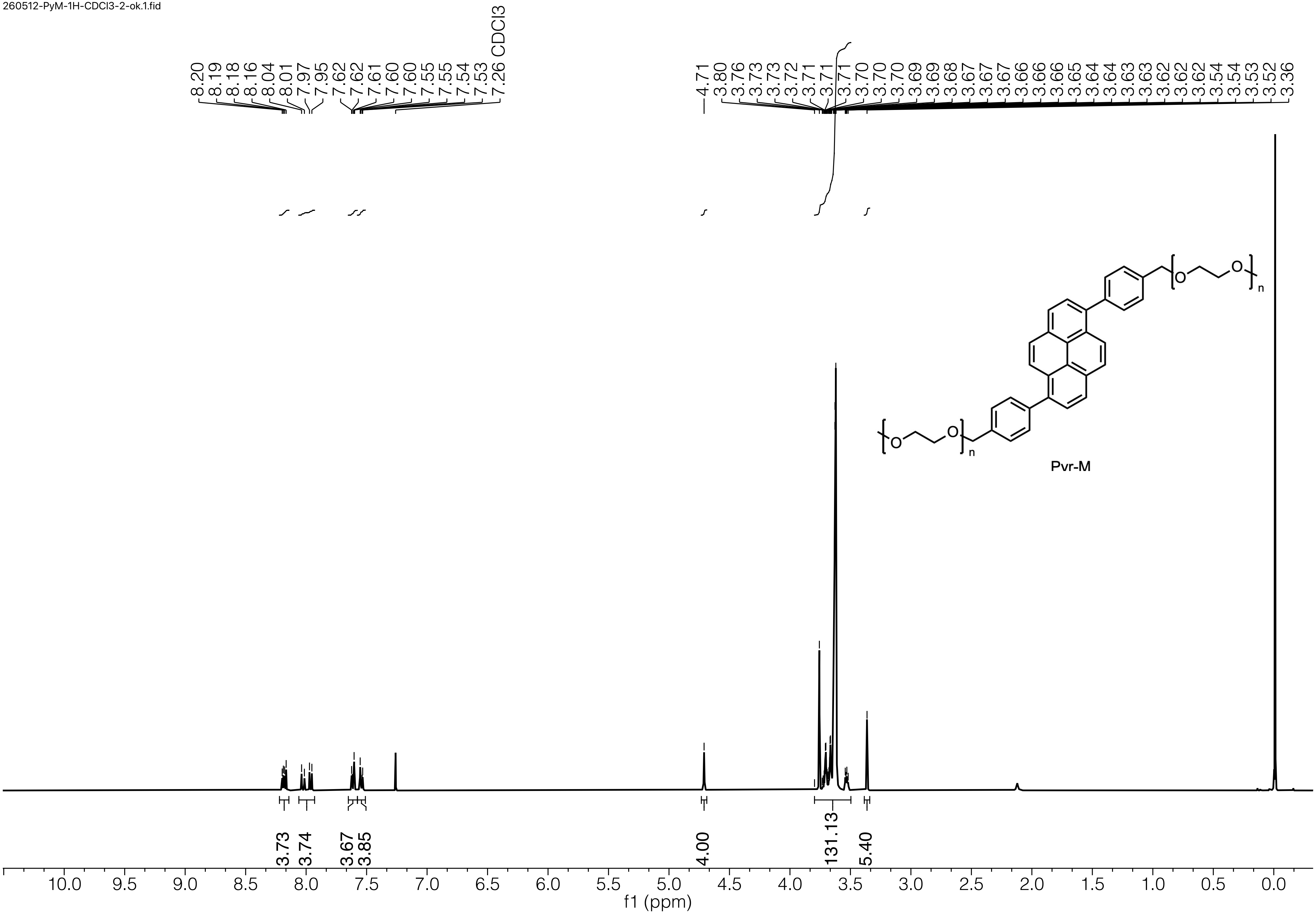

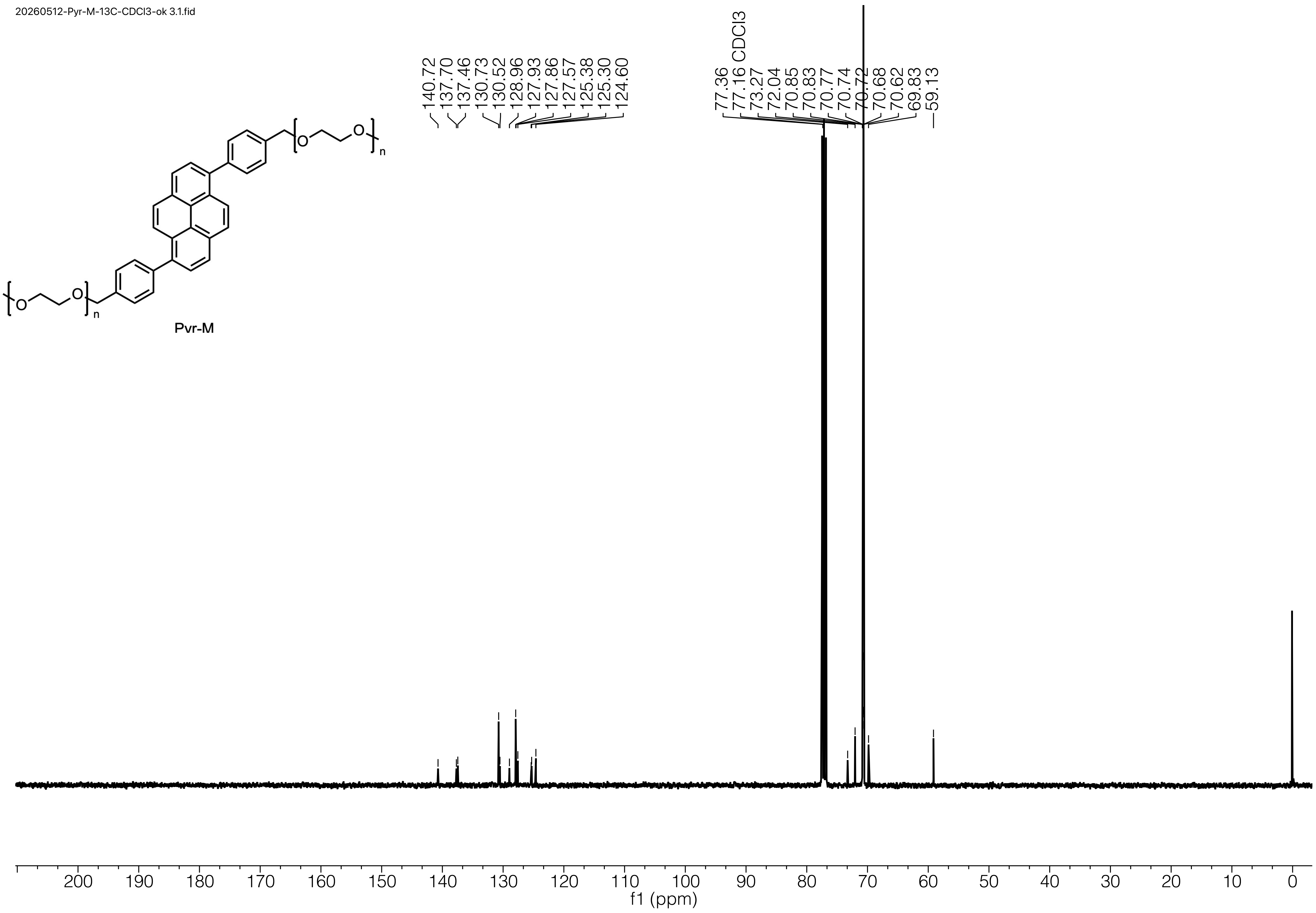
20260512-Pyr-M-13C-CDCl3-ok 3.1.fid
140.72
137.70
137.46
130.73
130.52
128.96
127.93
127.86
127.57
125.38
125.30
124.60
77.36
77.16 CDCl3
73.27
72.04
70.85
70.83
70.77
70.74
70.72
70.68
70.62
69.83
59.13
Pyr-M
n
n
200 190 180 170 160 150 140 130 120 110 100 90 80 70 60 50 40 30 20 10 0
f1 (ppm)

Supplementary Figure S2. NMR spectra of synthesized compounds.

^1H, ^13C, and, where applicable, ^11B NMR spectra of the synthesized dibromo precursors **1a–6a**, bis(pinacol boronate) derivatives **1b–6b**, polymers **#1–#6**, and **Pyr-M**. Spectra were recorded in CDCl3 unless otherwise noted; the ^1H NMR spectrum of **6a** was recorded in acetone-d6. Residual solvent peaks are indicated in the spectra.
